# Multivalent, Stabilized Mannose‐6‐Phosphates for the Targeted Delivery of Toll‐Like Receptor Ligands and Peptide Antigens

**DOI:** 10.1002/cbic.202000538

**Published:** 2020-10-23

**Authors:** Niels R. M. Reintjens, Elena Tondini, Christopher Vis, Toroa McGlinn, Nico J. Meeuwenoord, Tim P. Hogervorst, Herman S. Overkleeft, Dmitri V. Filippov, Gijsbert A. van der Marel, Ferry Ossendorp, Jeroen D. C. Codée

**Affiliations:** ^1^ Leiden Institute of Chemistry Leiden University Einsteinweg 55 2333 CC Leiden Netherlands; ^2^ Department of Immunology Leiden University Medical Center Leiden University Albinusdreef 2 2333 ZA Leiden Netherlands

**Keywords:** C-glycoside, mannose-6-phosphate receptor, peptide conjugates, solid-phase peptide synthesis, TLR ligands

## Abstract

Mannose‐6‐phosphate (M6P) is recognized by the mannose‐6‐phosphate receptor and plays an important role in the transport of cargo to the endosomes, making it an attractive tool to improve endosomal trafficking of vaccines. We describe herein the assembly of peptide antigen conjugates carrying clusters of mannose‐6‐*C*‐phosphonates (M6Po). The M6Po's are stable M6P mimics that are resistant to cleavage of the phosphate group by endogenous phosphatases. Two different strategies for the incorporation of the M6Po clusters in the conjugate have been developed: the first relies on a “post‐assembly” click approach employing an M6Po bearing an alkyne functionality; the second hinges on an M6Po *C*‐glycoside amino acid building block that can be used in solid‐phase peptide synthesis. The generated conjugates were further equipped with a TLR7 ligand to stimulate dendritic cell (DC) maturation. While antigen presentation is hindered by the presence of the M6Po clusters, the incorporation of the M6Po clusters leads to increased activation of DCs, thus demonstrating their potential in improving vaccine adjuvanticity by intraendosomally active TLR ligands.

## Graphical abstract

**Enhancing the immune response**: Two mannose‐6‐*C*‐phosphonate (M6Po) building blocks have been synthesized that can be incorporated into a conjugate by a “post‐assembly” click or an online SPPS approach to improve endosomal trafficking of vaccines. The generated M6Po conjugates, which were also equipped with a TLR7 ligand, were able to enhance the immune response by activation and maturation of dendritic cells.

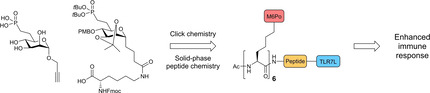

## Introduction

Carbohydrates play an important role in many biological processes, such as cell‐cell communication, pathogen recognition and protein folding. Mannose‐6‐phosphate (M6P), a d‐mannopyranose bearing a phosphate group at the C‐6 position, serves as a signaling moiety on the termini of glycan branches mounted on newly synthesized proteins in the trans‐Golgi network and is essential for the transportation of these proteins to the late endosomes and lysosomes. The mannose‐6‐phosphate receptor (MPR), a P‐type lectin, plays an important role in this transportation through binding to M6P.[^1^, ^2^] There are two members of this lectin family: the cation‐dependent mannose‐6‐phosphate receptor (CD‐MPR) and the cation‐independent mannose‐6‐phosphate receptor (CI‐MPR). The latter has a high affinity for ligands containing multiple M6Ps due to the presence of two M6P binding domains, which can simultaneously bind two M6Ps.[^3^, ^4^, ^5^] A small fraction of MPRs can be found on the cell surface of which only CI‐MPR binds and internalizes M6P‐bound substrates.^[6]^ Therefore, the CI‐MPR is an efficient tool for targeted delivery to the endosomes, as was shown by the conjugation of M6P analogues to acid α‐glucosidase leading to improved delivery of this enzyme in the treatment of the lysosomal myopathy Pompe disease.^[7]^ The MPR has also been exploited as a drug delivery system for cancer therapy,[^8^, ^9^] for example, doxorubicin was delivered via mannose‐6‐phosphate‐modified human serum albumin as carrier and *N*‐hexanoyl‐d‐*erythro*‐sphingosine with M6P‐functionalized liposomes.[^10^, ^11^] Our group has previously reported that a cathepsin inhibitor that is covalently attached to an M6P cluster could effectively be delivered into the endolysosomal pathway.^[12]^

The activity of peptide‐based anti‐cancer vaccines may be enhanced by the targeted delivery of these antigens to antigen presenting cells. In this context, mannosylated antigens, destined for the mannose receptor or DC‐SIGN present on dendritic cells (DCs), have been widely explored.[^13^, ^14^, ^15^, ^16^] We, therefore, reasoned that conjugate vaccines in which an M6P moiety is covalently bound to an antigenic peptide might be targeted effectively to immune cells expressing the MPR, leading to improved uptake and more efficient delivery to the lysosomes where the cargo is processed for MHC loading, ultimately resulting in enhanced antigen presentation. Since dephosphorylation by endogenous phosphatases is one of the potential drawbacks of the use of M6P, several stable M6P analogues have previously been evaluated, such as a malonyl ether, a malonate or an isosteric *C*‐phosphonate ester.[^17^, ^18^] The *C*‐phosphonate proved to be a stable and effective replacement for the phosphate monoester.^[19]^

**Figure 1:**
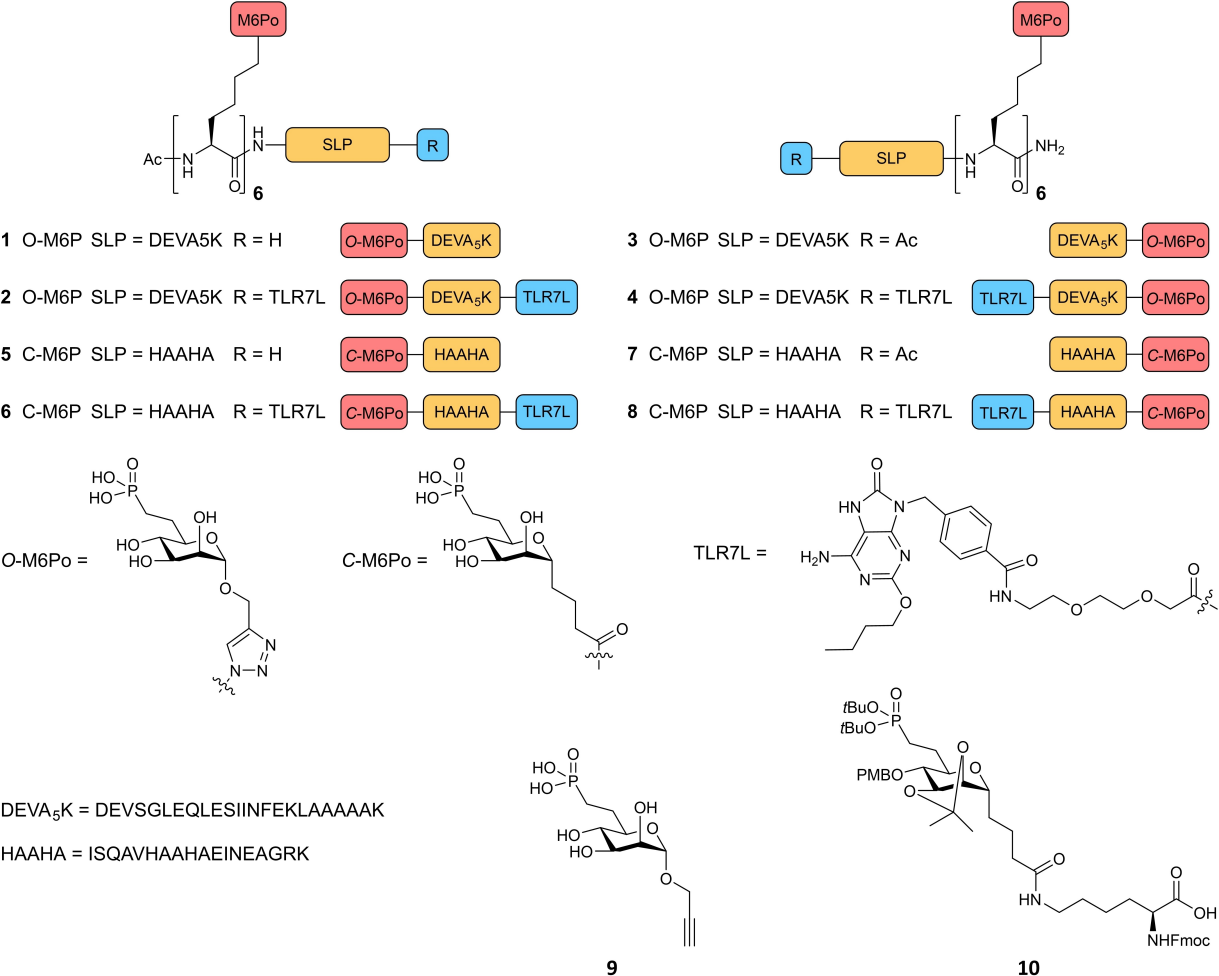
Structures of the *O*‐M6Po conjugates **1**–**4**, *C*‐M6Po conjugates **5**–**8**, and building blocks **9** and **10**.

We here describe the incorporation of a cluster of mannose‐6‐phosphonates (M6Po) in two types of peptide antigen‐conjugates (**1**–**8**, Figure 1), wherein two different M6Po building blocks, **9** and **10**, respectively, are used to construct M6Po‐clusters and conjugate them to either the N‐ or the C‐terminus of a synthetic long peptide (SLP). In this study, two different ovalbumin derived SLPs are used: DEVA_5_K (DEVSGLEQLESIINFEKLAAAAAK), which harbours the MHC‐I presented epitope SIINFEKL, and HAAHA (ISQAVHAAHAEINEAGRK), which contains an MHC‐II epitope. Our goal is to enhance the immune response by increasing endosomal trafficking of the peptide vaccine via the MPR which led to the design of bis‐conjugates (**2**, **4**, **6**, and **8**) in which the Toll‐like receptor 7 ligand (TLR7L) 2‐alkoxy‐8‐oxo‐adenine is added at either the N‐ or the C‐terminus of the M6Po‐SLP.[^20^, ^21^] We coupled an α‐configured spacer,[^12^, ^18^, ^22^] carrying an alkyne function to the *O*‐M6Po building block **9** to allow for the copper mediated 1,3‐dipolar cycloaddition to azide functions incorporated in the SLP, while *C*‐M6Po building block **10** was used to generate the HAAHA‐conjugates via solid‐phase peptide synthesis (SPPS). This building block is a *C*‐analogue^[23]^ of *O*‐M6Po, in which the anomeric oxygen is replaced with a CH_2_, preventing hydrolysis under the acidic conditions used in SPPS. An additional advantage of this SPPS‐compatible building block is the possibility to prepare conjugates of peptides that are not suitable for copper mediated 1,3‐dipolar cycloaddition. It also allows one to incorporate azide or alkyne click handles in conjugate vaccine constructs that can be exploited for labelling or visualization purposes.

We report herein the synthesis and immunological evaluation of these dual conjugated peptide vaccine constructs.

## Results and Discussion

### Synthesis of M6Po‐conjugates

**Scheme 1:**
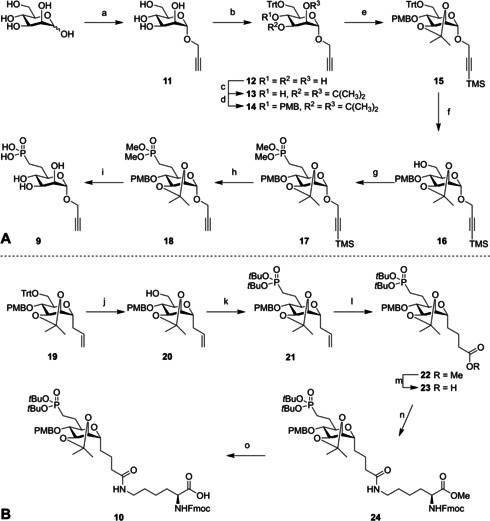
Synthesis of alkyne building blocks **9** and **10**. a) i: Ac_2_O, pyridine; ii: propargyl alcohol, BF_3_ ⋅ OEt_2_, 50 °C; iii: NaOMe, MeOH, 70 % over three steps; b) TrtCl, Et_3_N, DMF, 60 °C, 83 %; c) *p*‐toluenesulfonic acid, 2,2‐dimethoxypropane, 87 %; d) *p*‐methoxybenzyl chloride, NaH, DMF, 95 %; e) TMSCl, *n*BuLi, THF, −78 °C, 97 %; f) i: *p*‐toluenesulfonic acid, CH_2_Cl_2_/MeOH; ii: *p*‐toluenesulfonic acid, 2,2‐dimethoxypropane; iii: 1 M HCl, EtOAc, 0 °C, 98 % over three steps: g) i: Tf_2_O, pyridine, CH_2_Cl_2_, −40 °C; ii: *n*BuLi, dimethyl methylphosphonate, THF, −70 to −50 °C, 72 % over two steps; h) TBAF, THF, quant.; i) i: TMSBr, pyridine, MeCN; ii: AcOH/H_2_O, 90 °C, 81 % over two steps; j) i: *p*‐toluenesulfonic acid, CH_2_Cl_2_/MeOH; ii: *p*‐toluenesulfonic acid, 2,2‐dimethoxypropane; iii: 1 M HCl, EtOAc, 0 °C, 75 % over three steps; k) i: Tf_2_O, pyridine, CH_2_Cl_2_, −40 °C; ii: *n*BuLi, di‐*tert*‐butyl methylphosphonate, THF, −70 to −50 °C, 72 % over two steps; l) i: methyl acrylate, CuI, Grubbs 2nd‐gen. catalyst, 1,2‐dichloroethene (DCE), 60 °C; ii: NaBH_4_, RuCl_3_, MeOH, DCE, 45 °C, 72 % over two steps; m) LiOH, THF/H_2_O, quant; n) Fmoc‐l‐Lys‐OMe, HCTU, DIPEA, DMF, 86 %; o) LiOH, THF/H_2_O, 0 °C, 80 %.

The first type of *O*‐M6Po conjugates comprises the sixfold addition of α‐propargyl mannose‐6‐phosphonate (*O*‐M6Po) building block (**9**) to azide containing peptides. Synthesis of the required building block **9** started from propargyl‐d‐mannose **11** (Scheme 1A). As we found that the use of *para*‐methoxybenzyl ethers for the protection of the secondary alcohols led to the formation of a 3,6 ether bridge when the C‐6 hydroxy group was triflated, to enable the subsequent substitution by a phosphonate anion (see Scheme S1 in the Supporting Information), we placed an isopropylidene group over the 2,3‐*cis*‐diol system to prevent this intramolecular side reaction.[^24^, ^25^] Thus, tritylation of **11** and subsequent installation of the isopropylidene gave **13**, of which the remaining alcohol was masked as a *p*‐methoxybenzyl ether to give the fully protected mannose **14**. The alkyne in **14** was protected with a TMS group using TMSCl and *n*BuLi at −78 °C. Removal of the trityl in the thus obtained **15** with a catalytic amount of *p*‐toluenesulfonic acid in CH_2_Cl_2_/MeOH was accompanied by partial removal of the isopropylidene ketal. Reinstallation of the isopropylidene and subsequent deprotection of the mixed ketal simultaneously formed on the primary alcohol gave **16** in 98 % over three steps. Alcohol **16** was treated with Tf_2_O and pyridine at −40 °C^[26]^ and the obtained crude triflate was added to a mixture of dimethyl methylphosphonate and *n*BuLi in THF at −70 °C, yielding compound **17** in 72 % over two steps. Removal of the TMS protecting group gave **18**, which was transformed into key building block **9** by a two‐step deprotection sequence. The deprotection of the phosphonate using TMSBr was followed by the removal of the *p*‐methoxybenzyl and isopropylidene groups by treatment with AcOH/H_2_O at 90 °C to deliver key building block **9** in 27 % yield over 15 steps starting from d‐mannose.

En route to mannose‐6‐phosphonate SPPS building block **10** (Scheme 1B), the trityl group of known compound **19** ^[23]^ was removed as described above to give alcohol **20** in 72 % over three steps. Conversion of **20** to the primary triflate, followed by nucleophilic substitution with the anion of di‐*tert*‐butyl methylphosphonate^[27]^ gave phosphonate **21** in 72 % over two steps on 3 mmol scale.^[28]^ Cross metathesis with methyl acrylate, followed by the reduction of the double bond with NaBH_4_ and ruthenium trichloride then resulted in compound **22**.[^29^, ^30^] Hydrolysis of the obtained methyl ester, was followed by condensation with Fmoc‐l‐Lys‐OMe and subsequent treatment of **24** with LiOH at 0 °C, which left the Fmoc group unaffected, then delivered the key SPPS building block **10** in 80 % yield.

**Scheme 2:**
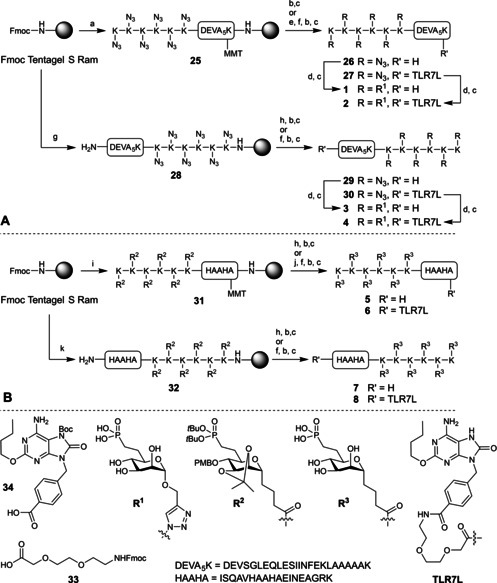
Synthesis of A) *O*‐M6Po conjugates **1**–**4** and B) *C*‐M6Po conjugates **5**–**8**. a) i: 20 % piperidine, DMF; ii: Fmoc SPPS cycle for K(N_3_)‐K(N_3_)‐K(N_3_)‐K(N_3_)‐K(N_3_)‐K(N_3_)‐DEVSGLEQLESIINFEKLAAAAAK; iii: 20 % piperidine, DMF; iv: Ac_2_O, DIPEA, DMF; b) TFA/TIS/H_2_O (95 : 2.5 : 2.5, *v*/*v*/*v*), 3 h; c) RP‐HPLC; d) **9**, 20 mM Tris/150 mM NaCl buffer, CuSO_4_/NaAsc/TBTA, H_2_O/DMSO; e) TFA/TIS/CH_2_Cl_2_ (2 : 2 : 96, *v*/*v*/*v*); f) i: (2‐(2‐(Fmoc‐amino)ethoxy)ethoxy)acetic acid, HCTU, DIPEA, DMF; ii: 20 % piperidine, DMF; iii: 4‐((2‐butoxy‐6‐((*tert*‐butoxycarbonyl)amino)‐8‐oxo‐7,8‐dihydro‐9*H*‐purin‐9‐yl)methyl)benzoid acid, HCTU, DIPEA, DMF; g) i: 20 % piperidine, DMF; ii: Fmoc SPPS cycle for DEVSGLEQLESIINFEKLAAAAAK‐K(N_3_)‐K(N_3_)‐K(N_3_)‐K(N_3_)‐K(N_3_)‐K(N_3_); iii: 20 % piperidine, DMF; h) Ac_2_O, DIPEA, DMF; i) i: 20 % piperidine, DMF; ii: Fmoc SPPS cycle for ISQAVHAAHAEINEAGRK; iii: 20 % piperidine, DMF; iv: **10**, HCTU, DIPEA, DMF; v: 5× repeat of iii and iv; vi: 20 % piperidine, DMF; vii: Ac_2_O, DIPEA, DMF; j) AcOH/TFE/CH_2_Cl_2_ (1 : 2 : 7, *v*/*v*/*v*); k) i: 20 % piperidine, DMF; ii: **10**, HCTU, DIPEA, DMF; iii: 5× repeat of i and ii; iv: 20 % piperidine, DMF; v: Fmoc SPPS cycle for ISQAVHAAHAEINEAGRK; vi: 20 % piperidine, DMF. Yield peptides and conjugates: **26**) 4.6 mg, 1 %; **27**) 4.0 mg, 2 %; **29**) 17.4 mg, 6 %; **30**) 8.2 mg, 2 %; **1**) 0.3 mg, 5 %; **2**) 1.0 mg, 18 %; **3**) 0.9 mg, 18 %; **4**) 3.3 mg, 31 %; **5**) 13.3 mg, 10 %; **6**) 11.0 mg, 8 %; **7**) 3.1 mg, 2 %; **8**) 17.0 mg, 11 %.

Next, the assembly of the (*O*‐M6Po)_6_‐SIINFEKL conjugates was undertaken. Immobilized peptides **25** and **28** were prepared through standard SPPS HCTU/Fmoc chemistry using Tentagel S Ram as solid support (Scheme 2A). The C‐terminal lysine of **25** was protected with a MMT group since we aimed to make M6Po‐peptide conjugates as well as conjugates containing both an M6Po‐cluster and a TLR7 ligand. TFA/TIS/H_2_O (95 : 2.5 : 2.5, *v*/*v*/*v*) treatment removed all protecting groups and cleaved the peptides from the resin to give peptides **26** and **29** in 1 and 6 % yield, respectively, after purification. Alternatively, the MMT protecting group at the C‐terminal lysine of **25** was selectively deprotected using a cocktail of TFA/TIS/CH_2_Cl_2_ (2 : 2 : 96, *v*/*v*/*v*). The released amine was subsequently coupled with the spacer **33** and the Boc‐protected TLR7 ligand building block **34**.^[21]^ After deprotection, release from the resin and RP‐HPLC purification peptides **27** and **30** were obtained in a 2 % yield. Coupling of *O*‐M6Po building block **9** to peptides **26**, **27**, **29** and **30** was performed using a cocktail of CuSO_4_, sodium ascorbate and tris(benzyltriazolylmethyl)amine in DMSO/H_2_O,^[31]^ with the addition of a 20 mM Tris/150 mM NaCl buffer. After RP‐HPLC, conjugates **1**–**4** were obtained in 5 % (0.3 mg), 18 % (0.9 mg), 18 % (1.0 mg) and 31 % (3.3 mg) yield respectively.

The HAAHA peptide contains two histidines, which can coordinate to copper and thereby inhibit the reduction of Cu^II^ to Cu^I^.^[32]^ Therefore, conjugates **5**–**8** were generated by an online SPPS synthesis (Scheme 2B) using *C‐*M6Po building block **10**, which is equipped with acid‐labile protecting groups that can be removed at the end of the SPPS concomitantly with all other acid‐labile peptide protecting groups and release of the peptide from the resin. Tentagel S Ram resin was elongated with ISQAVHAAHAEINEAGRK using automated SPPS, of which the lysine(MMT) at the C terminus will be used for elongation at a later stage of the synthesis. Six consecutive coupling and Fmoc removal cycles with building block **10** gave immobilized peptide **31**. Peptide **32**, bearing the *C‐*M6Po cluster at the C‐terminal end, was generated by assembling the hexa‐*C‐*M6Po peptide through manual couplings of building block **10**, followed by automated SPPS to assemble the rest of the peptide. Immobilized and protected peptides **31** and **32** were deprotected and simultaneously cleaved from the resin with the TFA/TIS cocktail to provide conjugates **5** (13.3 mg) and **7** (3.1 mg) after purification by RP‐HPLC in 10 and 8 % yield, respectively, demonstrating the suitability of **10** for SPPS. To obtain conjugate **6**, bearing the TLR7 ligand, the MMT group in **31** was selectively removed with a cocktail of AcOH/TFE/CH_2_Cl_2_ (1 : 2 : 7, *v*/*v*/*v*). The obtained free amine was elongated with spacer **33** and Boc‐protected TLR7 ligand building block **34** to give conjugate **6** (11.0 mg) in 2 % yield, after removal of all the protecting groups, cleavage from the resin and RP‐HPLC purification. The same sequence of events was applied to the N‐terminal amine in immobilized peptide **32** to afford conjugate **8** (17.0 mg) in 8 % yield.

### Immunological evaluation

**Figure 2:**
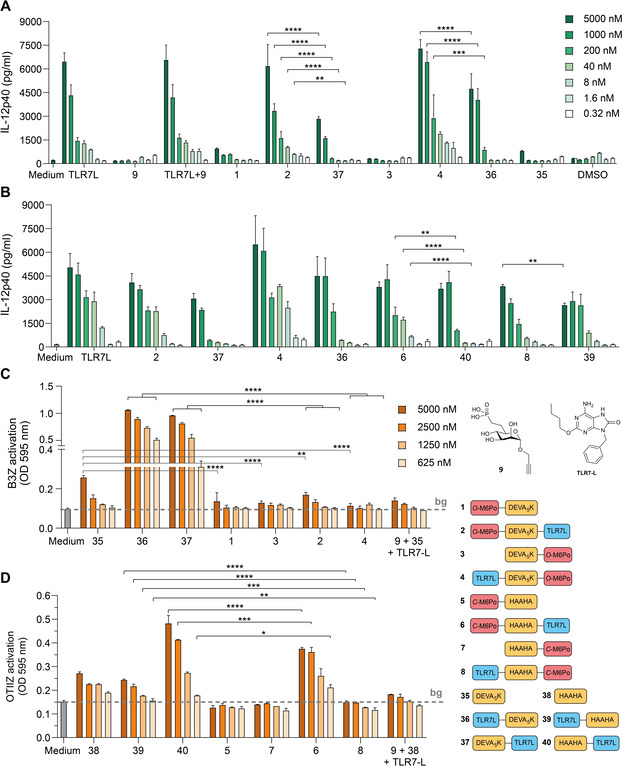
M6Po conjugation enhances DC activation of the TLR7 ligand conjugates but inhibits antigen presentation. A) Murine bone‐marrow‐derived dendritic cells were stimulated in triplicate for 24 h with the indicated conjugates of the DEVA_5_K peptide. The induction of DC maturation was analyzed by measuring IL‐12 production with sandwich ELISA specific for the IL‐12p40 subunit. The bars represent the standard deviation of the mean of the triplicates. B) The induction of IL‐12p40 of the DEVA_5_K and the HAAHA conjugates was compared by stimulating bone‐marrow‐derived dendritic cells for 24 h, followed by sandwich ELISA. C) and D) Antigen uptake and presentation to T cells was measured by incubating dendritic cells in duplicate with the indicated compounds for 3 h, followed by incubation with the reporter hybridoma T cell lines. The SIINFEKL‐specific hybridoma T cell line B3Z served as readout for the DEVA_5_K peptide, while the OTIIZ hybridoma cell line was used to detect presentation of the HAAHA epitope. B3Z or OTIIZ activation was determined by colorimetric reaction of the lacZ reporter enzyme. Results are representative of three independently performed experiments. The statistical differences between compounds at the same concentration were calculated by two‐way ANOVA followed by multiple comparison and Tukey corrections. * *p*<0.05, ** *p*<0.01, *** *p*<0.001, **** *p*<0.0001.

We assessed the capacity of the conjugates **1**–**8** to induce maturation of dendritic cells (DCs) and stimulate antigen presentation (Figure 2). In these assays reference compounds **35**–**40**, lacking the mannose‐6‐phosphonate clusters, were used as a control (Scheme S2). The activation of DCs can be measured by the detection of the production of interleukin‐12 (IL‐12).

First, the *O*‐M6Po conjugates **1**–**4** were evaluated (Figure 2A). To this end, murine bone marrow/derived DCs were stimulated for 24 hours with the compounds and the amount of secreted IL‐12 was measured in the supernatant. As expected, conjugation with solely a cluster of M6Po (as in conjugates **1** and **3**) does not induce DC maturation. In contrast, the TLR7 ligand SLP conjugates (**36** and **37**) induce IL12 production due to stimulation of the TLR7 receptor.

Interestingly, the conjugates carrying the M6Po‐cluster and the TLR7 ligand (**2** and **4**) induced a stronger activation of the DCs than their counterparts solely bearing a TLR7 ligand (i. e., SLP‐conjugates **36** and **37**). The position of the M6Po‐clusters and TLR7 ligand in these conjugates did not seem to influence the activity of the conjugates. Similar effects were observed for the conjugates **6** and **8** with the *C*‐M6Po‐clusters, indicating that the *C*‐mannosyl‐6‐*C*‐phosphonate is an adequate mimic of its *O*‐mannosyl counterpart and that the enhanced stimulatory effect is independent of the peptide sequence (Figure 2B, see also Figure S1). Overall this indicates that conjugation of the M6Po‐cluster to a peptide antigen adjuvanted with a TLR7 ligand enhances DC maturation by improving uptake of the conjugate and/or trafficking of the conjugates to the endosomally located TLR7 receptor.

Processing of the two peptides was investigated by assessing presentation of the SIINFEKL epitope on MHC‐I the DCs to CD8^+^ T cells and presentation of the helper epitope (HAAHA) to CD4^+^ T cells. For the former assay, the hybridoma (B3Z) CD8^+^ T cell line was used, while the latter employed the OTIIZ hybridoma CD4^+^ T cell line. As can be seen in Figure 2C and D, attachment of the TLR7 ligand to the SLPs led to the enhanced presentation of the antigens (e. g., **35** vs. **36**/**37** and **38** vs. **40**), however the inclusion of the M6Po‐clusters hampered presentation through both the MHC class I and MHC class II pathways (e. g., **36** vs. **4**, **37** vs. **2** and **40** vs. **6**). This indicates that the conjugation of M6Po‐clusters affects intracellular trafficking or processing of the peptides.

## Conclusion

In conclusion, we have described the development of two mannose‐6‐*C*‐phosphonate (M6Po) building blocks which allow for stabilized M6P‐analogues to be incorporated in peptide sequences to target the M6P receptor. To prevent dephosphorylation by endogenous phosphatases, the O‐phosphate in the naturally occurring mannose‐6‐phosphate was replaced by a C‐phosphonate moiety. The first building block carries an *O*‐propargyl group at the anomeric center of the mannose‐6‐*C*‐phosphonate, which allows for the incorporation of the M6Po in peptide sequences through an azide‐alkyne click reaction. The second building block, a *C*‐mannoside, was designed and synthesized for application in solid‐phase peptide synthesis. The acid‐stable anomeric linkage and protecting groups used enabled the streamlined in‐line incorporation of the building block during SPPS. With the building blocks, various peptide conjugates were assembled, containing either an MHC‐I or an MHC‐II epitope, an M6Po‐cluster presenting six mannose‐6‐phosphonates, and a TLR7 ligand. Although immunological evaluation has shown that conjugation of the M6Po‐clusters inhibits antigen presentation, the ability of the TLR7 ligand conjugates to induce DC maturation was significantly improved. While the M6Po clusters effectively trafficked the conjugates to the endosome, where the conjugates interacted with TLR7 receptor, the processing of the conjugates was impeded by the M6Po‐clusters. Future conjugates will be designed featuring cleavable linkers that allow for the release of the clusters during endolysosomal processing, effectively liberating the incorporated peptide antigens for presentation.

## Conflict of interest

The authors declare no conflict of interest.

## Supporting information

As a service to our authors and readers, this journal provides supporting information supplied by the authors. Such materials are peer reviewed and may be re‐organized for online delivery, but are not copy‐edited or typeset. Technical support issues arising from supporting information (other than missing files) should be addressed to the authors.

Supplementary
