# Supplementary material for: Multivalent, Stabilized Mannose‐6‐Phosphates for the Targeted Delivery of Toll‐Like Receptor Ligands and Peptide Antigens
Source: Chembiochem. 2020 Oct 23;22(2):434–40. doi: 10.1002/cbic.202000538 (PMC7894537; doi:10.1002/cbic.202000538)

ChemBioChem

Supporting Information

Multivalent, Stabilized Mannose-6-Phosphates for the Targeted Delivery of Toll-Like Receptor Ligands and Peptide Antigens

Niels R. M. Reintjens, Elena Tondini, Christopher Vis, Toroa McGlinn, Nico J. Meeuwenoord, Tim P. Hogervorst, Herman S. Overkleeft, Dmitri V. Filippov, Gijsbert A. van der Marel, Ferry Ossendorp, and Jeroen D. C. Codée*

Supplementary information

Supplementary Schemes and Figures 2
Material and methods 3
Synthesis 4
Supplemental references 18
LC-MS spectra 19
NMR spectra 38

Supplementary Schemes and Figures

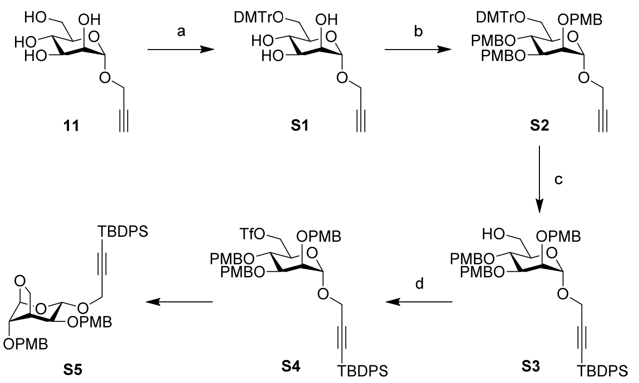

Scheme S1. Synthesis of alcohol **S3** and the cyclized product **S5**. *Reagents and conditions*: a) DMTrCl, pyridine, 67%); b) PMBCl, NaH, DMF, 85%; c) *i*. *n*-BuLi, TBDPSCl, THF, -70°C to rt; *ii*. AcOH, DCM, H_2O, 68% over two steps; d) 2,6-lutidine, Tf_2O, DCM, -40°C.

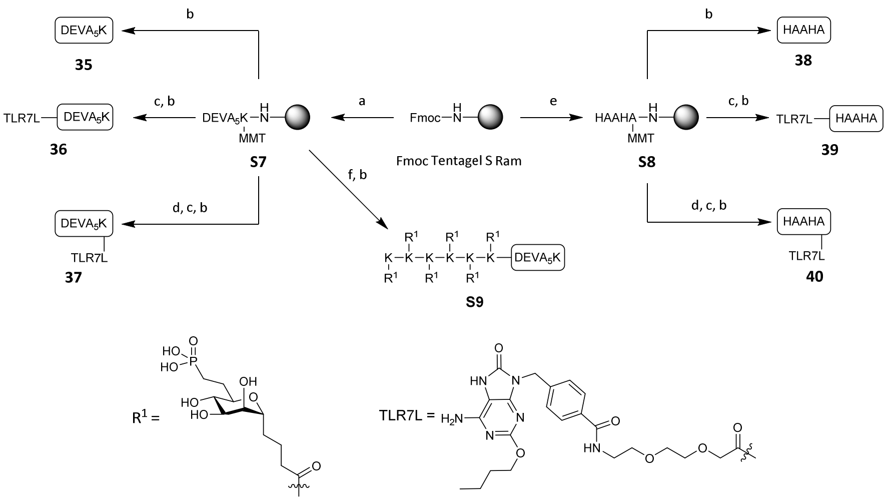

Scheme S2. Synthesis of reference compounds **35**-**40** and **S9**. *Reagents and conditions*: a) *i*. 20% piperidine, DMF; *ii*. Fmoc SPPS cycle for DEVSGLEQLESIINFEKLAAAAAK; *iii*. 20% piperidine, DMF; b) *i*. TFA/TIS/H_2O (95/2.5/2.5 v/v/v), 3h; *ii*. RP-HPLC; c) *i*. {2-[2-(Fmoc-amino)ethoxy]ethoxy}acetic acid, HCTU, DIPEA, DMF; *ii*. 20% piperidine, DMF; *iii*. 4-((2-butoxy-6-((*tert*-butoxycarbonyl)amino)-8-oxo-7,8-dihydro-9H-purin-9-yl)methyl)benzoid acid, HCTU, DIPEA, DMF; d) *i*. Ac_2O, DIPEA, DMF; *ii*. TFA/TIS/DCM (2/2/96 v/v/v); e) *i*. 20% piperidine, DMF; *ii*. Fmoc SPPS cycle for ISQAVHAAHAEINEAGRK; *iii*. 20% piperidine, DMF; f) *i*. **10**, HCTU, DIPEA, DMF; *ii*. 20% piperidine, DMF; *iii*. 5x repeat of *i*. and *ii*.; *iv*. 20% piperidine, DMF; *v*. Ac_2O, DIPEA, DMF; Yield peptides and conjugates: **35**) 12 mg, 16%; **36**) 4.1 mg, 5%; **37**) 9.4 mg, 10%; **38**) 16.8 mg, 30%; **39**) 10.3 mg, 14%; **40**) 7.6 mg, 10%; **S9**) 8.2 mg, 4%.

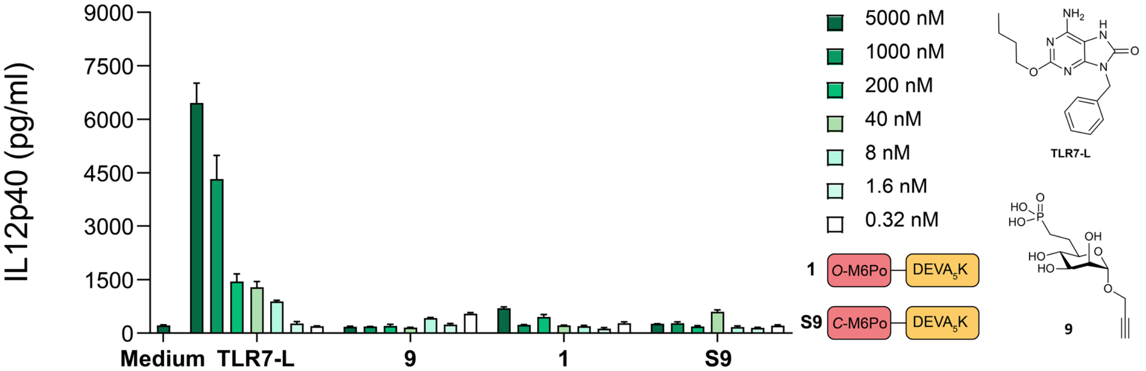

Figure S1. Comparison between *O*-M6Po (**1**) and *C*-M6Po (**S9**) DEVA$_5$K conjugates. Murine bone marrow-derived dendritic cells were stimulated in triplicates for 3 hours with the indicated conjugates of the DEVA$_5$K peptide. After 16 hours, the induction of DC maturation was analyzed by measuring IL-12p40 production. The bars indicate the standard deviation of the mean of the triplicates.

Material and methods

Cell culture

Bone marrow-derived dendritic cells were differentiated from bone marrow stem cells that were harvested from the femurus and tibia of C57BL/6 mice and cultured in IMDM (Lonza) supplemented with FCS (Greiner) , Glutamax (Gibco), penicillin (Gibco), β-mercaptoethanol (Merck) and R1 supernatant. R1 supernatant was obtained by culturing NIH3T3 fibroblasts transfected with GM-CSF. D1 cells, a long-term growth factor-dependent immature myeloid dendritic cell line of splenic origin derived from a female C57BL/6 mouse, was cultured in IMDM supplemented with FCS, Glutamax, penicillin, β-mercaptoethanol and R1 supernatant. B3Z and OTIIZ hybridomas cell lines were cultured in IMDM medium supplemented with FCS, penicillin and streptomycin (Gibco), glutamine (Gibco,), β-mercaptoethanol, and hygromycin B (AG Scientific), to maintain expression of the lacZ reporter gene.

In vitro dendritic cell maturation assay

The test compounds were dissolved in DMSO at a concentration of 1 mM and sonicated in water bath for 15 minutes. Murine bone marrow-derived dendritic cells were seeded in 96-well plates at a density of 50.000 cells/well and incubated with titrated amounts of compounds. After 3 hours of incubation, the cells were washed once and incubated with fresh medium. After 16 hours, supernatant was harvested for ELISA analysis (Biolegend) to measure the amount of produced IL-12p40.

In vitro antigen presentation assay

The test compounds were dissolved in DMSO at a concentration of 1 mM and sonicated in water bath for 15 minutes. 50.000 D1 cells were seeded in 96-well flat bottom plates and incubated with the indicated test compounds. After 2 hours 50.000 B3Z or OTIIZ were added per well and incubated with the D1 cells overnight. The following day, B3Z or OTIIZ activation was detected by measurement of absorbance at 595 nm upon color conversion of chlorophenol red-β-D-galactopyranoside (Calbiochem) the lacZ reporter enzyme.

General experimental

All reagents were of commercial grade and used as received unless stated otherwise. Reaction solvents were of analytical grade and when used under anhydrous conditions stored over flame-dried 3Å molecular sieves. All moisture and oxygen sensitive reactions were performed under an argon atmosphere. Column chromatography was performed on silica gel (Screening Devices BV, 40-63 µm, 60 Å). For TLC analysis, pre-coated silica gel aluminum sheets (Merck, silica gel 60, F254) were used with detection by UV-absorption (254/366 nm) where applicable. Compounds were visualized on TLC by UV absorption (245 nm), or by staining with one of the following TLC stain solutions: $(NH_4)_6Mo_7O_{24}\cdot H_2O$ (25 g/L), $(NH_4)_4Ce(SO_4)_4\cdot 2H_2O$ (10 g/L) and 10% H_2SO_4 in H_2O; bromocresol (0.4 g/L) in EtOH; $KMnO_4$ (7.5 g/L), K_2CO_3 (50 g/L) in H_2O. Staining was followed by charring at ~150°C. ^{1}H, ^{13}C and ^{31}P NMR spectra were recorded on a Bruker AV-400 (400/100/162 MHz) spectrometer and all individual signals were assigned using 2D-NMR spectroscopy. Chemical shifts are given in ppm (δ) relative to TMS (0 ppm) in $CDCl_3$ or via the solvent residual peak. Coupling constants (*J*) are given in Hz. LC-MS analysis were done on an Agilent Technologies 1260 Infinity system with a C18 Gemini 3 µm, C18, 110 Å, 50 x 4.6 mm column. Absorbance was measured at 214 nm and 256 nm and an Agilent Technologies 6120 Quadrupole mass spectrometer was used as detector. Peptides and conjugates were purified with a Gilson GX-281 preparative

HPLC with a Gemini-NX 5u, C18, 110 Å, 250 x 10.0 mm column. Peptide fragments were synthesized with automated solid phase peptide synthesis on an Applied Biosystems 433A Peptide Synthesizer. Optical rotations were measured on an Anton Paar Modular Circular Polarimeter MCP 100/150. High resolution mass spectra were recorded on a Synapt G2-Si or a Q Exactive HF Orbitrap equipped with an electrospray ion source positive mode. Infrared spectra were recorded on a Perkin Elmer Spectrum 2 FT-IR. The results from the *in vitro* assays were analysed with GraphPad Prism Version 7.00 for Windows, GraphPad Software.

Automated solid phase synthesis general experimental information

The automated solid-phase peptide synthesis was performed on a 250 µmol scale on a Protein Technologies Tribute-UV IR Peptide Synthesizer applying Fmoc based protocol starting from Tentagel S RAM resin (loading 0.22 mmol/g). The synthesis was continued with Fmoc-amino acids specific for each peptide. The consecutive steps performed in each cycle for HCTU chemistry on 250 µmol scale: 1) Deprotection of the Fmoc-group with 20% piperidine in DMF for 10 min; 2) DMF wash; 3) Coupling of the appropriate amino acid using a four-fold excess. Generally, the Fmoc amino acid (1.0 mmol) was dissolved in 0.2 M HCTU in DMF (5 mL), the resulting solution was transferred to the reaction vessel followed by 2 mL of 1 M DIPEA in DMF to initiate the coupling. The reaction vessel was then shaken for 30 min at 50°C; 4) DMF wash; 5) capping with 10% Ac_2O in 0.1 M DIPEA in DMF; 6) DMF wash; 7) DCM wash. Aliquots of resin of the obtained sequences were checked on an analytical Agilent Technologies 1260 Infinity system with a Gemini 3 µm, C18, 110 Å, 50 x 4.6 mm column. The Fmoc amino acids applied in the synthesis were: Fmoc-Ala-OH, Fmoc-Arg(Pbf)-OH, Fmoc-Asn(Trt)-OH, Fmoc-Asp(O*t*Bu)-OH, Fmoc-Gln(Trt)-OH, Fmoc-Glu(O*t*Bu)-OH, Fmoc-Gly-OH, Fmoc-His(Trt)-OH, Fmoc-Ile-OH, Fmoc-Leu-OH, Fmoc-Lys(N_3)-OH, Fmoc-Lys(Boc)-OH, Fmoc-Lys(MMT)-OH, Fmoc-Phe-OH, Fmoc-Ser(*t*Bu)-OH Fmoc-Val-OH.

General procedure for cleavage from the resin, deprotection and purification

30 µmol resin was washed with DMF, DCM and dried after the last synthesis step followed by a treatment for 180 minutes with 0.6 mL cleavage cocktail of 95% TFA, 2.5% TIS and 2.5% H_2O. The suspension was filtered, the resin was washed with 0.6 mL of the cleavage cocktail, and the combined TFA solutions were added dropwise to cold Et_2O and stored at -20°C overnight. The obtained suspension of the product in Et_2O was centrifuged, Et_2O was removed and the precipitant was dissolved in $CH_3CN/H_2O/$*t*BuOH (1/1/1 v/v/v) or DMSO/$CH_3CN/H_2O/$*t*BuOH (3/1/1/1 v/v/v/v). Purification was performed on a Gilson GX-281 preparative RP-HPLC with a Gemini-NX 5u, C18, 110 Å, 250 x 10.0 mm column.

Synthesis

Propargyl α-D-mannopyranoside (11)

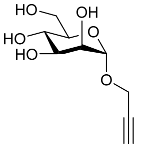

To a solution of D-mannose (28.1 g, 156 mmol, 1.0 eq.) in pyridine (0.22 L) was added acetic anhydride (0.10 L, 1.1 mol, 6.8 eq.) and DMAP (0.37 g, 3.0 mmol, 0.02 eq.) at 0°C. After 30 minutes the solution was allowed to warm-up to room temperature and stirred for an additional 5.5 hours. The reaction mixture was cooled to 0°C and quenched by slowly adding MeOH. The mixture was diluted with EtOAc, washed with 1 M HCl (3x), dried over $MgSO_4$, filtered and concentrated *in vacuo*. Co-evaporation with toluene (2x) under an argon atmosphere yielded acetyl 2,3,4,6-tetra-*O*-acetyl-α/β-D-mannoside as an oil, which was dissolved in DCM (0.45 L). Propargyl alcohol (14.0 mL, 240 mmol, 1.5 eq.) and $BF_3{\cdot}OEt_2$ (30.0 mL, 239 mmol, 1.5 eq.) were added and the solution was stirred overnight at 50°C. TLC analysis showed complete conversion and the reaction mixture was cooled to 0°C, followed by quenching with Et_3N to pH 8. The dark solution was washed with sat. aq. $NaHCO_3$ (1x), dried over Na_2SO_4, filtered and concentrated *in vacuo*. Purification by flash column chromatography (50→100% Et_2O in pentane) yielded propargyl 2,3,4,6-tetra-*O*-acetyl-α-D-mannopyranoside (55.3 g, 143 mmol), which was dissolved in MeOH (0.30 L). Sodium methoxide (5.4 M in MeOH, 11 mL, 60 mmol, 0.40 eq.) was added and the solution was stirred for 30 minutes, after which it was acidified by the addition of amberlite H^+ resin. The mixture was filtered and concentrated *in vacuo*. Crystallization by EtOH/pentane afforded the title compound (22.9 g, 105 mmol, 67% over three steps) as a white solid. R_f: 0.39 (1/4 MeOH/DCM); $[\alpha]_D^{25}$ +145° (*c* = 0.53, MeOH); ^1H NMR (MeOD, 400 MHz, HH-COSY, HSQC): δ 4.96 (d, 1H, *J* = 1.6 Hz, H-1), 4.27 (d, 2H, *J* = 2.4 Hz, CH_2 propargyl), 3.84 (dd, 1H, *J* = 11.8, 2.3 Hz, C*H*H-6), 3.80 (dd, 1H, *J* = 3.2, 1.7 Hz, H-2), 3.74 – 3.65 (m, 2H, H-3, C*H*H-6), 3.62 (t, 1H, *J* = 9.4 Hz, H-4), 3.51 (ddd, 1H, *J* = 8.7, 5.8, 2.2 Hz, H-5), 2.86 (t, 1H, *J* = 2.4 Hz, CH propargyl); ^{13}C-APT NMR (MeOD, 101 MHz, HSQC): δ 99.7 (C-1), 80.0 (C_q propargyl), 76.0 (CH propargyl), 75.0 (C-5), 72.4 (C-3), 72.0 (C-2), 68.4 (C-4), 62.8 (CH_2-6), 54.8 (CH_2 propargyl); FT-IR (neat, cm^{-1}): 3370, 2931, 2584, 1982, 1639, 1365, 1263, 1132, 1058, 1007, 965, 912, 880, 812, 685, 515; HRMS: $[M+Na]^+$ calcd. for $C_9H_{14}O_6Na$: 241.0688, found 241.0684.

Propargyl-6-*O*-(4,4'-dimethoxytrityl)-*α*-D-mannopyranoside (S1)

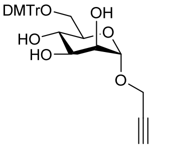

DMTrCl (9.63 g, 28.4 mmol, 1.4 eq.) was added to a solution of compound **7** (4.40 g, 20.2 mmol, 1.0 eq) in pyridine (79 ml). The reaction was stirred overnight and quenched with sat. aq. $NaHCO_3$ and diluted with DCM. The organic layer was washed with H_2O (3x) and brine (1x) and the combined aqueous layers were extracted with DCM (3x). The combined organic layers were dried over Na_2SO_4, filtered and concentrated *in vacuo*. Purification by column chromatography (0→100% EtOAc in pentane + 0.5% Et_3N) yielded **S1** as an orange foam (7.01 g, 13.5 mmol, 67%). ^{1}H NMR (MeOD, 400 MHz, HH-COSY, HSQC): δ 7.48 (d, 2H, *J* = 7.6 Hz, Ar), 7.40-7.30 (m, 4H, Ar), 7.24 (t, 2H, *J* = 7.6 Hz, Ar), 7.16 (t, 1H, *J* = 7.3 Hz, Ar), 6.82 (d, 4H, *J* = 8.9 Hz, Ar), 5.03 (s, 1H, H-1), 4.39 (d, 2H, *J* = 2.3 Hz, CH_2 propargyl), 3.86-3.81 (m, 1H, H-2), 3.73 (s, 7H, H-5, 2x CH_3 DMTr), 3.66 (dd, 1H, *J* = 9.3, 3.4 Hz, H-3), 3.54 (t, 1H, *J* = 9.6 Hz, H-4), 3.47-3.40 (m, 1H, C*H*H-6), 3.26 (dd, 1H, *J* = 9.7, 7.4 Hz, C*H*H-6), 2.90 (t, 1H, *J* = 2.3 Hz, CH propargyl); ^{13}C NMR (MeOD, 101 MHz, HSQC): δ 159.9, 146.7, 137.5, 131.3, 129.4, 128.6, 127.6, 114.0 (Ar), 99.5 (C-1), 87.1 (C_q DMTr), 79.9, 76.2 (C≡C), 74.1 (C-5), 72.8 (C-3), 71.8 (C-2), 67.0 (C-4), 64.9 (CH_2-6), 55.7 (CH_3 DMTr), 54.5 (CH_2 propargyl); FT-IR (neat, cm^{-1}): 3437, 3281, 3001, 2928, 2835, 1969, 1726, 1607, 1582, 1506, 1462, 1443, 1414, 1379, 1300, 1246, 1175, 1132, 1069, 1030, 1007, 984, 968, 914, 903, 881, 827, 810, 791, 772, 754, 727, 700, 667, 637, 623; HRMS: $[M+Na]^+$ calcd. for $C_{30}H_{32}O_8Na$: 543.19894, found 543.19856.

Propargyl-6-*O*-(4,4'-dimethoxytrityl)-2,3,4-tri-*O*-*p*-methoxybenzyl-*α*-D-mannopyranoside (S2)

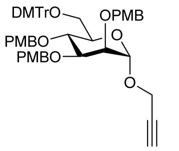

After co-evaporation with toluene (3x), compound **S1** (6.45 g, 12.4 mmol, 1.0 eq.) was dissolved in DMF (120 ml) under an argon atmosphere and cooled to 0°C. Sodium hydride (60% dispersion in mineral oil, 2.50 g, 62.5 mmol, 5.0 eq.) and *p*-methoxybenzyl chloride (7.5 ml, 54 mmol, 4.4 eq.) were added and the reaction was allowed to warm-up to room temperature and stirred overnight. After quenching with MeOH at 0°C, the reaction mixture was diluted with Et_2O and washed with H_2O (3x). The combined aqueous layers were extracted with Et_2O (3x). The combined organic layers were dried over Na_2SO_4, filtered and concentrated *in vacuo*. Purification by column chromatography (0→30% EtOAc in pentane + 0.5% Et_3N) yielded the title compound as a yellow foam (9.34 g, 10.6 mmol, 85%). ^{1}H NMR (CD_2Cl_2, 400 MHz, HH-COSY, HSQC): δ 7.63 (d, 2H, *J* = 7.6 Hz, Ar), 7.47 (t, 6H, *J* = 7.3 Hz, Ar), 7.39 (dd, 4H, *J* = 12.0, 8.0 Hz, Ar), 7.34-7.28 (m, 1H, Ar), 7.00-6.86 (m, 10H, Ar), 6.82 (d, 2H, *J* = 8.5 Hz, Ar), 5.25 (s, 1H, H-1), 4.86-4.63 (m, 5H, 2x CH_2 PMB, 1x C*H*H PMB), 4.43 (s, 2H, CH_2 propargyl), 4.31 (d, 1H, *J* = 10.3 Hz, C*H*H PMB), 4.03-3.90 (m, 3H, H-2, H-3, H-4), 3.85 (d, 16H, *J* = 10.1 Hz, H-5, 3x CH_3 PMB, 2x CH_3 DMTr), 3.57 (d, 1H, *J* = 9.5 Hz C*H*H-6), 3.29 (q, 1H, *J* = 9.7, 5.5 Hz, *C*HH-6), 2.63 (s, 1H, CH propargyl); ^{13}C NMR (CD_2Cl_2, 101 MHz, HSQC): δ 159.7, 159.6, 159.5, 158.9, 145.7, 136.7, 136.4, 131.2, 131.0, 130.6, 130.5, 130.0, 129.9, 129.9, 129.9, 129.8, 129.6, 128.6, 128.2, 127.0, 114.1, 114.0, 113.9, 113.7, 113.4 (Ar), 96.8 (C-1), 86.0 (C_q DMTr), 80.1 (C-5), 79.5 (C≡C), 75.4 (C-3), 75.0 (C≡C), 75.0 (C-2), 74.8, 73.0 (CH_2 PMB), 72.8 (C-4), 72.1 (CH_2 PMB), 63.2 (CH_2-6), 55.5 (CH_3 PMB/DMTr), 54.2 (CH_2 propargyl); FT-IR (neat, cm^{-1}): 3283, 2999, 2930, 2911, 2864, 2835, 1676, 1611, 1585, 1510, 1462, 1443, 1422, 1385, 1362, 1300, 1244, 1173, 1136, 1105, 1086, 1053, 1030, 934, 914, 903, 822, 791, 754, 727, 700, 673, 658, 637; HRMS: $[M+Na]^+$ calcd. for $C_{54}H_{56}O_{11}Na$: 903.37148, found 903.37152.

***tert*-Butyldiphenylsilylpropargyl 2,3,4-tri-*O*-*p*-methoxybenzyl-*α*-D-mannopyranoside (S3)**

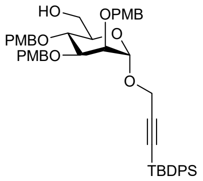

Compound **S2** (2.21 g, 2.85 mmol, 1.0 eq) was co-evaporated with toluene (3x) and dissolved in THF (30 ml) under an argon atmosphere. The reaction was cooled to -70°C, followed by the addition of *n*-butyllithium (1.6 M in hexanes, 1.9 ml, 3.0 mmol, 1.1 eq). After stirring for 1 hour at -70°C, TBDPSCl (0.91 ml, 3.50 mmol, 1.23 eq) was added and the reaction was slowly brought to room temperature. After stirring overnight the reaction was quenched with MeOH at 0°C, diluted with DCM and washed with brine (1x). The aqueous layer was extracted with DCM (3x) and the combined organic layers were dried over Na_2SO_4, filtered and concentrated *in vacuo*. The obtained intermediate was dissolved in a DCM/H_2O/AcOH mixture (4/1/15 v/v/v, 20 mL). After stirring for 4 hours, the reaction was quenched with sat. aq. $NaHCO_3$ and diluted with Et_2O. The reaction mixture was washed with sat. aq. $NaHCO_3$ (3x) and the combined aqueous layers were extracted with Et_2O (3x). The combined organic layers were dried over $MgSO_4$, filtered and concentrated *in vacuo*. Purification by column chromatography (0→30% EtOAc in pentane) yielded **S3** as a colorless foam (1.59 g, 1.95 mmol, 68%). $[\alpha]_D^{20}$= +16.92° (c = 1.06, $CHCl_3$); ^{1}H NMR ($CDCl_3$, 400 MHz, HH-COSY, HSQC): δ 7.83-7.71 (m, 4H, Ar), 7.46-7.31 (m, 6H, Ar), 7.29-7.16 (m, 6H, Ar), 6.85 (d, 4H, *J* = 7.5 Hz, Ar), 6.76 (d, 2H, *J* = 8.6 Hz, Ar), 5.21 (d, 1H, *J* = 1.3 Hz, H-1), 4.86 (d, 1H, *J* = 10.5 Hz, C*H*H PMB), 4.69-4.47 (m, 5H, 2x CH_2 PMB, 1x C*H*H PMB), 4.35 (s, 2H, CH_2 propargyl), 3.94 (d, 1H, *J* = 9.6 Hz, H-3), 3.89 (dd, 1H, *J* = 9.4, 2.8 Hz, H-5), 3.84-3.80 (m, 2H, H-2, C*H*H-6), 3.79 (s, 6H, 2x CH_3 PMB), 3.75 (d, 1H, *J* = 4.6 Hz, C*H*H-6), 3.73 (s, 3H, CH_3 PMB), 3.66-3.59 (m, 1H, H-4), 1.99 (s, 1H, OH), 1.10 (s, 9H, *t*Bu); ^{13}C NMR ($CDCl_3$, 101 MHz, HSQC): δ 159.4, 159.3, 159.2, 135.7, 135.6, 132.9, 130.7, 130.1, 129.8, 129.6,

129.3, 127.9, 113.9, 104.6 (Ar), 96.1 (C-1), 87.4 (C≡C), 79.7 (C-5), 75.0 (CH_2 PMB), 74.5 (C-3), 74.0 (C-2), 72.7 (C-4), 72.3, 71.8 (CH_2 PMB), 62.4 (CH_2-6), 55.4, 55.4, 55.3 (CH_3 PMB), 54.9 (CH_2 propargyl), 29.8 (C≡C), 27.2 (CH_3 *t*Bu), 18.6 (C_q *t*Bu); FT-IR (neat, cm^{-1}): 2951, 2928, 2857, 1757, 1738, 1612, 1512, 1462, 1427, 1364, 1302, 1244, 1217, 1173, 1136, 1109, 1076, 1034, 1013, 978, 939, 912, 893, 849, 820, 743, 700, 648, 602; HRMS: $[M+Na]^+$ calcd. for $C_{49}H_{56}O_9SiNa$: 839.35858, found 839.35843.

Cyclized product (S5)

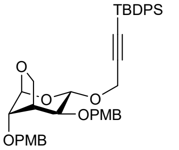

S5 was obtained instead of **S4** after treatment of **S3** with 2,6-lutidine and Tf_2O at -40°C. $[\alpha]_D^{20}$= +11.57° (c = 1.66, $CHCl_3$); ^1H NMR ($CDCl_3$, 400 MHz, HH-COSY, HSQC): δ 7.86-7.78 (m, 4H, Ar), 7.44-7.34 (m, 6H, Ar), 7.32-7.20 (m, 4H, Ar), 6.90-6.80 (m, 4H, Ar), 5.30-5.26 (m, 1H, H-1), 4.76 (d, 1H, *J* = 11.7 Hz, C*H*H PMB), 4.67-4.52 (m, 4H, 2x C*H*H PMB, CH_2 propargyl), 4.43 (d, *J* = 11.7 Hz, 1H, C*H*H PMB), 4.37 (t, 1H, *J* = 2.8 Hz, H-4), 4.18-4.12 (m, 1H . H-5), 4.04 (d, 1H, *J* = 10.6 Hz, C*H*H-6), 3.92-3.86 (m, 2H, H-3, C*H*H-6), 3.81 (s, 3H, CH_3 PMB), 3.76 (s, 3H, CH_3 PMB), 3.73 (dd, 1H, *J* = 6.6, 1.5 Hz, H-2), 1.12 (s, 9H, *t*Bu); ^{13}C NMR ($CDCl_3$, 101 MHz, HSQC): δ 159.6, 159.2, 135.7, 133.1, 133.0, 130.6, 129.8, 129.7, 129.6, 129.4, 127.9, 127.8, 114.0, 113.7, 105.2 (Ar), 100.4 (C-1), 87.2 (C≡C), 77.0 (C-3), 75.7 (C-5), 75.1 (C-2), 73.6 (C-4), 72.4, 71.8 (CH_2 PMB), 69.4 (CH_2-6), 56.9 (CH_2 propargyl), 55.3 (CH_3 PMB), 27.1 (CH_3 *t*Bu), 26.6 (C≡C), 18.6 (C *t*Bu); FT-IR (neat, cm^{-1}): 2953, 2990, 2889, 2857, 1611, 1512, 1462, 1427, 1362, 1302, 1246, 1173, 1144, 1103, 1080, 1032, 1009, 997, 966, 941, 905, 881, 847, 819, 735, 698, 667, 608; HRMS: $[M+Na]^+$ calcd. for $C_{41}H_{46}O_7SiNa$: 701.29050, found 701.29002.

Propargyl 6-*O*-trityl-α-D-mannopyranoside (12)

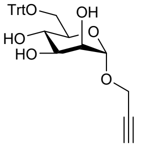

Trityl chloride (57.2 g, 205 mmol, 1.5 eq.) and Et_3N (46 mL, 0.33 mol, 2.5 eq.) were added to a solution of compound **11** (29.0 g, 133 mmol, 1.0 eq.) in DMF (0.44 L). The mixture was heated to 60°C for 4 hours, followed by addition of trityl chloride (38.1 g, 137 mmol, 1.0 eq.) and Et_3N (28 mL, 0.20 mol, 1.5 eq.). After stirring for one hour, TLC analysis showed complete conversion of the starting material and the reaction mixture was cooled to room temperature. The mixture was diluted with EtOAc, washed with H_2O (3x), dried over Na_2SO_4, filtered and concentrated *in vacuo*. After purification by column chromatography (30→100% EtOAc in pentane), the title compound (50.4 g, 109 mmol, 82%) was obtained as a white foam. R_f: 0.65 (1/4 pentane/EtOAc); $[\alpha]_D^{25}$ +45.3° (*c* = 0.91, $CHCl_3$); ^1H NMR (CD_3CN, 400 MHz, HH-COSY, HSQC): δ 7.53 – 7.45 (m, 6H, Ar), 7.38 – 7.30 (m, 6H, Ar), 7.30 – 7.22 (m, 3H, Ar), 4.96 (d, 1H, *J* = 1.2 Hz, H-1), 4.44 – 4.30 (m, 2H, CH_2 propargyl), 3.81 – 3.75 (m, 1H, H-2), 3.67 (ddd, 1H, *J* = 9.1, 7.1, 1.7 Hz, H-5), 3.55 (ddd, 1H, *J* = 9.5, 6.3, 3.5 Hz, H-3), 3.52 – 3.42 (m, 1H, H-4), 3.36 – 3.28 (m, 2H, C*H*H-6, OH), 3.26 (d, 1H, *J* = 6.4 Hz, OH), 3.18 (dd, 1H, *J* = 9.9, 7.0 Hz, C*H*H-6), 3.11 (d, 1H, *J* = 5.0 Hz, OH), 2.78 (t, 1H, *J* = 2.4 Hz, CH propargyl); ^{13}C-APT NMR (CD_3CN, 101 MHz, HSQC): δ 145.2 (C_q Trt), 129.6, 128.8, 128.0 (Ar), 99.1 (C-1), 87.2 (C_q Trt), 80.0 (C_q propargyl), 76.1 (CH propargyl), 73.3 (C-5), 72.5 (C-3), 71.2 (C-2), 68.6 (C-4), 64.7 (CH_2-6), 54.4 (CH_2 propargyl); FT-IR (neat, cm^{-1}): 3412, 3290, 3059, 3033, 2928, 2119, 1597, 1490, 1449, 1377, 1320, 1221, 1184, 1134, 1074, 1049, 1005, 986, 900, 844, 810, 765, 748, 702, 650, 633, 582, 531; HRMS: $[M+Na]^+$ calcd. for $C_{28}H_{28}O_6Na$: 483.1784, found 483.1780.

Propargyl 2,3-di-*O*-isopropylidene-6-*O*-trityl-α-D-mannopyranoside (13)

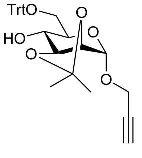

To a solution of compound **12** (50.4 g, 109 mmol, 1.0 eq.) in 2,2-dimethoxypropane (0.55 L) was added *p*-toluenesulfonic acid (3.22 g, 16.9 mmol, 0.15 eq.) at 0°C. After stirring for 1.5 hours, TLC analysis showed complete conversion of the starting material. The mixture was quenched by the addition of Et_3N (8 mL), diluted with brine and extracted with DCM (2x). The combined organic layers were dried over Na_2SO_4, filtered and concentrated *in vacuo*. Purification by column chromatography (10→50% Et_2O in pentane) and crystallization in DCM/pentane yielded compound **13** (47.7 g, 95.4 mmol, 87%) as a white solid. R_f: 0.20 (7/3 pentane/Et_2O); $[\alpha]_D^{25}$ +23.0° (*c* = 0.67, $CHCl_3$); ^1H NMR (CD_3CN, 400 MHz, HH-COSY, HSQC): δ 7.52 – 7.45 (m, 6H, Ar), 7.38 – 7.30 (m, 6H, Ar), 7.30 – 7.24 (m, 3H, Ar), 5.21 (s, 1H, H-1), 4.50 – 4.35 (m, 2H, CH_2 propargyl), 4.14 (d, 1H, *J* = 5.6 Hz, H-2), 3.98 – 3.92 (m, 1H, H-3), 3.69 – 3.61 (m, 1H, H-5), 3.49 – 3.42 (m, 1H, H-4), 3.34 (dd, 1H, *J* = 10.1, 1.7 Hz, C*H*H-6), 3.24 – 3.14 (m, 2H, C*H*H-6, OH), 2.79 (t, 1H, *J* = 2.4 Hz, CH propargyl), 1.45 (s, 3H, CH_3 isopropylidene), 1.32 (s, 3H, CH_3 isopropylidene); ^{13}C-APT NMR (CD_3CN, 101 MHz, HSQC): δ 145.1 (C_q Trt), 129.5, 128.8, 128.1 (Ar), 110.0 (C_q isopropylidene), 96.2 (C-1), 87.3 (C_q Trt), 79.7 (C_q propargyl), 79.6 (C-3), 76.4 (C-2, CH propargyl), 70.9 (C-5), 69.9 (C-4), 64.3 (CH_2-6), 54.5 (CH_2 propargyl), 28.2, 26.5 (CH_3 isopropylidene); FT-IR (neat, cm^{-1}): 3279, 2935, 1490, 1448, 1374, 1225, 1168, 1136, 1103, 1075, 1047, 1029, 992, 918, 898, 851, 822, 786, 767, 743, 705, 696, 650, 634, 583, 543, 532, 471; HRMS: $[M+Na]^+$ calcd. for $C_{31}H_{32}O_6Na$: 523.2097, found 523.2095.

Propargyl 2,3-di-*O*-isopropylidene-4-*O*-*p*-methoxybenzyl-6-*O*-trityl-α-D-mannopyranoside (14)

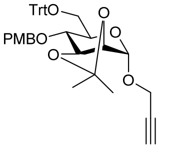

After co-evaporation with toluene (1x) under an argon atmosphere, compound **13** (47.7 g, 95.4 mmol, 1.0 eq.) was dissolved in DMF (0.48 L) and cooled to 0°C. Sodium hydride (60% dispersion in mineral oil, 4.59 g, 115 mmol, 1.2 eq.) and *p*-methoxybenzyl chloride (15.6 mL, 115 mmol, 1.2 eq.) were added to the mixture. After 3 hours stirring at 0°C, the suspension was allowed to warm-up to room temperature and stirred for an additional 2 hours. The reaction mixture was quenched by the addition of MeOH at 0°C, diluted with H_2O and extracted with EtOAc (2x). The combined organic layers were dried over Na_2SO_4, filtered and concentrated *in vacuo*. After purification by column chromatography (5→40% Et_2O in pentane) and crystallization in DCM/pentane the title compound (56.4 g, 90.9 mmol, 95%) was obtained as a white solid. R_f: 0.50 (7/3 pentane/Et_2O); $[\alpha]_D^{25}$ +29.8° (*c* = 0.94, $CHCl_3$); ^1H NMR (CD_3CN, 400 MHz, HH-COSY, HSQC): δ 7.49 – 7.43 (m, 6H, Ar), 7.36 – 7.25 (m, 9H, Ar), 6.94 – 6.89 (m, 2H, Ar), 6.79 – 6.74 (m, 2H, Ar), 5.24 (s, 1H, H-1), 4.59 (d, 1H, *J* = 11.1 Hz, C*H*H PMB), 4.47 – 4.34 (m, 2H, CH_2 propargyl), 4.29 (d, 1H, *J* = 11.1 Hz, C*H*H PMB), 4.21 – 4.15 (m, 2H, H-2, H-3), 3.76 (s, 3H, CH_3 PMB), 3.69 (ddd, 1H, *J* = 10.2, 6.2, 1.6 Hz, H-5), 3.46 (dd, 1H, *J* = 10.3, 6.4 Hz, H-4), 3.38 (dd, 1H, *J* = 10.1, 1.7 Hz, C*H*H-6), 3.09 (dd, 1H, *J* = 10.1, 6.3 Hz, C*H*H-6), 2.78 (t, 1H, *J* = 2.4 Hz, CH propargyl), 1.49 (s, 3H, CH_3 isopropylidene), 1.35 (s, 3H, CH_3 isopropylidene); ^{13}C-APT NMR (CD_3CN, 101 MHz, HSQC): δ 160.1 (C_q PMB), 145.0 (C_q Trt), 131.2 (C_q PMB), 130.5, 129.6, 128.8, 128.1, 114.4 (Ar), 110.1 (C_q isopropylidene), 96.2 (C-1), 87.2 (C_q Trt), 79.7 (C_q propargyl), 79.5 (C-3), 76.4 (C-2), 76.4 (CH propargyl), 76.3 (C-4), 73.1 (CH_2 PMB), 69.7 (C-5), 64.1 (CH_2-6), 55.8 (CH_3 PMB), 54.6 (CH_2 propargyl), 28.2, 26.5 (CH_3 isopropylidene); FT-IR (neat, cm^{-1}): 3286, 1612, 1586, 1513, 1490, 1449, 1372, 1302, 1245, 1220, 1171, 1145, 1076, 1059, 1029, 998, 915, 899, 863, 821, 777, 765, 737, 699, 644, 632, 587, 550, 518, 468; HRMS: $[M+Na]^+$ calcd. for $C_{39}H_{40}O_7Na$: 643.2672, found 643.2677.

Trimethylsilylpropargyl 2,3-di-*O*-isopropylidene-4-*O*-*p*-methoxybenzyl-6-*O*-trityl-α-D-mannopyranoside (15)

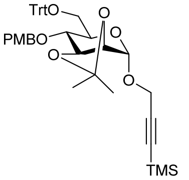

Compound **14** (38.1 g, 61.3 mmol, 1.0 eq.) was co-evaporated twice with toluene under an argon atmosphere and dissolved in THF (0.61 L). The solution was cooled to -78°C, followed by the addition of *n*-butyllithium (1.6 M in hexane, 46 mL, 74 mmol, 1.2 eq.). After 15 minutes, TMSCl (12 mL, 95 mmol, 1.5 eq.) was added dropwise to the pink mixture. The resulting yellow mixture was allowed to warm-up to -50°C over two hours and the reaction was quenched by the addition of sat. aq. NH_4Cl. The mixture was diluted with EtOAc and washed with sat. aq. NH_4Cl (1x) and sat. aq. $NaHCO_3$ (1x). The organic layer was dried over Na_2SO_4, filtered and concentrated *in vacuo*. Purification by column chromatography (2→15% Et_2O in pentane) gave compound **15** in quantitative yield (44.1 g). R_f: 0.38 (9/1 pentane/Et_2O); $[\alpha]_D^{25}$ +33.8° (*c* = 0.45, $CHCl_3$); ^1H NMR (CD_3CN, 400 MHz, HH-COSY, HSQC): δ 7.49 – 7.43 (m, 6H, Ar), 7.36 – 7.24 (m, 9H, Ar), 6.94 – 6.89 (m, 2H, Ar), 6.79 – 6.73 (m, 2H, Ar), 5.24 (s, 1H, H-1), 4.59 (d, 1H, *J* = 11.1 Hz, C*H*H PMB), 4.47 – 4.34 (m, 2H, CH_2 propargyl), 4.30 (d, 1H, *J* = 11.1 Hz, C*H*H PMB), 4.21 – 4.15 (m, 2H, H-2, H-3), 3.75 (s, 3H, CH_3 PMB), 3.71 – 3.65 (m, 1H, H-5), 3.48 (dd, 1H, *J* = 10.4, 6.2 Hz, H-4), 3.39 (dd, 1H, *J* = 10.0, 1.7 Hz, C*H*H-6), 3.10 (dd, 1H, *J* = 10.0, 6.2 Hz, C*H*H-6), 1.50 (s, 3H, CH_3 isopropylidene), 1.35 (s, 3H, CH_3 isopropylidene), 0.15 (s, 9H, 3x CH_3 TMS); ^{13}C-APT NMR (CD_3CN, 101 MHz, HSQC): δ 160.1 (C_q PMB), 145.0 (C_q Trt), 131.2 (C_q PMB), 130.4, 129.6, 128.8, 128.1, 114.4 (Ar), 110.1 (C_q isopropylidene), 101.7 (C≡C), 96.3 (C-1), 92.4 (C≡C), 87.3 (C_q Trt), 79.5 (C-3), 76.5 (C-2), 76.3 (C-4), 73.1 (CH_2 PMB), 69.7 (C-5), 64.1 (CH_2-6), 55.8 (CH_3 PMB), 55.3 (CH_2 propargyl), 28.3, 26.6 (CH_3 isopropylidene), -0.1 (CH_3 TMS); FT-IR (neat, cm^{-1}): 3059, 3033, 2988, 2934, 2179, 1613, 1587, 1514, 1491, 1449, 1381, 1372, 1302, 1248, 1221, 1171, 1146, 1082, 1031, 999, 966, 946, 899, 846, 763, 747, 708, 633, 588, 551, 522, 475; HRMS: $[M+Na]^+$ calcd. for $C_{42}H_{48}O_7SiNa$: 715.3067, found 715.3068.

Trimethylsilylpropargyl 2,3-di-*O*-isopropylidene-4-*O*-*p*-methoxybenzyl-α-D-mannopyranoside (16)

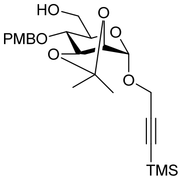

A mixture of compound **15** (42.8 g, 61.3 mmol, 1.0 eq.), *p*-toluenesulfonic acid (4.76 g, 24.5 mmol, 0.4 eq.) in DCM/MeOH (2/1 v/v, 0.42 L) was stirred at room temperature for 1.5 hours, after which TLC analysis showed complete conversion of the starting material. The reaction mixture was quenched by the addition of sat. aq. $NaHCO_3$ (0.50 L) and extracted with EtOAc (1x). The organic layer was dried over Na_2SO_4, filtered and concentrated *in vacuo*. The obtained residue was dissolved in a mixture of 2,2-dimethoxypropane/DCM (4/1, 0.35 L). *p*-Toluenesulfonic acid (1.17 g, 6.02 mmol, 0.1 eq.) was added and the mixture was stirred at room temperature for 25 minutes. The reaction mixture was quenched with sat. aq. $NaHCO_3$ (0.50 L) and extracted with EtOAc (1x). The organic layer was dried over Na_2SO_4, filtered and concentrated *in vacuo*. The obtained intermediates were dissolved in EtOAc (0.28 L) and cooled to 0°C. 1 M HCl (30 mL) was added and the mixture was allowed to warm-up to room temperature. After stirring for 1 hour, TLC analysis showed complete conversion and the reaction was quenched with sat. aq. $NaHCO_3$ (0.50 L) at 0°C, followed by extraction with EtOAc (1x). The organic layer was

dried over Na_2SO_4, filtered and concentrated *in vacuo*. Purification by column chromatography (20→60% Et_2O in pentane) yielded the title compound (26.0 g, 57.8 mmol, 94%) as an oil. R_f: 0.47 (4/1 pentane/EtOAc); $[\alpha]_D^{25}$ +79.6° (*c* = 0.57, $CHCl_3$); ^1H NMR ($CDCl_3$, 400 MHz, HH-COSY, HSQC): δ 7.31 – 7.23 (m, 2H, Ar), 6.90 – 6.81 (m, 2H, Ar), 5.23 (s, 1H, H-1), 4.82 (d, 1H, *J* = 11.1 Hz, C*H*H PMB), 4.56 (d, 1H, *J* = 11.1 Hz, C*H*H PMB), 4.36 – 4.29 (m, 1H, H-3), 4.23 (d, 2H, *J* = 2.3 Hz, CH_2 propargyl), 4.18 (d, 1H, *J* = 6.2 Hz, H-2), 3.86 – 3.81 (m, 1H, C*H*H-6), 3.80 (s, 3H, CH_3 PMB), 3.76 – 3.68 (m, 1H, C*H*H-6), 3.64 – 3.57 (m, 1H, H-5), 3.56 – 3.49 (m, 1H, H-4), 1.94 (br, 1H, OH), 1.52 (s, 3H, CH_3 isopropylidene), 1.38 (s, 3H, CH_3 isopropylidene), 0.17 (s, 9H, 3x CH_3 TMS); ^{13}C-APT NMR ($CDCl_3$, 101 MHz, HSQC): δ 159.4, 130.3 (C_q PMB), 129.8, 113.9 (Ar), 109.6 (C_q isopropylidene), 100.1 (C≡C), 95.7 (C-1), 92.3 (C≡C), 78.8 (C-3), 75.8 (C-2), 75.7 (C-4), 72.7 (CH_2 PMB), 69.0 (C-5), 62.6 (CH_2-6), 55.4 (CH_3 PMB), 55.1 (CH_2 propargyl), 28.1, 26.5 (CH_3 isopropylidene), -0.1 (CH_3 TMS); FT-IR (neat, cm^{-1}): 3493, 2936, 2178, 1613, 1587, 1514, 1457, 1372, 1302, 1246, 1220, 1171, 1142, 1075, 1033, 994, 965, 948, 914, 842, 788, 760, 737, 701, 650, 637, 580, 515; HRMS: $[M+Na]^+$ calcd. for $C_{23}H_{34}O_7SiNa$: 473.1971, found 473.1968.

Trimethylsilylpropargyl 6-deoxy-2,3-di-*O*-isopropylidene-4-*O*-*p*-methoxybenzyl-6-di-methoxyphosphonomethyl-α-D-mannopyranoside (17)

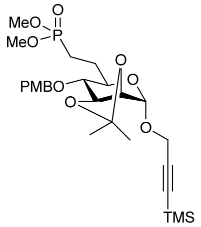

Alcohol **16** (9.05 g, 20.1 mmol, 1.0 eq.) was co-evaporated with toluene (2x) under an argon atmosphere and dissolved in dry DCM (0.10 L). The solution was cooled to -40°C, followed by the addition of pyridine (4.0 mL, 50.0 mmol, 2.5 eq.) and trifluoromethanesulfonic anhydride (5.1 mL, 30.3 mmol, 1.5 eq.). After stirring for 80 minutes at -40°C, the mixture was diluted with cold DCM and washed with cold sat. aq. $CuSO_4$ (1x), cold sat. aq. $NaHCO_3$ (1x) and cold brine (1x). The organic layer was dried over Na_2SO_4, filtered and concentrated *in vacuo* at 30°C. The obtained triflate was co-evaporated with toluene (2x) under an argon atmosphere and used without further purification. Dimethyl methylphosphonate (10 g) was co-evaporated with toluene (2x) under an argon atmosphere. 3.0 equivalents of dimethyl methylphosphonate (7.65 g, 60.8 mmol) were dissolved in dry THF (40 mL) and cooled to -70°C, followed by the addition of *n*-butyllithium (1.6 M in hexane, 38 mL, 60.8 mmol, 3.0 eq.). After 1.5 hours, a solution of the obtained triflate in dry THF (2x 17 mL) was added via a canula over 30 minutes. The reaction mixture was allowed to warm-up to -50°C over 2 hours. The reaction was subsequently quenched by the addition of a solution of AcOH in THF (60 mL, 2 M, 6.0 eq.) and diluted with EtOAc. The organic layer was washed with sat. aq. $NaHCO_3$ (1x), dried over Na_2SO_4, filtered and concentrated *in vacuo*. Purification by column chromatography (5→50% acetone in DCM + 0.1% Et_3N) afforded the title compound (7.31 g, 13.1 mmol, 65%) as an oil. R_f: 0.55 (4/1 DCM/acetone); $[\alpha]_D^{25}$ +70.6° (*c* = 0.49, $CHCl_3$); ^1H NMR ($CDCl_3$, 400 MHz, HH-COSY, HSQC): δ 7.04 (d, 2H, *J* = 8.6 Hz, Ar), 6.63 (d, 2H, *J* = 8.6 Hz, Ar), 4.94 (s, 1H, H-1), 4.59 (d, 1H, *J* = 11.3 Hz, C*H*H PMB), 4.31 (d, 1H, *J* = 11.3 Hz, C*H*H PMB), 4.03 (t, 1H, *J* = 6.3 Hz, H-3), 3.98 (d, 2H, *J* = 10.6 Hz, CH_2 propargyl), 3.95 – 3.90 (m, 1H, H-2), 3.54 (s, 3H, CH_3 PMB), 3.50 (s, 3H, OCH_3), 3.47 (s, 3H, OCH_3), 3.34 – 3.24 (m, 1H, H-5), 3.00 (dd, 1H, *J* = 9.9, 7.0 Hz, H-4), 1.97 – 1.83 (m, 1H, C*H*H-6), 1.73 – 1.58 (m, 1H, C*H*H-7), 1.58 – 1.38 (m, 2H, C*H*H-6, C*H*H-7), 1.28 (s, 3H, CH_3 isopropylidene), 1.14 (s, 3H, CH_3 isopropylidene), -0.04 (s, 9H, 3x CH_3 TMS); ^{13}C-APT NMR ($CDCl_3$, 101 MHz, HSQC): δ 158.8, 129.7 (C_q Ar), 129.2, 113.2 (Ar), 108.7 (C_q isopropylidene), 99.8 (C≡C), 94.8 (C-1), 91.4 (C≡C), 78.1 (C-3), 77.6 (C-4), 75.2 (C-2), 71.7 (CH_2 PMB), 67.6, 67.4 (C-5), 54.6 (CH_3 PMB), 54.3 (CH_2 propargyl), 51.8, 51.7, 51.7, 51.7 (OCH_3), 27.6, 25.8 (CH_3 isopropylidene), 24.0 (CH_2-6), 20.5, 19.1 (CH_2-7), -0.7 (CH_3 TMS); ^{31}P-APT NMR ($CDCl_3$, 162 MHz): δ 35.0; FT-IR (neat, cm^{-1}): 2176, 1612, 1586, 1515, 1458, 1372, 1302, 1245, 1220, 1170, 1140, 1062, 1029, 916, 842, 808, 760, 736, 701, 636, 584, 523, 486; HRMS: $[M+Na]^+$ calcd. for $C_{26}H_{41}O_9PSiNa$: 579.2155, found 579.2158.

Propargyl 6-deoxy-2,3-di-*O*-isopropylidene-4-*O*-*p*-methoxybenzyl-6-di-methoxyphosphonomethyl-α-D-mannopyranoside (18)

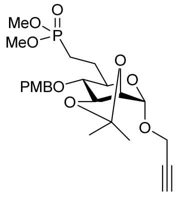

TBAF (1 M in THF, 24.5 mL, 2.0 eq.) was added to a solution of compound **17** (6.86 g, 12.1 mmol, 1.0 eq.) in THF (60 mL) at 0°C. After stirring for 15 minutes, TLC analysis showed complete conversion of the starting material. The mixture was diluted with EtOAc and washed with sat. aq. $NaHCO_3$ (1x). The organic layer was dried over Na_2SO_4, filtered and concentrated *in vacuo*. Purification by column chromatography (5→15% acetone in DCM + 0.1% Et_3N) gave compound **18** in quantitative yield (5.94 g). R_f: 0.44 (4/1 DCM/acetone); $[\alpha]_D^{25}$ +67.9° (*c* = 1.3, $CHCl_3$); ^1H NMR ($CDCl_3$, 400 MHz, HH-COSY, HSQC): δ 7.25 (m, 2H, Ar), 6.84 (d, 2H, *J* = 8.0 Hz, Ar), 5.16 (s, 1H, H-1), 4.79 (d, 1H, *J* = 11.1 Hz, C*H*H PMB), 4.50 (d, 1H, *J* = 11.1 Hz, C*H*H PMB), 4.27 – 4.21 (m, 1H, H-3), 4.18 (dd, 2H, *J* = 8.1, 2.4 Hz, CH_2 propargyl), 4.12 (d, 1H, *J* = 5.6 Hz, H-2), 3.77 (s, 3H, CH_3 PMB), 3.71 (s, 3H, OCH_3), 3.69 (s, 3H, OCH_3), 3.54 – 3.46 (m, 1H, H-5), 3.21 (dd, 1H, *J* = 9.8, 7.0 Hz, H-4), 2.44 (t, 1H, *J* = 2.4 Hz, CH propargyl), 2.17 – 2.05 (m, 1H, C*H*H-6), 1.97 – 1.79 (m, 1H, C*H*H-7), 1.79 – 1.60 (m, 2H, C*H*H-6, C*H*H-7), 1.48 (s, 3H, CH_3 isopropylidene), 1.34 (s, 3H, CH_3 isopropylidene); ^{13}C NMR (101 MHz, $CDCl_3$) δ 159.3, 130.2 (C_q Ar), 129.8,

113.8 (Ar), 109.4 (C_q isopropylidene), 95.4 (C-1), 78.6 (C-3), 78.5 (C_q propargyl), 78.4 (C-4), 75.7 (C-2), 75.3 (CH propargyl), 72.5 (CH_2 PMB), 68.3, 68.1 (C-5), 55.3 (CH_3 PMB), 54.2 (CH_2 propargyl), 52.4, 52.4 (OCH_3), 28.1, 26.3 (CH_3 isopropylidene), 24.6 (CH_2-6), 21.1, 19.7 (CH_2-7); ^{31}P-APT NMR ($CDCl_3$, 162 MHz): δ 35.2; FT-IR (neat, cm^{-1}): 3280, 2936, 1612, 1514, 1458, 1373, 1302, 1244, 1221, 1171, 1140, 1064, 1031, 916, 853, 810, 637, 591, 521; HRMS: $[M+Na]^+$ calcd. for $C_{23}H_{33}O_9PNa$: 507.1760, found 507.1760.

Propargyl 6-deoxy-6-phosphonomethy*l-α-D*-mannopyranoside (9)

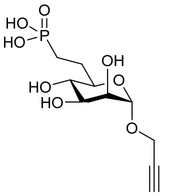

Compound **18** (1.0 g, 2.1 mmol, 1.0 eq.) was co-evaporated with toluene (2x) under an argon atmosphere and dissolved in dry MeCN (25 mL). Pyridine (1.8 mL, 22 mmol, 11 eq.) was added and the solution was cooled to 0°C. TMSBr (5.6 mL, 42 mmol, 20 eq.) was added and a glass stopper was put on the flask. After stirring at 0°C for 2 hours, the mixture was quenched with pyridine and diluted with H_2O. The obtained mixture was concentrated *in vacuo* and co-evaporated with dioxane (2x). The residue was dissolved in EtOAc and washed with brine (2x). The organic layer was dried over Na_2SO_4, filtered and concentrated *in vacuo*. The obtained intermediate (R_f = 0.14 (MeOH/DCM: 2/3 v/v) was dissolved in a mixture of AcOH/Milli-Q H_2O (1/1, v/v, 25 mL) and heated to 90°C for 8 hours, after which it was cooled to room temperature. The reaction mixture was diluted with Milli-Q H_2O and concentrated *in vacuo*. After co-evaporation with Milli-Q H_2O (5x), the residue was dissolved in Milli-Q H_2O, washed with DCM (4x) and concentrated *in vacuo*. Lyophilization yielded the title compound (0.50 g, 1.7 mmol, 81%) as a white solid. R_f: 0.40 (3/2 DCM/MeOH); ^{1}H NMR (D_2O, 500 MHz, HH-COSY, HSQC): δ 4.97 (d, 1H, *J* = 1.7 Hz, H-1), 4.36 – 4.23 (m, 2H, CH_2 propargyl), 3.92 (dd, 1H, *J* = 3.4, 1.8 Hz, H-2), 3.73 (dd, 1H, *J* = 9.3, 3.5 Hz, H-3), 3.60 – 3.47 (m, 2H, H-4, H-5), 2.90 (t, 1H, *J* = 2.4 Hz, CH propargyl), 2.13 – 2.00 (m, 1H, C*H*H-6), 1.90 – 1.75 (m, 1H, C*H*H-7), 1.75 – 1.52 (m, 2H, C*H*H-6, C*H*H-7); ^{13}C-APT NMR (D_2O, 126 MHz, HSQC): δ 98.7 (C-1), 78.9 (C_q propargyl), 76.3 (CH propargyl), 73.0, 72.9 (C-5), 70.4 (C-3), 70.0 (C-4), 70.0 (C-2), 54.6 (CH_2 propargyl), 25.2 (CH_2-6), 24.4, 23.3 (CH_2-7); ^{31}P-APT NMR (D_2O, 162 MHz, HMBC): δ 26.1; HRMS: $[M+Na]^+$ calcd. for $C_{10}H_{17}O_8PNa$: 319.0559, found 319.0566.

3-(2,3-*O*-isopropylidene-4-*O*-*p*-methoxybenzyl-α-D-mannopyranosyl)-1-propene (20)

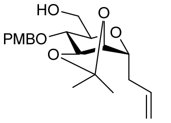

A solution of 3-(2,3-*O*-isopropylidene-4-*O*-*p*-methoxybenzyl-6-*O*-trityl-α-D-mannopyranosyl)-1-propene[1] (60.3 g, 99.4 mmol, 1.0 eq.) and *p*-toluenesulfonic acid (7.70 g, 39.7 mmol, 0.4 eq.) in DCM/MeOH (2/1 v/v, 0.66 L) was stirred for one hour, after which TLC analysis showed complete conversion of the starting material. The reaction mixture was quenched by the addition of sat. aq. $NaHCO_3$ (0.50 L) and extracted with EtOAc (3x). The combined organic layers were dried over Na_2SO_4, filtered and concentrated *in vacuo*. The obtained residue was dissolved in a mixture of 2,2-dimethoxypropane/DCM (4/1, 0.50 L). *p*-Toluenesulfonic acid (1.93 g, 9.94 mmol, 0.1 eq.) was added and the mixture was stirred for 5 minutes, after which it was quenched with sat. aq. $NaHCO_3$ (0.50 L) and extracted with EtOAc (1x). The organic layer was dried over Na_2SO_4, filtered and concentrated *in vacuo*. The obtained intermediates were dissolved in EtOAc (0.45 L) and cooled to 0°C. 1 M HCl (45 mL) was added and after 30 minutes the mixture was allowed to warm-up to room temperature. After stirring for an additional 30 minutes, TLC analysis showed complete conversion and the reaction was quenched with sat. aq. $NaHCO_3$ (0.50 L) at 0°C. The organic layer was separated and the aqueous layer was extracted with EtOAc (1x). The combined organic layers were dried over Na_2SO_4, filtered and concentrated *in vacuo*. After purification by column chromatography (20→70% Et_2O in pentane) the title compound (35.2 g, 96.6 mmol, 97%) was obtained as a clear oil. R_f: 0.53 (2/3 pentane/Et_2O); $[\alpha]_D^{25}$ +29.8° (*c* = 1.0, $CHCl_3$); ^{1}H NMR ($CDCl_3$, 400 MHz, HH-COSY, HSQC): δ 7.30 – 7.24 (m, 2H, Ar), 6.90 – 6.84 (m, 2H, Ar), 5.88 – 5.76 (m, 1H, CH_2-C*H*=CH_2), 5.17 – 5.09 (m, 2H, CH_2-CH=C*H*$_2$), 4.81 (d, 1H, *J* = 11.1 Hz, C*H*H PMB), 4.55 (d, 1H, *J* = 11.1 Hz, C*H*H PMB), 4.30 (t, 1H, *J* = 6.7 Hz, H-3), 4.07 (dd, 1H, *J* = 6.4, 4.5 Hz, H-2), 3.96 – 3.90 (m, 1H, H-1), 3.80 (s, 3H, CH_3 PMB), 3.73 (dd, 1H, *J* = 11.5, 3.0 Hz, C*H*H-6), 3.67 – 3.54 (m, 2H, H-4, C*H*H-6), 3.53 – 3.45 (m, 1H, H-5), 2.43 – 2.31 (m, 2H, C*H*$_2$-CH=CH_2), 1.97 (br, 1H, OH), 1.52 (s, 3H, CH_3 isopropylidene), 1.38 (s, 3H, CH_3 isopropylidene); ^{13}C-APT NMR (CD_3CN, 101 MHz, HSQC): δ 159.5 (C_q PMB), 133.9 (CH_2-*C*H=CH_2), 130.2 (C_q PMB), 129.9 (Ar), 118.0 (CH_2-CH=*C*H$_2$), 113.9 (Ar), 109.4 (C_q isopropylidene), 78.7 (C-3), 76.3 (C-2), 75.9 (C-4), 72.7 (C-1), 72.7 (CH_2 PMB), 71.9 (C-5), 62.9 (CH_2-6), 55.4 (CH_3 PMB), 36.4 (*C*H$_2$-CH=CH_2), 27.9, 25.9 (CH_3 isopropylidene); FT-IR (neat, cm^{-1}): 3480, 2985, 2935, 1642, 1612, 1587, 1514, 1457, 1381, 1302, 1245, 1218, 1168, 1139, 1062, 1034, 992, 918, 863, 821, 638, 582, 515; HRMS: $[M+Na]^+$ calcd. for $C_{20}H_{28}O_6Na$: 387.1784, found 387.1786.

Di-*tert*-butyl methylphosphonate S6

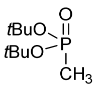

To a solution of di-*tert*-butyl phosphite (26 g, 0.13 mol, 1.0 eq.) in THF (0.25 L) was added slowly a solution of *n*-butyllithium (1.6 M in hexane, 99 ml, 0.16 mol, 1.2 eq.) at -78°C under an argon atmosphere. After stirring for 1 h, a solution of iodomethane in THF (2 M, 85 ml, 0.17 mol, 1.3 eq.) was added and the reaction was allowed to warm up to room temperature overnight. Concentration *in vacuo* and purification by column chromatography (0→50% EtOAc in pentane) yielded the title compound as a slightly yellow liquid (21.7 g, 104 mmol, 80%). ^{1}H NMR ($CDCl_3$, 400 MHz, HH-COSY, HSQC): δ 1.50 (s, 18H, 6x CH_3 *t*Bu), 1.42 (d, 3H, J = 17.4 Hz, CH_3); ^{13}C NMR ($CDCl_3$, 101 MHz, HSQC): δ 81.4, 81.3 (C_q *t*Bu), 30.4 (CH_3 *t*Bu), 17.1, 15.7 (CH_3); ^{31}P NMR (CDCl3, 162 MHz): δ 21.9; FT-IR (neat, cm^{-1}): 2980, 1370, 1310, 1257, 1173, 1040, 983, 771; HRMS: $[M+Na]^+$ calcd. for $C_9H_{21}O_3PNa$: 231.11205, found 231.11206.

3-(6-deoxy-2,3-*O*-isopropylidene-4-*O*-*p*-methoxybenzyl-*6*-di-*tert*-butoxyphosphonomethyl-α-D-mannopyranosyl)-1-propene (21)

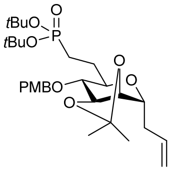

Alcohol **20** (1.11 g, 3.04 mmol, 1.0 eq.) was co-evaporated with toluene (2x) under an argon atmosphere and dissolved in dry DCM (15 mL). The solution was cooled to -40°C, followed by the addition of pyridine (0.6 mL, 7.4 mmol, 2.4 eq.) and trifluoromethanesulfonic anhydride (0.8 mL, 4.8 mmol, 1.6 eq.). After stirring for one hour at -40°C, TLC analysis showed complete conversion of the starting material. The reaction mixture was diluted with cold DCM and washed with cold sat. aq. $CuSO_4$ (1x), cold sat. aq. $NaHCO_3$ (1x) and cold brine (1x). The organic layer was dried over Na_2SO_4, filtered and concentrated *in vacuo* at 30°C. The obtained triflate was co-evaporated with toluene (2x) under an argon atmosphere and used without further purification. Di-*tert*-butyl methylphosphonate **S6** (4 g) was co-evaporated with toluene (2x) under an argon atmosphere. Di-*tert*-butyl methylphosphonate **S6** (1.85 g, 8.88 mmol, 2.9 eq.) was dissolved in dry THF (6.0 mL) and cooled to -78°C, followed by the addition of *n*-butyllithium (1.6 M in hexane, 5.7 mL, 9.1 mmol, 3.0 eq.). After two hours, a solution of the crude triflate in dry THF (2x 2.5 mL) was added via a canula over 10 minutes. The reaction mixture was allowed to warm-up to -50°C over 2.5 hours. The reaction was subsequently quenched by the addition of a solution of AcOH in THF (36 1 M, 14 mL) and diluted with EtOAc. The organic layer was washed with sat. aq. NaHCO (1x), dried over Na_2SO_4, filtered and concentrated *in vacuo*. Purification by column chromatography (5→25% acetone in DCM + 0.1% Et_3N) and size exclusions (DCM/MeOH: 1/1) gave the title compound (1.22 g, 2.20 mmol, 72%). R$_f$: 0.52 (9/1 DCM/acetone); $[\alpha]_D^{25}$ +0.87° (c = 0.11, $CHCl_3$); ^{1}H NMR ($CDCl_3$, 400 MHz, HH-COSY, HSQC): δ 7.27 (d, 2H, J = 4.2 Hz, Ar), 6.88 – 6.82 (m, 2H, Ar), 5.88 – 5.73 (m, 1H, CH_2-C*H*=CH_2), 5.14 – 5.02 (m, 2H, CH_2-CH=C*H*$_2$), 4.80 (d, 1H, J = 11.0 Hz, C*H*H PMB), 4.55 (d, 1H, J = 11.1 Hz, C*H*H PMB), 4.23 (t, 1H, J = 6.4 Hz, H-3), 4.03 (dd, 1H, J = 6.2, 4.9 Hz, H-2), 3.85 – 3.76 (m, 4H, H-1, CH_3 PMB), 3.45 – 3.31 (m, 2H, H-4, H-5), 2.43 – 2.24 (m, 2H, C*H*$_2$-CH=CH_2), 2.07 – 1.79 (m, 2H, CH_2-6/7), 1.76 – 1.54 (m, 2H, CH_2-6/7), 1.47 (s, 21H, CH_3 isopropylidene, 6x CH_3 *t*Bu), 1.37 (s, 3H, CH_3 isopropylidene); ^{13}C-APT NMR ($CDCl_3$, 101 MHz, HSQC): δ 159.4 (C_q PMB), 134.2 (CH_2-*C*H=CH_2), 130.3 (C_q PMB), 129.9 (Ar), 117.7 (CH_2-CH=*C*H$_2$), 113.9 (Ar), 109.3 (C_q isopropylidene), 81.4, 81.3 (C_q *t*Bu), 79.1 (C-4), 78.5 (C-3), 76.4 (C-2), 72.7 (CH_2 PMB), 72.3, 72.2 (C-5), 71.9 (C-1), 55.4 (CH_3 PMB), 36.7 (*C*H$_2$-CH=CH_2), 30.6, 30.5 (CH_3 *t*Bu), 28.0 (CH_3 isopropylidene), 27.4, 26.1, 26.1 (CH_2-6/7), 26.0 (CH_3 isopropylidene), 25.9 (CH_2-6/7); ^{31}P-APT NMR ($CDCl_3$, 162 MHz): δ 24.1; FT-IR (neat, cm^{-1}): 2979, 2934, 1643, 1613, 1586, 1514, 1458, 1393, 1369, 1302, 1244, 1219, 1171, 1077, 1036, 972, 916, 867, 822, 737, 696, 639, 519, 486; HRMS: $[M+H]^+$ calcd. for $C_{29}H_{48}O_8P$: 555.30813, found 555.30800.

Methyl 4-(6-deoxy-2,3-*O*-isopropylidene-4-*O*-*p*-methoxybenzyl-*6*-di-*tert*-butoxyphosphonomethyl-α-D-mannopyranosyl)-butanoate (22)

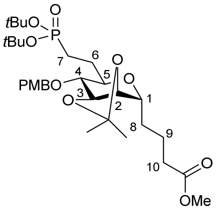

Compound **21** (8.26 g, 14.9 mmol, 1.0 eq.) was co-evaporated with toluene (2x) under an argon atmosphere, before being dissolved in dry DCE (0.15 L). Methyl acrylate (3.8 mL, 42 mmol, 2.8 eq.), CuI (0.43 g, 2.3 mmol, 0.15 eq.) and Grubbs 2nd generation catalyst (0.76 g, 0.89 mmol, 0.06 eq.) were added and the flask was covered in aluminum foil. The suspension was heated to 60°C and stirred for 48 hours, after which it was concentrated *in vacuo* and co-evaporated with toluene (5x) under an argon atmosphere. The residue was dissolved in dry DCE (75 mL) and cooled to 0°C. Two empty balloons were placed on the flask, followed by the addition of ruthenium trichloride (0.59 g, 2.8 mmol, 0.19 eq.) and $NaBH_4$ (1.80 g, 47.6 mmol, 3.2 eq.). Methanol (12.0 mL, 0.3 mol, 20 eq.) was carefully added to the suspension over 30 minutes, after which the mixture was allowed to warm-up up to room temperature over 15 minutes. The mixture was subsequently heated to 45°C for 5.5 hours. The reaction mixture was cooled to room temperature, diluted with brine and extracted with DCM (3x). The combined organic layers were dried over Na_2SO_4, filtered and concentrated *in vacuo*. Purification by column chromatography (10→30% acetone in pentane + 0.1% Et_3N)

afforded compound **22** (6.59 g, 10.7 mmol, 72% over two steps). R_f: 0.73 (7/3 DCM/acetone); $[\alpha]_D^{25}$ +15.5° (*c* = 0.53, $CHCl_3$); ^{1}H NMR ($CDCl_3$, 400 MHz, HH-COSY, HSQC): δ 7.23 (d, 2H, *J* = 8.7 Hz, Ar), 6.83 – 6.77 (m, 2H, Ar), 4.75 (d, 1H, *J* = 11.1 Hz, C*H*H PMB), 4.50 (d, 1H, *J* = 11.1 Hz, C*H*H PMB), 4.17 (t, 1H, *J* = 6.4 Hz, H-3), 3.93 (dd, 1H, *J* = 6.2, 4.9 Hz, H-2), 3.73 (s, 3H, CH_3 PMB), 3.71 – 3.65 (m, 1H, H-1), 3.60 (s, 3H, OCH_3), 3.36 – 3.27 (m, 2H, H-4, H-5), 2.32 – 2.23 (m, 2H, CH_2-10), 2.02 – 1.89 (m, 1H, C*H*H-6/7/8/9), 1.89 – 1.69 (m, 2H, CH_2-6/7/8/9), 1.69 – 1.49 (m, 5H, CH_2-6/7/8/9), 1.47 (s, 3H, CH_3 isopropylidene), 1.45 – 1.40 (m, 18H, 6x CH_3 *t*Bu), 1.31 (s, 3H, CH_3 isopropylidene); ^{13}C-APT NMR ($CDCl_3$, 101 MHz, HSQC): δ 173.6 (C=O), 159.2, 130.2 (C_q PMB), 129.7, 113.7 (Ar), 109.1 (C_q isopropylidene), 81.4, 81.3, 81.3, 81.3 (C_q *t*Bu), 78.8 (C-4), 78.3 (C-3), 76.9 (C-2), 72.5 (CH_2 PMB), 71.9, 71.8 (C-5), 71.7 (C-1), 55.2 (CH_3 PMB), 51.5 (OCH_3), 33.5 (CH_2-10), 31.2 (CH_2-9), 30.4, 30.4, 30.3, 30.3 (CH_3 *t*Bu), 27.8 (CH_3 isopropylidene), 27.1, 26.0, 26.0 (CH_2-6 or CH_2-7), 25.8 (CH_3 isopropylidene), 25.6 (CH_2-6 or CH_2-7), 21.0 (CH_2-8); ^{31}P-APT NMR ($CDCl_3$, 162 MHz): δ 24.5; FT-IR (neat, cm^{-1}): 2979, 2935, 1737, 1612, 1514, 1457, 1393, 1369, 1302, 1244, 1219, 1170, 1082, 1037, 1006, 974, 918, 866, 823, 791, 739, 695, 520, 487; HRMS: $[M+Na]^+$ calcd. for $C_{31}H_{51}O_{10}PNa$: 637.3118, found 637.3124.

4-(6-deoxy-2,3-*O*-isopropylidene-4-*O*-*p*-methoxybenzyl-*6*-di-*tert*-butoxyphosphonomethyl-α-D-mannopyranosyl)-butanoic acid (23)

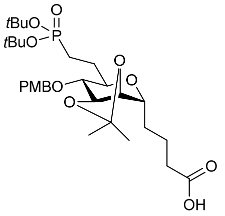

Methyl ester **22** (6.59 g, 10.7 mmol, 1.0 eq.) was dissolved in in a mixture of THF/H_2O (4/1, v/v, 0.10 L). LiOH (1.35 g, 32 mmol, 3.0 eq.) was added and the mixture was stirred for 7 hours, after which TLC analysis showed complete conversion of the starting material. The reaction mixture was cooled to 0°C, acidified with 3 M HCl to pH = 4-5 and extracted with DCM (2x). The combined organic layers were dried over Na_2SO_4, filtered and concentrated *in vacuo*. The title compound was obtained in quantitative yield (6.65 g) and used without further purification. R_f: 0.28 (7/3 DCM/acetone); $[\alpha]_D^{25}$ +20.0° (*c* = 0.18, $CHCl_3$); ^{1}H NMR ($CDCl_3$, 400 MHz, HH-COSY, HSQC): δ 7.27 (d, 2H, J = 8.7 Hz, Ar), 6.85 (d, 2H, J = 8.6 Hz, Ar), 4.80 (d, 1H, J = 11.1 Hz, C*H*H PMB), 4.54 (d, 1H, J = 11.1 Hz, C*H*H PMB), 4.22 (t, 1H, J = 6.6 Hz, H-3), 3.97 (t, 1H, J = 6.1 Hz, H-2), 3.79 (s, 3H, CH_3 PMB), 3.73 – 3.65 (m, 1H, H-1), 3.43 – 3.34 (m, 2H, H-4, H-5), 2.35 (t, 2H, J = 7.2 Hz, CH_2-10), 2.03 – 1.57 (m, 8H, CH_2-6/7/8/9), 1.56 – 1.42 (m, 21H, CH_3 isopropylidene, 6x CH_3 *t*Bu), 1.36 (s, 3H, CH_3 isopropylidene); ^{13}C-APT NMR ($CDCl_3$, 101 MHz, HSQC): δ 176.1 (C=O), 159.4, 130.4 (C_q PMB), 129.9, 113.9 (Ar), 109.4 (C_q isopropylidene), 82.4, 82.3, 82.3, 82.2 (C_q *t*Bu), 79.1 (C-4), 78.7 (C-3), 77.3 (C-2), 72.6 (CH_2 PMB), 72.4, 72.2 (C-5), 71.7 (C-1), 55.4 (CH_3 PMB), 34.1 (CH_2-10), 31.9 (CH_2-8), 30.5, 30.5, 30.5, 30.5 (CH_3 *t*Bu), 27.9 (CH_3 isopropylidene), 27.0, 26.1, 26.1 (CH_2-6/7), 25.7 (CH_3 isopropylidene), 25.5 (CH_2-6/7), 21.2 (CH_2-9); ^{31}P-APT NMR ($CDCl_3$, 162 MHz): δ 24.8; FT-IR (neat, cm^{-1}): 2980, 2935, 1724, 1613, 1586, 1514, 1458, 1394, 1370, 1302, 1245, 1217, 1158, 1081, 1037, 980, 918, 867, 822, 793, 735, 701, 661, 519, 486; HRMS: $[M+Na]^+$ calcd. for $C_{30}H_{49}O_{10}PNa$: 623.2961, found 623.2971.

***N*$_\alpha$-Fmoc-*N*$_\varepsilon$-[butan-4-(6-deoxy-2,3-*O*-isopropylidene-4-*O*-*p*-methoxybenzyl-*6*-di-*tert*-butoxyphosphonomethyl-α-D-mannopyranosyl)-amide]-L-lysine-methyl ester (24)**

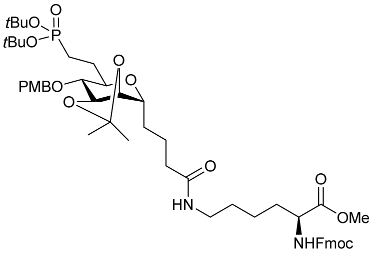

Compound **23** (1.78 g, 2.97 mmol, 1.0 eq.) and Fmoc-L-lysine-OMe[1] (1.39 g, 3.32 mmol, 1.12 eq.) were dissolved in DMF (15 mL). HCTU (1.47 g, 3.55 mmol, 1.2 eq.) and DIPEA (1.6 mL, 9.2 mmol, 3.0 eq.) were added and the solution was stirred for 2 hours. The reaction mixture was diluted with EtOAc and was washed with 1 M HCl (1x), sat. aq. $NaHCO_3$ (1x), brine (1x). The organic layer was dried over Na_2SO_4, filtered and concentrated *in vacuo*. Purification by column chromatography (10→100% acetone in DCM) gave compound **24** (2.25 g, 2.33 mmol, 78%) as an oil. R_f: 0.31 (9/1 DCM/MeOH); $[\alpha]_D^{25}$ +14.7 (*c* = 0.44, $CHCl_3$); ^{1}H NMR ($CDCl_3$, 500 MHz, HH-COSY, HSQC): δ 7.76 (d, 2H, *J* = 7.5 Hz, Ar), 7.61 (t, 2H, *J* = 8.1 Hz, Ar), 7.40 (t, 2H, *J* = 7.4 Hz, Ar), 7.31 (t, 2H, *J* = 7.5 Hz, Ar), 7.29 – 7.23 (m, 2H, Ar), 6.85 (d, 2H, *J* = 8.5 Hz, Ar), 6.68 (br, 1H, NH), 5.60 (d, 1H, *J* = 8.1 Hz, N*H*Fmoc), 4.78 (d, 1H, *J* = 11.2 Hz, C*H*H PMB), 4.52 (d, 1H, *J* = 11.3 Hz, C*H*H PMB), 4.45 – 4.38 (m, 1H, C*H*H Fmoc), 4.38 – 4.30 (m, 2H, CH L-Lys, C*H*H Fmoc), 4.26 – 4.18 (m, 2H, H-3, CH Fmoc), 3.95 (t, 1H, *J* = 6.2 Hz, H-2), 3.79 (s, 3H, CH_3 PMB), 3.74 (s, 3H, OCH_3), 3.63 – 3.55 (m, 1H, H-1), 3.42 – 3.32 (m, 2H, H-4, H-5), 3.32 – 3.20 (m, 1H, C*H*H ε-L-Lys), 3.20 – 3.12 (m, 1H, C*H*H ε-L-Lys), 2.25 – 2.13 (m, 2H, CH_2-10), 1.99 – 1.52 (m, 10H, 5x CH_2-6/7/8/9, β/γ/δ-L-Lys), 1.51 – 1.43 (m, 23H, 1x CH_2-6/7/8/9, β/γ/δ-L-Lys, CH_3 isopropylidene, 6x CH_3 *t*Bu), 1.40 – 1.32 (m, 5H, 1x CH_2-6/7/8/9, β/γ/δ-L-Lys, CH_3 isopropylidene); ^{13}C-APT NMR ($CDCl_3$, 126 MHz, HSQC): δ 173.1 (C=O), 159.4 (C_q PMB), 156.2 (C=O), 144.1, 143.9, 141.4 (C_q Fmoc), 130.4 (C_q PMB), 129.9, 127.8, 127.2, 125.3, 125.2, 120.1, 113.9 (Ar), 109.4 (C_q isopropylidene), 81.8, 81.8, 81.7, 81.6 (C_q *t*Bu), 79.1 (C-4), 78.6 (C-3), 78.6 (C-2), 72.8, 72.6 (C-5), 72.6 (CH_2 PMB), 71.6 (C-1), 67.1 (CH_2 Fmoc), 55.4 (CH_3 PMB), 53.9 (CH L-Lys), 52.5 (OCH_3), 47.3 (CH Fmoc), 39.0 (CH_2

ε-L-Lys), 36.2 (CH_2-10), 32.1 (CH_2 β-L-Lys), 31.9 (CH_2 δ-L-Lys), 30.6, 30.5, 30.5 (CH_3 *t*Bu), 29.2, 27.8 (CH_3 isopropylidene), 27.2, 26.2, 26.2, 26.0 (CH_2-6/7), 25.6 (CH_3 isopropylidene), 22.6 (CH_2 γ-L-Lys), 22.2 (CH_2-9); ^{31}P-APT NMR ($CDCl_3$, 162 MHz): δ 24.3; FT-IR (neat, cm^{-1}): 3281, 2980, 2935, 1721, 1650, 1613, 1514, 1451, 1370, 1246, 1172, 1082, 1037, 981, 916, 867, 823, 760, 732, 646, 621, 538; HRMS: $[M+Na]^+$ calcd. for $C_{52}H_{73}N_2O_{13}PNa$: 987.4748, found 987.4761.

N_α-Fmoc-N_ε-[butan-4-(6-deoxy-2,3-*O*-isopropylidene-4-*O*-*p*-methoxybenzyl-*6*-di-*tert*-butoxyphosphonomethyl-α-D-mannopyranosyl)-amide]-L-lysine (10)

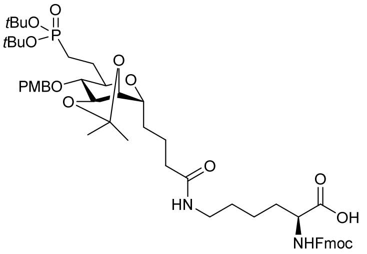

Compound **24** (2.20 g, 2.28 mmol, 1.0 eq.) was dissolved in THF (23 mL) and cooled to 0°C. A solution of LiOH in H_2O (0.30 M, 15 mL, 4.5 mmol, 2.0 eq.) was added and the mixture was stirred vigorously for 40 minutes, after which the mixture was acidified by the addition of 1 M HCl to pH = 3-4 and diluted with brine. The mixture was extracted with EtOAc (2x) and the combined organic layers were dried over Na_2SO_4, filtered and concentrated *in vacuo*. After purification by column chromatography (2→10% MeOH in DCM + 0.1% AcOH), the title compound (1.68 g, 1.77 mmol, 78%) was obtained as a white foam. R_f: 0.36 (9/1 DCM/MeOH + 0.1% AcOH); $[\alpha]_D^{25}$ +31.9° (*c* = 0.32, DCM); ^{1}H NMR (MeOD, 500 MHz, HH-COSY, HSQC): δ 7.78 (d, 2H, *J* = 7.6 Hz, Ar), 7.67 (t, 2H, *J* = 8.4 Hz, Ar), 7.38 (td, 2H, *J* = 7.4, 1.1 Hz, Ar), 7.30 (td, 2H, *J* = 7.5, 1.2 Hz, Ar), 7.28 – 7.23 (m, 2H, Ar), 6.89 – 6.84 (m, 2H, Ar), 4.75 (d, 1H, *J* = 11.3 Hz, CH_2 PMB), 4.53 (d, 1H, *J* = 11.3 Hz, CH_2 PMB), 4.35 (dd, 2H, *J* = 7.0, 2.4 Hz, CH_2 Fmoc), 4.23 – 4.18 (m, 2H, H-3, CH Fmoc), 4.13 (dd, 1H, *J* = 9.2, 4.6 Hz, CH L-Lys), 4.00 – 3.96 (m, 1H, H-2), 3.77 (s, 3H, CH_3 PMB), 3.71 – 3.65 (m, 1H, H-1), 3.40 – 3.33 (m, 2H, H-4, H-5), 3.19 – 3.13 (m, 2H, CH_2 ε-L-Lys), 2.22 – 2.15 (m, 2H, CH_2-10), 1.99 – 1.38 (m, 35H, 7x CH_2-6/7/8/9, β/γ/δ-L-Lys, CH_3 isopropylidene, 6x CH_3 *t*Bu), 1.33 (s, 3H, CH_3 isopropylidene); ^{13}C-APT NMR (MeOD, 126 MHz, HSQC): δ 175.6, 160.8 (C=O), 158.6 (C_q PMB), 145.4, 145.2, 142.6 (C_q Fmoc), 131.6 (C_q PMB), 130.9, 128.8, 128.2, 128.2, 126.3, 126.2, 121.0, 114.7 (Ar), 110.3 (C_q isopropylidene), 83.5, 83.5, 83.5, 83.5 (C_q *t*Bu), 79.7 (C-4), 79.7 (C-3), 78.2 (C-2), 73.2 (CH_2 PMB), 73.2 (C-1), 72.8, 72.7 (C-5), 67.9 (CH_2 Fmoc), 55.7 (CH_3 PMB), 55.5 (CH Fmoc), 48.4 (CH L-Lys), 40.1 (ε-L-Lys), 36.6 (CH_2-10), 32.5 (CH_2 β-L-Lys), 32.3 (CH_2 δ-L-Lys), 30.8, 30.8, 30.7, 30.7, 30.0 (CH_3 *t*Bu), 28.2 (CH_3 isopropylidene), 27.6, 27.1, 27.1, 26.4 (CH_2-6/7), 25.9 (CH_3 isopropylidene), 24.3 (CH_2 γ-L-Lys), 23.2 (CH_2-9); ^{31}P-APT NMR (MeOD, 202 MHz, HMBC): δ 25.7; FT-IR (neat, cm^{-1}): 3301, 2980, 2935, 1716, 1650, 1613, 1514, 1451, 1394, 1370, 1246, 1161, 1081, 1038, 985, 912, 866, 823, 760, 730, 647, 621, 540; HRMS: $[M+Na]^+$ calcd. for $C_{51}H_{71}N_2O_{13}PNa$: 973.4591, found 973.4603.

Ac-Lys(N_3)-Lys(N_3)-Lys(N_3)-Lys(N_3)-Lys(N_3)-Lys(N_3)-Asp-Glu-Val-Ser-Gly-Leu-Glu-Gln-Leu-Glu-Ser-Ile-Ile-Asn-Phe-Glu-Lys-Leu-Ala-Ala-Ala-Ala-Ala-Lys-NH_2 (26)

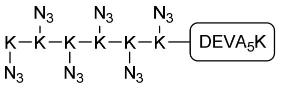

100 μmol of crude H-Asp(O*t*Bu)-Glu(O*t*Bu)-Val-Ser(*t*Bu)-Gly-Leu-Glu(O*t*Bu)-Gln(Trt)-Leu-Glu(O*t*Bu)-Ser(*t*Bu)-Ile-Ile-Asn(Trt)-Phe-Glu(O*t*Bu)-Lys(Boc)-Leu-Ala-Ala-Ala-Ala-Ala-Lys(MMT)-Tentagel(SRam) was elongated with Lys(N_3)-Lys(N_3)-Lys(N_3)-Lys(N_3)-Lys(N_3)-Lys(N_3) with standard HCTU/Fmoc chemistry concluding in final Fmoc removal with a solution of 20% piperidine in DMF (3x 3 min). The resin was treated with a mixture of Ac_2O/DMF/DIPEA (2x 2.0 mL, 20 min), and washed with DMF (3x) and DCM (3x). The peptide was cleaved from the resin after treatment with TFA/TIS/H_2O (95/2.5/2.5 v/v/v) (4.0 mL) for 3 hours the suspension was filtered and the residue was washed with the cleavage cocktail (4.0 mL). The product was precipitated with Et_2O. After purification by RP-HPLC and lyophilisation, compound **26** (4.6 mg, 1.3 μmol, 1%) was obtained as a white solid. LC-MS: Rt = 6.91 min (C18 Gemini, 10 - 90% MeCN, 11 min run); ESI-MS (m/z) found 1757.0 $[M+2H]^{2+}$; HRMS: $[M+2H]^{2+}$ calcd. for $C_{150}H_{249}N_{53}O_{45}$: 1756.44071, found 1756.44051.

Ac-Lys(N_3)-Lys(N_3)-Lys(N_3)-Lys(N_3)-Lys(N_3)-Lys(N_3)-Asp-Glu-Val-Ser-Gly-Leu-Glu-Gln-Leu-Glu-Ser-Ile-Ile-Asn-Phe-Glu-Lys-Leu-Ala-Ala-Ala-Ala-Ala-Lys(4-((6-amino-2-butoxy-8-oxo-7,8-dihydro-9H-purin-9-yl)methyl)-N-(2-(2-(2-amino-2-oxoethoxy)ethoxy)ethyl)benzamide)-NH_2 (27)

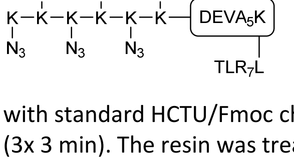

50 μmol of crude H-Lys(N_3)-Lys(N_3)-Lys(N_3)-Lys(N_3)-Lys(N_3)-Lys(N_3)-Asp(O*t*Bu)-Glu(O*t*Bu)-Val-Ser(*t*Bu)-Gly-Leu-Glu(O*t*Bu)-Gln(Trt)-Leu-Glu(O*t*Bu)-Ser(*t*Bu)-Ile-Ile-Asn(Trt)-Phe-Glu(O*t*Bu)-Lys(Boc)-Leu-Ala-Ala-Ala-Ala-Ala-Lys(MMT)-Tentagel(SRam) was elongated with Lys(N_3)-Lys(N_3)-Lys(N_3)-Lys(N_3)-Lys(N_3)-Lys(N_3) with standard HCTU/Fmoc chemistry concluding in final Fmoc removal with a solution of 20% piperidine in DMF (3x 3 min). The resin was treated with a mixture of Ac_2O/DMF/DIPEA (2x 1.0 mL, 20 min), and washed with DMF (3x) and DCM (3x). The peptide was treated with a continuous flow of a mixture of TFA/TIS/DCM (96/2/2 v/v/v,

15 mL) over 5 minutes. The resin was washed subsequently with DCM (5x), TFA/TIS/DCM (96/2/2 v/v/v, 2 mL), DCM (5x), 1 M DIPEA in NMP (2 mL), DCM (3x) and DMF (3x). A solution of {2-[2-(Fmoc-amino)ethoxy]ethoxy}acetic acid (41 mg, 106 µmol, 2.1 eq.) and HCTU (42 mg, 101 µmol, 2.0 eq.) in DMF (1.0 mL) and DIPEA (35 µL, 201 µmol, 4.0 eq.) were added and the suspension was shaken overnight. The resin was washed with DMF (5x), treated with 20% piperidine in DMF (1.0 mL, 2x 20 min) and washed with DMF (5x). A solution of 4-((2-butoxy-6-((*tert*-butoxycarbonyl)amino)-8-oxo-7,8-dihydro-9H-purin-9-yl)methyl)benzoid acid (47 mg, 102 µmol, 2.0 eq.) and HCTU (42 mg, 100 µmol, 2.0 eq.) and DIPEA (35 µL, 200 µmol, 4.0 eq.) were added and the suspension was shaken overnight. The peptide was cleaved from the resin after treatment with TFA/TIS/H_2O (95/2.5/2.5 v/v/v) (2.0 mL) for 3 hours the suspension was filtered and the residue was washed with the cleavage cocktail (2.0 mL). The product was precipitated with Et_2O. After purification by RP-HPLC and lyophilisation, compound **27** (4.0 mg, 1.1 µmol, 2%) was obtained as a white solid. LC-MS: Rt = 7.13 min (C18 Gemini, 10 - 90% MeCN, 11 min run); ESI-MS (m/z) found 1999.8 $[M+2H]^{2+}$; HRMS: $[M+3H]^{3+}$ calcd. for $C_{173}H_{278}N_{59}O_{51}$: 1332.69857, found 1332.69879.

Ac-Asp-Glu-Val-Ser-Gly-Leu-Glu-Gln-Leu-Glu-Ser-Ile-Ile-Asn-Phe-Glu-Lys-Leu-Ala-Ala-Ala-Ala-Ala-Lys-Lys(N_3)-Lys(N_3)-Lys(N_3)-Lys(N_3)-Lys(N_3)-Lys(N_3)-NH_2 (29)

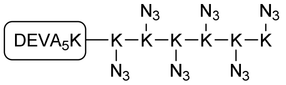

100 µmol of crude H-Asp(O*t*Bu)-Glu(O*t*Bu)-Val-Ser(*t*Bu)-Gly-Leu-Glu(O*t*Bu)-Gln(Trt)-Leu-Glu(O*t*Bu)-Ser(*t*Bu)-Ile-Ile-Asn(Trt)-Phe-Glu(O*t*Bu)-Lys(Boc)-Leu-Ala-Ala-Ala-Ala-Ala-Lys(MMT)-Tentagel(SRam) was elongated with Lys(N_3)-Lys(N_3)-Lys(N_3)-Lys(N_3)-Lys(N_3)-Lys(N_3) with standard HCTU/Fmoc chemistry concluding in final Fmoc removal with a solution of 20% piperidine in DMF (3x 3 min). The resin was treated with a mixture of Ac_2O/DMF/DIPEA (2x 2.0 mL, 20 min), and washed with DMF (3x) and DCM (3x). The peptide was cleaved from the resin after treatment with TFA/TIS/H_2O (95/2.5/2.5 v/v/v) (4.0 mL) for 3 hours the suspension was filtered and the residue was washed with the cleavage cocktail (4.0 mL). The product was precipitated with Et_2O. After purification by RP-HPLC and lyophilisation, compound **29** (17.4 mg, 5.5 µmol, 6%) was obtained as a white solid. LC-MS: Rt = 7.11 min (C18 Gemini, 10 - 90% MeCN, 11 min run); ESI-MS (m/z) found 1757.4 $[M+2H]^{2+}$; HRMS: $[M+2H]^{2+}$ calcd. for $C_{150}H_{249}N_{53}O_{45}$: 1756.44071, found 1756.44041.

(4-((6-amino-2-butoxy-8-oxo-7,8-dihydro-9H-purin-9-yl)methyl)-N-(2-(2-(2-amino-2-oxoethoxy)ethoxy)ethyl)benzamide)-Asp-Glu-Val-Ser-Gly-Leu-Glu-Gln-Leu-Glu-Ser-Ile-Ile-Asn-Phe-Glu-Lys-Leu-Ala-Ala-Ala-Ala-Ala-Lys-Lys(N_3)-Lys(N_3)-Lys(N_3)-Lys(N_3)-Lys(N_3)-Lys(N_3)-NH_2 (30)

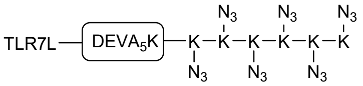

50 µmol of crude H-Lys(N_3)-Lys(N_3)-Lys(N_3)-Lys(N_3)-Lys(N_3)-Lys(N_3)-Asp(O*t*Bu)-Glu(O*t*Bu)-Val-Ser(*t*Bu)-Gly-Leu-Glu(O*t*Bu)-Gln(Trt)-Leu-Glu(O*t*Bu)-Ser(*t*Bu)-Ile-Ile-Asn(Trt)-Phe-Glu(O*t*Bu)-Lys(Boc)-Leu-Ala-Ala-Ala-Ala-Ala-Lys(MMT)-Tentagel(SRam) was elongated with Lys(N_3)-Lys(N_3)-Lys(N_3)-Lys(N_3)-Lys(N_3)-Lys(N_3) with standard HCTU/Fmoc chemistry concluding in final Fmoc removal with a solution of 20% piperidine in DMF (3x 3 min). The resin was washed with DMF (5x), followed by the addition of a solution of {2-[2-(Fmoc-amino)ethoxy]ethoxy}acetic acid (41 mg, 106 µmol, 2.1 eq.) and HCTU (42 mg, 101 µmol, 2.0 eq.) in DMF (1.0 mL) and DIPEA (35 µL, 201 µmol, 4.0 eq.). The suspension was shaken overnight. The resin was washed with DMF (5x), treated with 20% piperidine in DMF (1.0 mL, 2x 20 min) and washed with DMF (5x). A solution of 4-((2-butoxy-6-((*tert*-butoxycarbonyl)amino)-8-oxo-7,8-dihydro-9H-purin-9-yl)methyl)benzoid acid (47 mg, 102 µmol, 2.0 eq.) and HCTU (42 mg, 100 µmol, 2.0 eq.) and DIPEA (35 µL, 200 µmol, 4.0 eq.) were added and the suspension was shaken overnight. The peptide was cleaved from the resin after treatment with TFA/TIS/H_2O (95/2.5/2.5 v/v/v) (2.0 mL) for 3 hours the suspension was filtered and the residue was washed with the cleavage cocktail (2.0 mL). The product was precipitated with Et_2O. After purification by RP-HPLC and lyophilisation, compound **30** (8.2 mg, 2.1 µmol, 2%) was obtained as a white solid. LC-MS: Rt = 7.11 min (C18 Gemini, 10 - 90% MeCN, 11 min run); ESI-MS (m/z) found 1978.7 $[M+2H]^{2+}$; HRMS: $[M+3H]^{3+}$ calcd. for $C_{171}H_{276}N_{59}O_{50}$: 1318.69505, found 1318.69517.

Ac-Lys(M6Po)-Lys(M6Po)-Lys(M6Po)-Lys(M6Po)-Lys(M6Po)-Lys(M6Po)-Asp-Glu-Val-Ser-Gly-Leu-Glu-Gln-Leu-Glu-Ser-Ile-Ile-Asn-Phe-Glu-Lys-Leu-Ala-Ala-Ala-Ala-Ala-Lys-NH₂ (1)

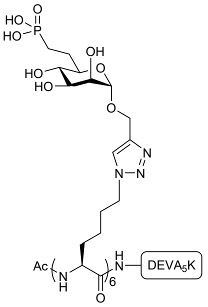

Azide **26** (4.24 mg, 1.21 µmol, 1.0 eq.) was dissolved in DMSO (641 µL), followed by the addition of a solution of compound **9** in DMSO (91.1 mM, 120 µL, 10.9 µmol, 9.0 eq.). 20 mM Tris/150 mM NaCl buffer (787 µL) and click-mixture (24 µL, 26 mg/mL $CuSO_4$ in H_2O, 120 mg/mL NaAsc in H_2O, 52 mg/mL TBTA in DMSO) were added. The reaction vessel was shaken for 3 hours, after which LC-MS analysis showed complete conversion. The reaction was quenched by the addition of EDTA (6.6 µL, 0.5 M in H_2O) and mixed for 15 minutes. After purification by RP-HPLC and lyophilisation, compound **1** (0.3 mg, 64 nmol, 5%) was obtained as a white solid. LC-MS: Rt = 4.56 min (C18 Gemini, 10 - 90% MeCN, 11 min run); HRMS: $[M+3H]^{3+}$ calcd. for $C_{210}H_{352}N_{53}O_{93}P_6$: 1763.42844, found 1763.42972.

Ac-Lys(M6Po)-Lys(M6Po)-Lys(M6Po)-Lys(M6Po)-Lys(M6Po)-Lys(M6Po)-Asp-Glu-Val-Ser-Gly-Leu-Glu-Gln-Leu-Glu-Ser-Ile-Ile-Asn-Phe-Glu-Lys-Leu-Ala-Ala-Ala-Ala-Ala-Lys(4-((6-amino-2-butoxy-8-oxo-7,8-dihydro-9H-purin-9-yl)methyl)-N-(2-(2-(2-amino-2-oxoethoxy)ethoxy)ethyl)benzamide)-NH₂ (2)

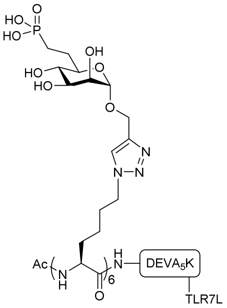

Azide **27** (3.7 mg, 0.93 µmol, 1.0 eq.) was dissolved in DMSO (491 µL), followed by the addition of a solution of compound **9** in DMSO (91.1 mM, 92 µL, 8.33 µmol, 9.0 eq.). 20 mM Tris/150 mM NaCl buffer (604 µL) and click-mixture (18 µL, 26 mg/mL $CuSO_4$ in H_2O, 120 mg/mL NaAsc in H_2O, 52 mg/mL TBTA in DMSO) were added. The reaction vessel was shaken for 3 hours, after which LC-MS analysis showed complete conversion. The reaction was quenched by the addition of EDTA (5.0 µL, 0.5 M in H_2O) and mixed for 15 minutes. After purification by RP-HPLC and lyophilisation, compound **2** (1.0 mg, 165 nmol, 18%) was obtained as a white solid. LC-MS: Rt = 4.89 min (C18 Gemini, 10 - 90% MeCN, 11 min run); HRMS: $[M+3H]^{3+}$ calcd. for $C_{233}H_{380}N_{59}O_{99}P_6$: 1924.83078, found 1924.83260.

Ac-Asp-Glu-Val-Ser-Gly-Leu-Glu-Gln-Leu-Glu-Ser-Ile-Ile-Asn-Phe-Glu-Lys-Leu-Ala-Ala-Ala-Ala-Ala-Lys-Lys(M6Po)-Lys(M6Po)-Lys(M6Po)-Lys(M6Po)-Lys(M6Po)-Lys(M6Po)-NH₂ (3)

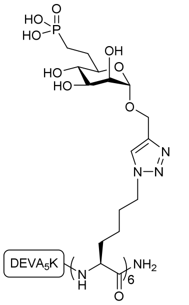

Azide **29** (3.2 mg, 0.92 µmol, 1.0 eq.) was dissolved in DMSO (480 µL), followed by the addition of a solution of compound **9** in DMSO (91.1 mM, 100 µL, 9.11 µmol, 9.0 eq.). 20 mM Tris/150 mM NaCl buffer (600 µL) and click-mixture (18 µL, 26 mg/mL $CuSO_4$ in H_2O, 120 mg/mL NaAsc in H_2O, 52 mg/mL TBTA in DMSO) were added. The reaction vessel was shaken for 2.5 hours, after which LC-MS analysis showed complete conversion. The reaction was quenched by the addition of EDTA (10 µL, 0.5 M in H_2O) and mixed for 15 minutes. After purification by RP-HPLC and lyophilisation, compound **3** (0.9 mg, 0.17 µmol, 18%) was obtained as a white solid. LC-MS: Rt = 4.45 min (C18 Gemini, 10 - 90% MeCN, 11 min run); HRMS: $[M+3H]^{3+}$ calcd. for $C_{210}H_{352}N_{53}O_{93}P_6$: 1763.42844, found 1763.43069.

(4-((6-amino-2-butoxy-8-oxo-7,8-dihydro-9H-purin-9-yl)methyl)-N-(2-(2-(2-amino-2-oxoethoxy)ethoxy)ethyl)benzamide)-Asp-Glu-Val-Ser-Gly-Leu-Glu-Gln-Leu-Glu-Ser-Ile-Ile-Asn-Phe-Glu-Lys-Leu-Ala-Ala-Ala-Ala-Ala-Lys-Lys(M6Po)-Lys(M6Po)-Lys(M6Po)-Lys(M6Po)-Lys(M6Po)-Lys(M6Po)-NH₂ (4)

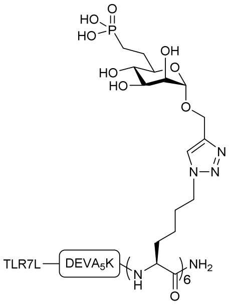

Azide **30** (7.4 mg, 1.86 µmol, 1.0 eq.) was dissolved in DMSO (989 µL), followed by the addition of a solution of compound **9** in DMSO (91.1 mM, 184 µL, 16.8 µmol, 9.0 eq.). 20 mM Tris/150 mM NaCl buffer (1.21 mL) and click-mixture (36 µL, 26 mg/mL $CuSO_4$ in H_2O, 120 mg/mL NaAsc in H_2O, 52 mg/mL TBTA in DMSO) were added. The reaction vessel was shaken for 3 hours, after which LC-MS analysis showed complete conversion. The reaction was quenched by the addition of EDTA (10 µL, 0.5 M in H_2O) and mixed for 15 minutes. After purification by RP-HPLC and lyophilisation, compound **4** (3.3 mg, 0.58 µmol, 31%) was obtained as a white solid. LC-MS: Rt = 4.71 min (C18 Gemini, 10 - 90% MeCN, 11 min run); HRMS: $[M+4H]^{4+}$ calcd. for $C_{231}H_{379}N_{59}O_{98}P_6$: 1433.37226, found 1433.37178.

Ac-[Lys(butan-4-(6-deoxy-*6*-phosphonomethyl-α-D-mannopyranosyl)-amide)]$_6$-Ile-Ser-Gln-Ala-Val-His-Ala-Ala-His-Ala-Glu-Ile-Asn-Glu-Ala-Gly-Arg-Lys-NH$_2$ (5)

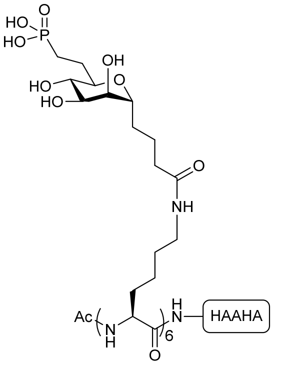

100 µmol of crude H-Ile-Ser(*t*Bu)-Gln(Trt)-Ala-Val-His(Trt)-Ala-Ala-His(Trt)-Ala-Glu(O*t*Bu)-Ile-Asn(Trt)-Glu(O*t*Bu)-Ala-Gly-Arg(Pbf)-Lys(MMT)-Tentagel(SRam) was washed with DMF (5x) and elongated by the addition of a solution of acid **10** (0.19 g, 0.20 mmol, 2.0 eq.) and HCTU (84 mg, 0.20 µmol, 2.0 eq.) in DMF (2.0 mL) and DIPEA (70 µL, 0.40 mmol, 4.0 eq.). The suspension was shaken overnight. The resin was washed with DMF (5x), treated with 20% piperidine in DMF (2.0 mL, 2x 20 min) and washed with DMF (5x). This was repeated another 5 times, after which the resin was treated with a mixture of Ac_2O/DMF/DIPEA (2x 2.0 mL, 20 min), and washed with DMF (3x) and DCM (3x). 30 µmol of crude Ac-[Lys(butan-4-(6-deoxy-2,3-*O*-isopropylidene-4-*O*-*p*-methoxybenzyl-*6*-di-*tert*-butoxyphosphonomethyl-*α*-D-mannopyranosyl)-amide)]$_6$-Ile-Ser(*t*Bu)-Gln(Trt)-Ala-Val-His(Trt)-Ala-Ala-His(Trt)-Ala-Glu(O*t*Bu)-Ile-Asn(Trt)-Glu(O*t*Bu)-Ala-Gly-Arg(Pbf)-Lys(MMT)-Tentagel(SRam) was taken and the peptide was cleaved from the resin after treatment with TFA/TIS/H_2O (95/2.5/2.5 v/v/v) (1.2 mL) for 3 hours the suspension was filtered and the residue was washed with the cleavage cocktail (1.2 mL). The product was precipitated with Et_2O. After purification by RP-HPLC and lyophilisation, compound **5** (13.3 mg, 2.9 µmol, 10%) was obtained as a white solid. LC-MS: Rt = 6.19 min (C18 Gemini, 0 - 50% MeCN, 11 min run); ESI-MS (m/z) found 1525.6 $[M+3H]^{3+}$; HRMS: $[M+4H]^{4+}$ calcd. for $C_{184}H_{325}N_{41}O_{80}P_6$: 1143.77568, found 1143.77633.

Ac-[Lys(butan-4-(6-deoxy-*6*-phosphonomethyl-α-D-mannopyranosyl)-amide)]$_6$-Ile-Ser-Gln-Ala-Val-His-Ala-Ala-His-Ala-Glu-Ile-Asn-Glu-Ala-Gly-Arg-Lys(4-((6-amino-2-butoxy-8-oxo-7,8-dihydro-9H-purin-9-yl)methyl)-N-(2-(2-(2-amino-2-oxoethoxy)ethoxy)ethyl)benzamide)-NH$_2$ (6)

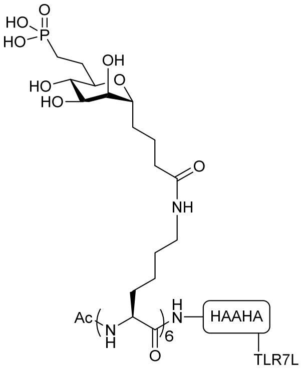

40 µmol of crude Ac-[Lys(butan-4-(6-deoxy-2,3-*O*-isopropylidene-4-*O*-*p*-methoxybenzyl-*6*-di-*tert*-butoxyphosphonomethyl-α-D-mannopyranosyl)-amide)]$_6$-Ile-Ser(*t*Bu)-Gln(Trt)-Ala-Val-His(Trt)-Ala-Ala-His(Trt)-Ala-Glu(O*t*Bu)-Ile-Asn(Trt)-Glu(O*t*Bu)-Ala-Gly-Arg(Pbf)-Lys(MMT)-Tentagel(SRam) was treated with a continuous flow of a mixture of AcOH/TFE/DCM (1/2/7 v/v/v, 30 mL) over 15 minutes. The resin was washed subsequently with DCM (5x), AcOH/TFE/DCM (1/2/7 v/v/v, 4 mL), DCM (5x), 1 M DIPEA in NMP (2x 3 mL), DCM (3x) and DMF (3x). A solution of {2-[2-(Fmoc-amino)ethoxy]ethoxy}acetic acid (34 mg, 88 µmol, 2.2 eq.) and HCTU (34 mg, 82 µmol, 2.1 eq.) in DMF (0.8 mL) and DIPEA (28 µL, 0.16 mmol, 4.0 eq.) were added and the suspension was shaken overnight. The resin was washed with DMF (5x), treated with 20% piperidine in DMF (0.8 mL, 2x 20 min) and washed with DMF (5x). A solution of 4-((2-butoxy-6-((*tert*-butoxycarbonyl)amino)-8-oxo-7,8-dihydro-9H-purin-9-yl)methyl)benzoid acid (38 mg, 83 µmol, 2.1 eq.) and HCTU (34 mg, 82 µmol, 2.1 eq.) and DIPEA (28 µL, 0.16 mmol, 4.0 eq.) were added and the suspension was shaken overnight. The resin was washed with DMF (3x), DCM (3x) and the peptide was cleaved from the resin after treatment with TFA/TIS/H_2O (95/2.5/2.5 v/v/v) (1.6 mL) for 3 hours the suspension was filtered and the residue was washed with the cleavage cocktail (1.6 mL). The product was precipitated with Et_2O. After purification by RP-HPLC and lyophilisation, compound **6** (3.1 mg, 0.61 µmol, 2%) was obtained as a white solid. LC-MS: Rt = 5.73 min (C18 Gemini, 10 - 50% MeCN, 11 min run); ESI-MS (m/z) found 1686.9 $[M+3H]^{3+}$; HRMS: $[M+4H]^{4+}$ calcd. for $C_{207}H_{353}N_{47}O_{86}P_6$: 1264.82744, found 1264.82866.

Ac-Ile-Ser-Gln-Ala-Val-His-Ala-Ala-His-Ala-Glu-Ile-Asn-Glu-Ala-Gly-Arg-Lys-Lys(butan-4-(6-deoxy-*6*-phosphonomethyl-α-D-mannopyranosyl)-amide)]$_6$-NH$_2$ (7)

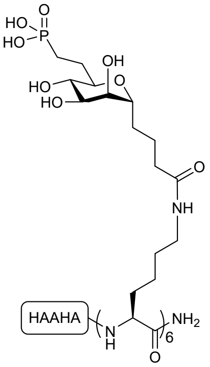

100 µmol of Tentagel S Ram was washed with DMF (5x) and elongated by the addition of a solution of acid **10** (0.19 g, 0.20 mmol, 2.0 eq.) and HCTU (84 mg, 0.20 mmol, 2.0 eq.) in DMF (2.0 mL) and DIPEA (70 µL, 0.40 mmol, 4.0 eq.). The suspension was shaken overnight. The resin was washed with DMF (5x), treated with 20% piperidine in DMF (2.0 mL, 2x 20 min) and washed with DMF (5x). This was repeated another 5 times, after which the resin was elongated using the synthesizer with H-Ile-Ser(*t*Bu)-Gln(Trt)-Ala-Val-His(Trt)-Ala-Ala-His(Trt)-Ala-Glu(O*t*Bu)-Ile-Asn(Trt)-Glu(O*t*Bu)-Ala-Gly-Arg(Pbf)-Lys(MMT) with standard HCTU/Fmoc chemistry concluding in final Fmoc removal with a solution of 20% piperidine in DMF (3x 3 min). 30 µmol of crude H-Ile-Ser(*t*Bu)-Gln(Trt)-Ala-Val-His(Trt)-Ala-Ala-His(Trt)-Ala-Glu(O*t*Bu)-Ile-Asn(Trt)-Glu(O*t*Bu)-Ala-Gly-Arg(Pbf)-Lys(MMT)-[Lys(butan-4-(6-deoxy-2,3-*O*-isopropylidene-4-*O*-*p*-methoxybenzyl-*6*-di-*tert*-butoxyphosphonomethyl-*α*-D-mannopyranosyl)-amide)]$_6$-Tentagel(SRam) was treated with a mixture of Ac_2O/DMF/DIPEA (2x 1.2 mL, 20 min), and washed with DMF (3x) and DCM (3x). The peptide was cleaved from the resin after treatment with TFA/TIS/H_2O (95/2.5/2.5 v/v/v) (1.2 mL) for 3 hours the suspension was filtered and the residue was washed with the cleavage cocktail (1.2 mL). The product was precipitated with Et_2O. After purification by RP-HPLC and lyophilisation, compound **7** (11.0 mg, 2.4 µmol, 8%) was obtained as a white solid. LC-MS: Rt = 6.00 min (C18 Gemini, 0 - 50% MeCN, 11 min run); ESI-MS (m/z) found 1525.6 $[M+3H]^{3+}$; HRMS: $[M+4H]^{4+}$ calcd. for $C_{184}H_{325}N_{41}O_{80}P_6$: 1143.77568, found 1143.77518.

(4-((6-amino-2-butoxy-8-oxo-7,8-dihydro-9H-purin-9-yl)methyl)-N-(2-(2-(2-amino-2-oxoethoxy)ethoxy)ethyl)benzamide)-Ile-Ser-Gln-Ala-Val-His-Ala-Ala-His-Ala-Glu-Ile-Asn-Glu-Ala-Gly-Arg-Lys-[Lys(butan-4-(6-deoxy-*6*-phosphonomethyl-α-D-mannopyranosyl)-amide)]$_6$-NH$_2$ (8)

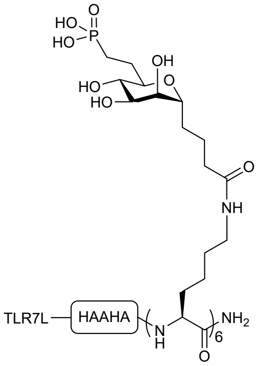

30 µmol of crude H-Ile-Ser(*t*Bu)-Gln(Trt)-Ala-Val-His(Trt)-Ala-Ala-His(Trt)-Ala-Glu(O*t*Bu)-Ile-Asn(Trt)-Glu(O*t*Bu)-Ala-Gly-Arg(Pbf)-Lys(MMT)-[Lys(butan-4-(6-deoxy-2,3-*O*-isopropylidene-4-*O*-*p*-methoxybenzyl-*6*-di-*tert*-butoxyphosphonomethyl-α-D-mannopyranosyl)-amide)]$_6$-Tentagel(SRam) was treated with a solution of {2-[2-(Fmoc-amino)ethoxy]ethoxy}acetic acid (27 mg, 70 µmol, 2.3 eq.) and HCTU (25 mg, 60 µmol, 2.0 eq.) in DMF (0.6 mL) and DIPEA (21 µL, 0.12 mmol, 4.0 eq.) were added and the suspension was shaken overnight. The resin was washed with DMF (5x), treated with 20% piperidine in DMF (0.6 mL, 2x 20 min) and washed with DMF (5x). A solution of 4-((2-butoxy-6-((*tert*-butoxycarbonyl)amino)-8-oxo-7,8-dihydro-9H-purin-9-yl)methyl)benzoid acid (29 mg, 63 µmol, 2.1 eq.) and HCTU (25 mg, 60 µmol, 2.0 eq.) and DIPEA (21 µL, 0.12 mmol, 4.0 eq.) were added and the suspension was shaken overnight. The resin was washed with DMF (3x), DCM (3x) and the peptide was cleaved from the resin after treatment with TFA/TIS/H_2O (95/2.5/2.5 v/v/v) (1.2 mL) for 3 hours the suspension was filtered and the residue was washed with the cleavage cocktail (1.2 mL). The product was precipitated with Et_2O. After purification by RP-HPLC and lyophilisation, compound **8** (17.0 mg, 3.4 µmol, 11%) was obtained as a white solid. LC-MS: Rt = 5.91 min (C18 Gemini, 10 - 50% MeCN, 11 min run); ESI-MS (m/z) found 1673.1 $[M+3H]^{3+}$; HRMS: $[M+4H]^{4+}$ calcd. for $C_{205}H_{351}N_{47}O_{85}P_6$: 1254.32479, found 1254.32528.

H-Asp-Glu-Val-Ser-Gly-Leu-Glu-Gln-Leu-Glu-Ser-Ile-Ile-Asn-Phe-Glu-Lys-Leu-Ala-Ala-Ala-Ala-Ala-Lys-NH$_2$ (35)

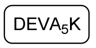

Tentagel S Ram resin loaded with H-Asp(O*t*Bu)-Glu(O*t*Bu)-Val-Ser(*t*Bu)-Gly-Leu-Glu(O*t*Bu)-Gln(Trt)-Leu-Glu(O*t*Bu)-Ser(*t*Bu)-Ile-Ile-Asn(Trt)-Phe-Glu(O*t*Bu)-Lys(Boc)-Leu-Ala-Ala-Ala-Ala-Ala-Lys(MMT) on 30 µmol scale was washed with DCM (5x). The peptide was cleaved from the resin after treatment with TFA/TIS/H_2O (95/2/2/1 v/v/v/v) (1.2 mL) for 3 hours the suspension was filtered and the residue was washed with the cleavage cocktail (1.2 mL). The product was precipitated with Et_2O. After purification by RP-HPLC and lyophilisation, compound **35** (12 mg, 4.7 µmol, 16%) was obtained as a white solid. LC-MS: Rt = 4.82 min (C18 Gemini, 10 - 90% MeCN, 11 min run); ESI-MS (m/z) found 1273.7 $[M+2H]^{2+}$; HRMS: $[M+2H]^{2+}$ calcd. for $C_{112}H_{187}N_{29}O_{38}$: 1273.17904, found 1273.17779.

(4-((6-amino-2-butoxy-8-oxo-7,8-dihydro-9H-purin-9-yl)methyl)-N-(2-(2-(2-amino-2-oxoethoxy)ethoxy)ethyl)benzamide)-Asp-Glu-Val-Ser-Gly-Leu-Glu-Gln-Leu-Glu-Ser-Ile-Ile-Asn-Phe-Glu-Lys-Leu-Ala-Ala-Ala-Ala-Ala-Lys-Lys(N$_3$)-Lys(N$_3$)-Lys(N$_3$)-Lys(N$_3$)-Lys(N$_3$)-Lys(N$_3$)-NH$_2$ (36)

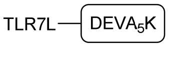

30 µmol of crude H-Lys(N_3)-Lys(N_3)-Lys(N_3)-Lys(N_3)-Lys(N_3)-Lys(N_3)-Asp(O*t*Bu)-Glu(O*t*Bu)-Val-Ser(*t*Bu)-Gly-Leu-Glu(O*t*Bu)-Gln(Trt)-Leu-Glu(O*t*Bu)-Ser(*t*Bu)-Ile-Ile-Asn(Trt)-Phe-Glu(O*t*Bu)-

Lys(Boc)-Leu-Ala-Ala-Ala-Ala-Ala-Lys(MMT)-Tentagel(SRam) was washed with DMF (5x), followed by the addition of a solution of {2-[2-(Fmoc-amino)ethoxy]ethoxy}acetic acid (25 mg, 65 µmol, 2.2 eq.) and HCTU (25 mg, 60 µmol, 2.0 eq.) in DMF (0.6 mL) and DIPEA (21 µL, 121 µmol, 4.0 eq.). The suspension was shaken overnight. The resin was washed with DMF (5x), treated with 20% piperidine in DMF (0.6 mL, 2x 20 min) and washed with DMF (5x). A solution of 4-((2-butoxy-6-((*tert*-butoxycarbonyl)amino)-8-oxo-7,8-dihydro-9H-purin-9-yl)methyl)benzoid acid (28 mg, 61 µmol, 2.0 eq.) and HCTU (25 mg, 60 µmol, 2.0 eq.) and DIPEA (21 µL, 121 µmol, 4.0 eq.) were added and the suspension was shaken overnight. The peptide was cleaved from the resin after treatment with TFA/TIS/H_2O (95/2.5/2.5 v/v/v) (1.2 mL) for 3 hours the suspension was filtered and the residue was washed with the cleavage cocktail (1.2 mL). The product was precipitated with Et_2O. After purification by RP-HPLC and lyophilisation, compound **36** (4.1 mg, 1.4 µmol, 5%) was obtained as a white solid. LC-MS: Rt = 5.34 min (C18 Gemini, 10 - 90% MeCN, 11 min run); ESI-MS (m/z) found 1516.0 $[M+2H]^{2+}$; HRMS: $[M+2H]^{2+}$ calcd. for $C_{135}H_{215}N_{35}O_{44}$: 1515.78424, found 1515.78242.

Ac-Asp-Glu-Val-Ser-Gly-Leu-Glu-Gln-Leu-Glu-Ser-Ile-Ile-Asn-Phe-Glu-Lys-Leu-Ala-Ala-Ala-Ala-Ala-Lys(4-((6-amino-2-butoxy-8-oxo-7,8-dihydro-9H-purin-9-yl)methyl)-N-(2-(2-(2-amino-2-oxoethoxy)ethoxy)ethyl)benzamide)-NH_2 (37)

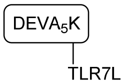

30 µmol of crude H-Asp(O*t*Bu)-Glu(O*t*Bu)-Val-Ser(*t*Bu)-Gly-Leu-Glu(O*t*Bu)-Gln(Trt)-Leu-Glu(O*t*Bu)-Ser(*t*Bu)-Ile-Ile-Asn(Trt)-Phe-Glu(O*t*Bu)-Lys(Boc)-Leu-Ala-Ala-Ala-Ala-Ala-Lys(MMT)-Tentagel(SRam) was was treated with a mixture of Ac_2O/DMF/DIPEA (2x 0.6 mL, 20 min), and washed with DMF (3x) and DCM (3x). The peptide was treated with a continuous flow of a mixture of TFA/TIS/DCM (96/2/2 v/v/v, 11 mL) over 15 minutes. The resin was washed subsequently with DCM (5x), TFA/TIS/DCM (96/2/2 v/v/v, 2 mL), DCM (5x), 1 M DIPEA in NMP (2 mL), DCM (3x) and DMF (3x). A solution of {2-[2-(Fmoc-amino)ethoxy]ethoxy}acetic acid (24 mg, 62 µmol, 2.1 eq.) and HCTU (25 mg, 60 µmol, 2.0 eq.) in DMF (0.6 mL) and DIPEA (21 µL, 121 µmol, 4.0 eq.) were added and the suspension was shaken overnight. The resin was washed with DMF (5x), treated with 20% piperidine in DMF (0.6 mL, 2x 20 min) and washed with DMF (5x). A solution of 4-((2-butoxy-6-((*tert*-butoxycarbonyl)amino)-8-oxo-7,8-dihydro-9H-purin-9-yl)methyl)benzoid acid (28 mg, 61 µmol, 2.0 eq.) and HCTU (26 mg, 62 µmol, 2.1 eq.) and DIPEA (21 µL, 121 µmol, 4.0 eq.) were added and the suspension was shaken overnight. The peptide was cleaved from the resin after treatment with TFA/TIS/H_2O (95/2.5/2.5 v/v/v) (1.2 mL) for 3 hours the suspension was filtered and the residue was washed with the cleavage cocktail (1.2 mL). The product was precipitated with Et_2O. After purification by RP-HPLC and lyophilisation, compound **37** (9.4 mg, 3.1 µmol, 10%) was obtained as a white solid. LC-MS: Rt = 5.41 min (C18 Gemini, 10 - 90% MeCN, 11 min run); ESI-MS (m/z) found 1536.9 $[M+2H]^{2+}$; HRMS: $[M+2H]^{2+}$ calcd. for $C_{137}H_{217}N_{35}O_{45}$: 1536.28784, found 1536.28769.

H-Ile-Ser-Gln-Ala-Val-His-Ala-Ala-His-Ala-Glu-Ile-Asn-Glu-Ala-Gly-Arg-Lys-NH_2 (38)

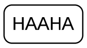

Tentagel S Ram resin loaded with H-Ile-Ser(*t*Bu)-Gln(Trt)-Ala-Val-His(Trt)-Ala-Ala-His(Trt)-Ala-Glu(O*t*Bu)-Ile-Asn(Trt)-Glu(O*t*Bu)-Ala-Gly-Arg(Pbf)-Lys(MMT)-NH_2 on 30 µmol scale was washed with DCM (5x). The peptide was cleaved from the resin after treatment with TFA/TIS/H_2O (95/2/2/1 v/v/v/v) (1.2 mL) for 3 hours the suspension was filtered and the residue was washed with the cleavage cocktail (1.2 mL). The product was precipitated with Et_2O. After purification by RP-HPLC and lyophilisation, compound **38** (16.8 mg, 8.8 µmol, 30%) was obtained as a white solid. LC-MS: Rt = 3.70 min (C18 Gemini, 10 - 50% MeCN, 11 min run); ESI-MS (m/z) found 1901.0 $[M+H]^{+}$; HRMS: $[M+2H]^{2+}$ calcd. for $C_{80}H_{135}N_{29}O_{25}$: 951.00865, found 951.00848.

(4-((6-amino-2-butoxy-8-oxo-7,8-dihydro-9H-purin-9-yl)methyl)-N-(2-(2-(2-amino-2-oxoethoxy)ethoxy)ethyl)benzamide)-Ile-Ser-Gln-Ala-Val-His-Ala-Ala-His-Ala-Glu-Ile-Asn-Glu-Ala-Gly-Arg-Lys-NH_2 (39)

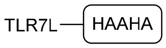

30 µmol of crude H-Ile-Ser(*t*Bu)-Gln(Trt)-Ala-Val-His(Trt)-Ala-Ala-His(Trt)-Ala-Glu(O*t*Bu)-Ile-Asn(Trt)-Glu(O*t*Bu)-Ala-Gly-Arg(Pbf)-Lys(MMT)-Tentagel(SRam) was washed with DMF (5x), followed by the addition of a solution of {2-[2-(Fmoc-amino)ethoxy]ethoxy}acetic acid (24 mg, 62 µmol, 2.1 eq.) and HCTU (25 mg, 60 µmol, 2.0 eq.) in DMF (0.6 mL) and DIPEA (21 µL, 121 µmol, 4.0 eq.). The suspension was shaken overnight. The resin was washed with DMF (5x), treated with 20% piperidine in DMF (0.6 mL, 2x 20 min) and washed with DMF (5x). A solution of 4-((2-butoxy-6-((*tert*-butoxycarbonyl)amino)-8-oxo-7,8-dihydro-9H-purin-9-yl)methyl)benzoid acid (28 mg, 61 µmol, 2.0 eq.) and HCTU (26 mg, 60 µmol, 2.0 eq.) and DIPEA (21 µL, 121 µmol, 4.0 eq.) were added and the suspension was shaken overnight. The peptide was cleaved from the resin after treatment with TFA/TIS/H_2O (95/2.5/2.5 v/v/v) (1.2 mL) for 3 hours the suspension was filtered and the residue was washed with the cleavage cocktail (1.2 mL). The product was precipitated with Et_2O. After purification by RP-HPLC and lyophilisation, compound **39** (10.3 mg, 4.3 µmol, 14%) was obtained as a white solid.

LC-MS: Rt = 6.17 min (C18 Gemini, 10 - 50% MeCN, 11 min run); ESI-MS (m/z) found 1193.7 $[M+2H]^{2+}$; HRMS: $[M+2H]^{2+}$ calcd. for $C_{103}H_{163}N_{65}O_{31}$: 1193.11216, found 1193.11176.

Ac-Ile-Ser-Gln-Ala-Val-His-Ala-Ala-His-Ala-Glu-Ile-Asn-Glu-Ala-Gly-Arg-Lys(4-((6-amino-2-butoxy-8-oxo-7,8-dihydro-9H-purin-9-yl)methyl)-N-(2-(2-(2-amino-2-oxoethoxy)ethoxy)ethyl)benzamide)-NH_2 (40)

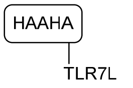

30 µmol of crude H-Ile-Ser(*t*Bu)-Gln(Trt)-Ala-Val-His(Trt)-Ala-Ala-His(Trt)-Ala-Glu(O*t*Bu)-Ile-Asn(Trt)-Glu(O*t*Bu)-Ala-Gly-Arg(Pbf)-Lys(MMT)-Tentagel(SRam) was was treated with a mixture of Ac_2O/DMF/DIPEA (2x 0.6 mL, 20 min), and washed with DMF (3x) and DCM (3x). The peptide was treated with a continuous flow of a mixture of TFA/TIS/DCM (96/2/2 v/v/v, 11 mL) over 15 minutes. The resin was washed subsequently with DCM (5x), TFA/TIS/DCM (96/2/2 v/v/v, 2 mL), DCM (5x), 1 M DIPEA in NMP (2 mL), DCM (3x) and DMF (3x). A solution of {2-[2-(Fmoc-amino)ethoxy]ethoxy}acetic acid (24 mg, 62 µmol, 2.1 eq.) and HCTU (25 mg, 60 µmol, 2.0 eq.) in DMF (0.6 mL) and DIPEA (21 µL, 121 µmol, 4.0 eq.) were added and the suspension was shaken overnight. The resin was washed with DMF (5x), treated with 20% piperidine in DMF (0.6 mL, 2x 20 min) and washed with DMF (5x). A solution of 4-((2-butoxy-6-((*tert*-butoxycarbonyl)amino)-8-oxo-7,8-dihydro-9H-purin-9-yl)methyl)benzoid acid (28 mg, 61 µmol, 2.0 eq.) and HCTU (26 mg, 62 µmol, 2.1 eq.) and DIPEA (21 µL, 121 µmol, 4.0 eq.) were added and the suspension was shaken overnight. The peptide was cleaved from the resin after treatment with TFA/TIS/H_2O (95/2.5/2.5 v/v/v) (1.2 mL) for 3 hours the suspension was filtered and the residue was washed with the cleavage cocktail (1.2 mL). The product was precipitated with Et_2O. After purification by RP-HPLC and lyophilisation, compound **40** (7.6 mg, 3.1 µmol, 10%) was obtained as a white solid. LC-MS: Rt = 6.47 min (C18 Gemini, 10 - 50% MeCN, 11 min run); ESI-MS (m/z) found 1214.3 $[M+2H]^{2+}$; HRMS: $[M+2H]^{2+}$ calcd. for $C_{105}H_{165}N_{35}O_{32}$: 1214.11745, found 1214.11682.

Ac-[Lys(butan-4-(6-deoxy-*6*-phosphonomethyl-α-D-mannopyranosyl)-amide)]$_6$-Asp-Glu-Val-Ser-Gly-Leu-Glu-Gln-Leu-Glu-Ser-Ile-Ile-Asn-Phe-Glu-Lys-Leu-Ala-Ala-Ala-Ala-Ala-Lys-NH_2 (S9)

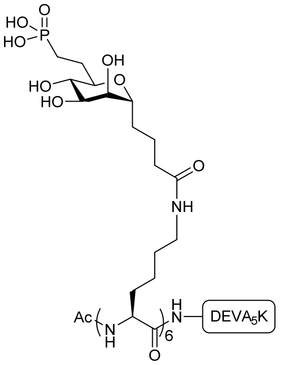

100 µmol of crude H-Asp(OtBu)-Glu(OtBu)-Val-Ser(tBu)-Gly-Leu-Glu(OtBu)-Gln(Trt)-Leu-Glu(OtBu)-Ser(tBu)-Ile-Ile-Asn(Trt)-Phe-Glu(OtBu)-Lys(Boc)-Leu-Ala-Ala-Ala-Ala-Ala-Lys(MMT)-Tentagel(SRam) was washed with DMF (5x) and elongated by the addition of a solution of acid **10** (0.19 g, 0.20 mmol, 2.0 eq.) and HCTU (84 mg, 0.20 µmol, 2.0 eq.) in DMF (2.0 mL) and DIPEA (70 µL, 0.40 mmol, 4.0 eq.). The suspension was shaken overnight. The resin was washed with DMF (5x), treated with 20% piperidine in DMF (2.0 mL, 2x 20 min) and washed with DMF (5x). This was repeated another 5 times. 40 µmol of crude H-[Lys(butan-4-(6-deoxy-2,3-*O*-isopropylidene-4-*O*-*p*-methoxybenzyl-*6*-di-*tert*-butoxyphosphonomethyl-α-D-mannopyranosyl)-amide)]$_6$-Asp(OtBu)-Glu(OtBu)-Val-Ser(tBu)-Gly-Leu-Glu(OtBu)-Gln(Trt)-Leu-Glu(OtBu)-Ser(tBu)-Ile-Ile-Asn(Trt)-Phe-Glu(OtBu)-Lys(Boc)-Leu-Ala-Ala-Ala-Ala-Ala-Lys(MMT)-Tentagel(SRam) was treated with a mixture of Ac_2O/DMF/DIPEA (2x 0.8 mL, 20 min), and washed with DMF (3x) and DCM (3x). The peptide was cleaved from the resin after treatment with TFA/TIS/H_2O (95/2.5/2.5 v/v/v) (1.6 mL) for 3 hours the suspension was filtered and the residue was washed with the cleavage cocktail (1.6 mL). The product was precipitated with Et_2O. After purification by RP-HPLC and lyophilisation, compound **43** (8.2 mg, 1.6 µmol, 4%) was obtained as a white solid. LC-MS: Rt = 4.74 min (C18 Gemini, 10 - 90% MeCN, 11 min run); ESI-MS (m/z) found 1740.1 $[M+3H]^{3+}$; HRMS: $[M+3H]^{3+}$ calcd. for $C_{216}H_{376}N_{41}O_{93}P_6$: 1739.47874, found 1739.47817.

Supplemental references

[1] N. Reintjens, T. Koemans, N. Zilverschoon, R. Castelli, R. Cordfunke, J.-W. Drijfhout, N. Meeuwenoord, H. Overkleeft, D. Filippov, G. Van der Marel and J. Codée, *European J. Org. Chem.* **2020**, DOI 10.1002/ejoc.202000587.

LCMS spectra

LC-MS of compound (**26**)

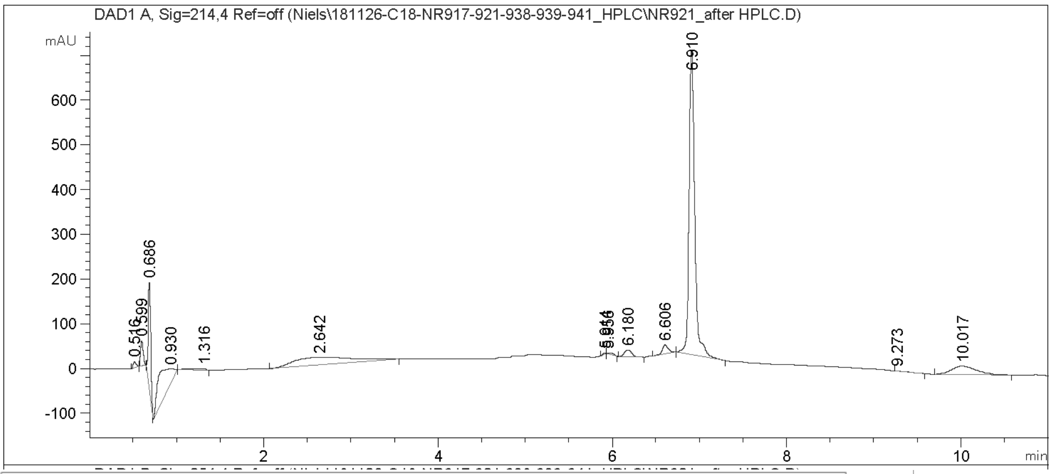

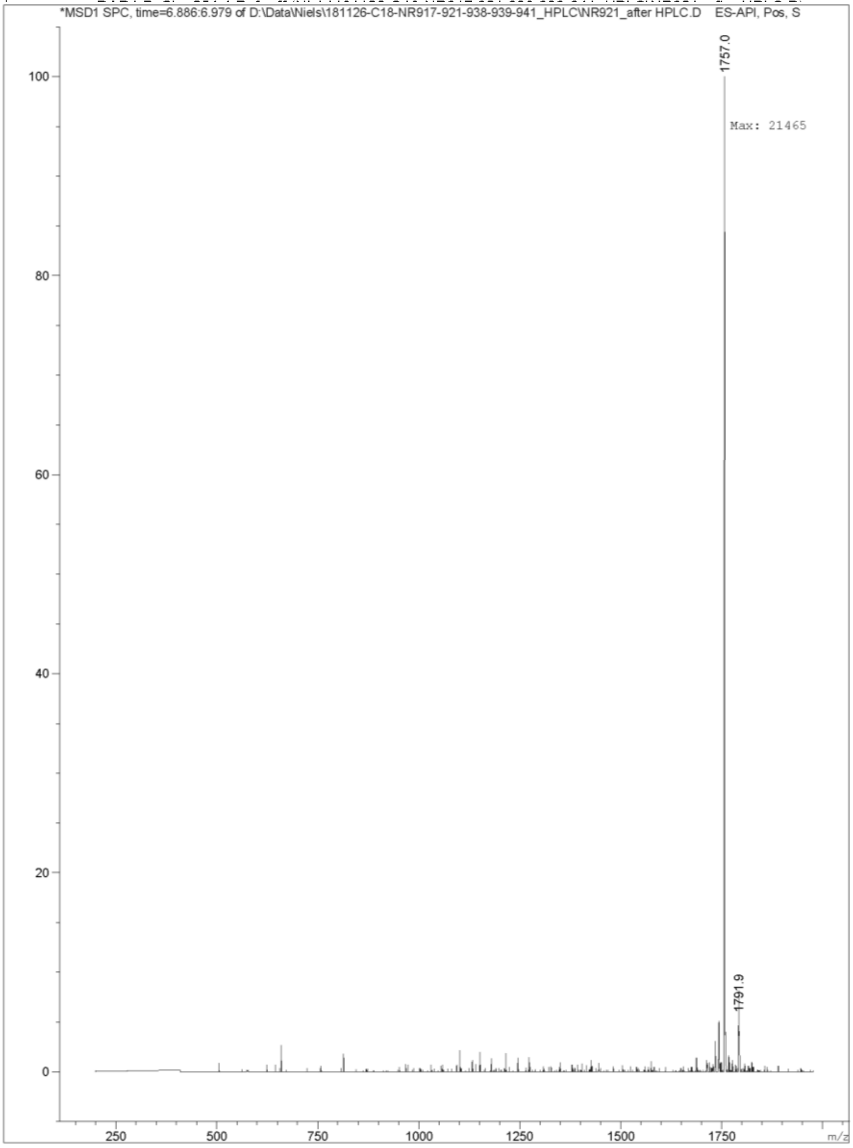

LC-MS of compound (**27**)

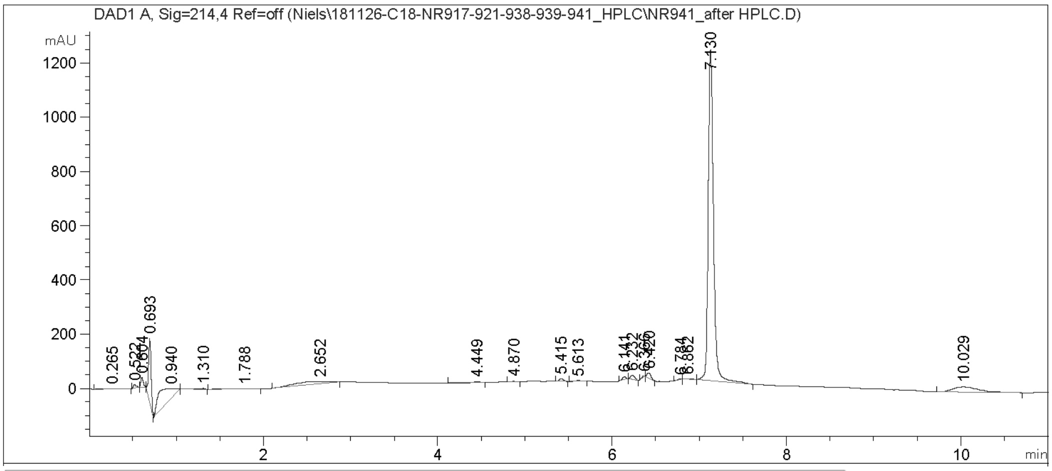

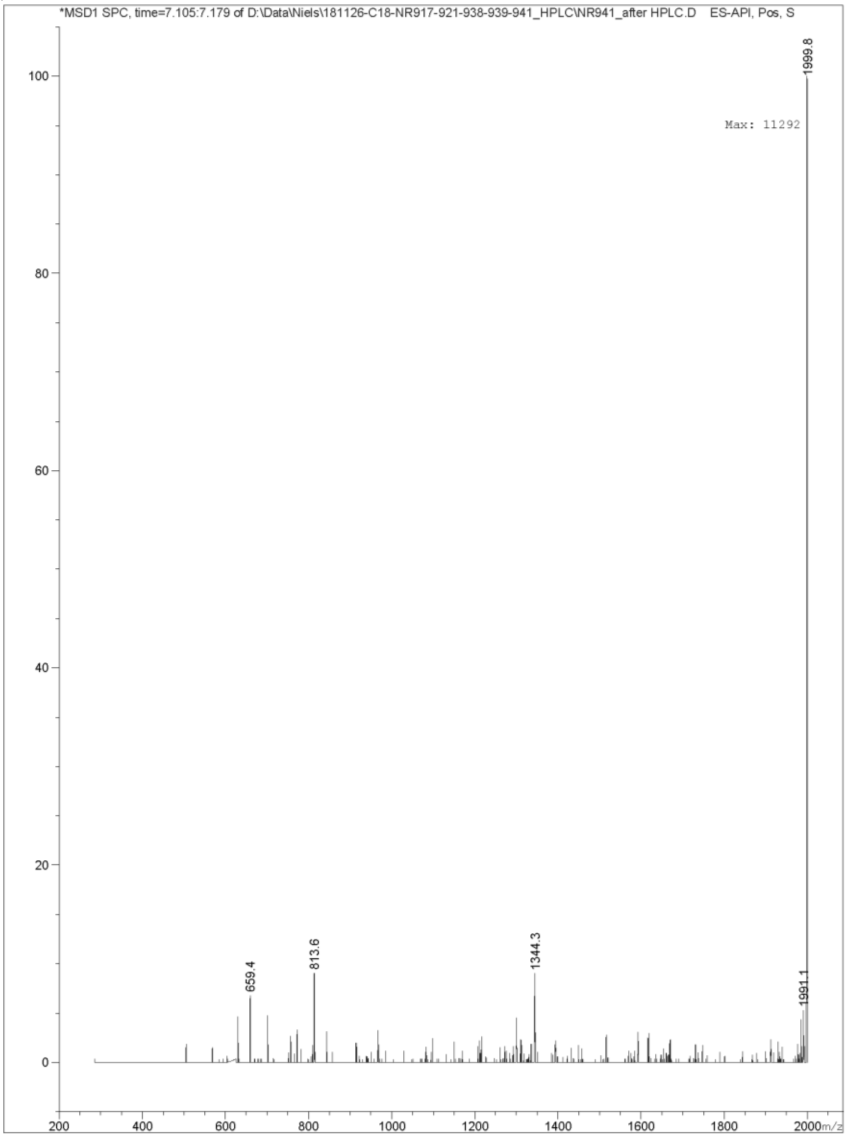

LC-MS of compound (**29**)

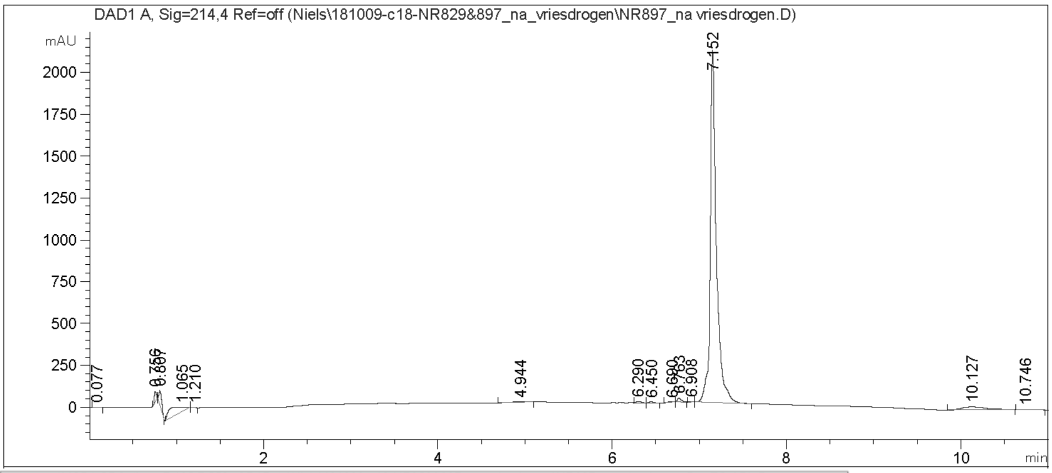

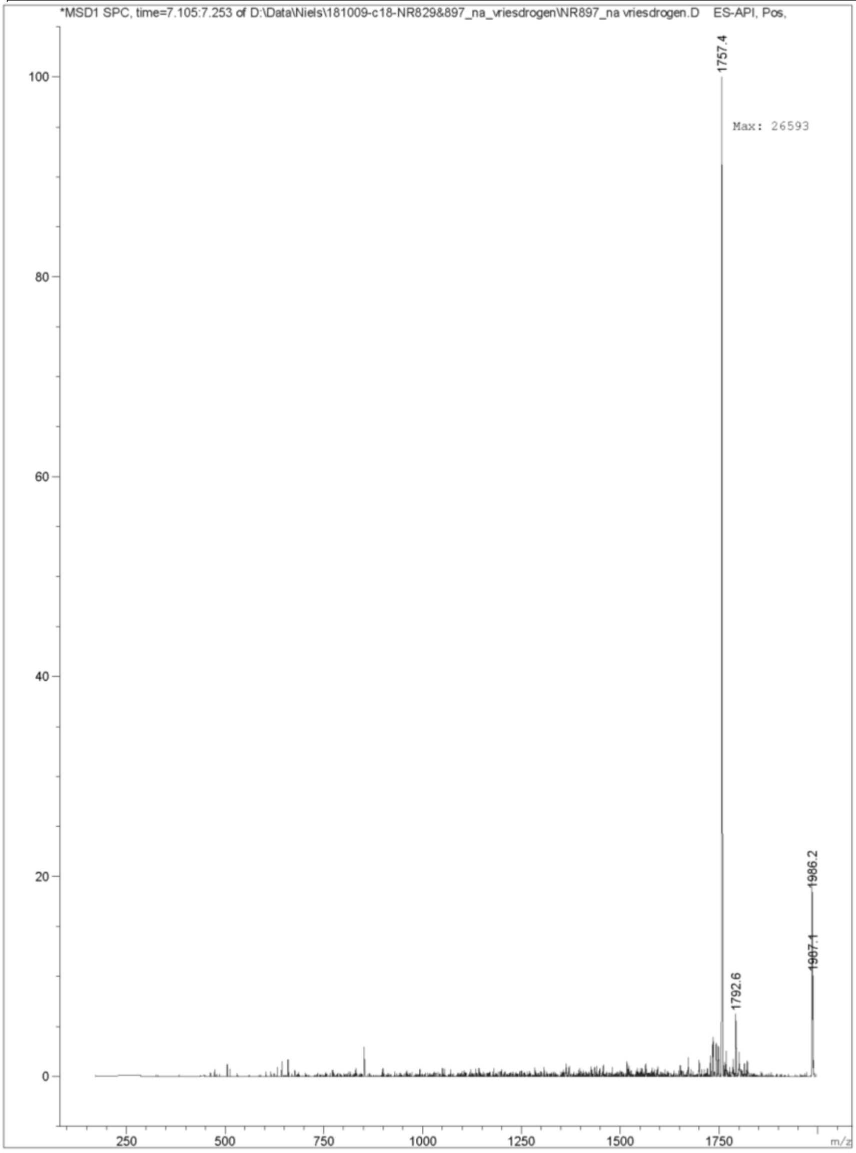

LC-MS of compound (**30**)

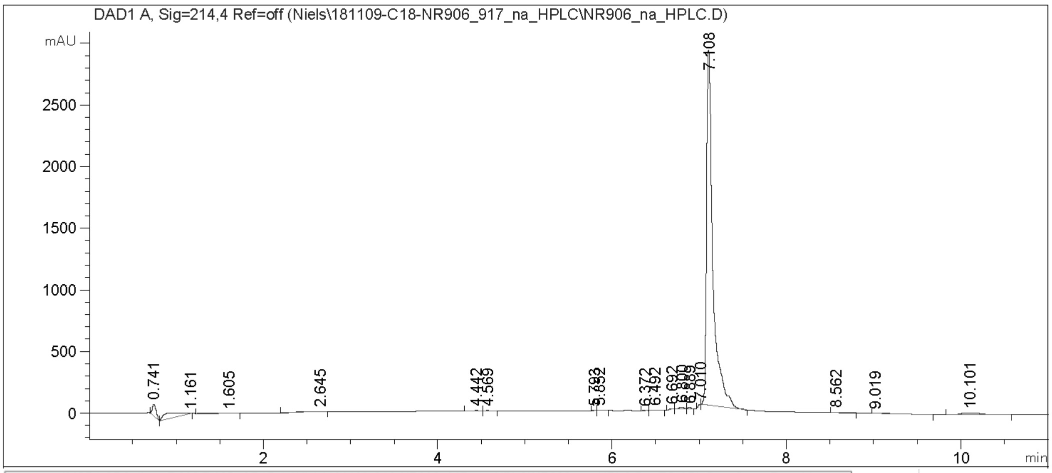

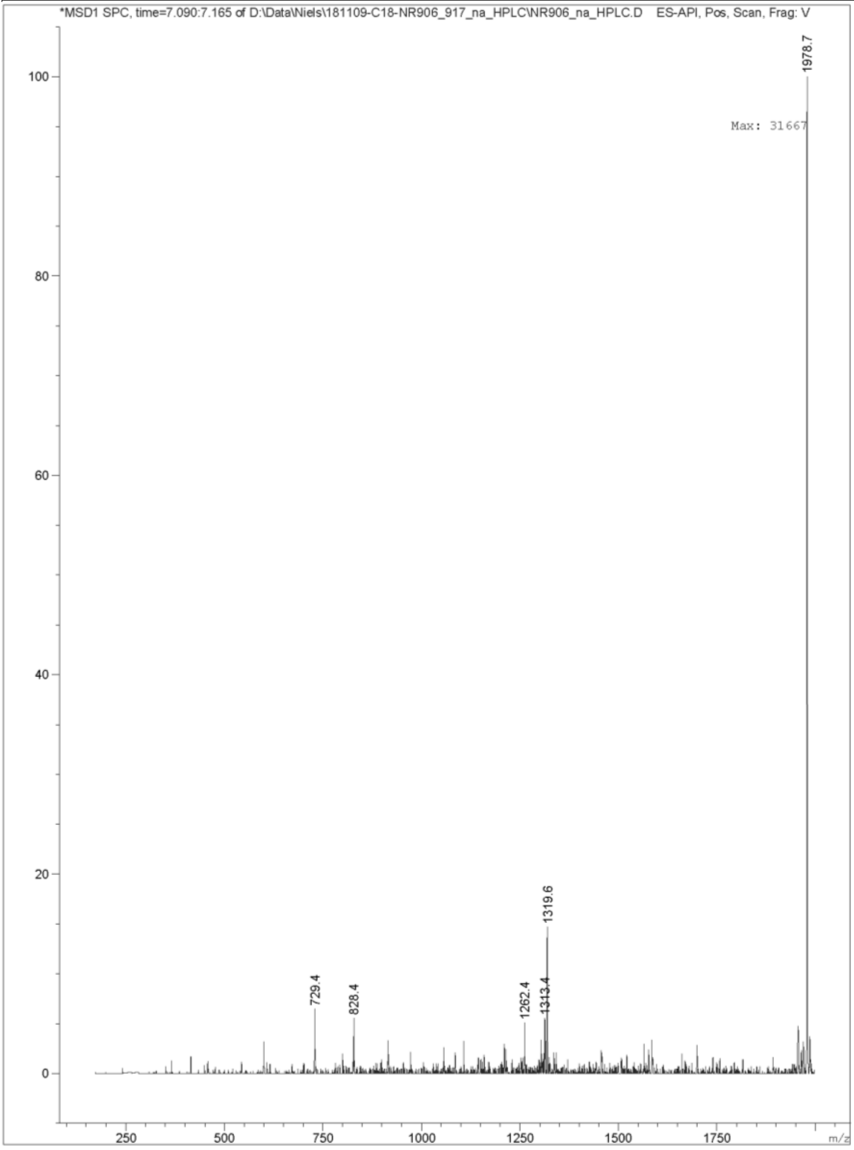

LC-MS of conjugate (**1**)

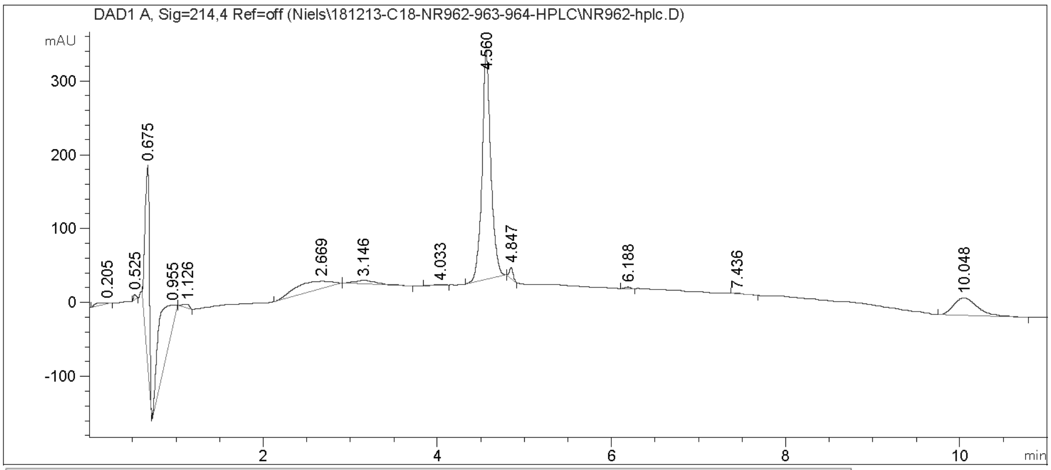

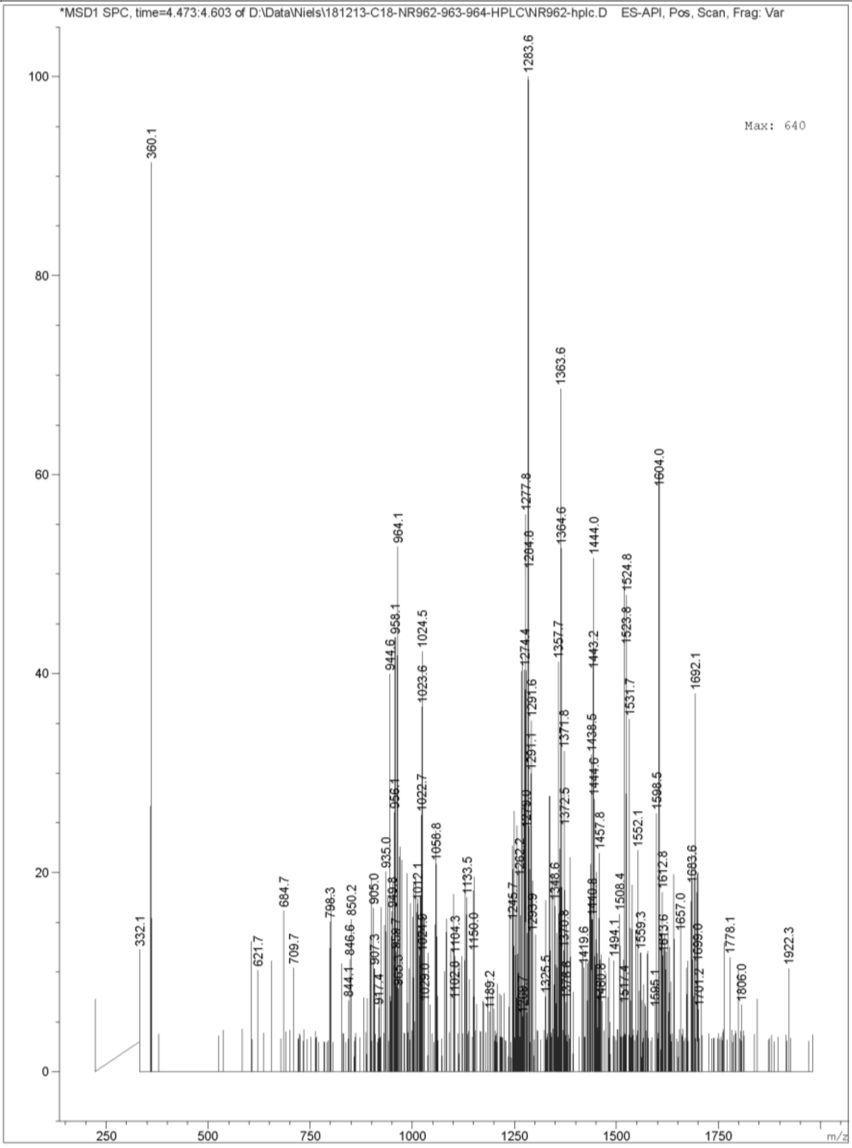

*Fragmentation occurs during MS, resulting in deglycosylated mass peaks.

LC-MS of conjugate (**2**)

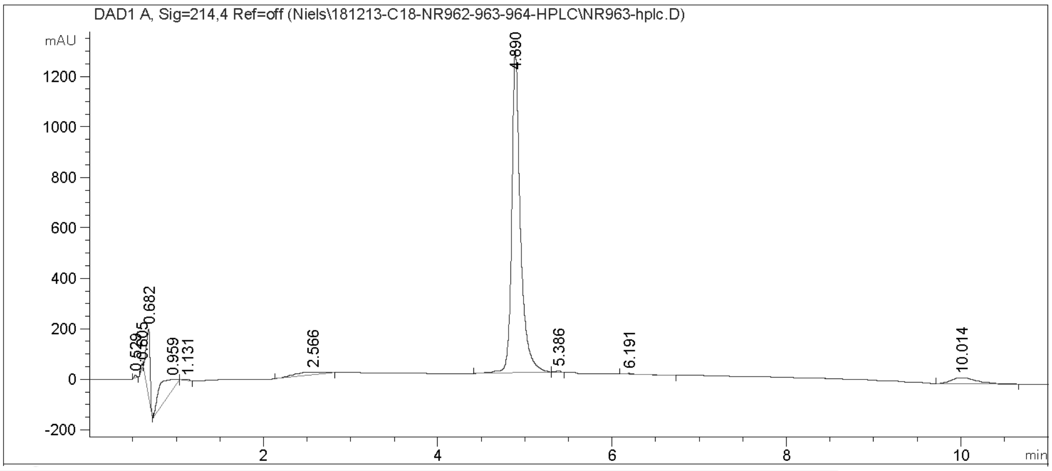

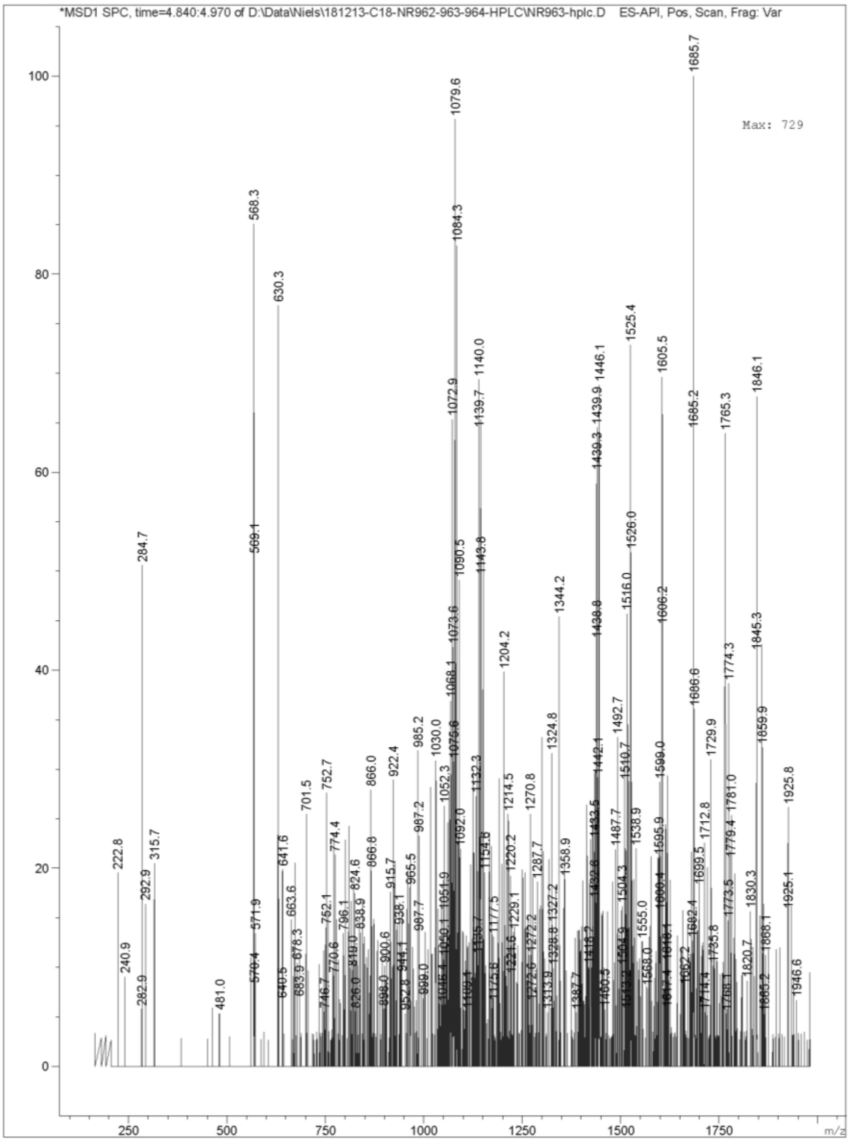

*Fragmentation occurs during MS, resulting in deglycosylated mass peaks.

LC-MS of conjugate (**3**)

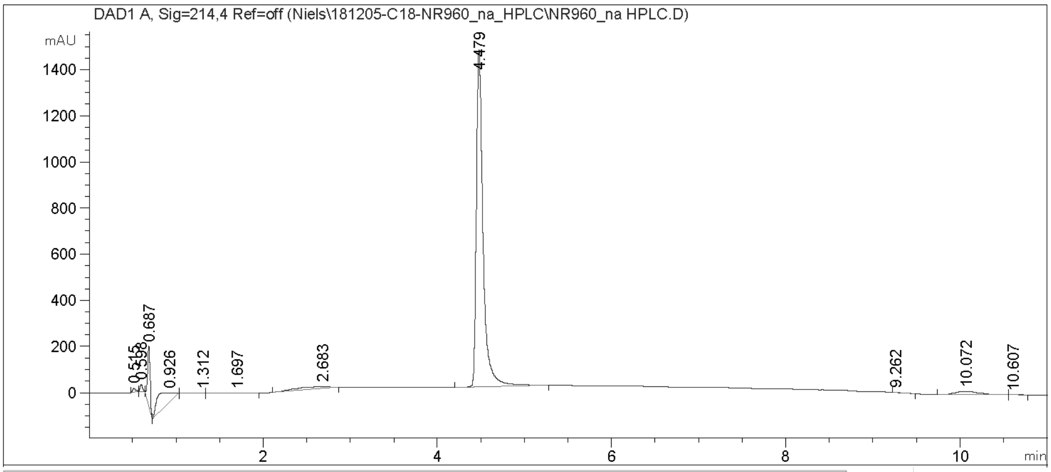

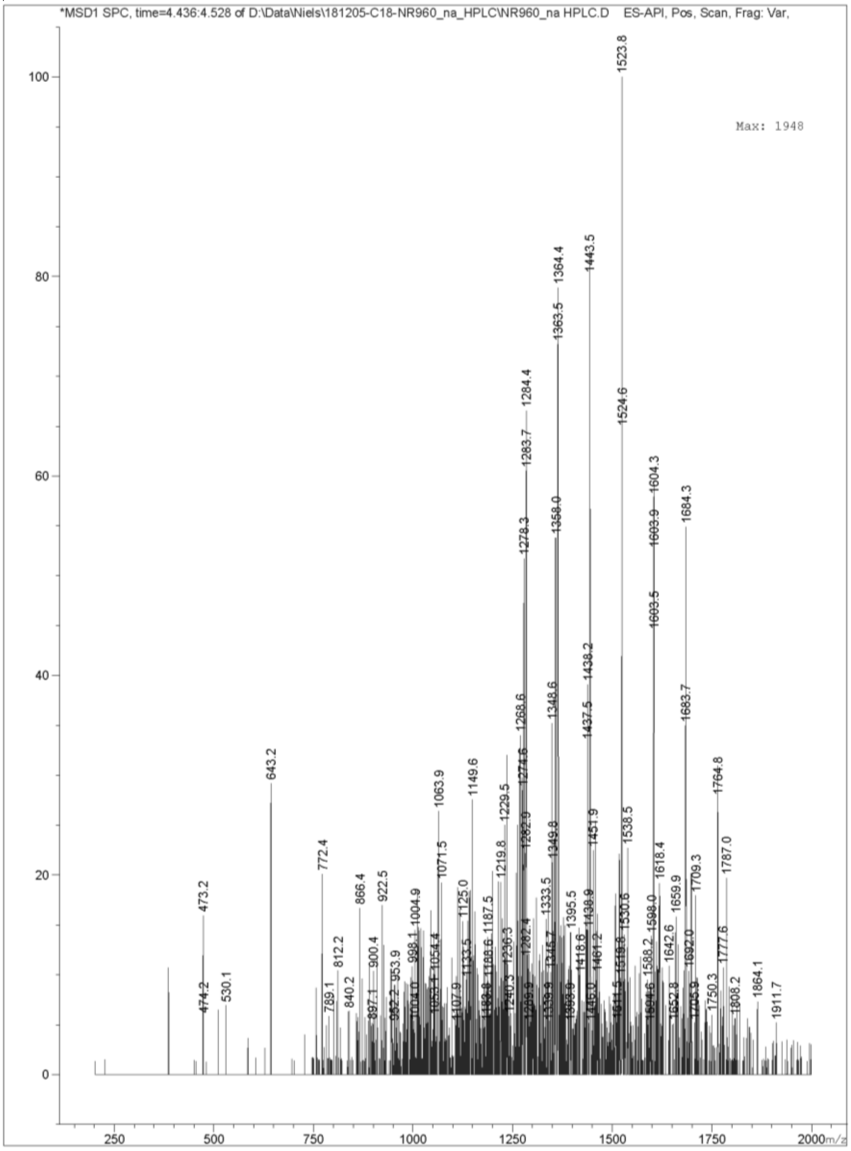

*Fragmentation occurs during MS, resulting in deglycosylated mass peaks.

LC-MS of conjugate (**4**)

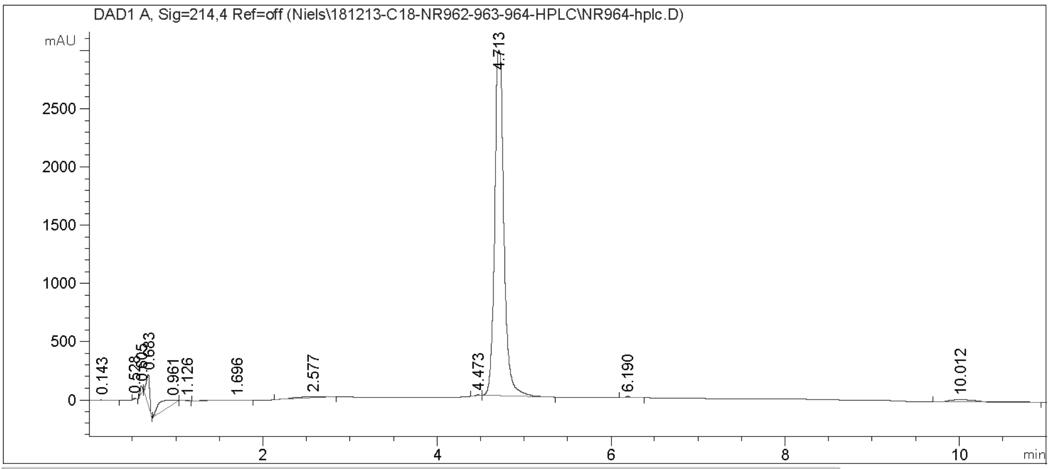

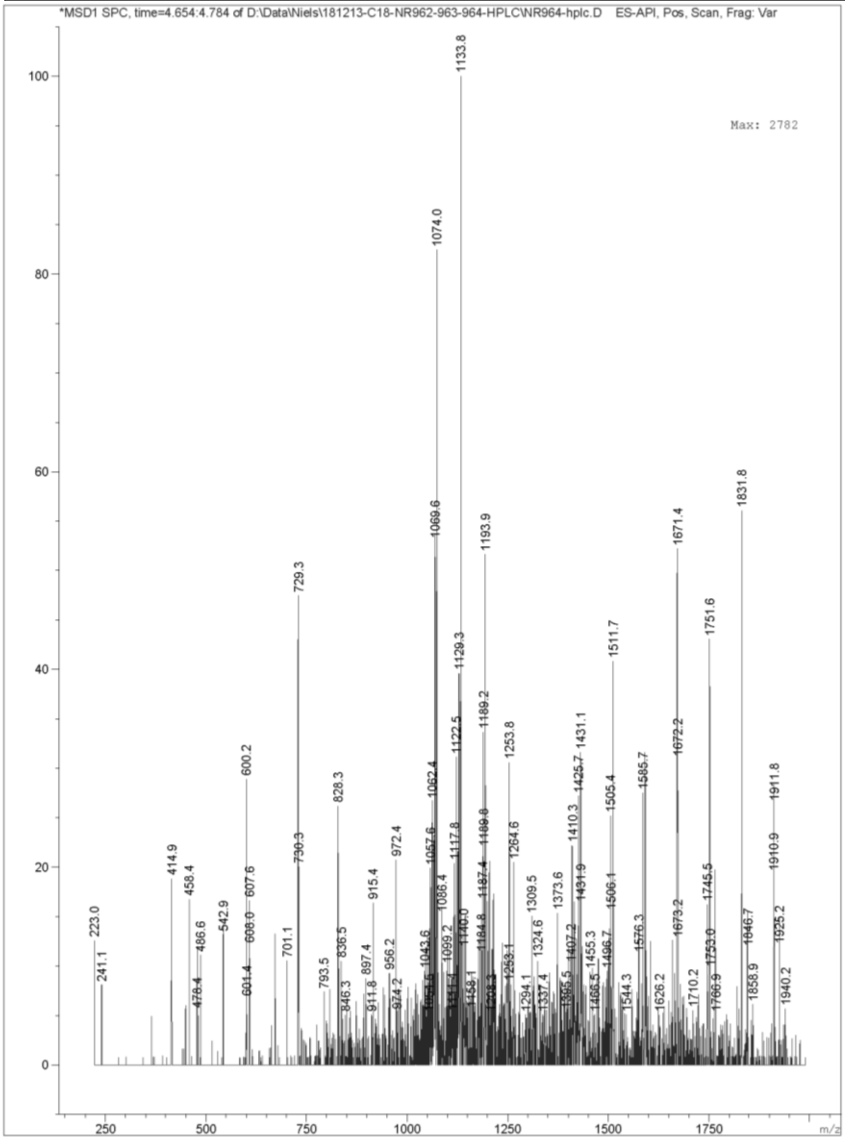

*Fragmentation occurs during MS, resulting in deglycosylated mass peaks.

LC-MS of conjugate (**5**)

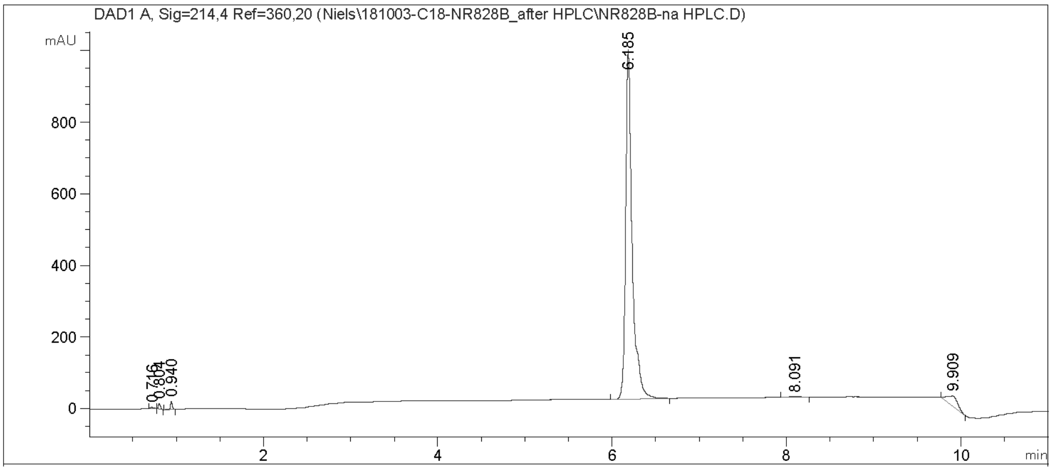

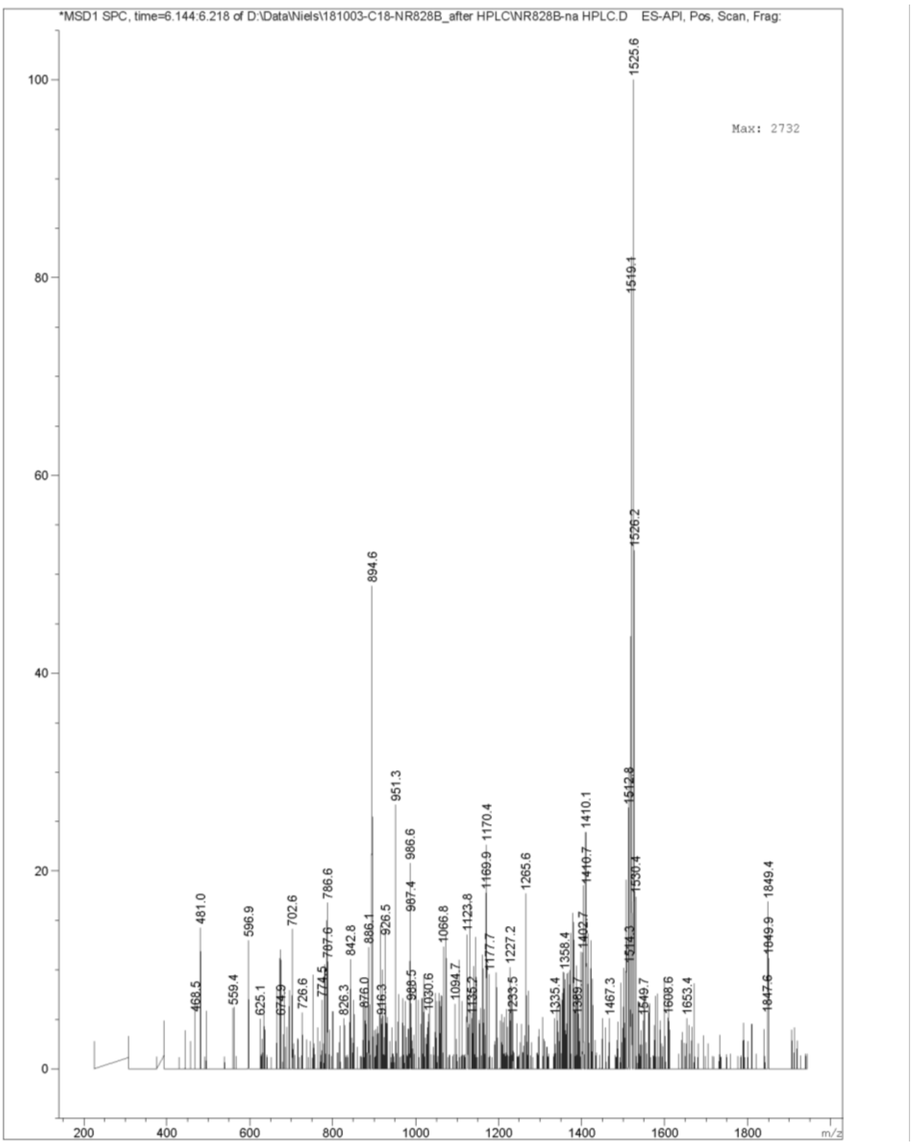

LC-MS of conjugate (**6**)

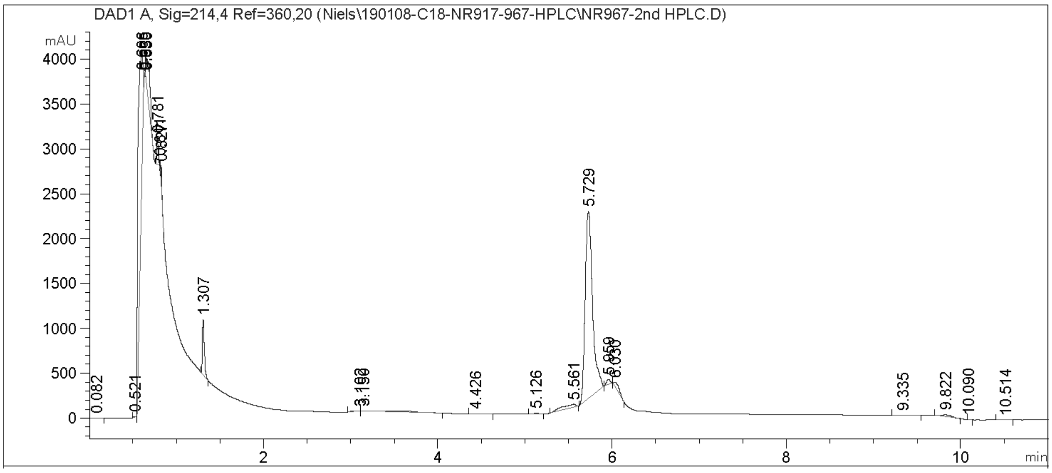

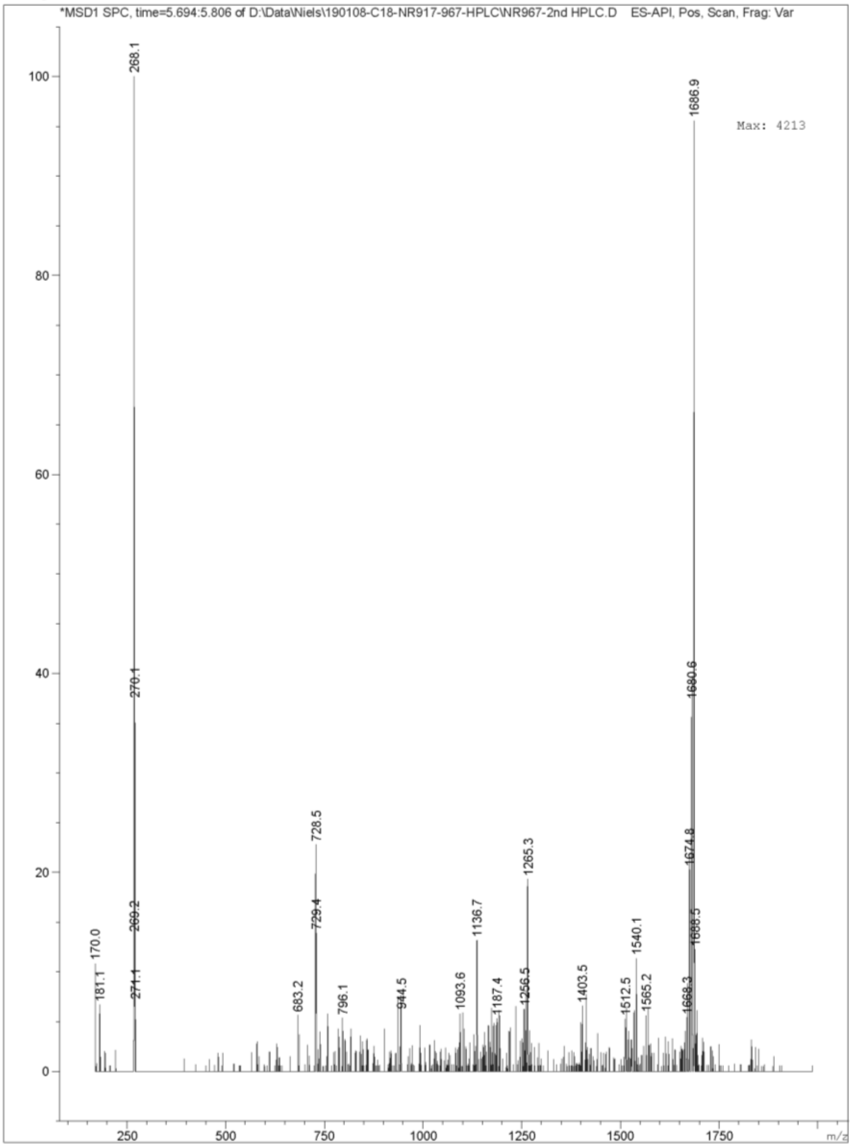

*Peak between 1-2 min comes from the DMSO.

LC-MS of conjugate (**7**)

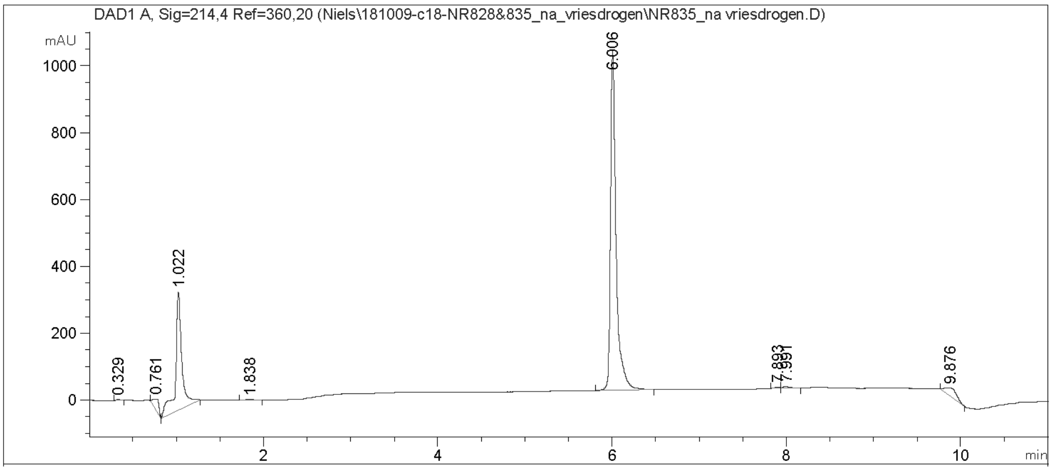

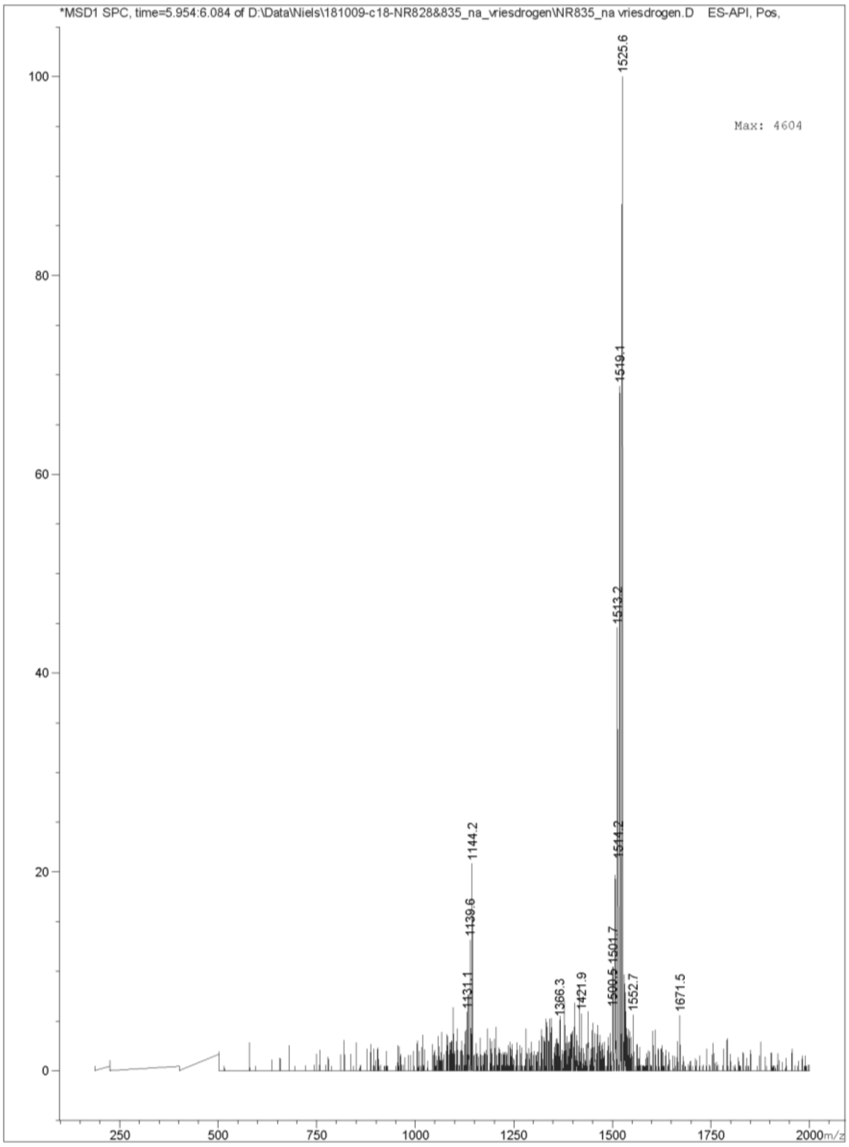

LC-MS of conjugate (**8**)

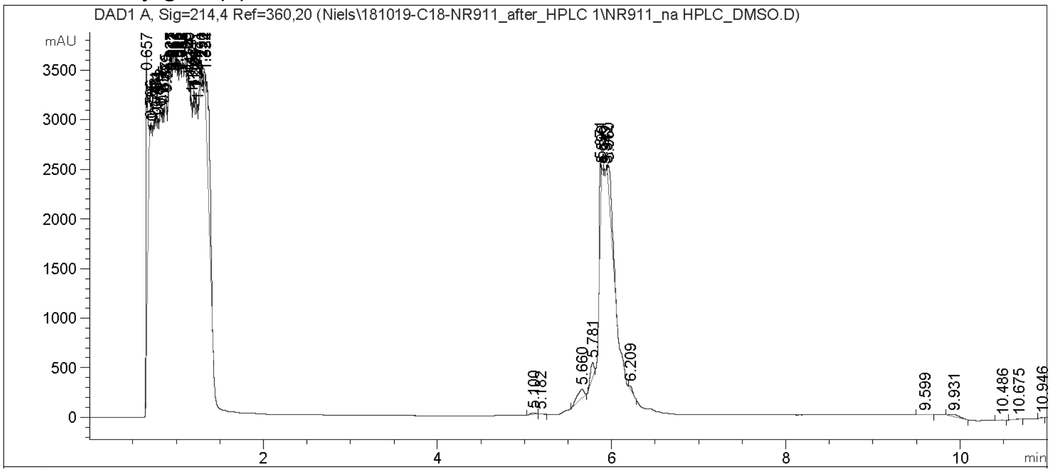

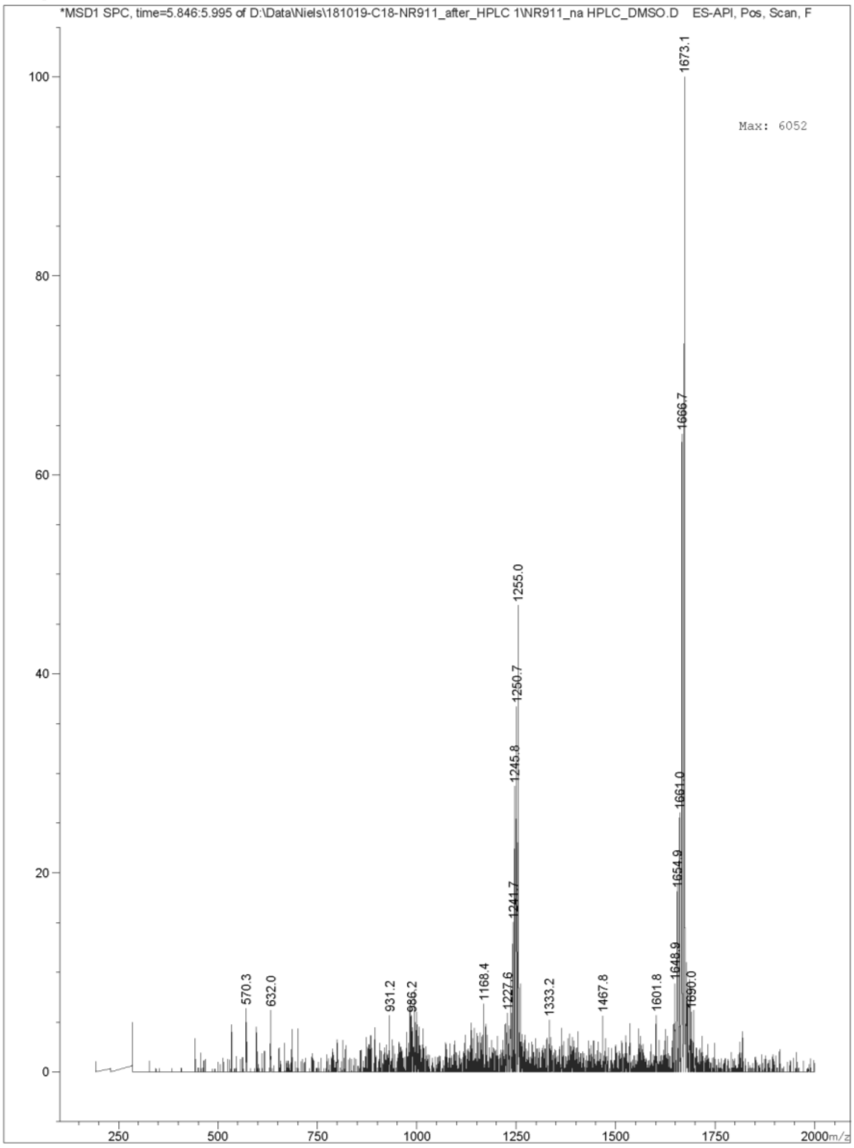

*Peak between 1-2 min comes from the DMSO.

LC-MS of compound **35**

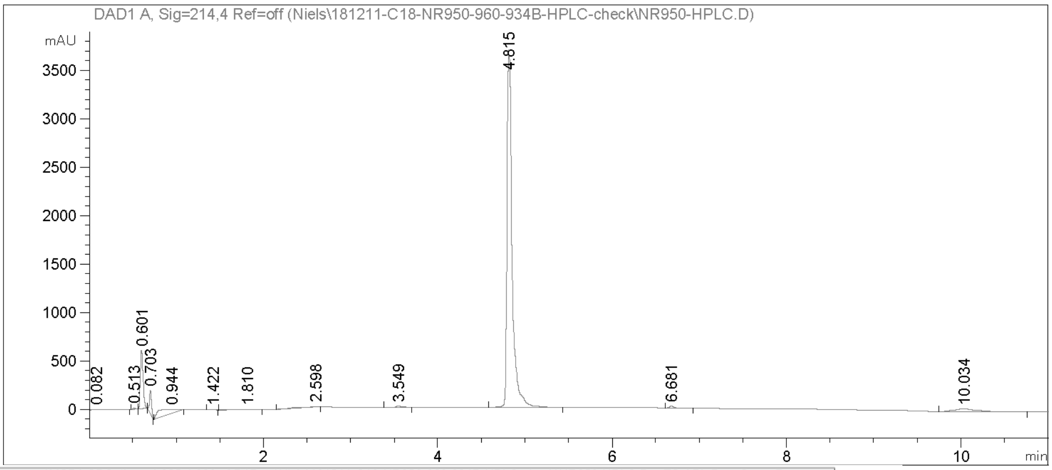

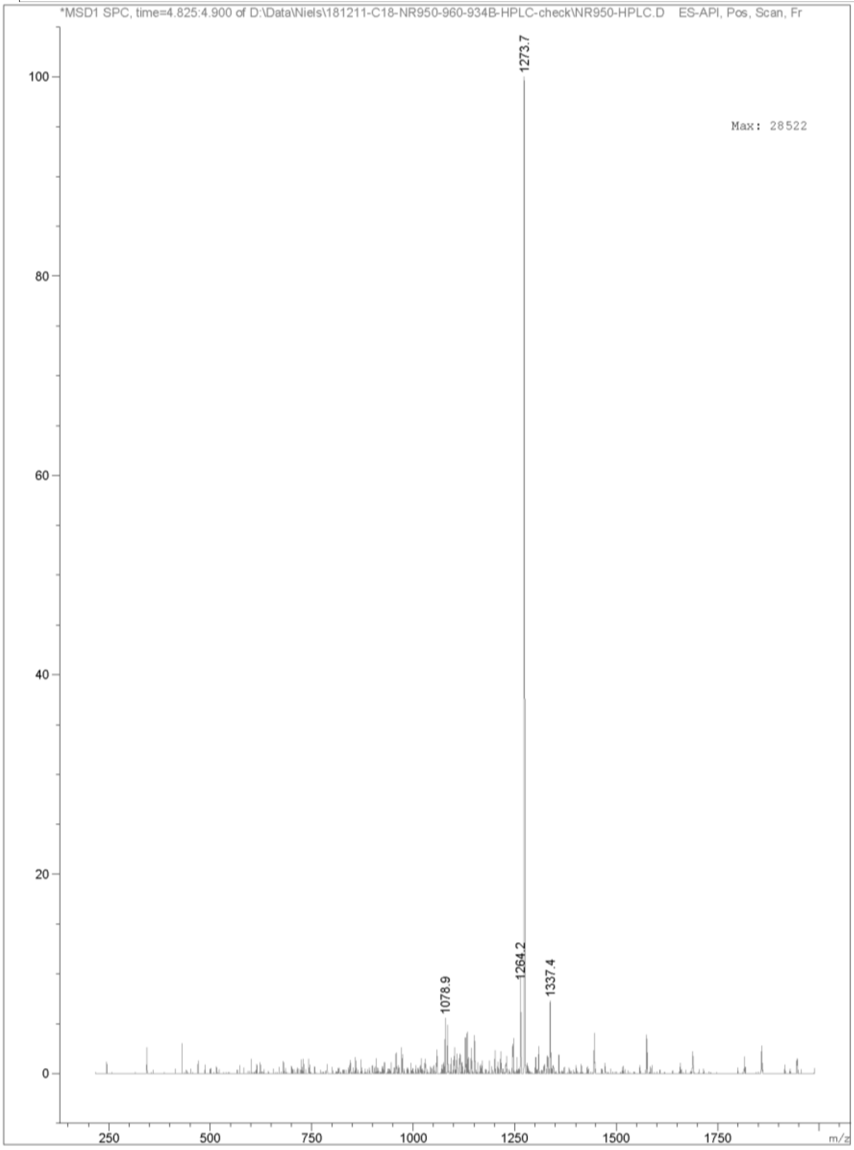

LC-MS of compound (**36**)

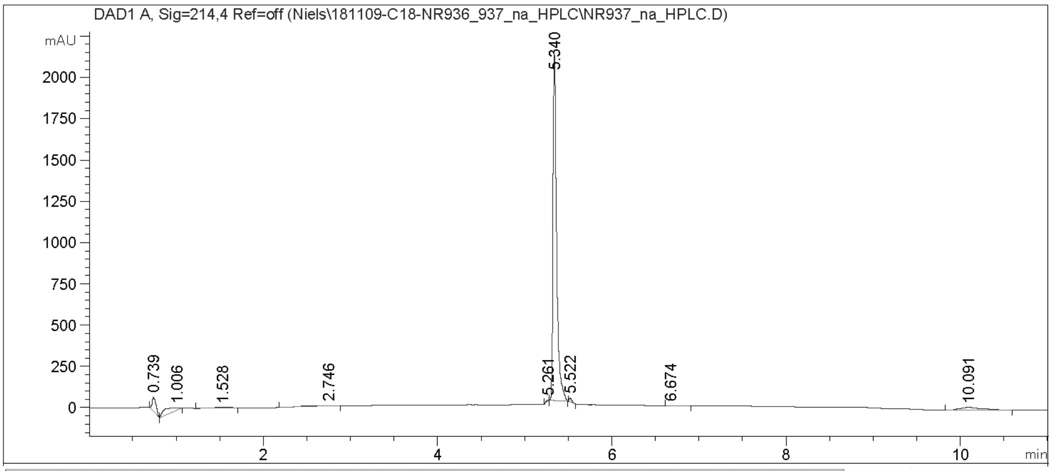

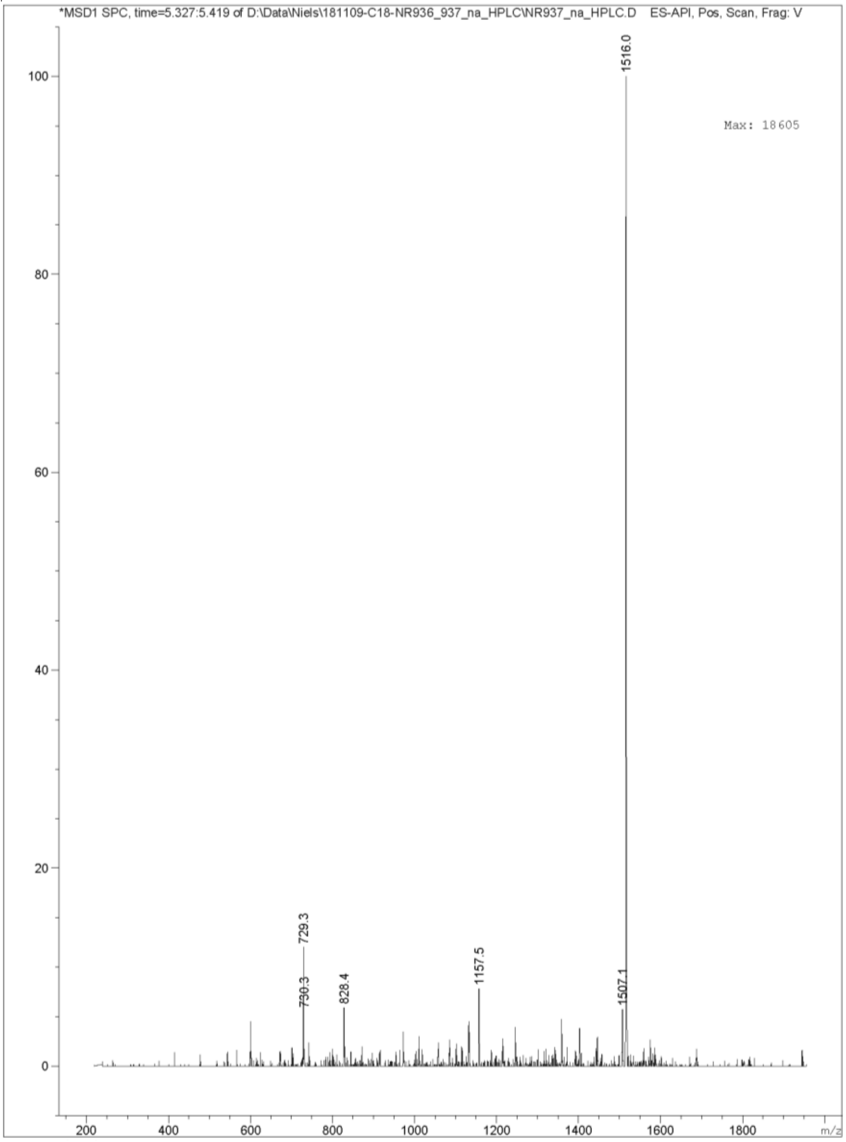

LC-MS of compound (**37**)

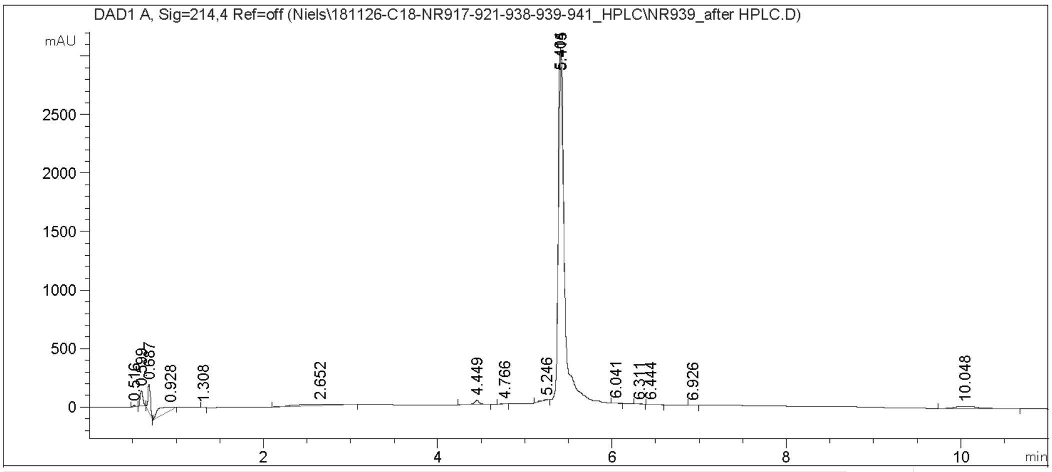

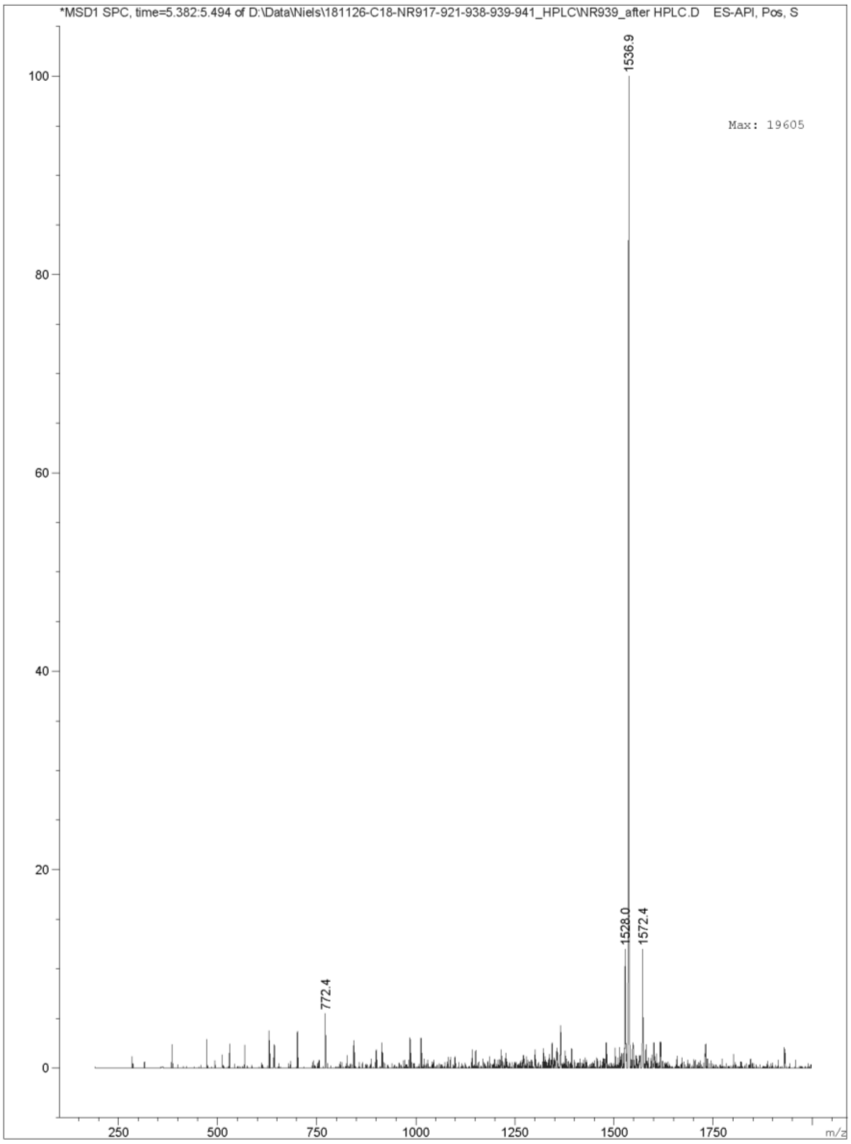

LC-MS of compound (**38**)

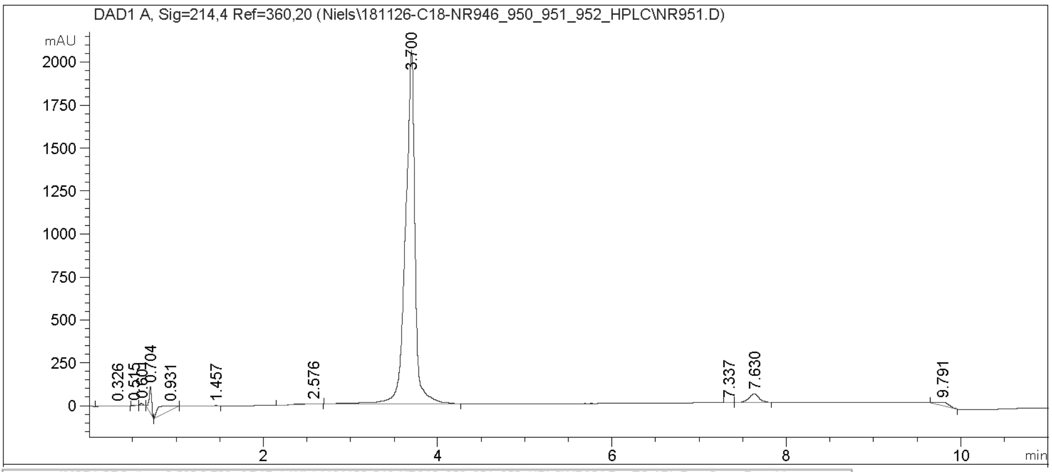

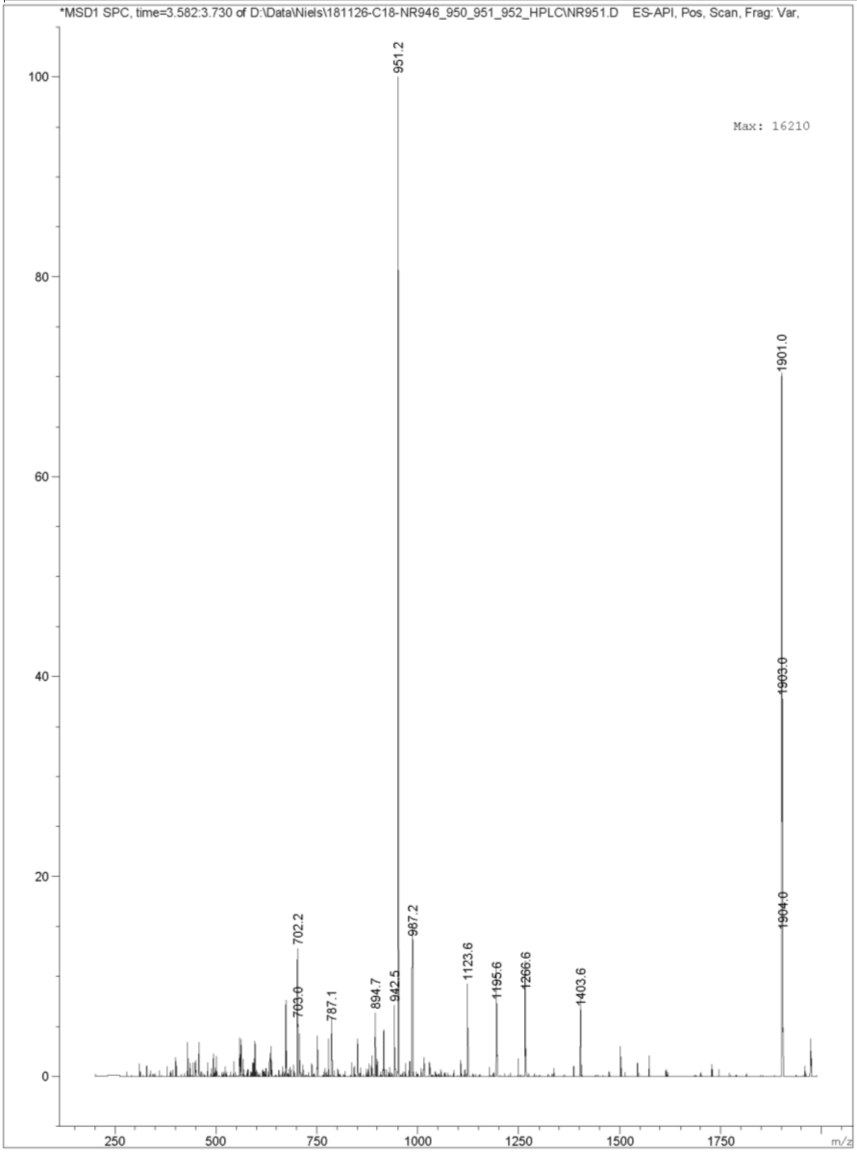

LC-MS of compound (**39**)

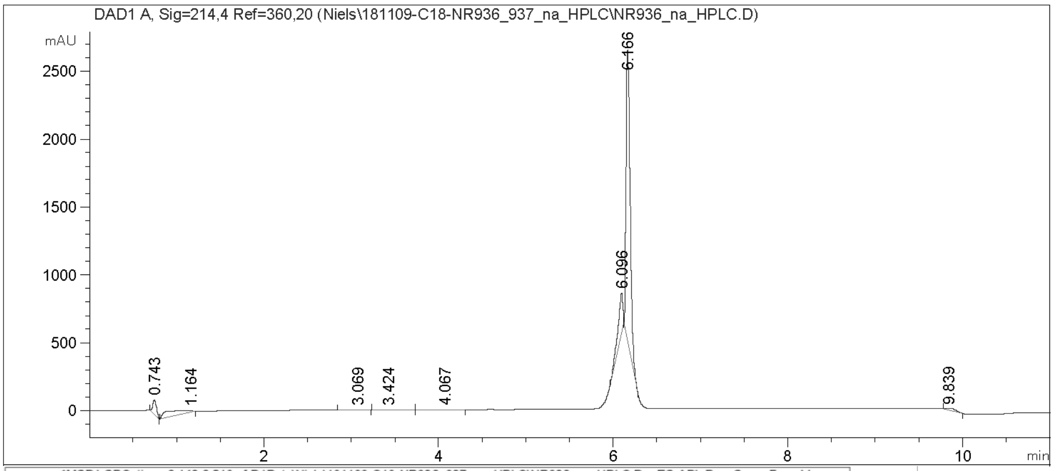

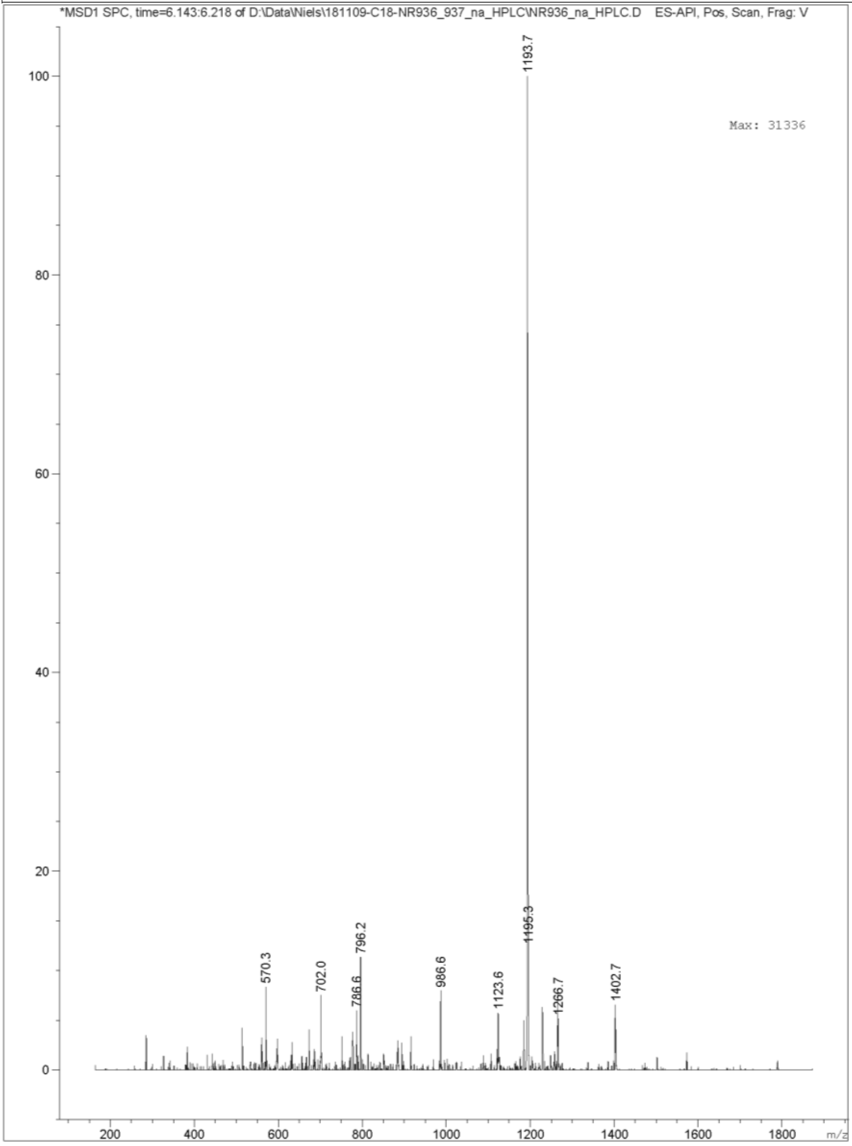

LC-MS of compound (**40**)

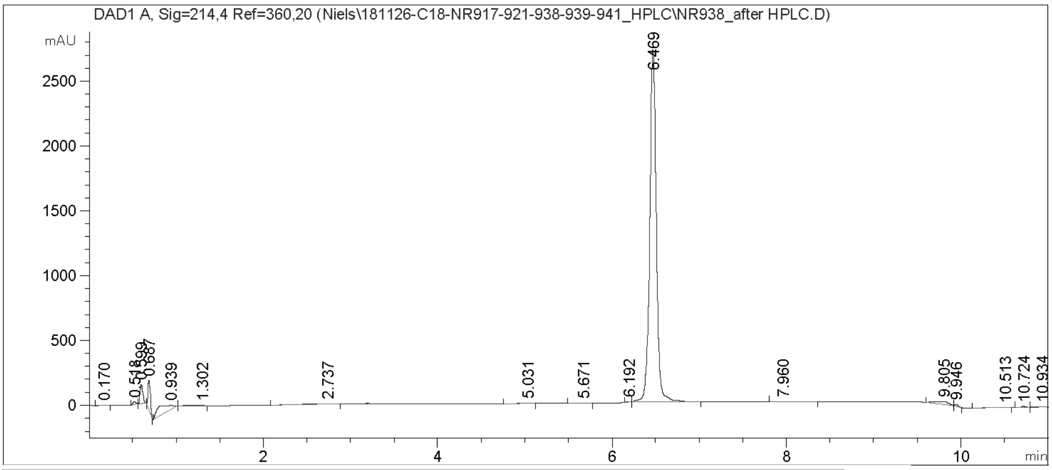

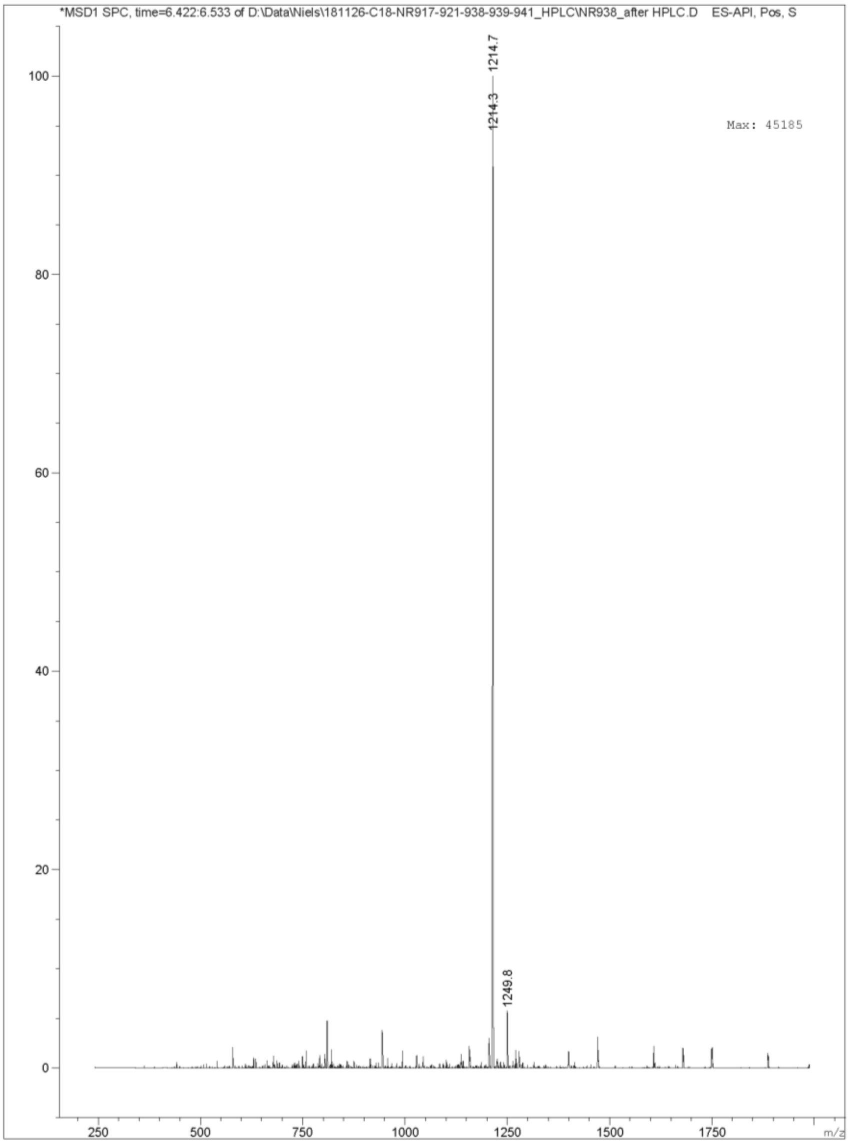

LC-MS of compound (**S9**)

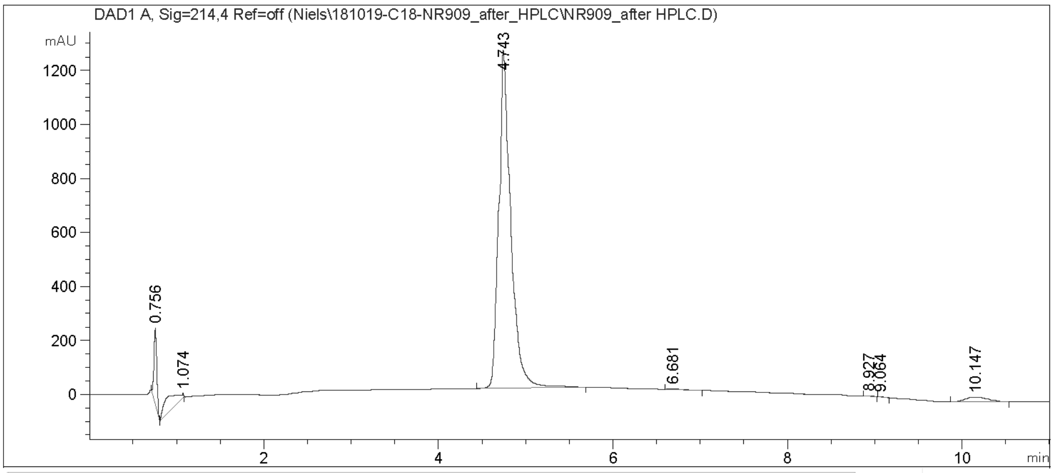

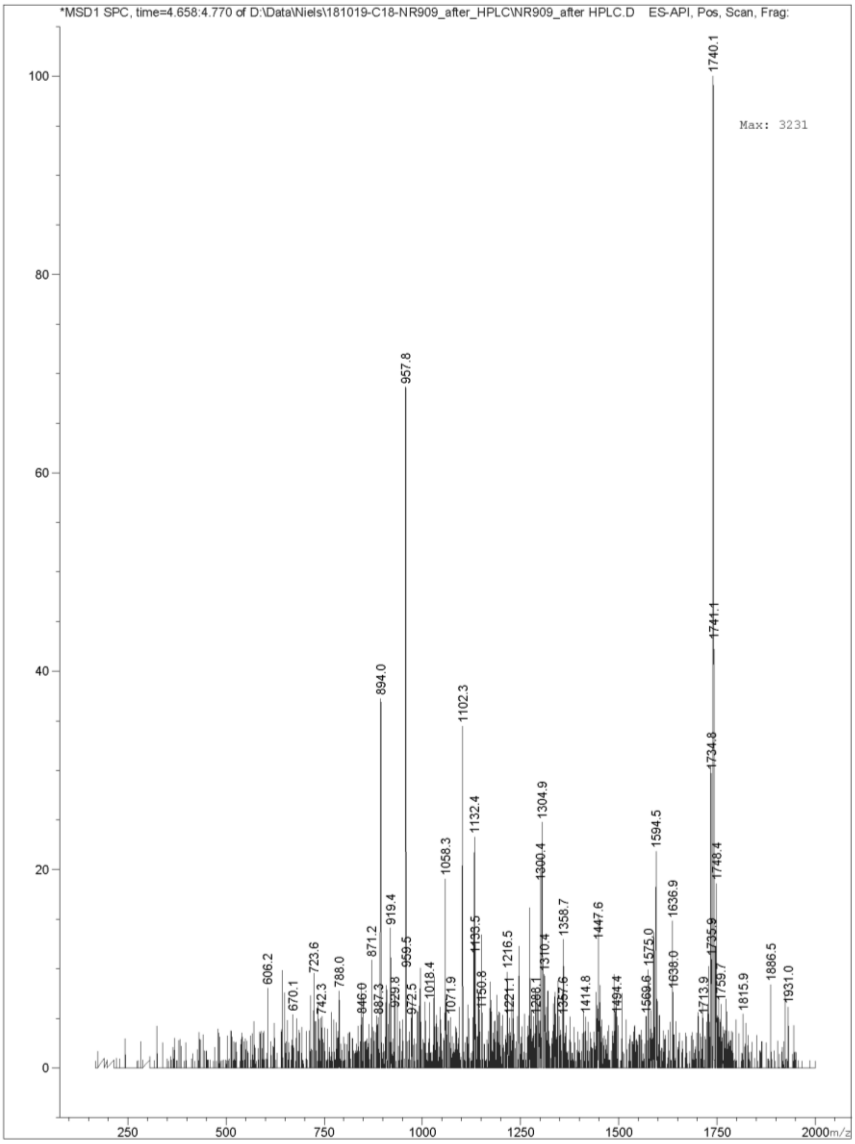

NMR spectra

^{1}H NMR, 400 MHz, $CDCl_3$ of compound **11**

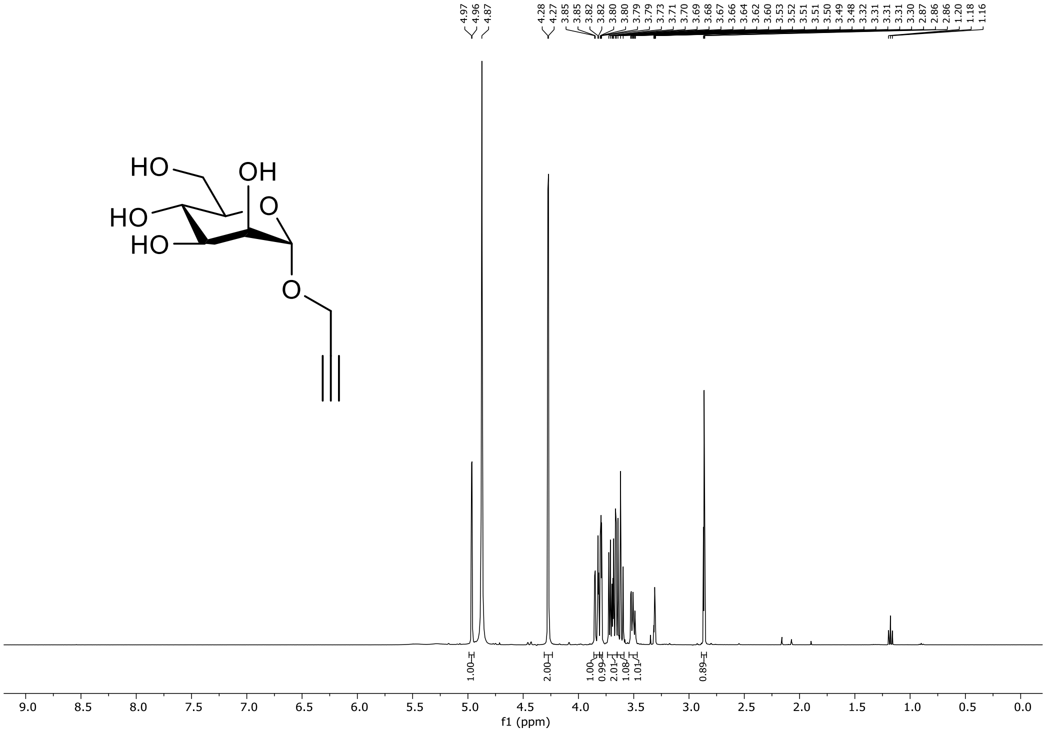

^{13}C-APT NMR, 101 MHz, $CDCl_3$ of compound **11**

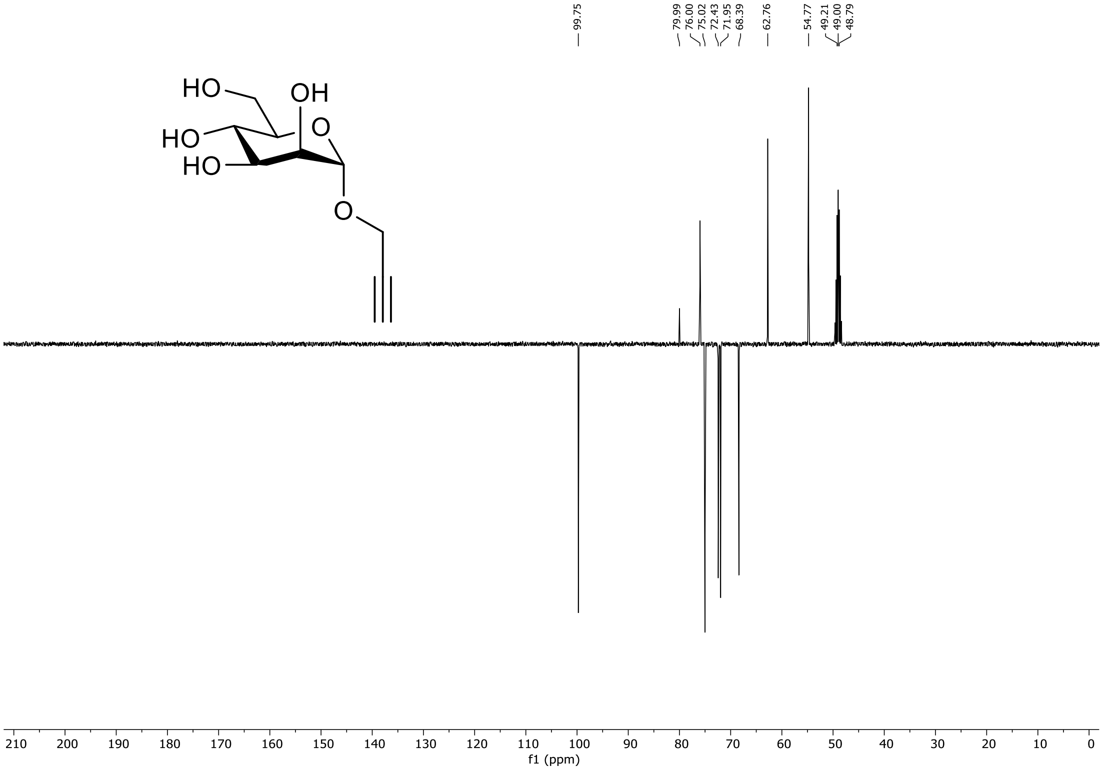

^{1}H-^{1}H COSY of compound **11**

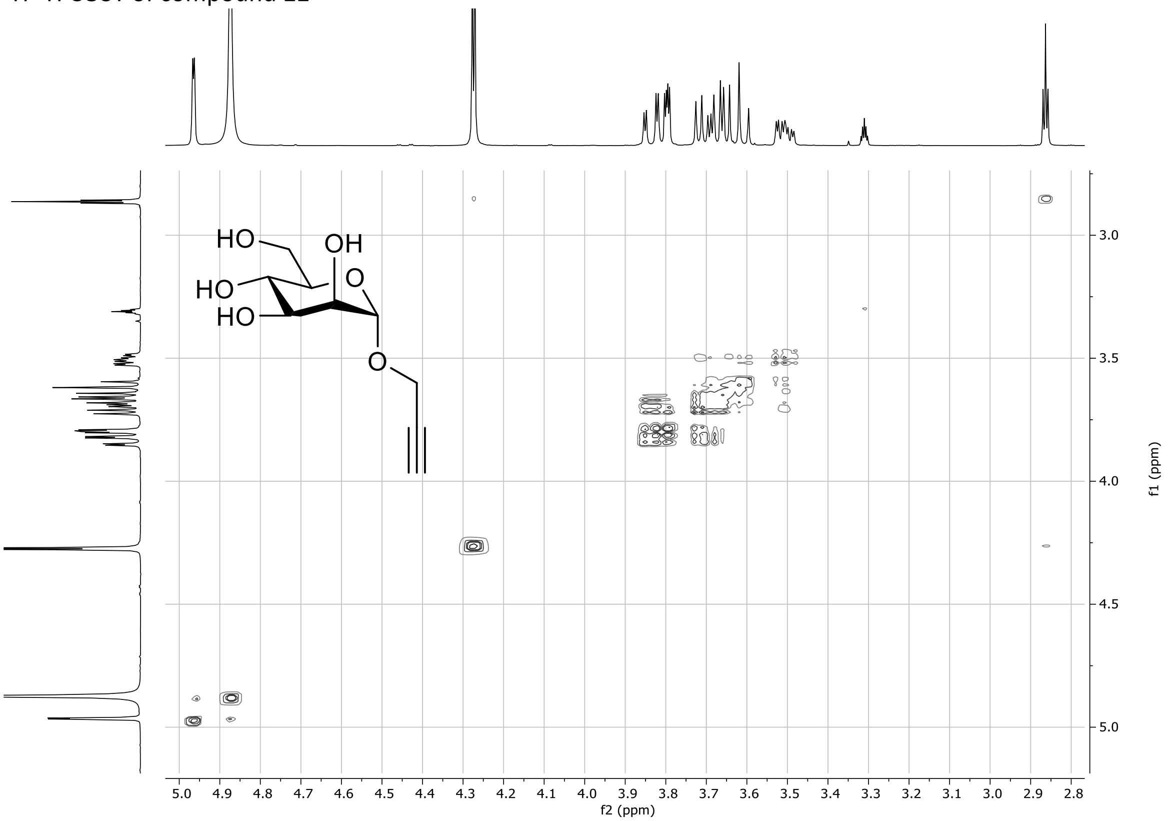

^{1}H-^{13}C HSQC of compound **11**

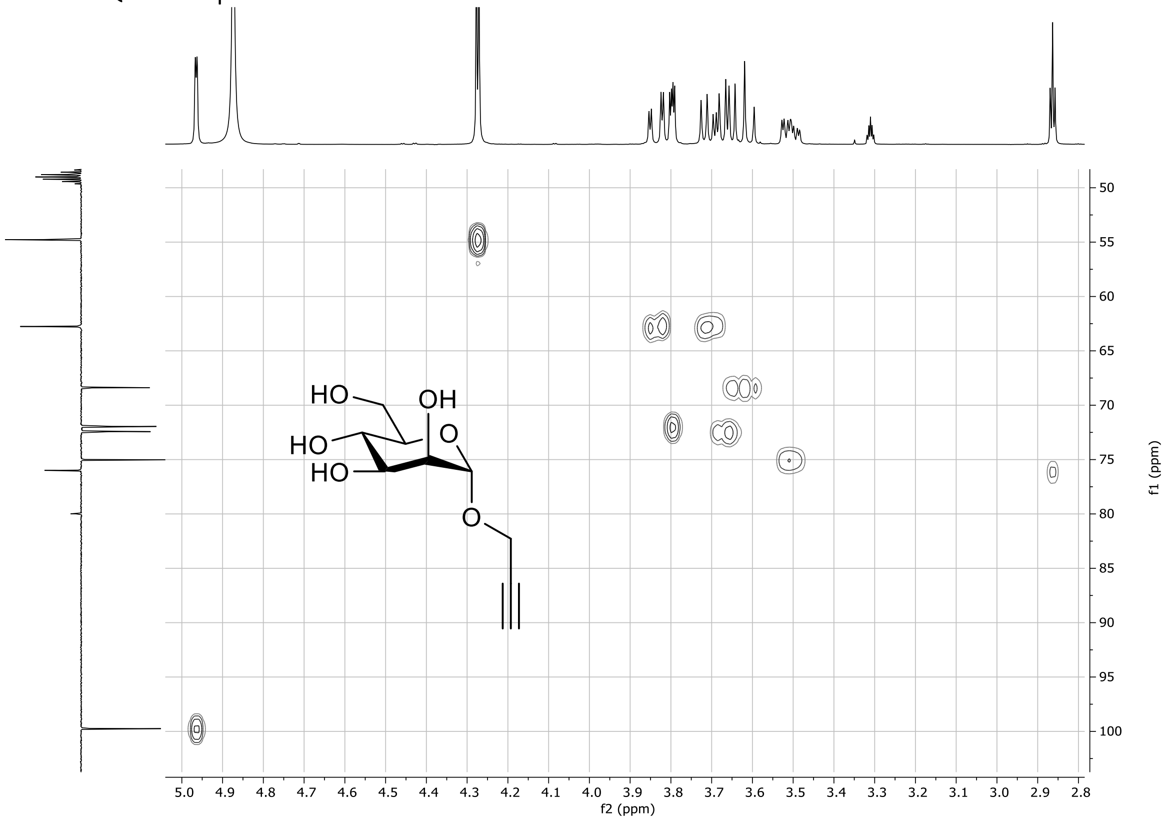

^{1}H NMR, 400 MHz, MeOD of compound **S1**

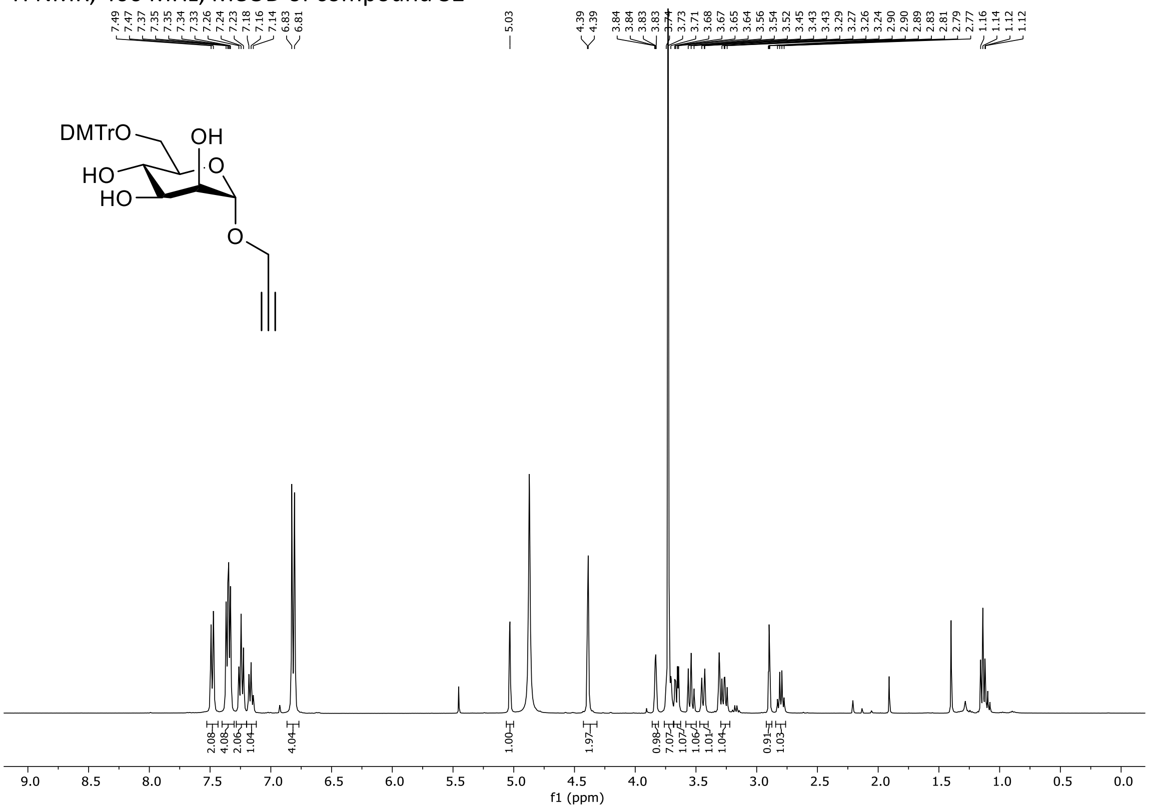

^{13}C-APT NMR, 101 MHz, MeOD of compound **S1**

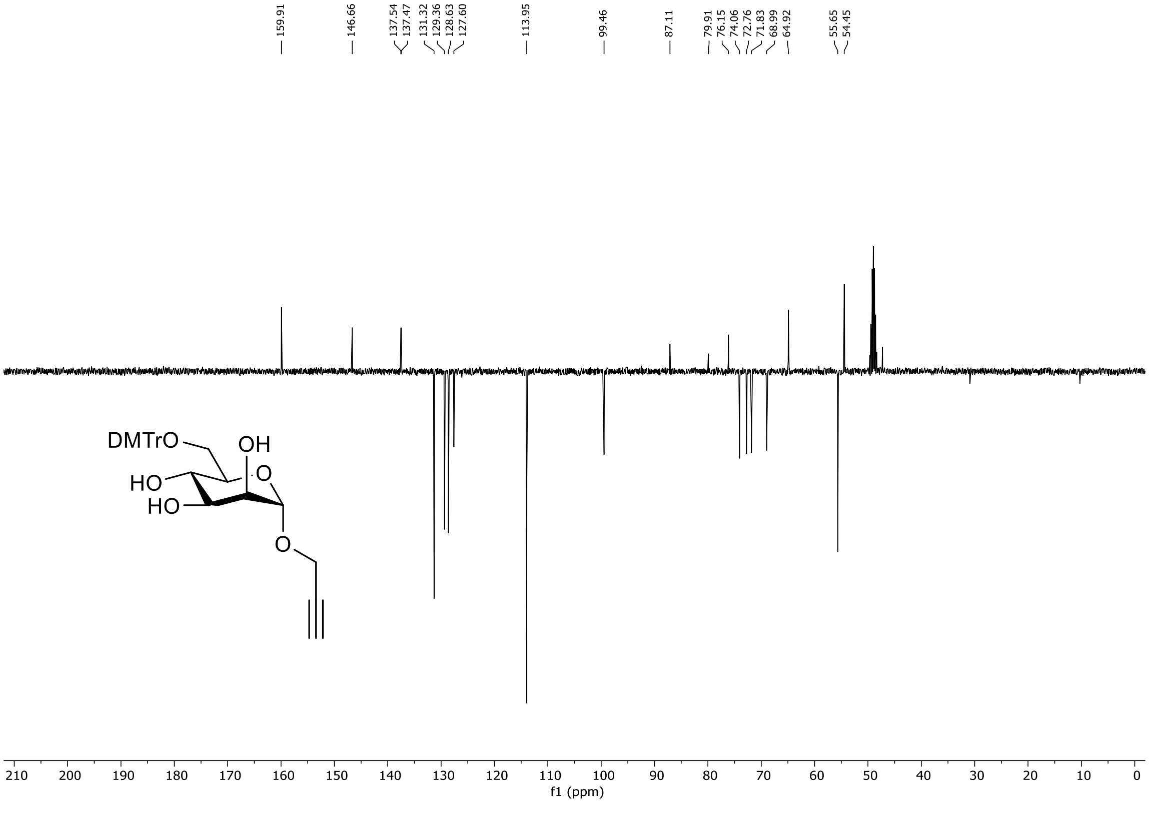

^1H-^1H COSY of compound **S1**

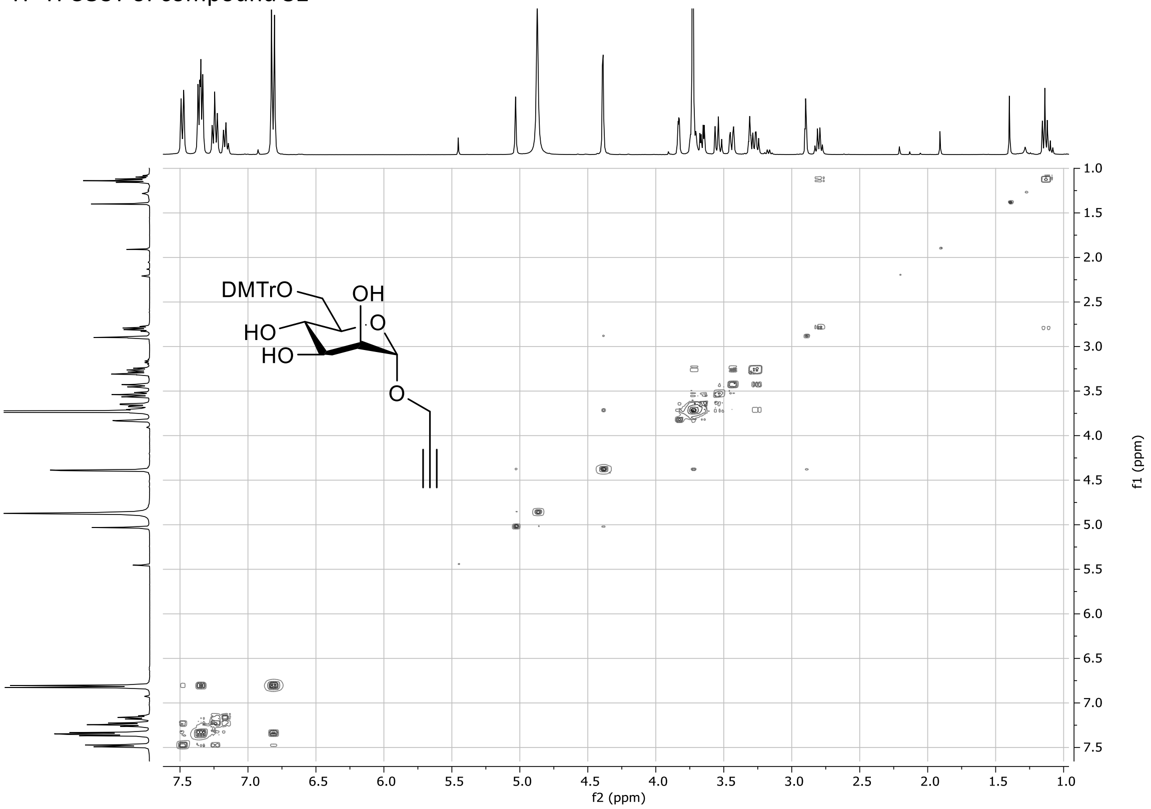

^1H-^{13}C HSQC of compound **S1**

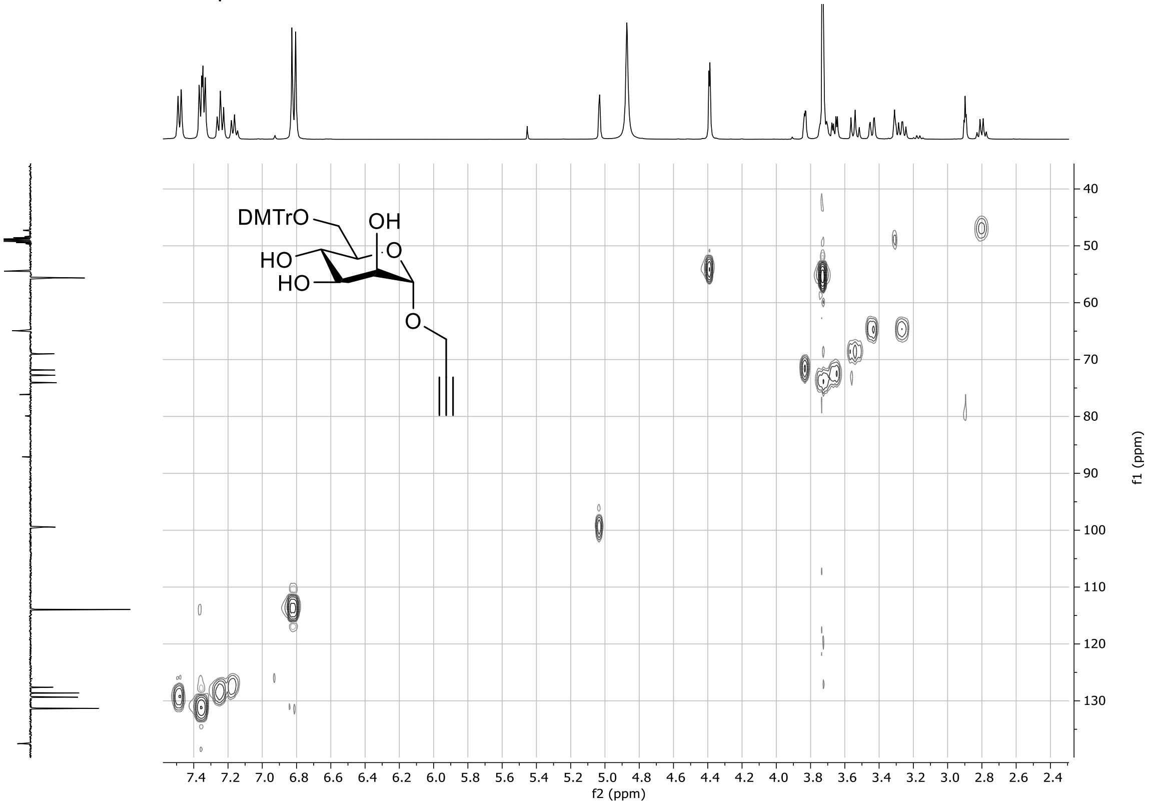

^{1}H NMR, 400 MHz, CD_2Cl_2 of compound **S2**

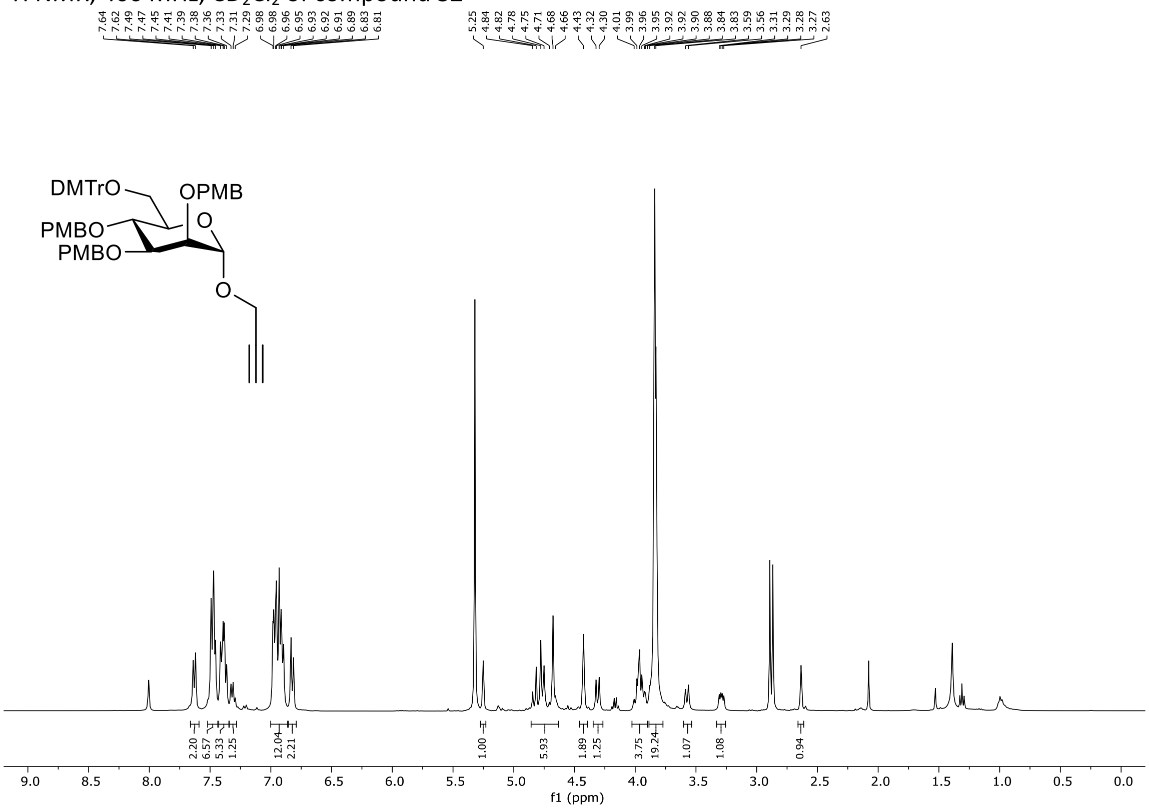

^{13}C-APT NMR, 101 MHz, CD_2Cl_2 of compound **S2**

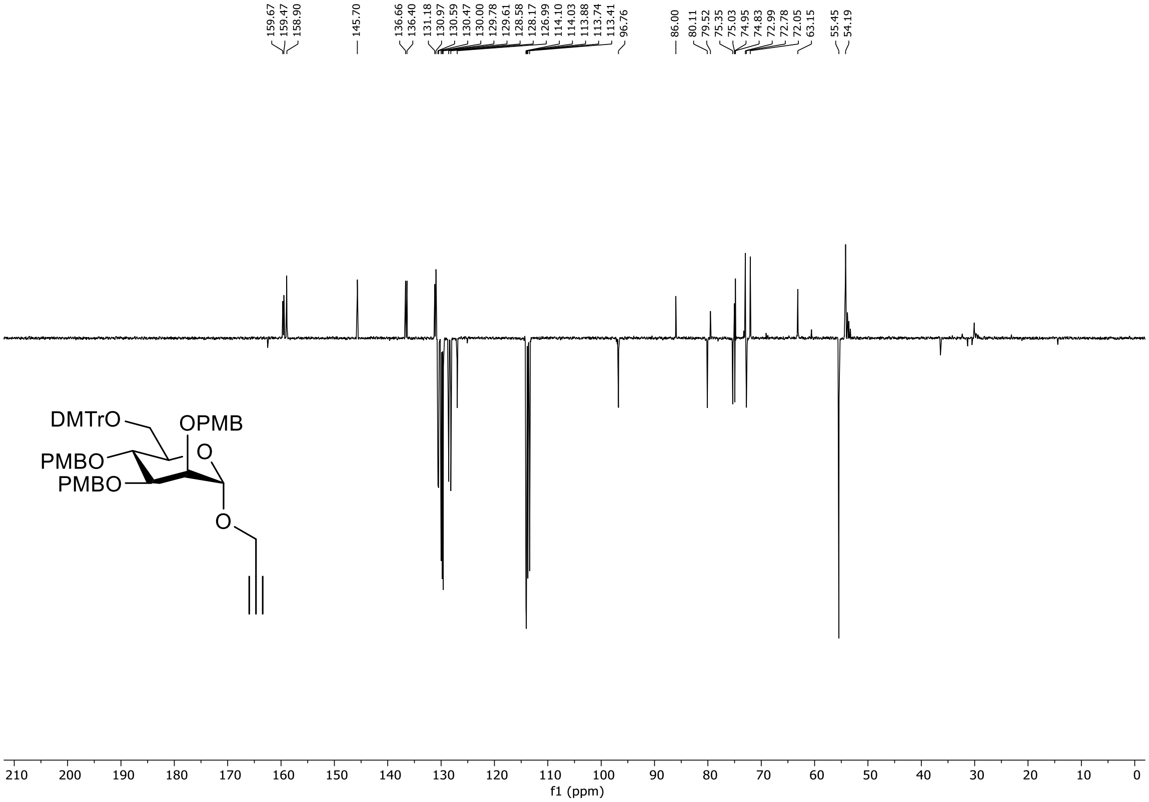

^{1}H-^{1}H COSY of compound **S2**

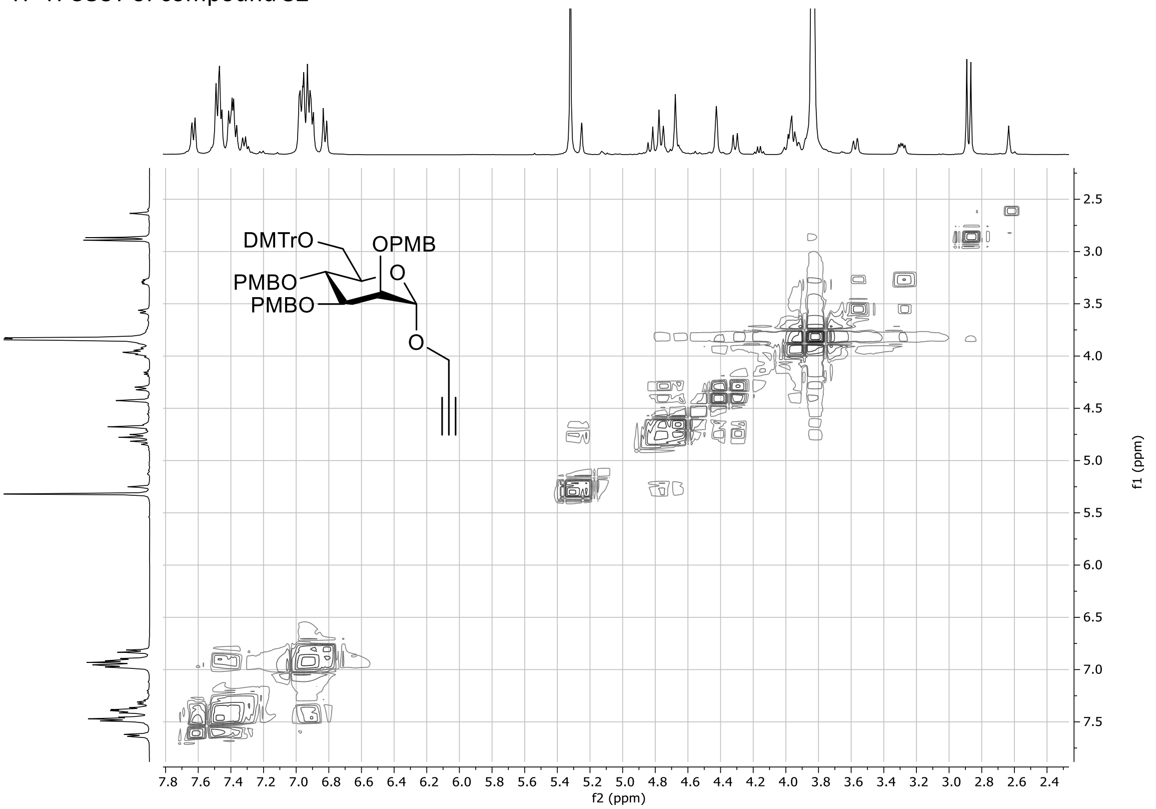

^{1}H-^{13}C HSQC of compound **S2**

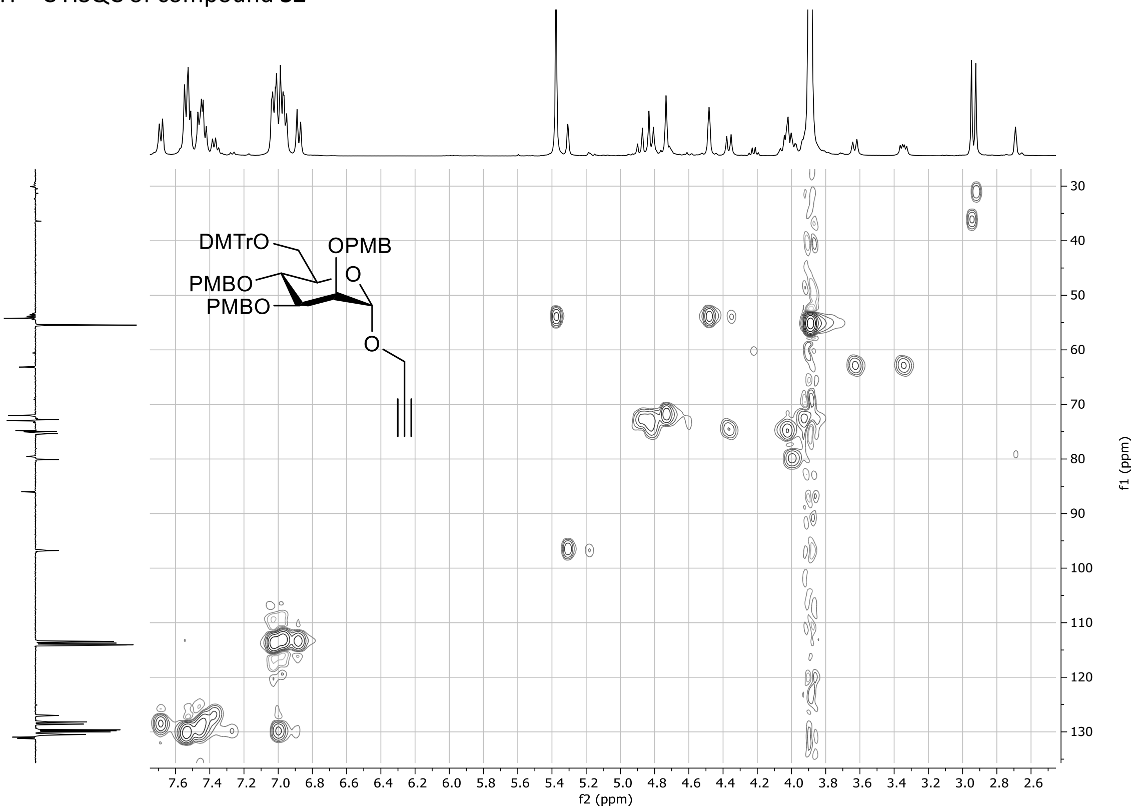

^{1}H NMR, 400 MHz, $CDCl_3$ of compound **S3**

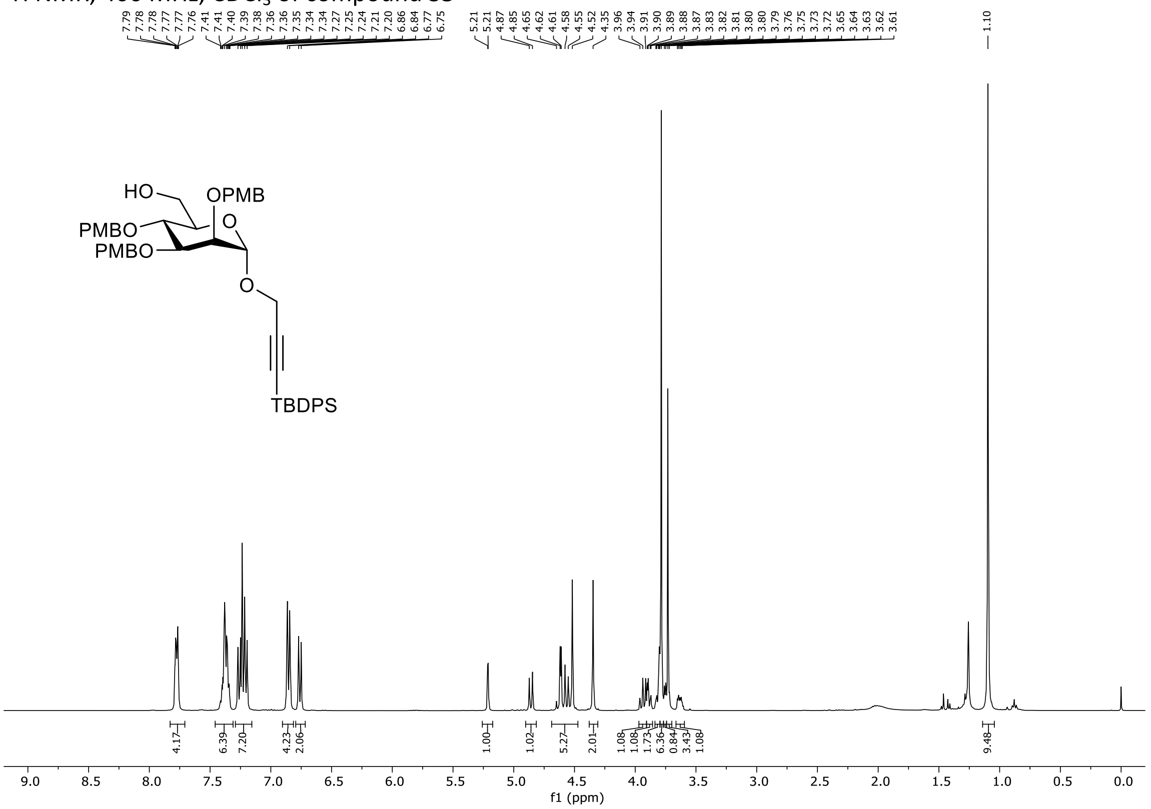

^{13}C-APT NMR, 101 MHz, $CDCl_3$ of compound **S3**

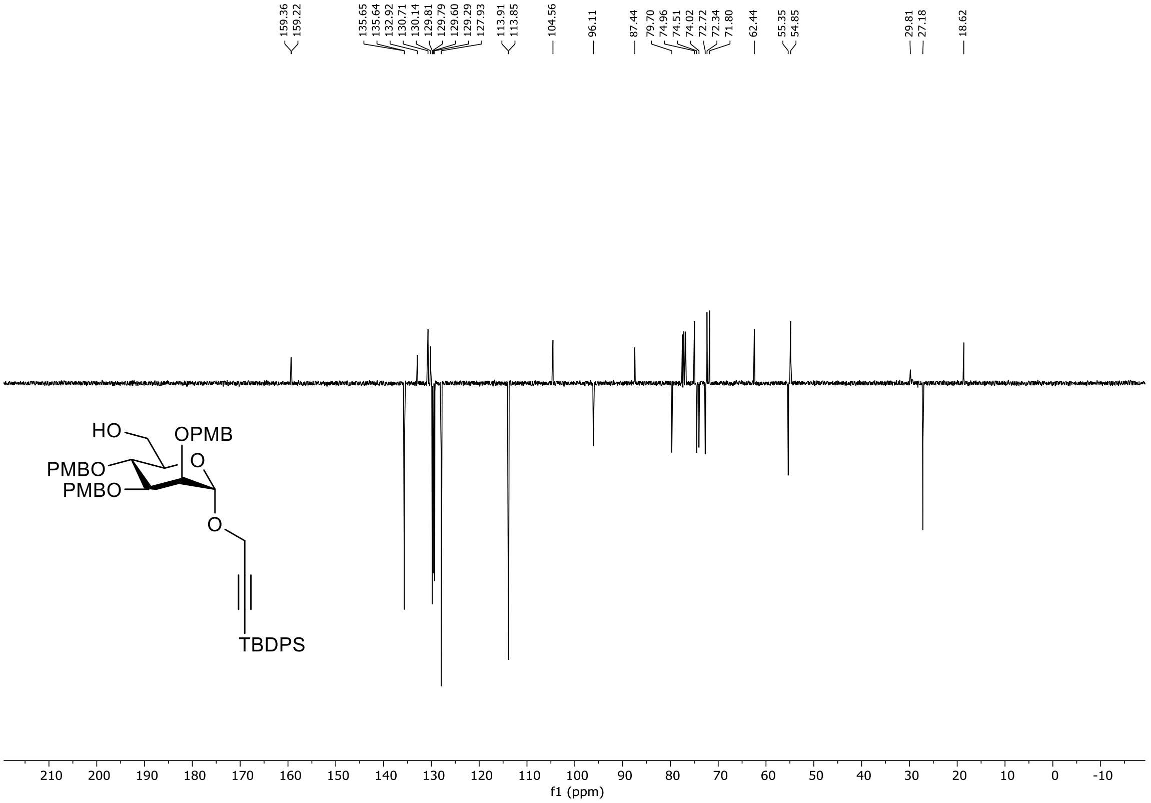

^{1}H-^{1}H COSY of compound **S3**

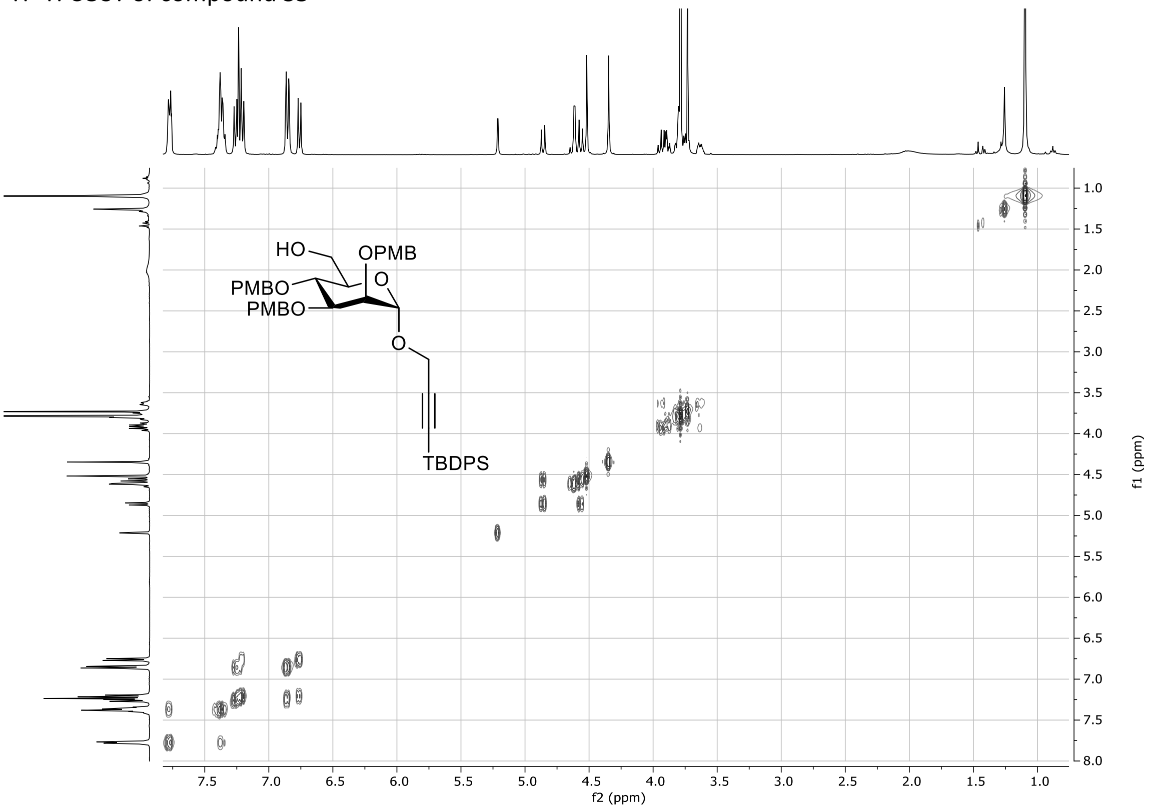

^{1}H-^{13}C HSQC of compound **S3**

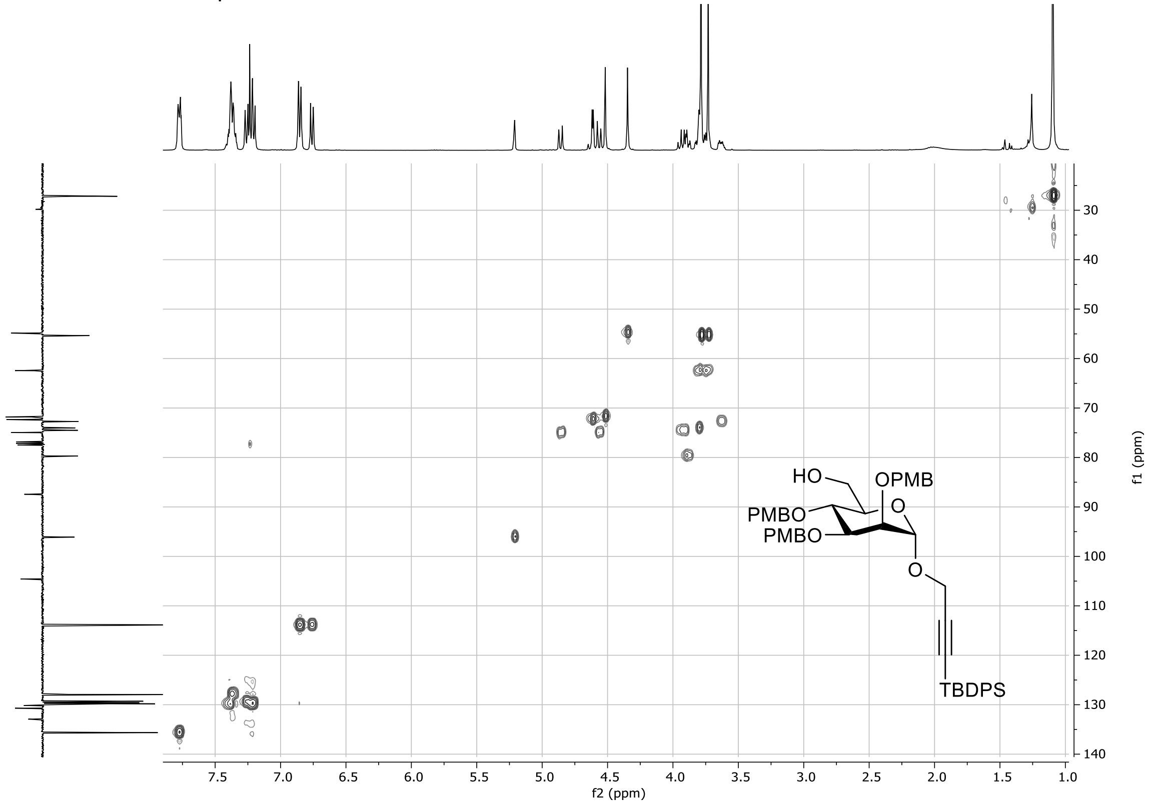

^{1}H NMR, 400 MHz, $CDCl_3$ of compound **S5**

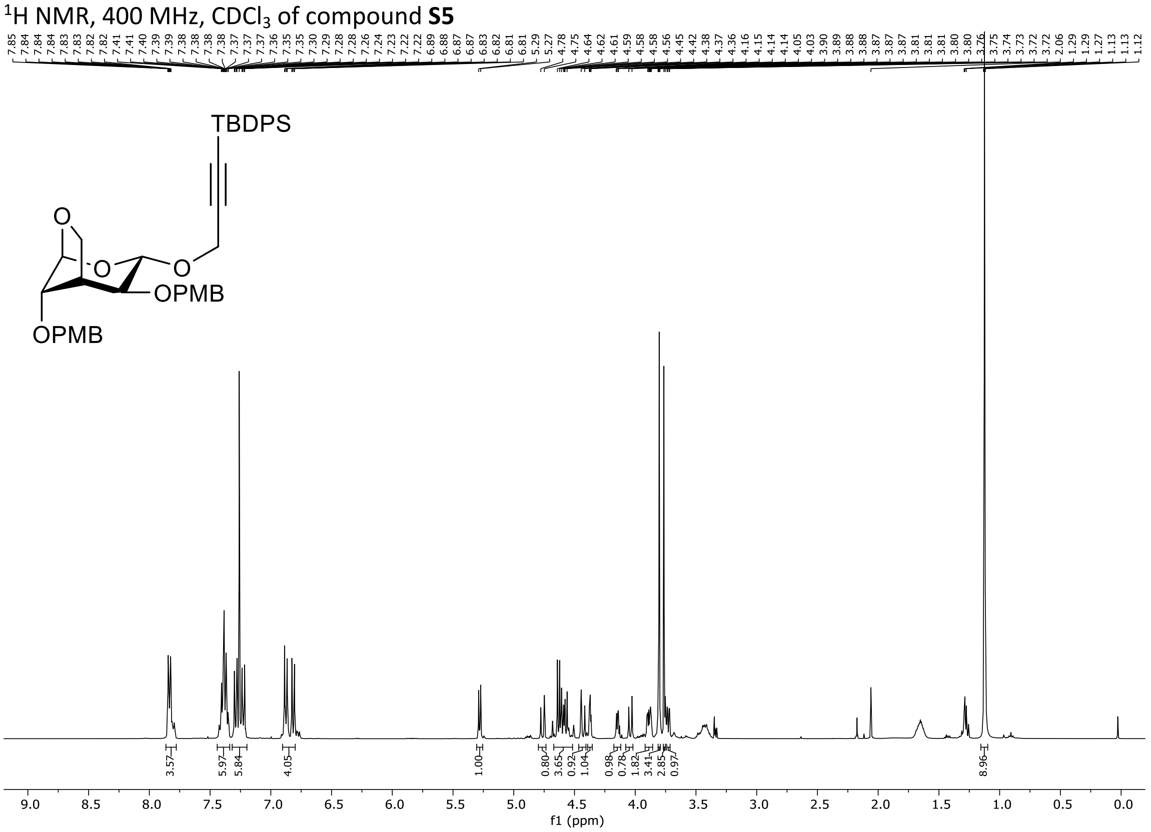

^{13}C-APT NMR, 101 MHz, $CDCl_3$ of compound **S5**

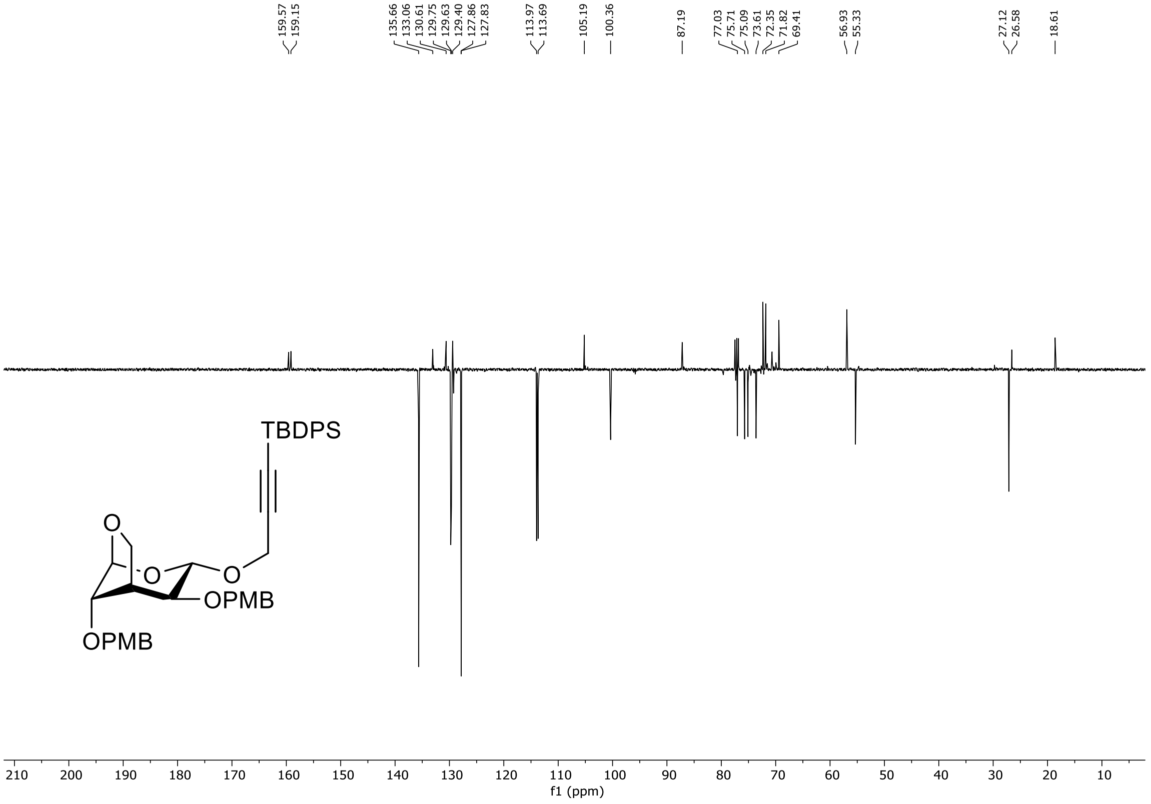

^{1}H-^{1}H COSY of compound **S5**

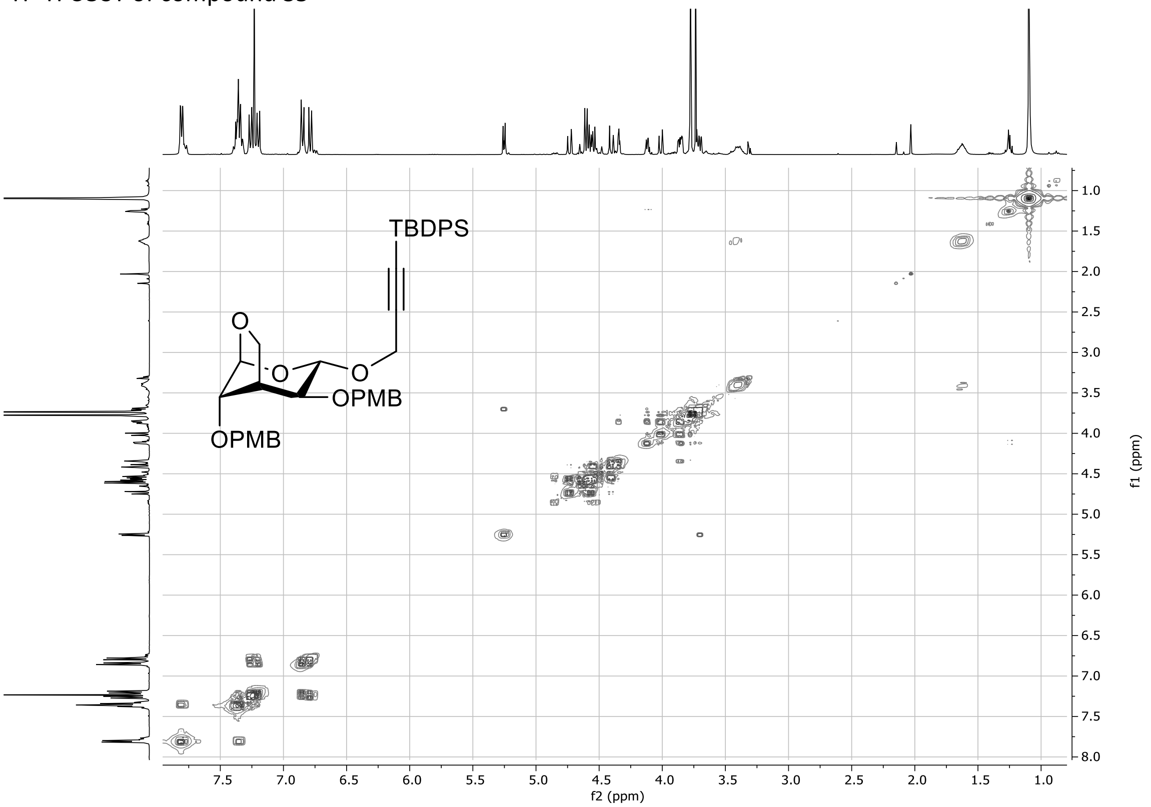

^{1}H-^{13}C HSQC of compound **S5**

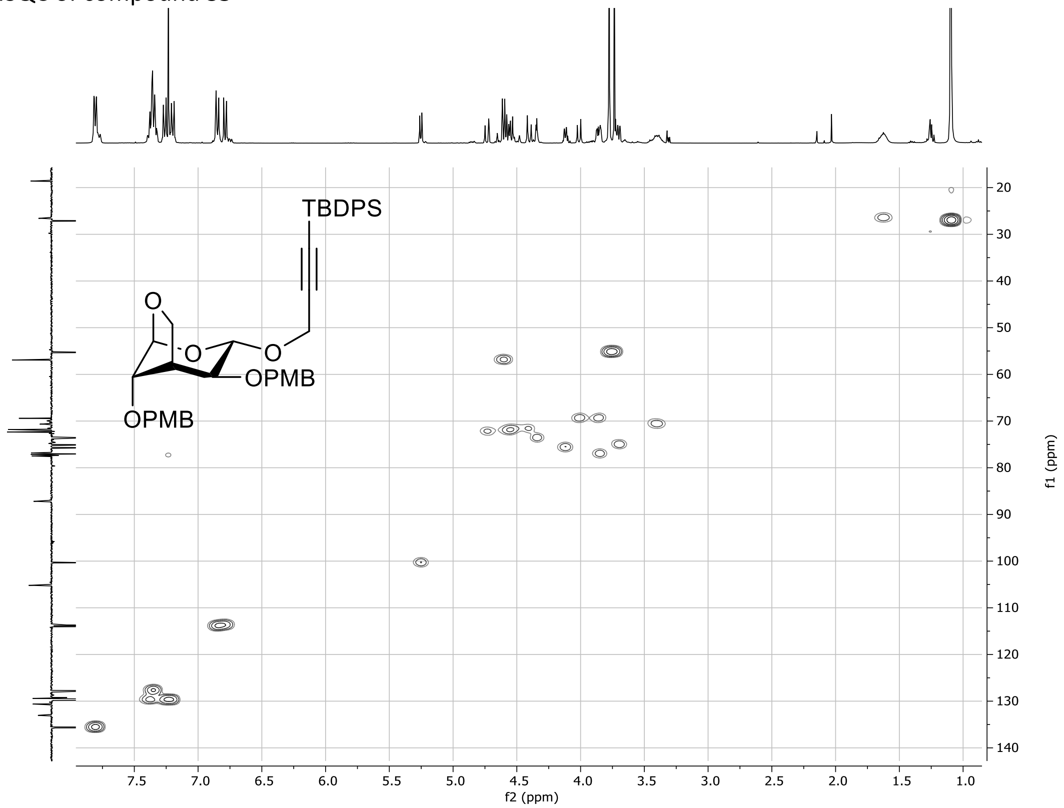

^{1}H NMR, 400 MHz, $CDCl_3$ of compound **12**

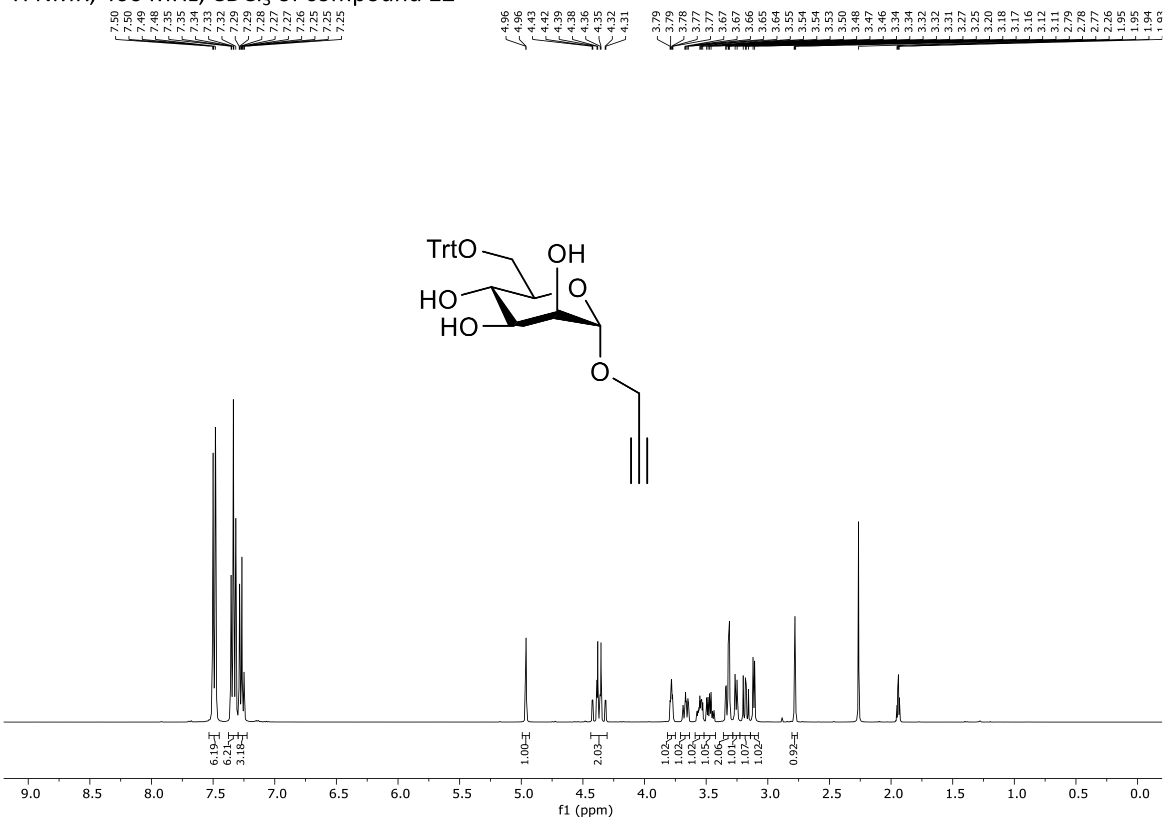

^{13}C-APT NMR, 101 MHz, $CDCl_3$ of compound **12**

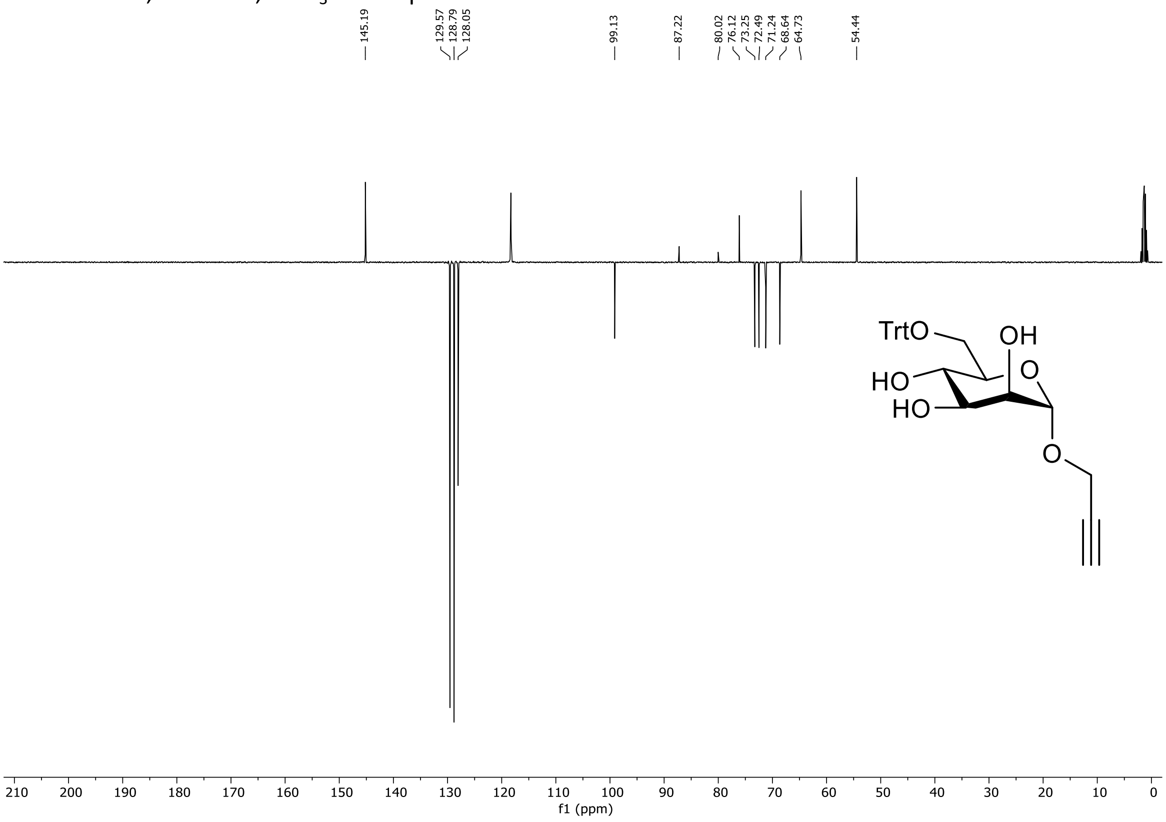

^{1}H-^{1}H COSY of compound **12**

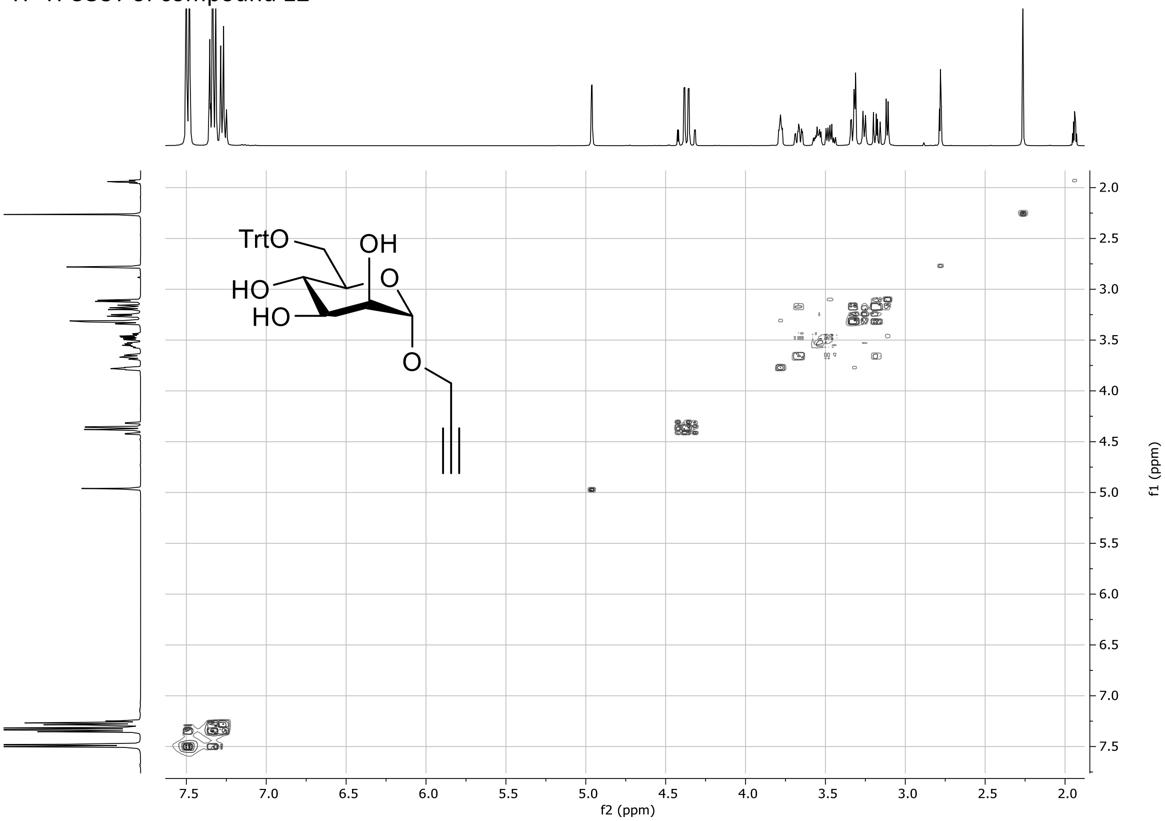

^{1}H-^{13}C HSQC of compound **12**

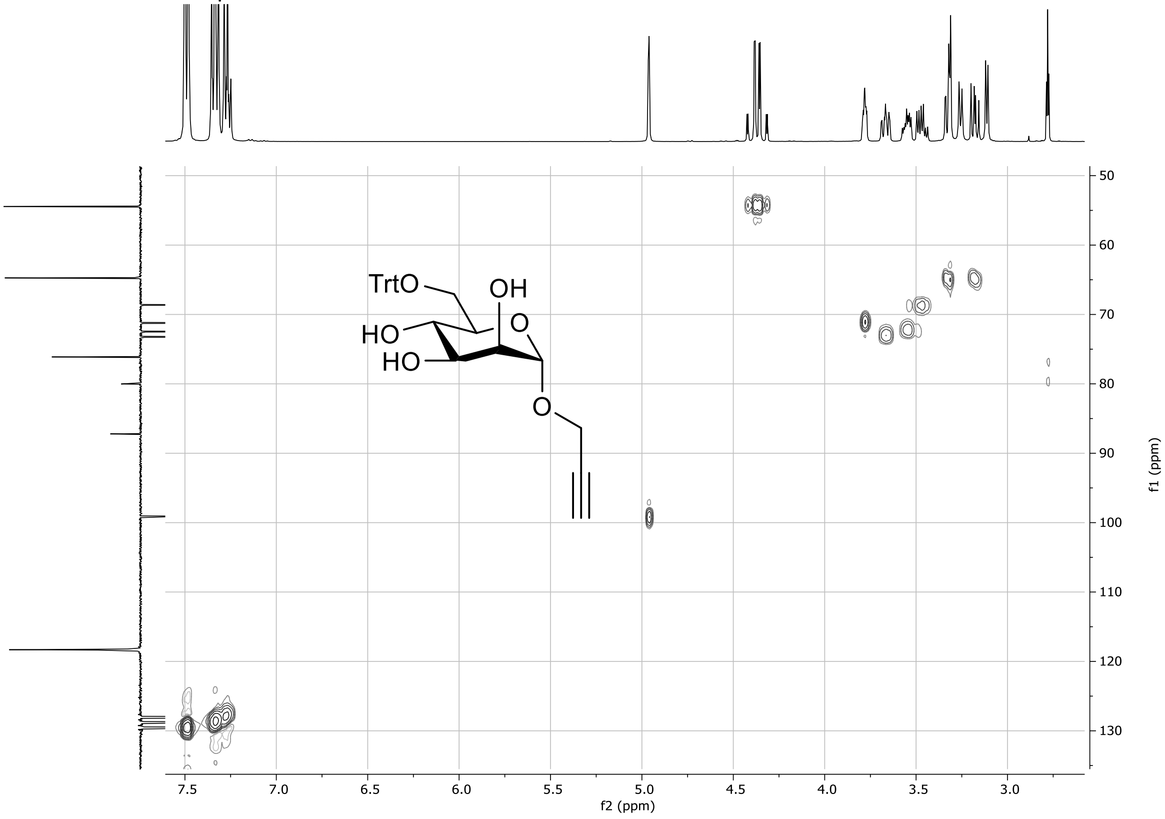

^{1}H NMR, 400 MHz, $CDCl_3$ of compound **13**

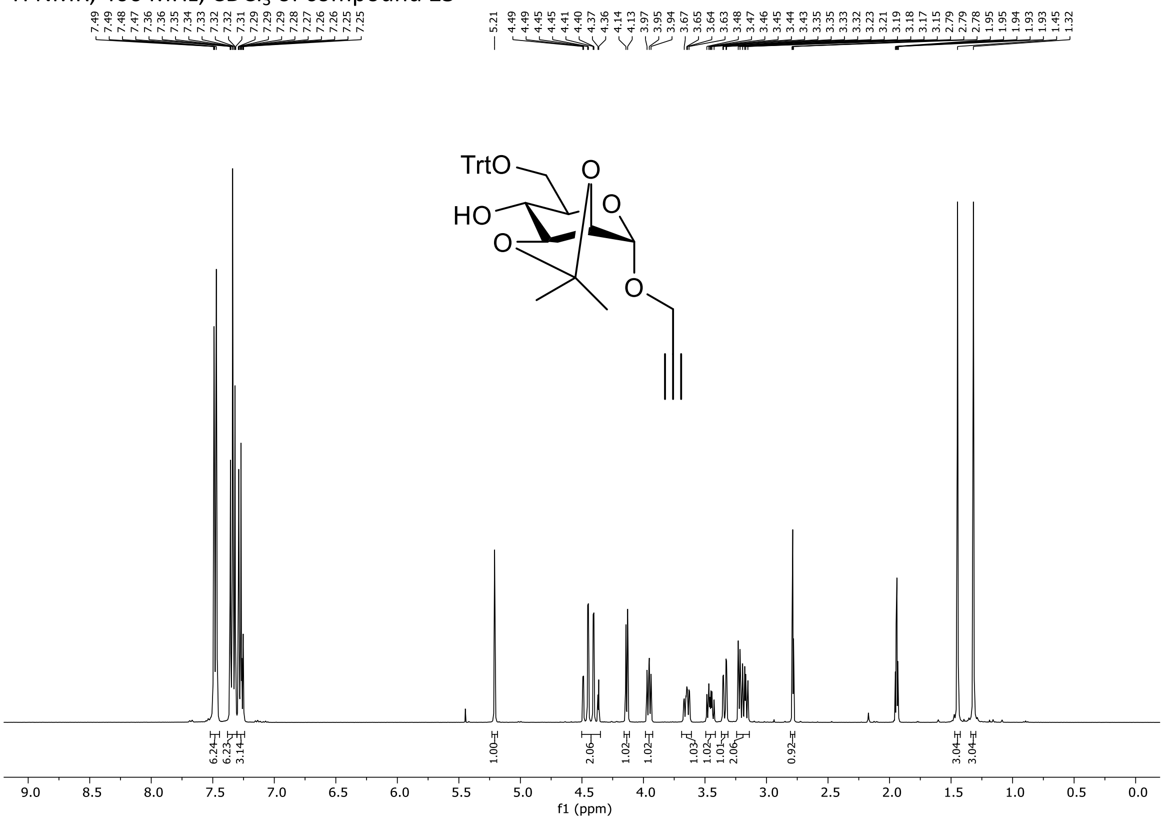

^{13}C-APT NMR, 101 MHz, $CDCl_3$ of compound **13**

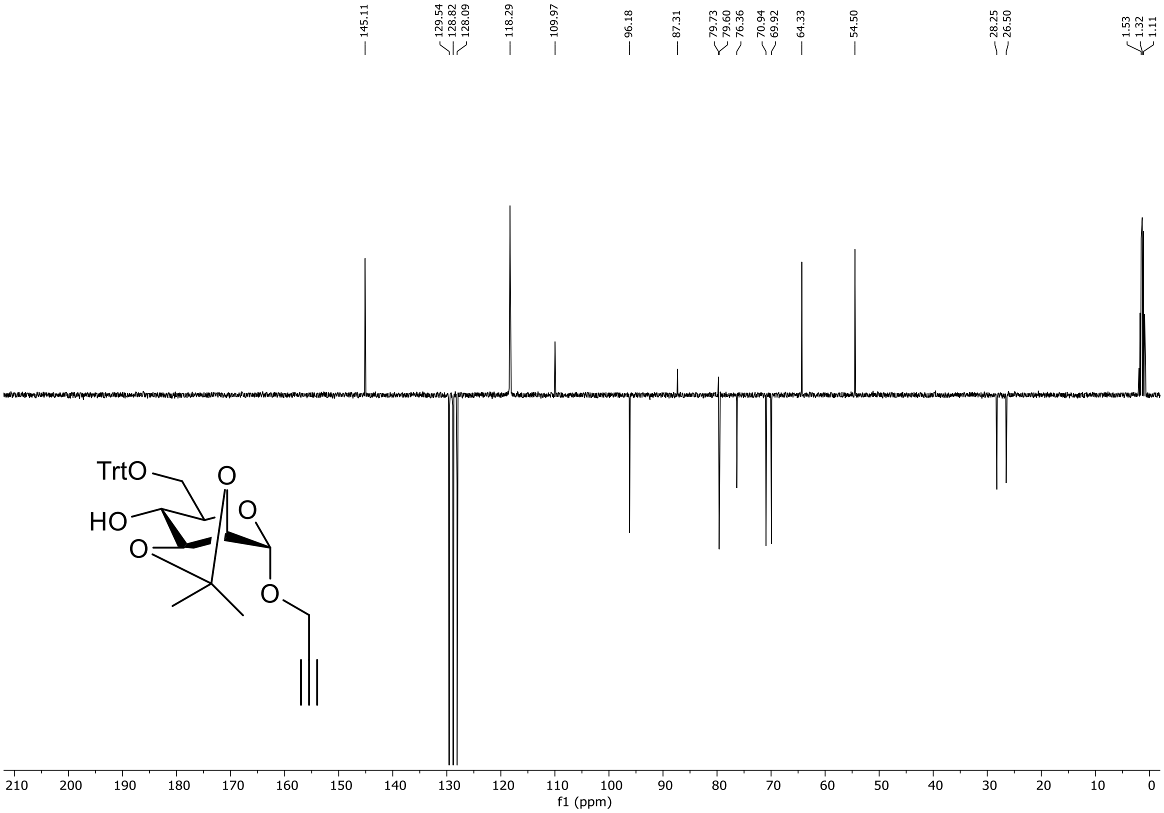

^{1}H-^{1}H COSY of compound **13**

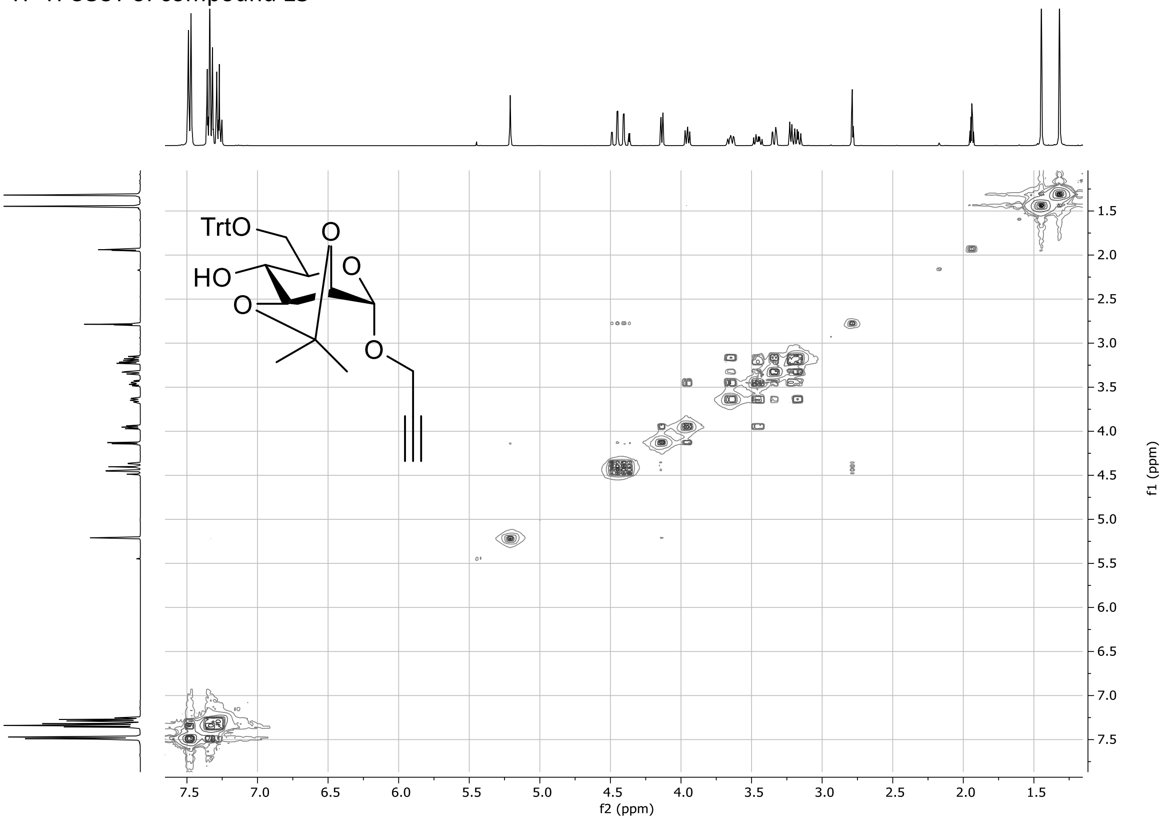

^{1}H-^{13}C HSQC of compound **13**

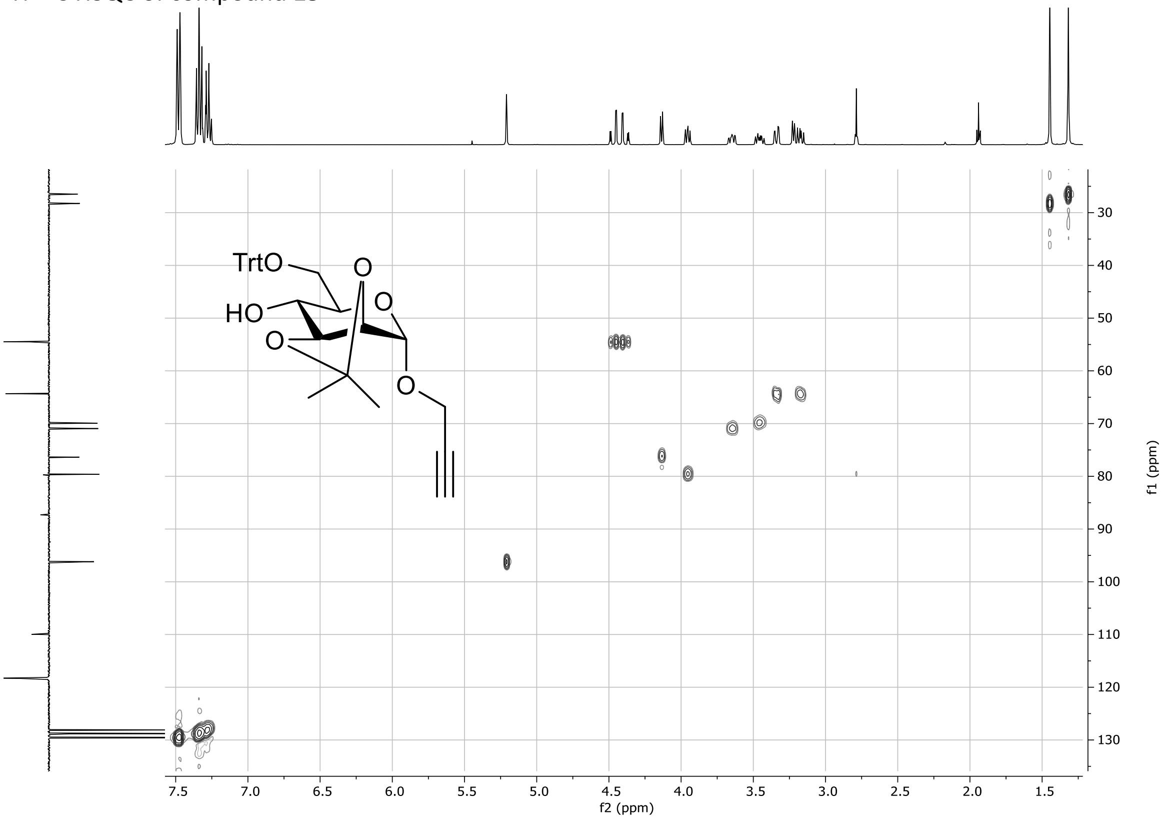

^{1}H NMR, 400 MHz, $CDCl_3$ of compound **14**

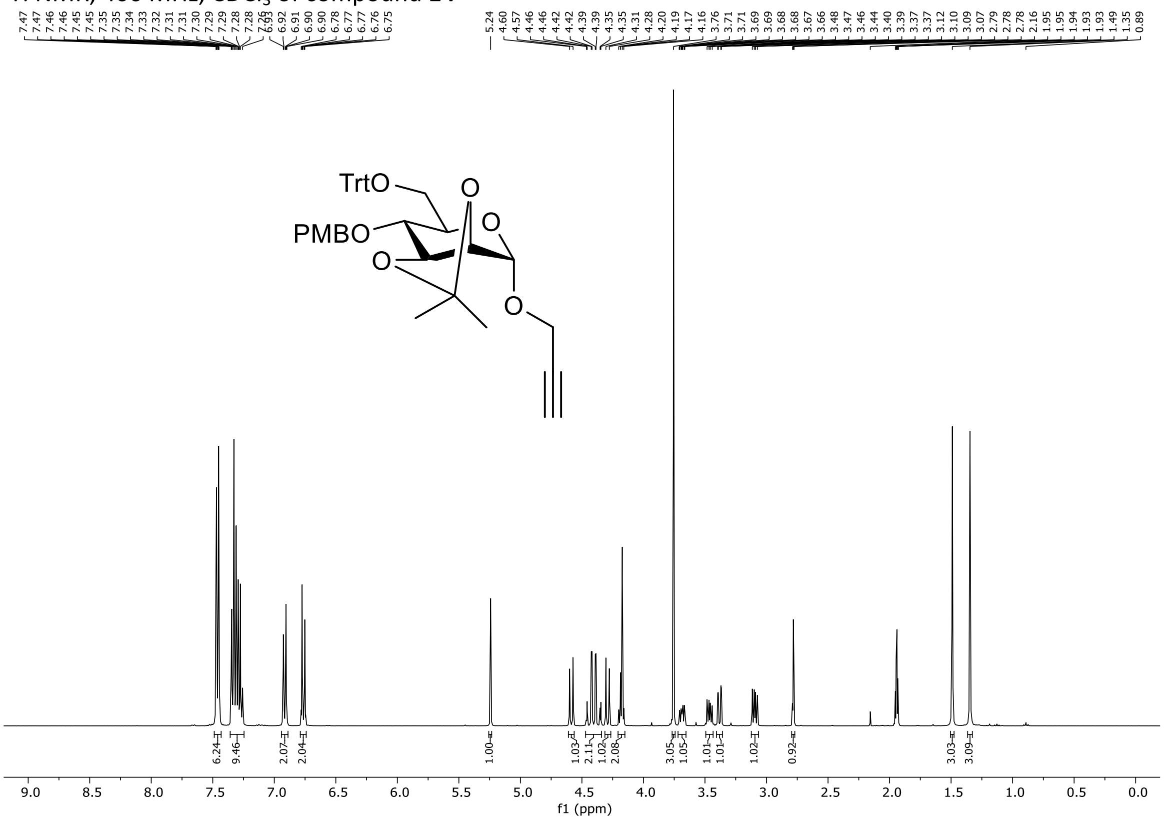

^{13}C-APT NMR, 101 MHz, $CDCl_3$ of compound **14**

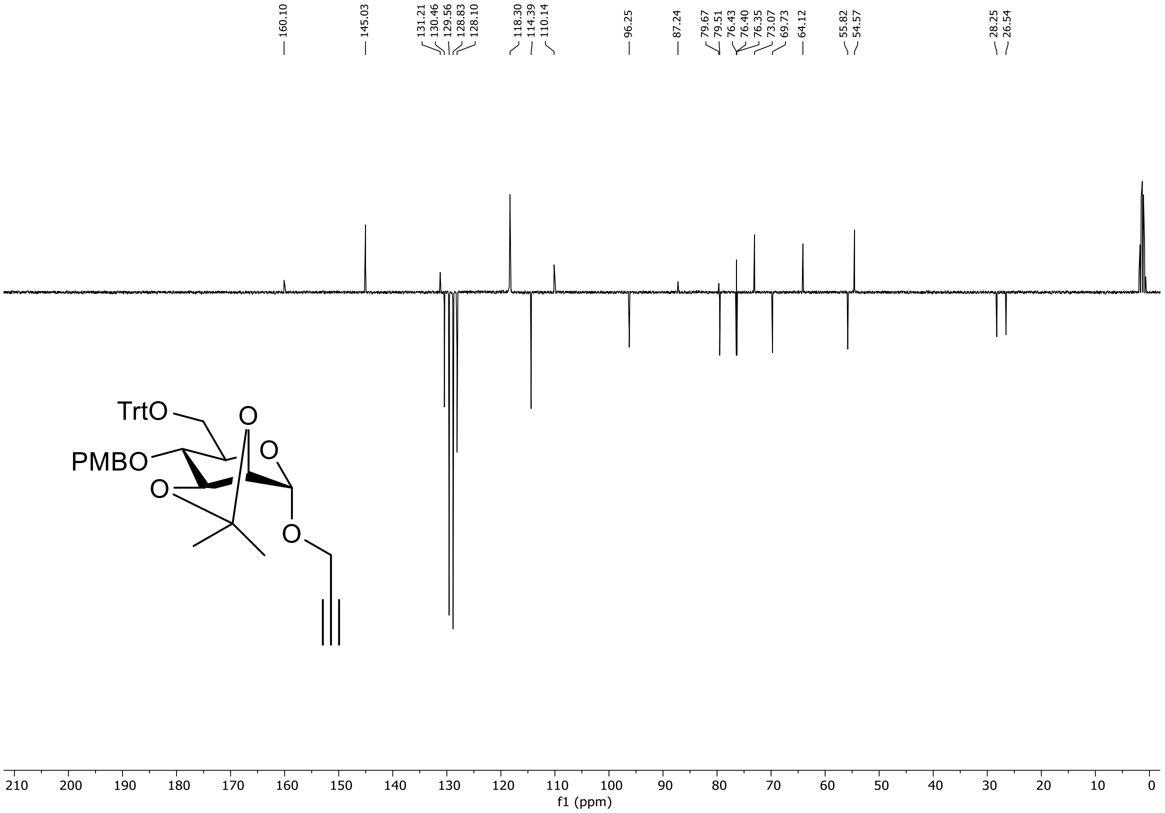

^{1}H-^{1}H COSY of compound **14**

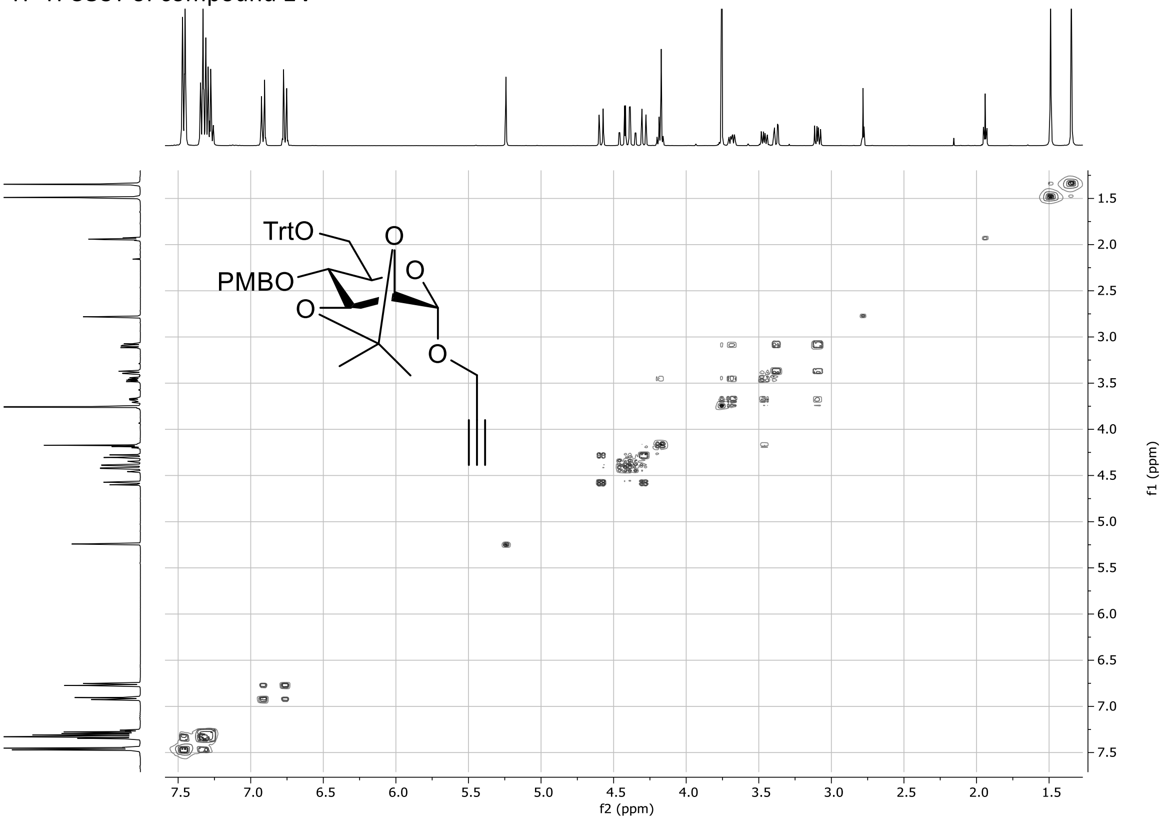

^{1}H-^{13}C HSQC of compound **14**

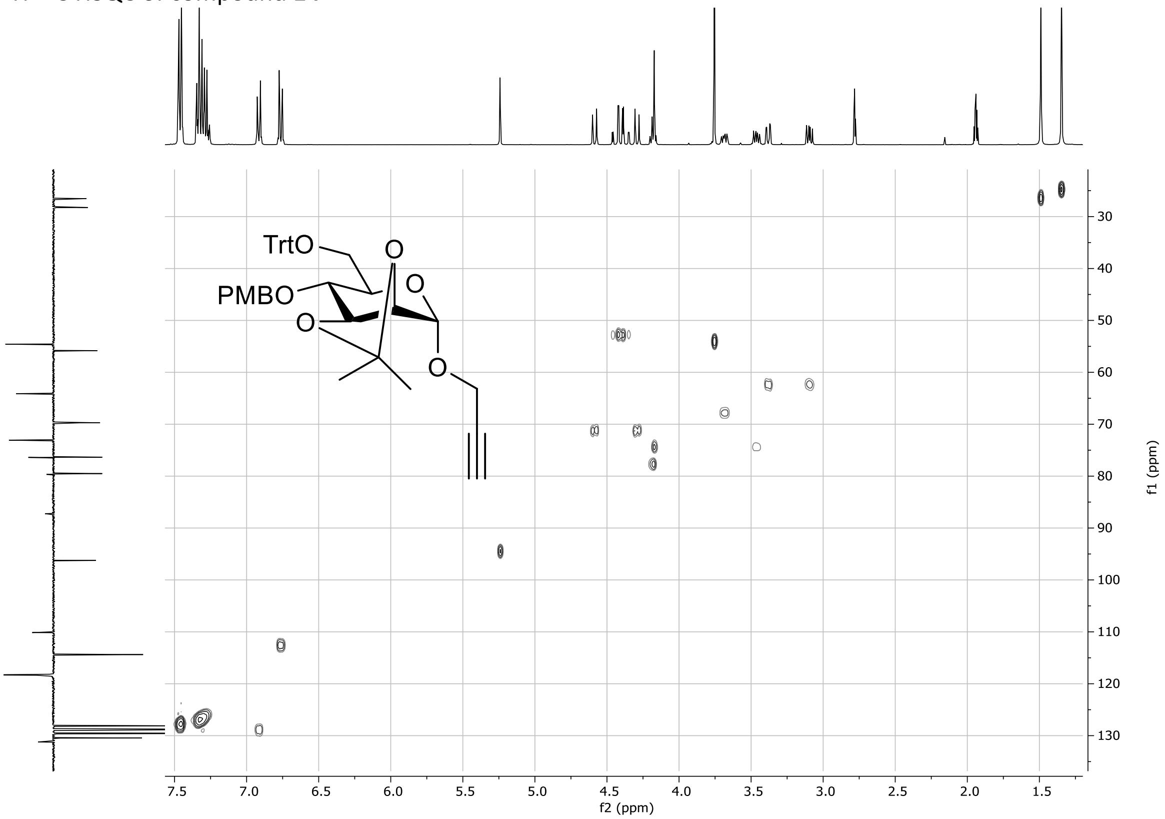

^{1}H NMR, 400 MHz, $CDCl_3$ of compound **15**

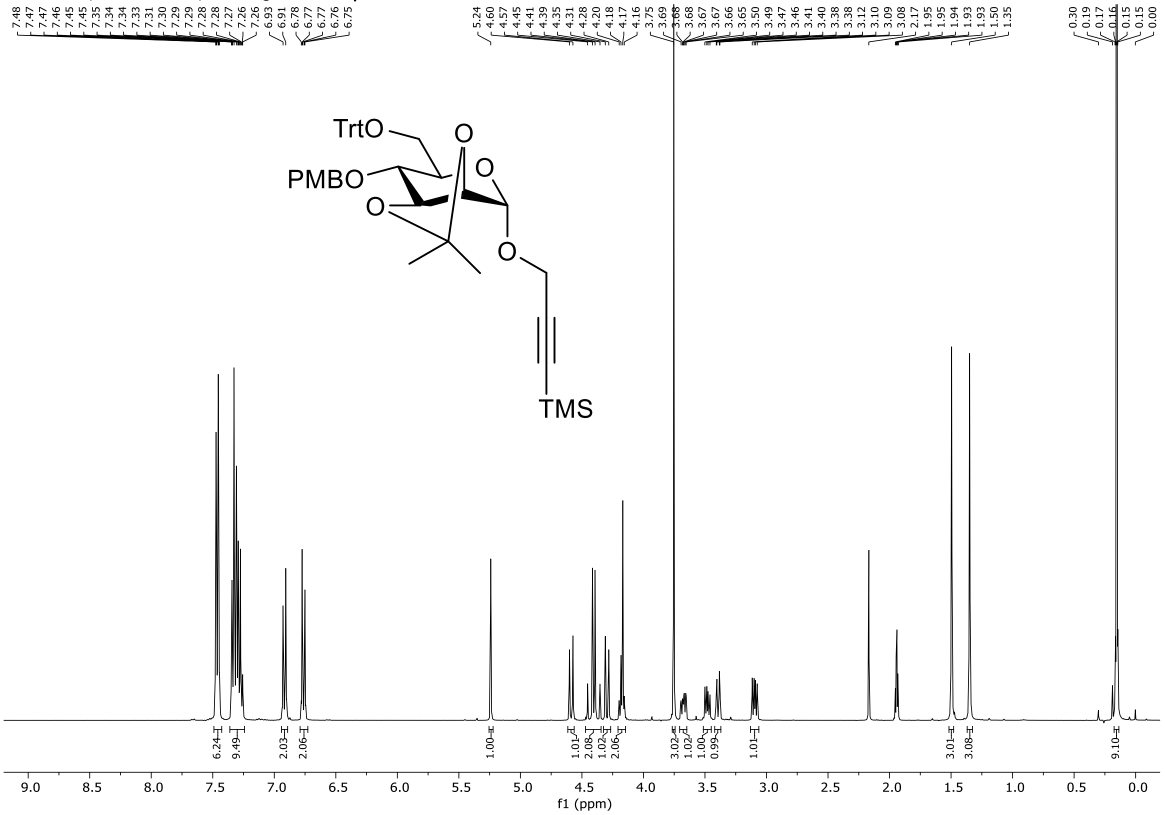

^{13}C-APT NMR, 101 MHz, $CDCl_3$ of compound **15**

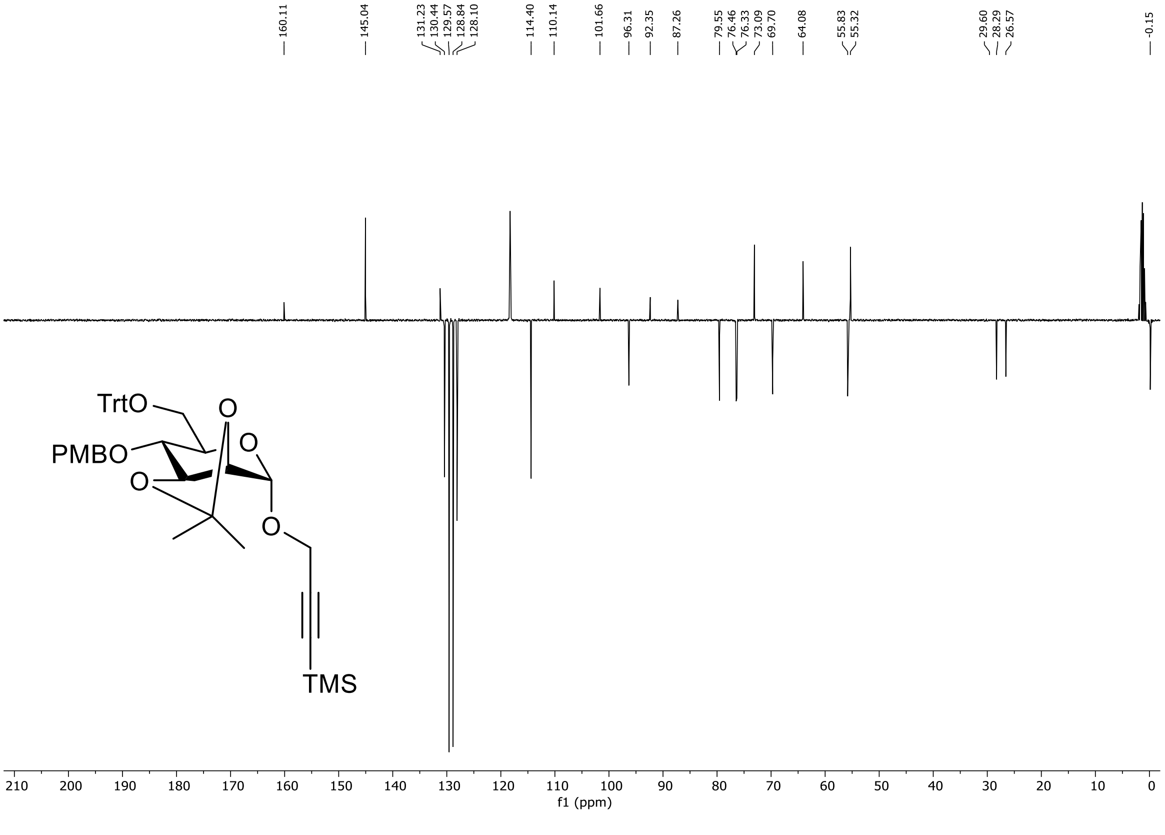

^{1}H-^{1}H COSY of compound **15**

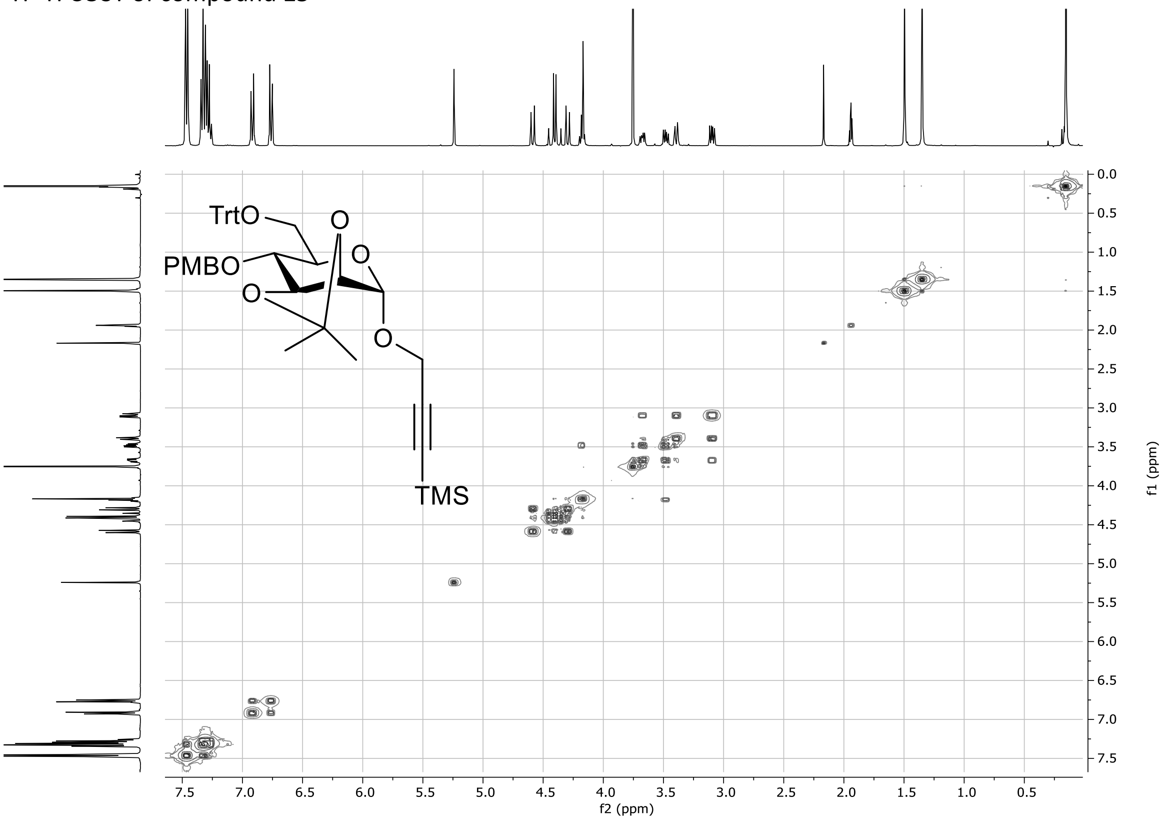

^{1}H-^{13}C HSQC of compound **15**

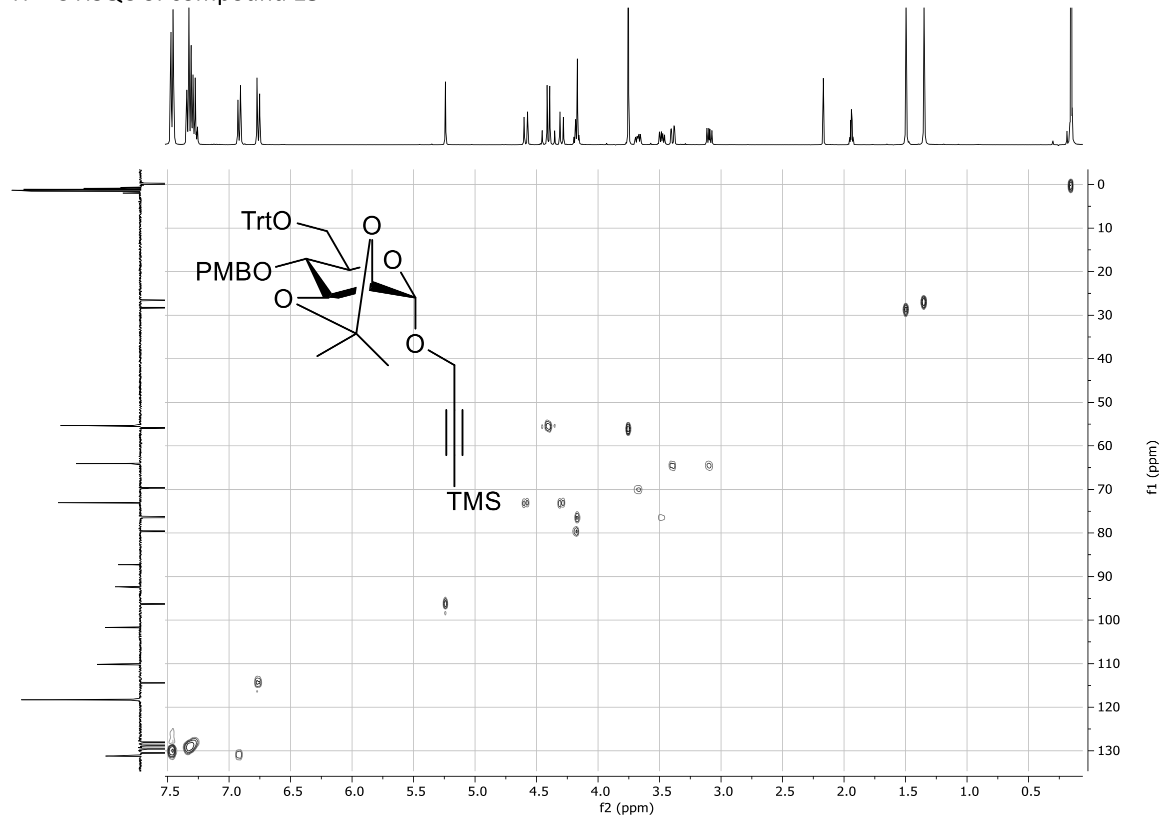

^{1}H NMR, 400 MHz, $CDCl_3$ of compound **16**

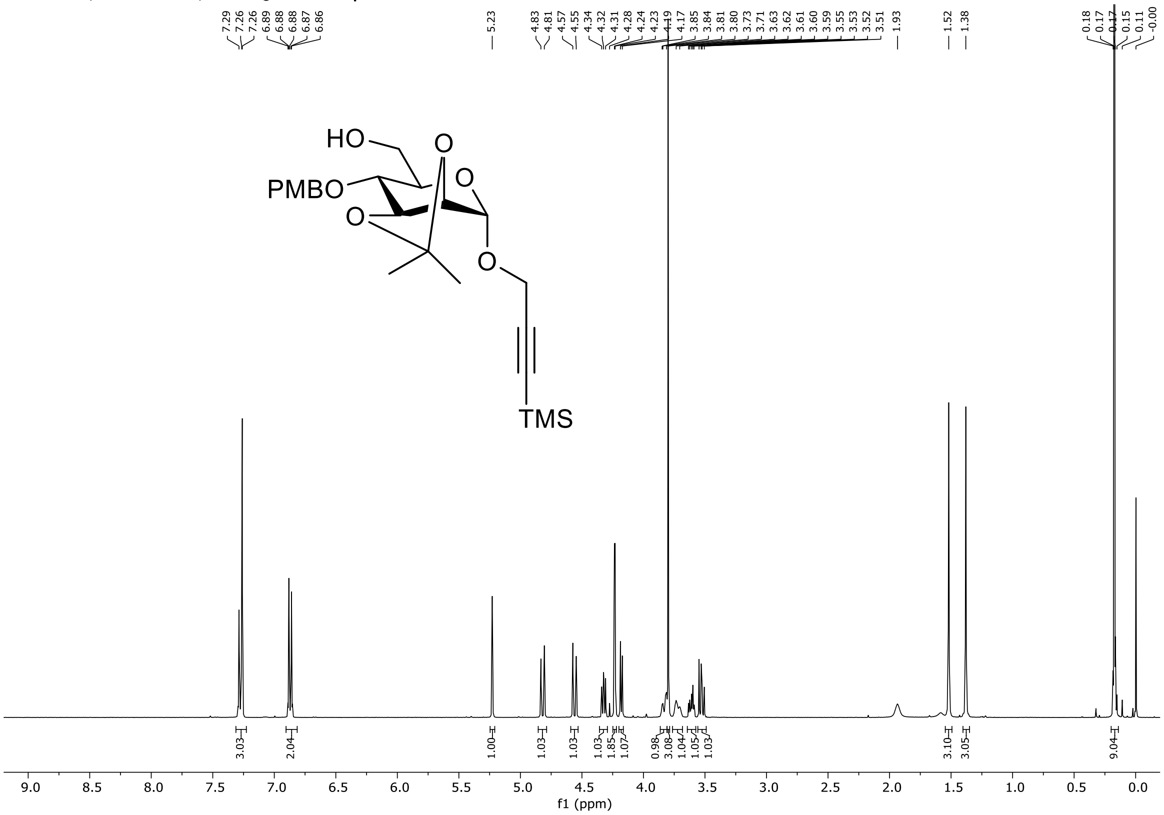

^{13}C-APT NMR, 101 MHz, $CDCl_3$ of compound **16**

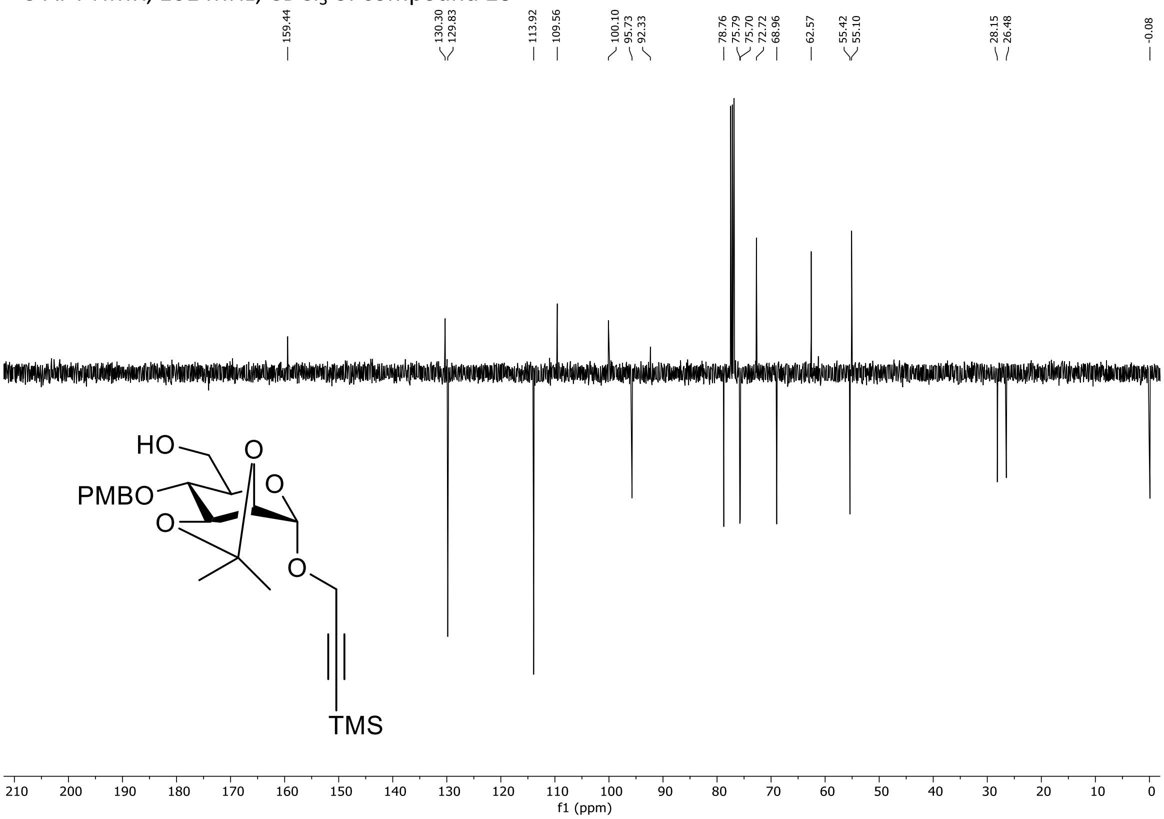

^{1}H-^{1}H COSY of compound **16**

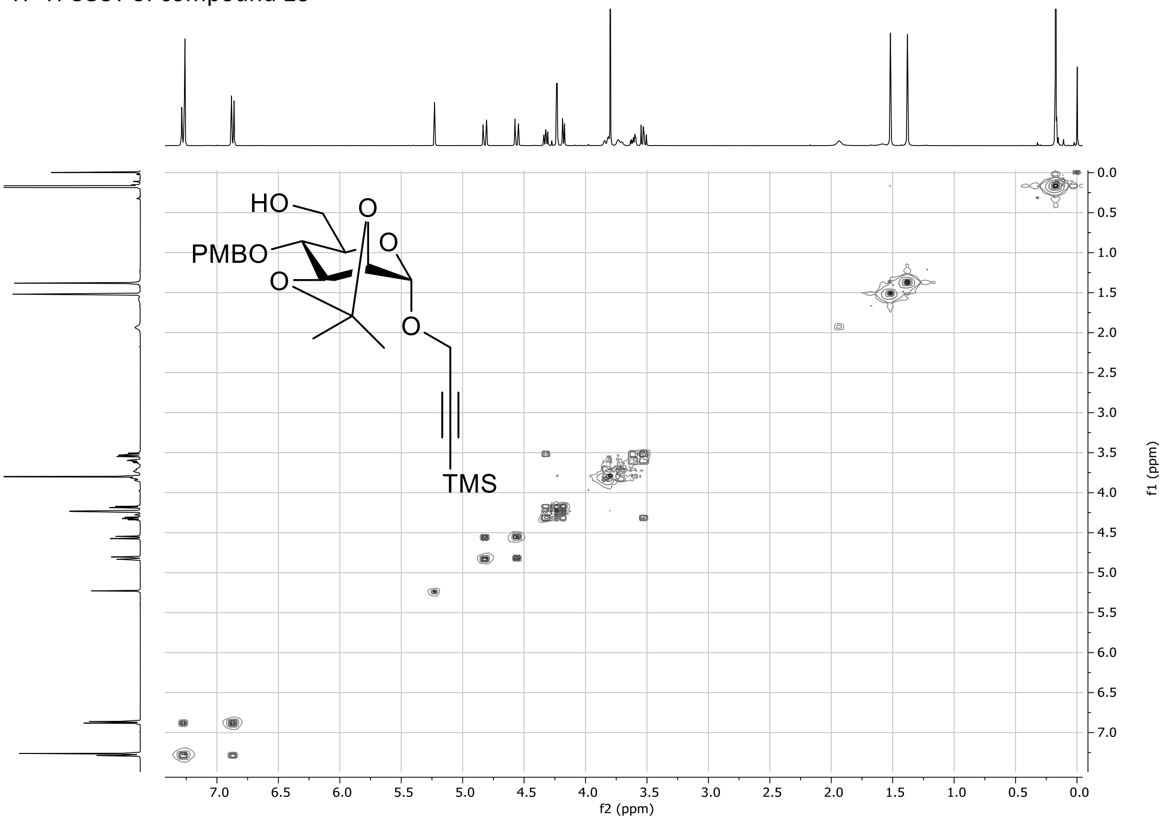

^{1}H-^{13}C HSQC of compound **16**

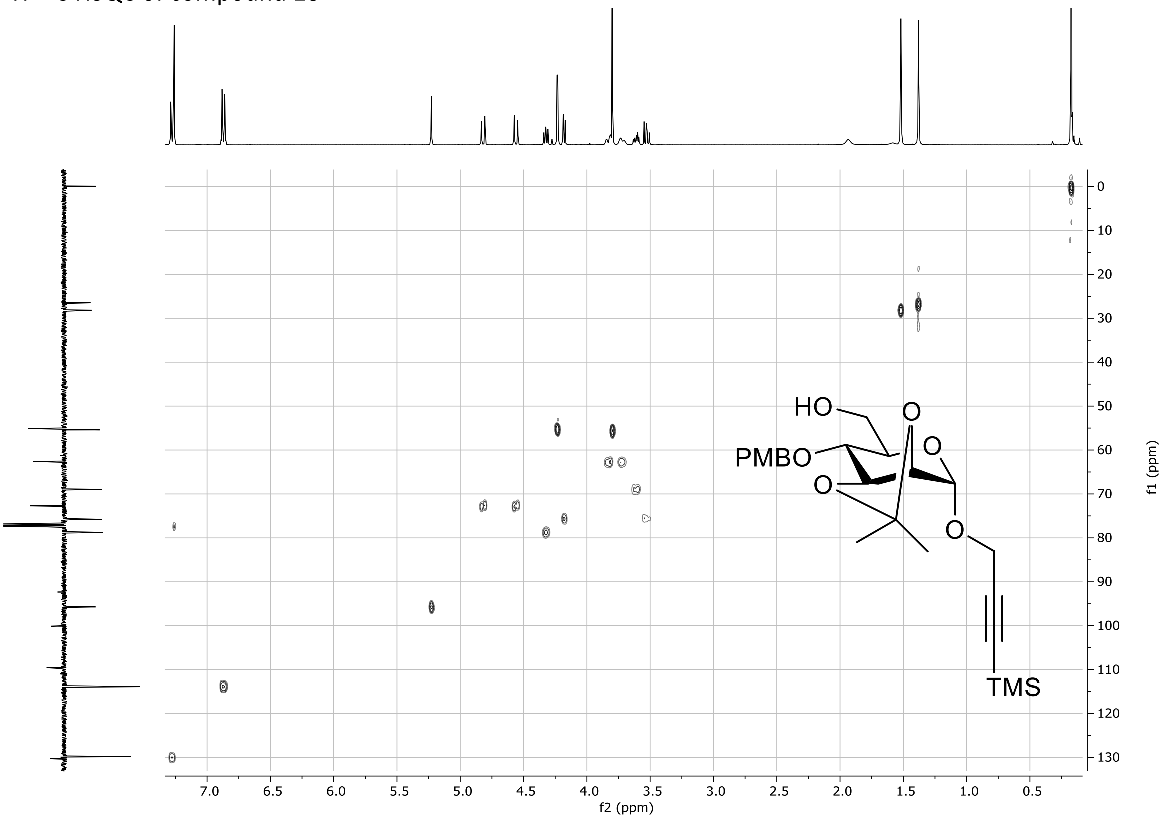

^{1}H NMR, 400 MHz, $CDCl_3$ of compound **17**

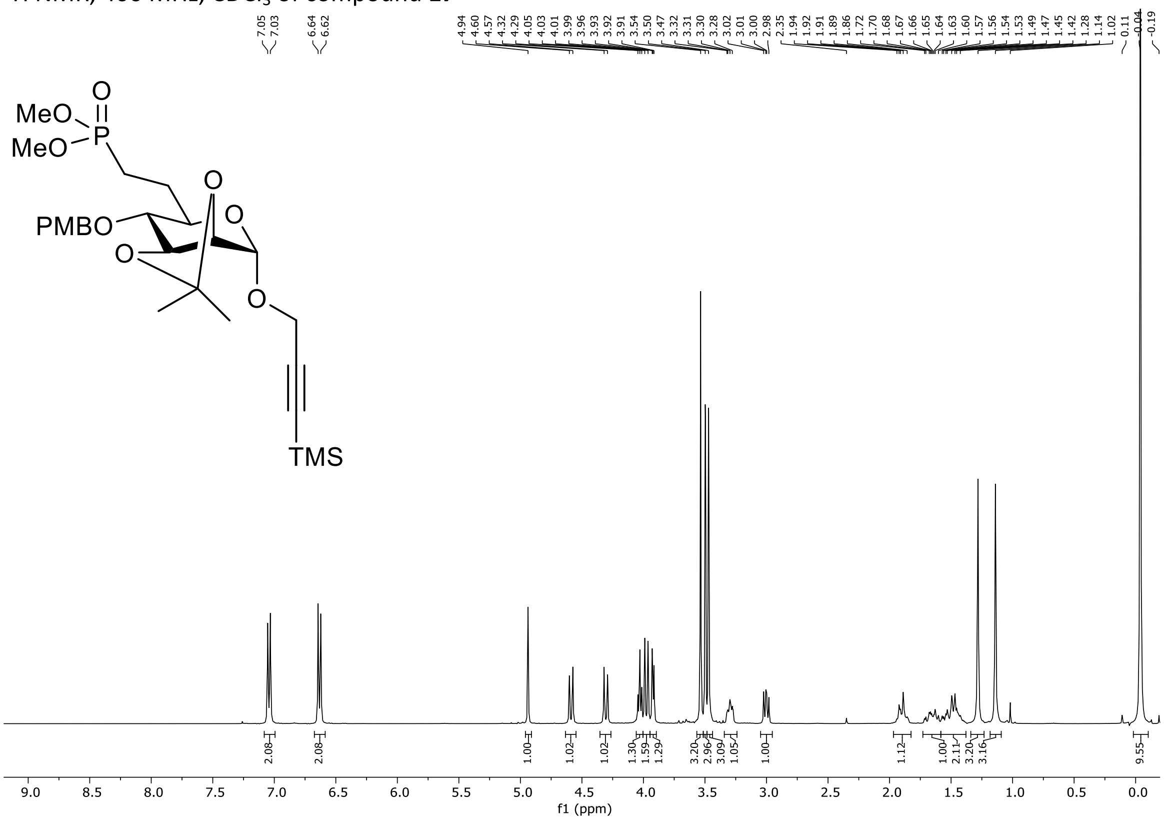

^{13}C-APT NMR, 101 MHz, $CDCl_3$ of compound **17**

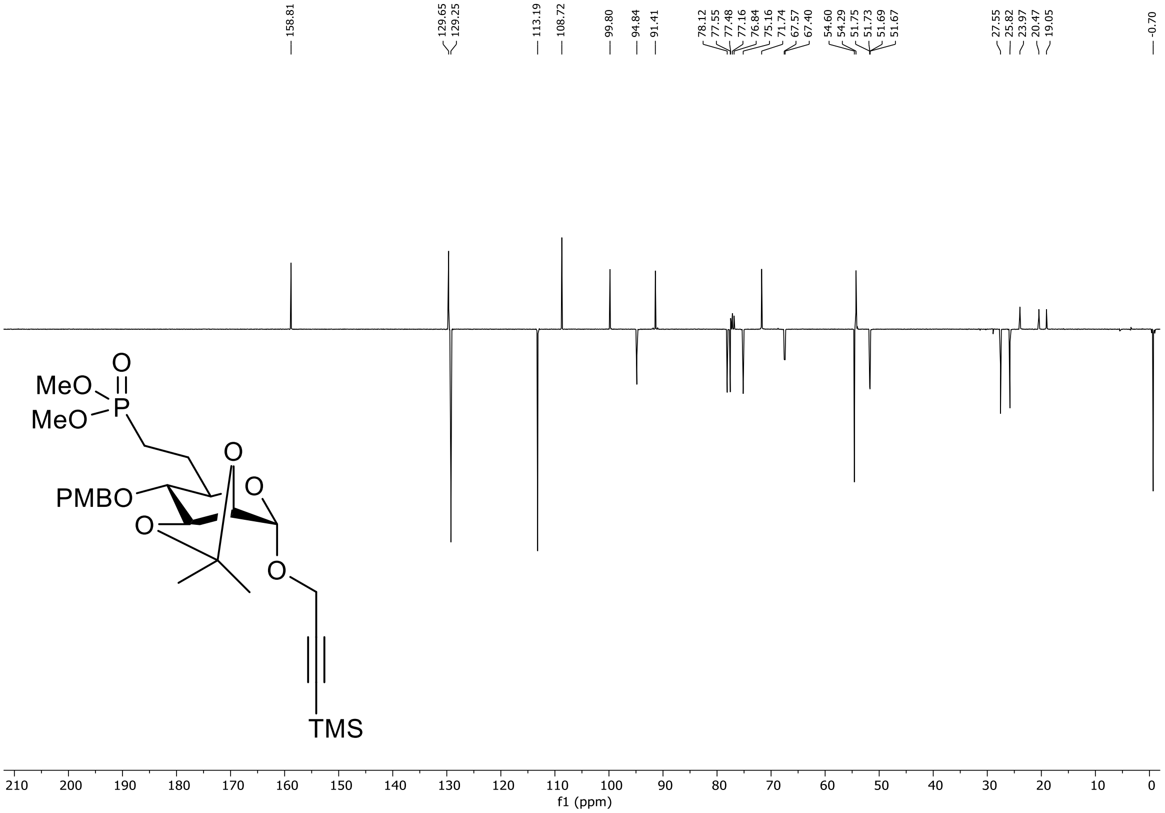

^{1}H-^{1}H COSY of compound **17**

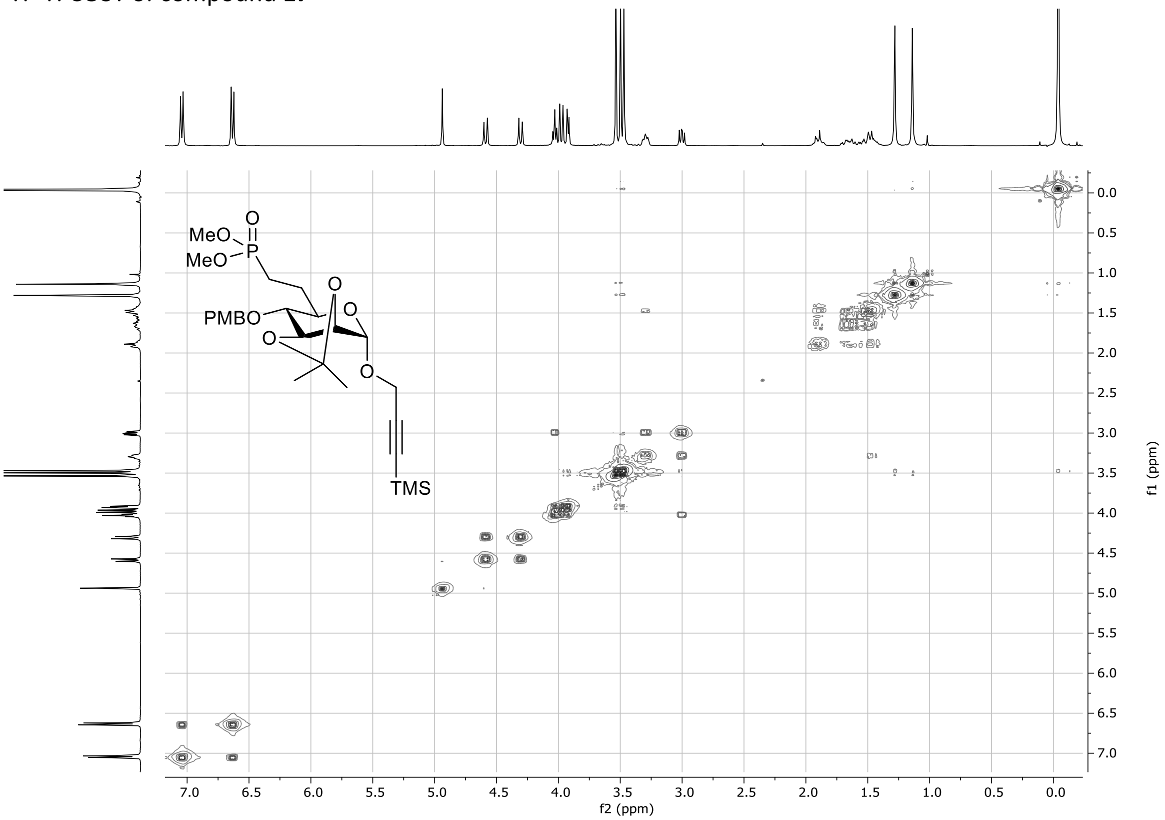

^{1}H-^{13}C HSQC of compound **17**

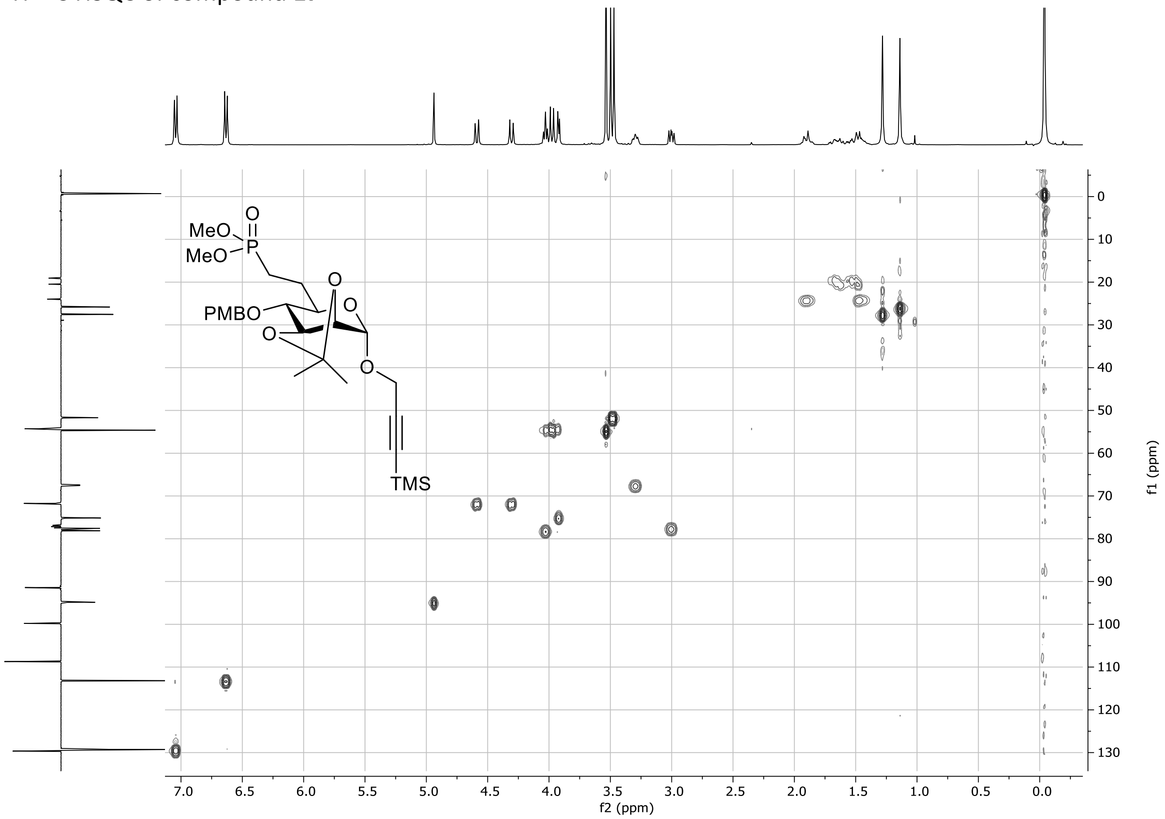

^{31}P NMR of compound **17**

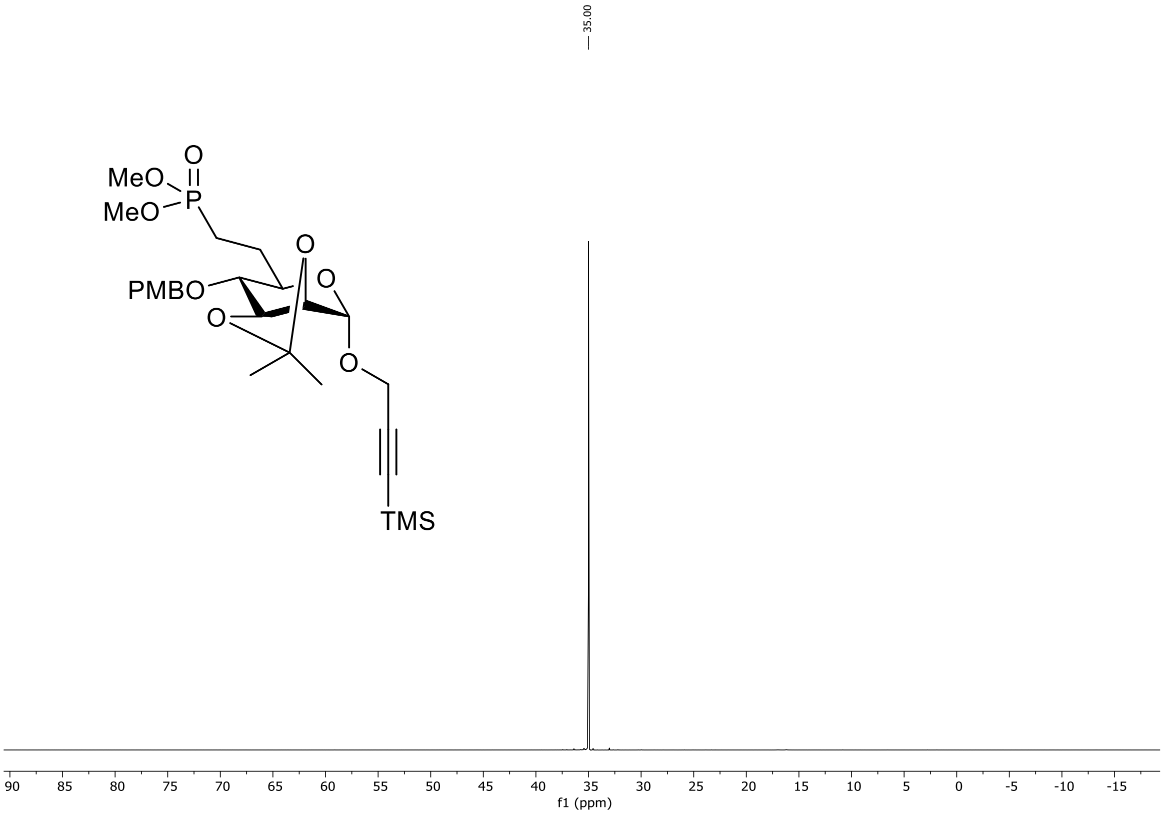

^{1}H NMR, 400 MHz, $CDCl_3$ of compound **18**

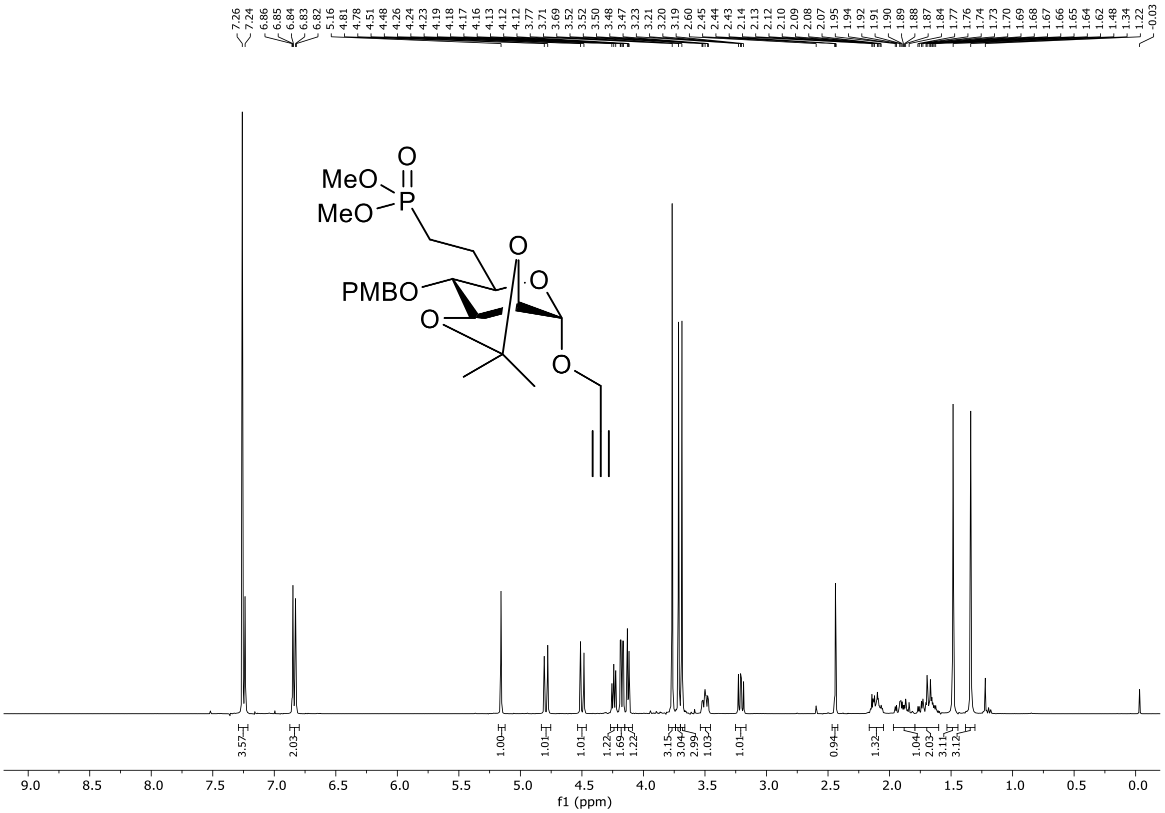

^{13}C-APT NMR, 101 MHz, $CDCl_3$ of compound **18**

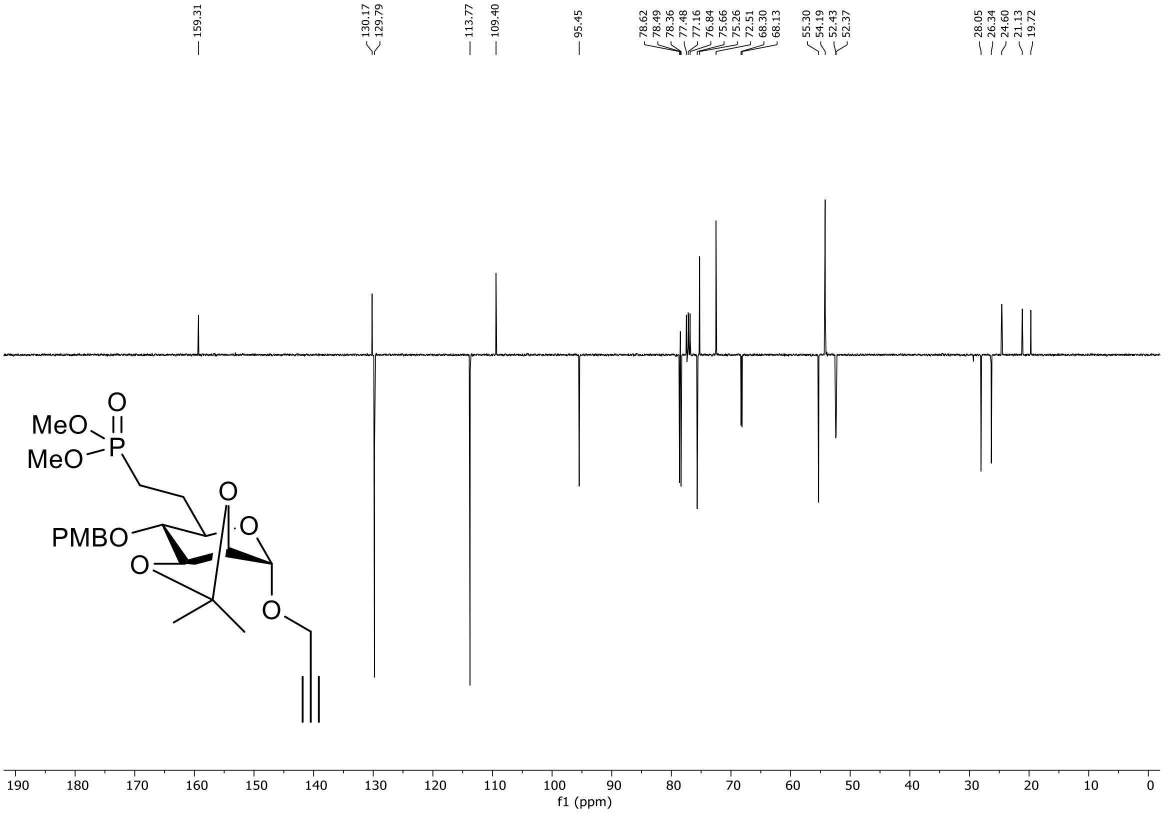

^{1}H-^{1}H COSY of compound **18**

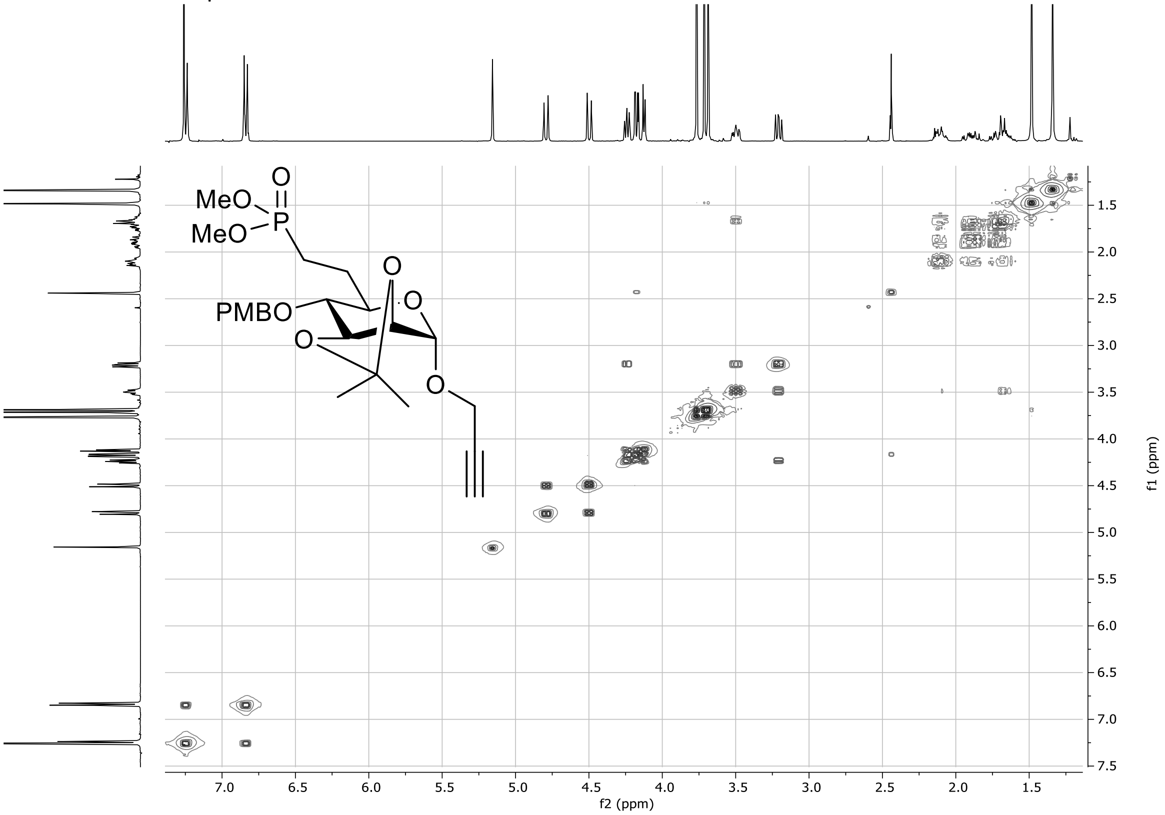

^{1}H-^{13}C HSQC of compound **18**

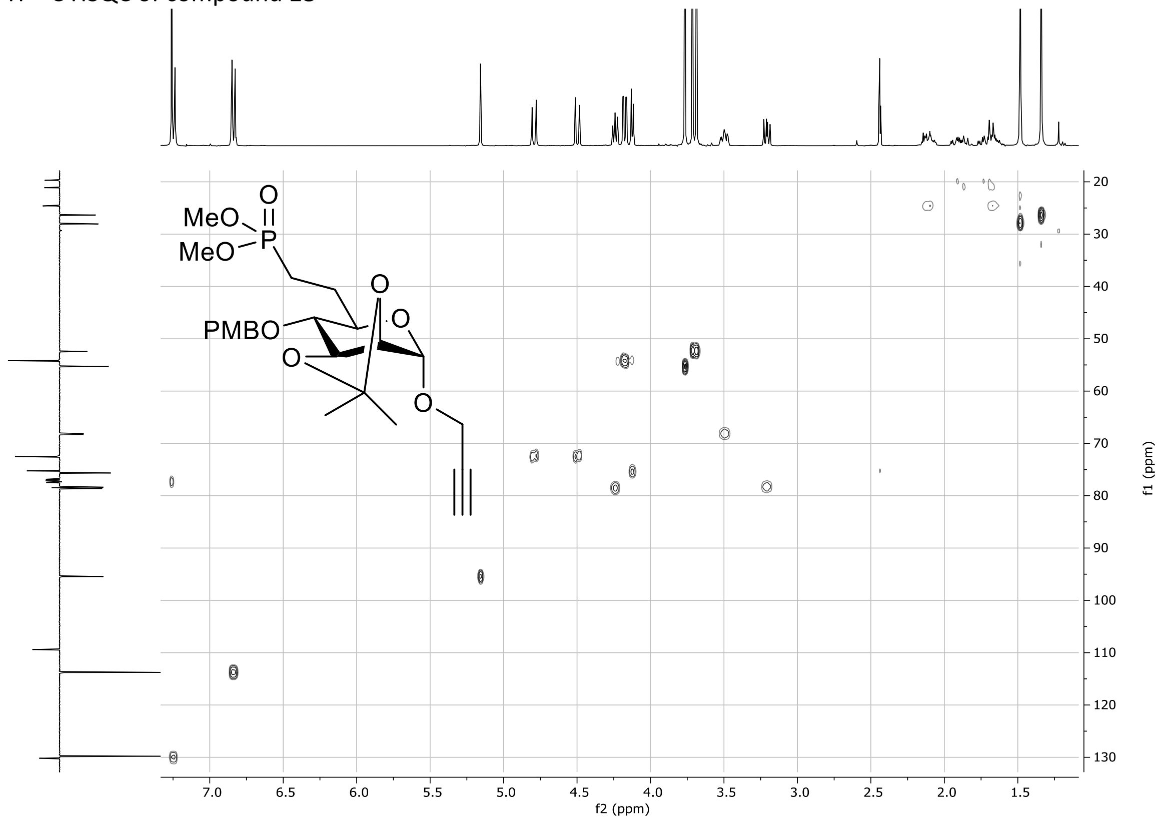

^{31}P NMR of compound **18**

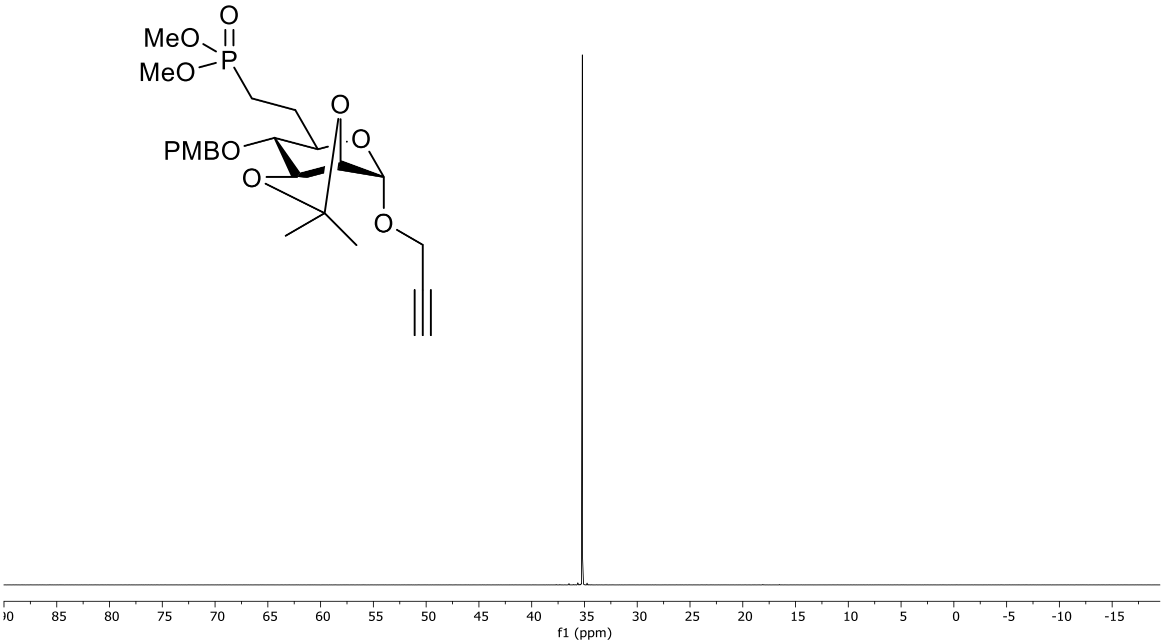

^{1}H NMR, 400 MHz, $CDCl_3$ of compound **9**

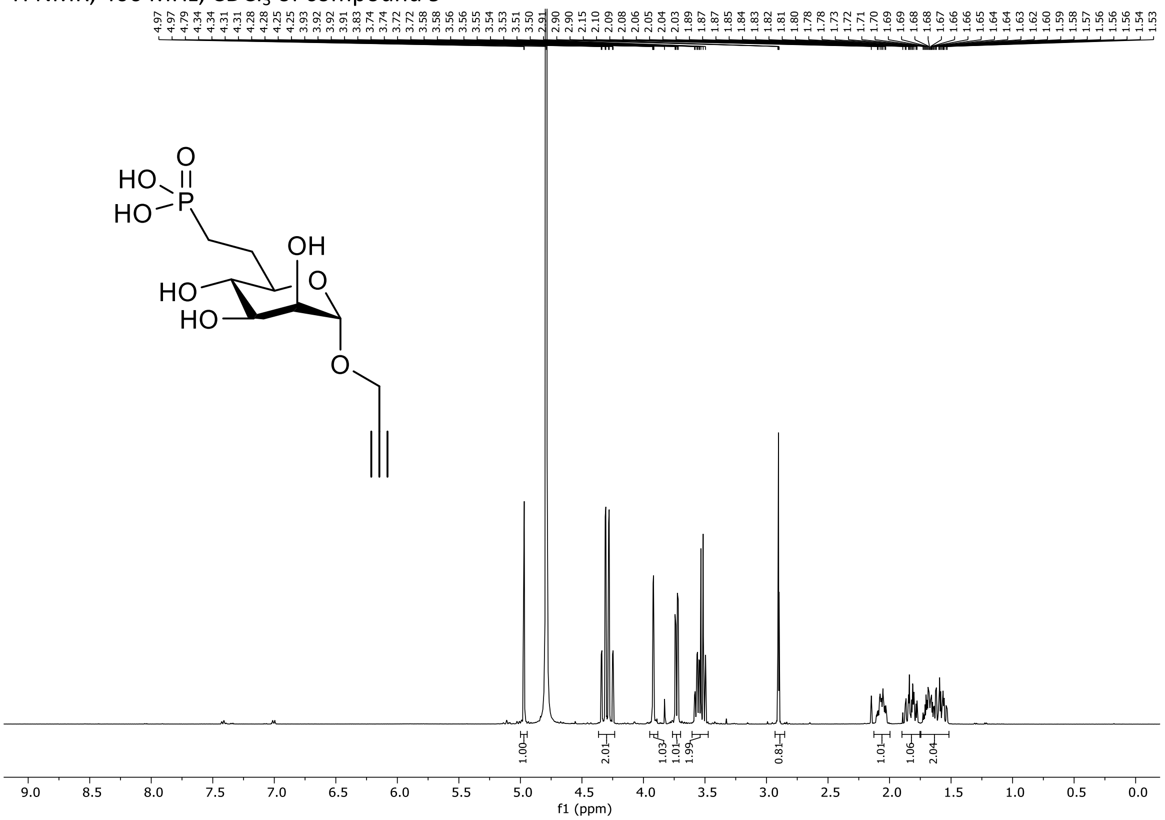

^{13}C-APT NMR, 101 MHz, $CDCl_3$ of compound **9**

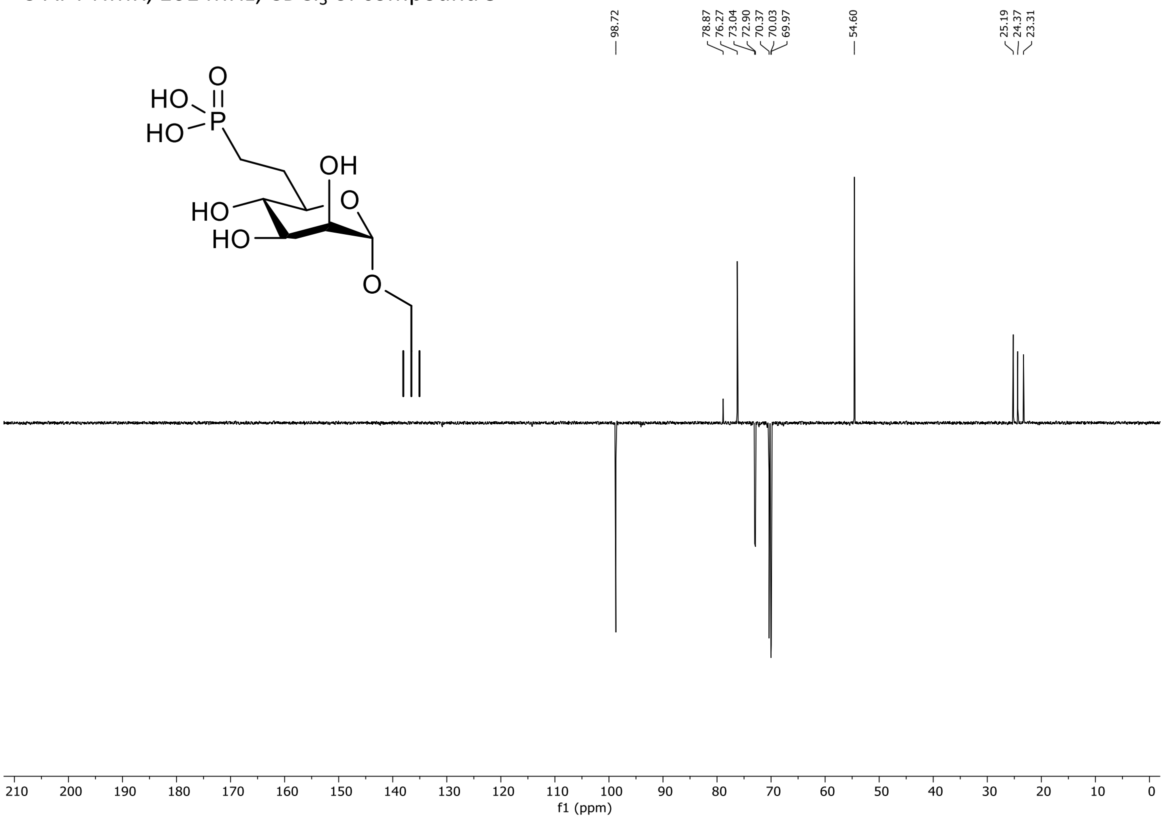

^1H-^1H COSY of compound **9**

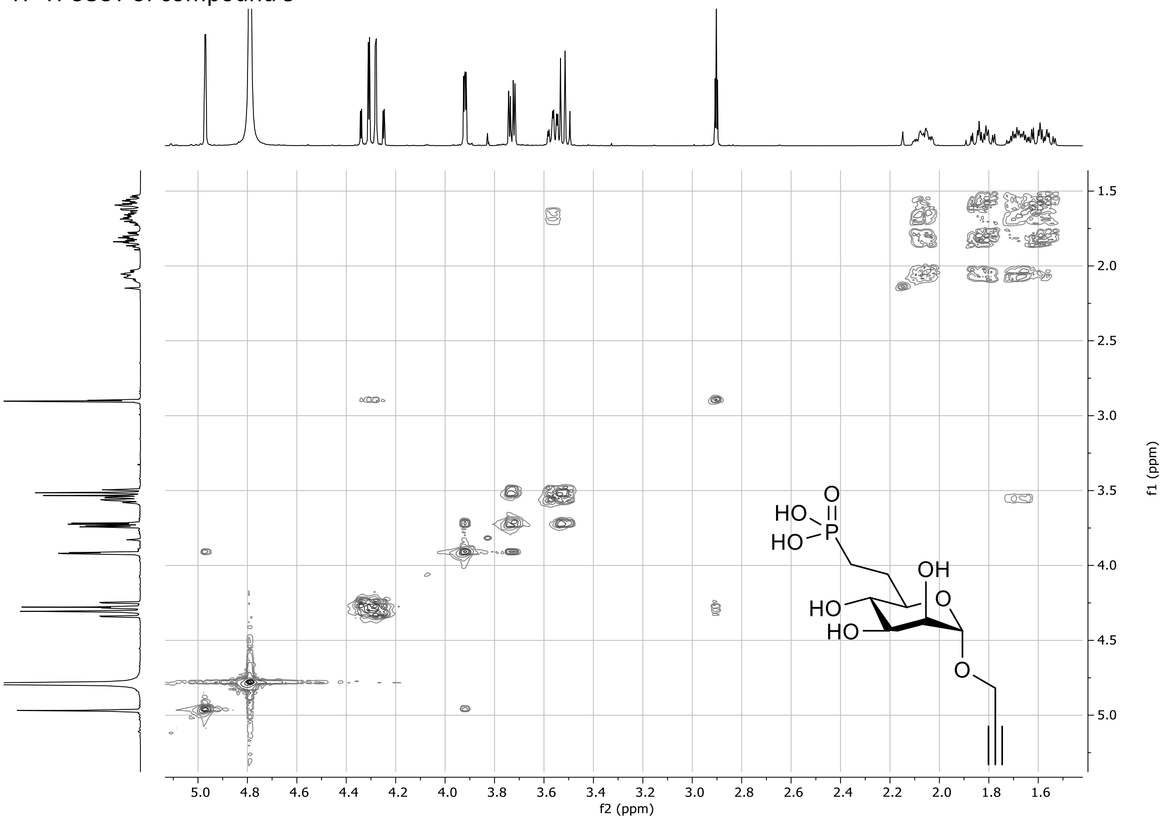

^1H-^{13}C HSQC of compound **9**

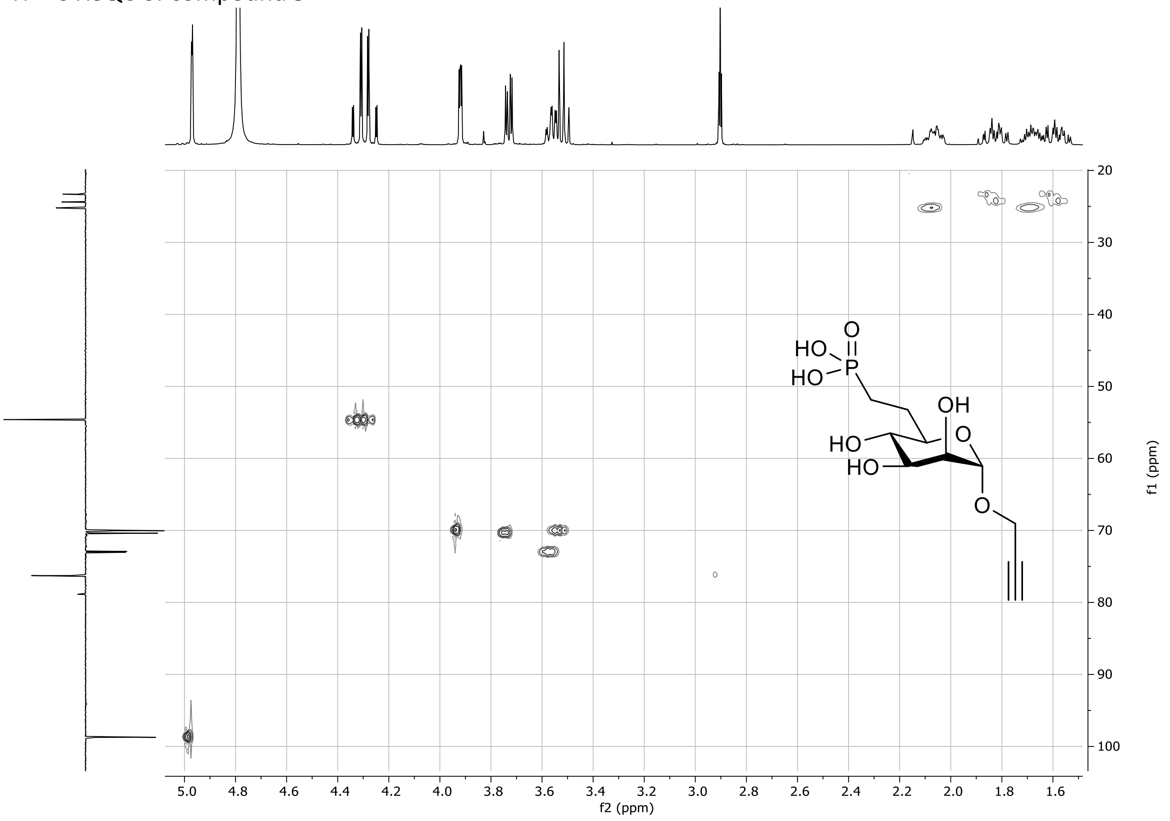

^{31}P NMR of compound **9**

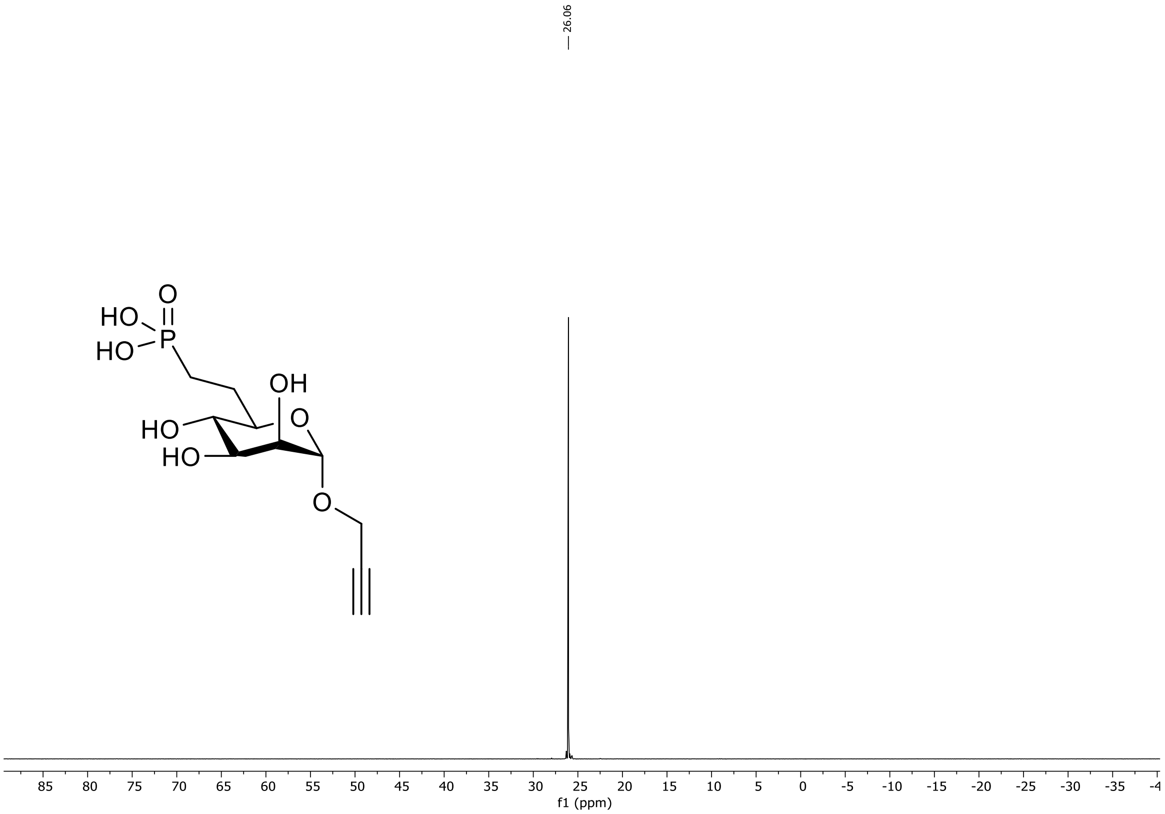

^{1}H NMR, 400 MHz, $CDCl_3$ of compound **20**

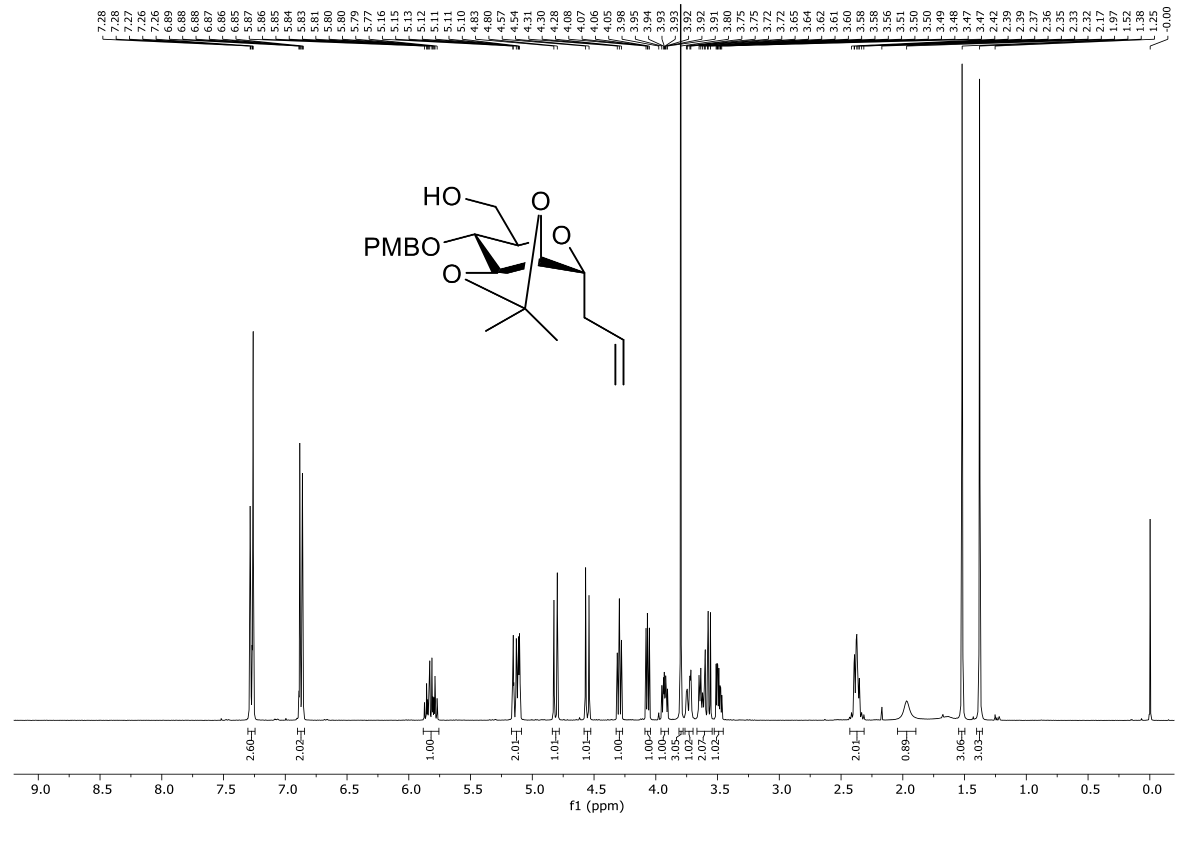

^{13}C-APT NMR, 101 MHz, $CDCl_3$ of compound **20**

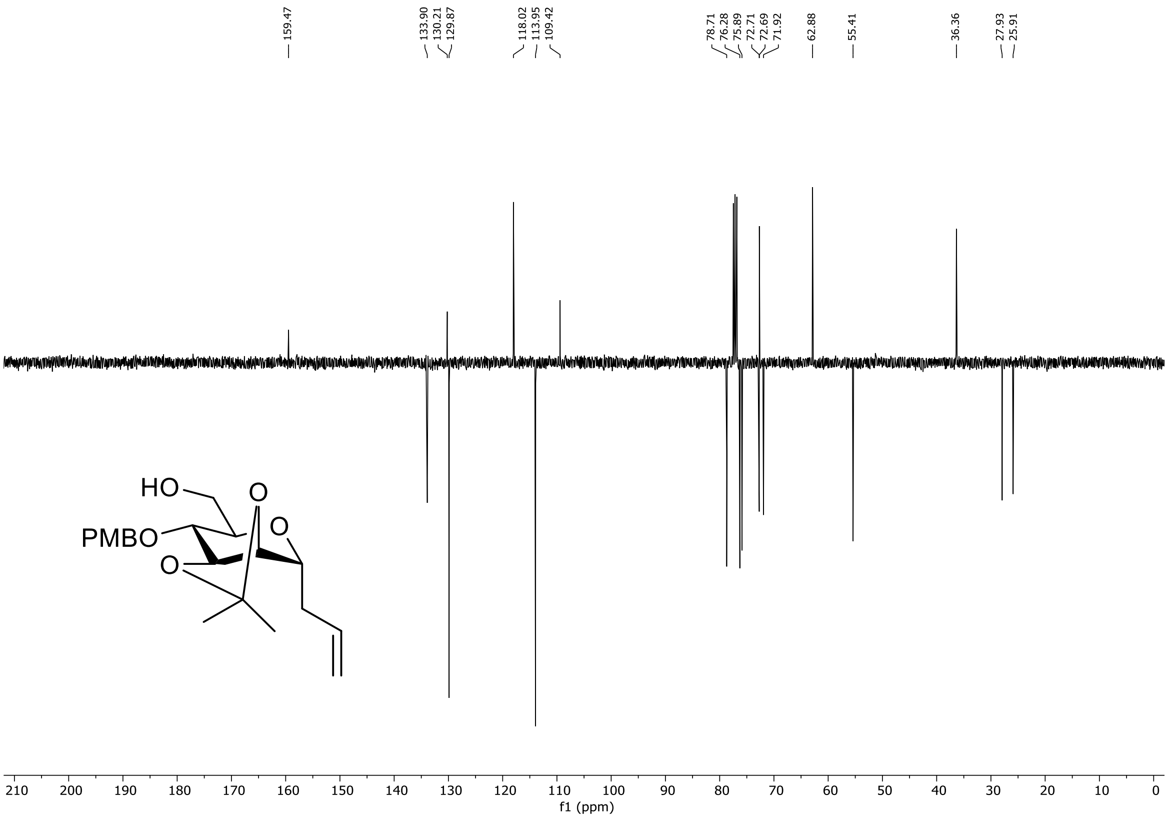

^{1}H-^{1}H COSY of compound **20**

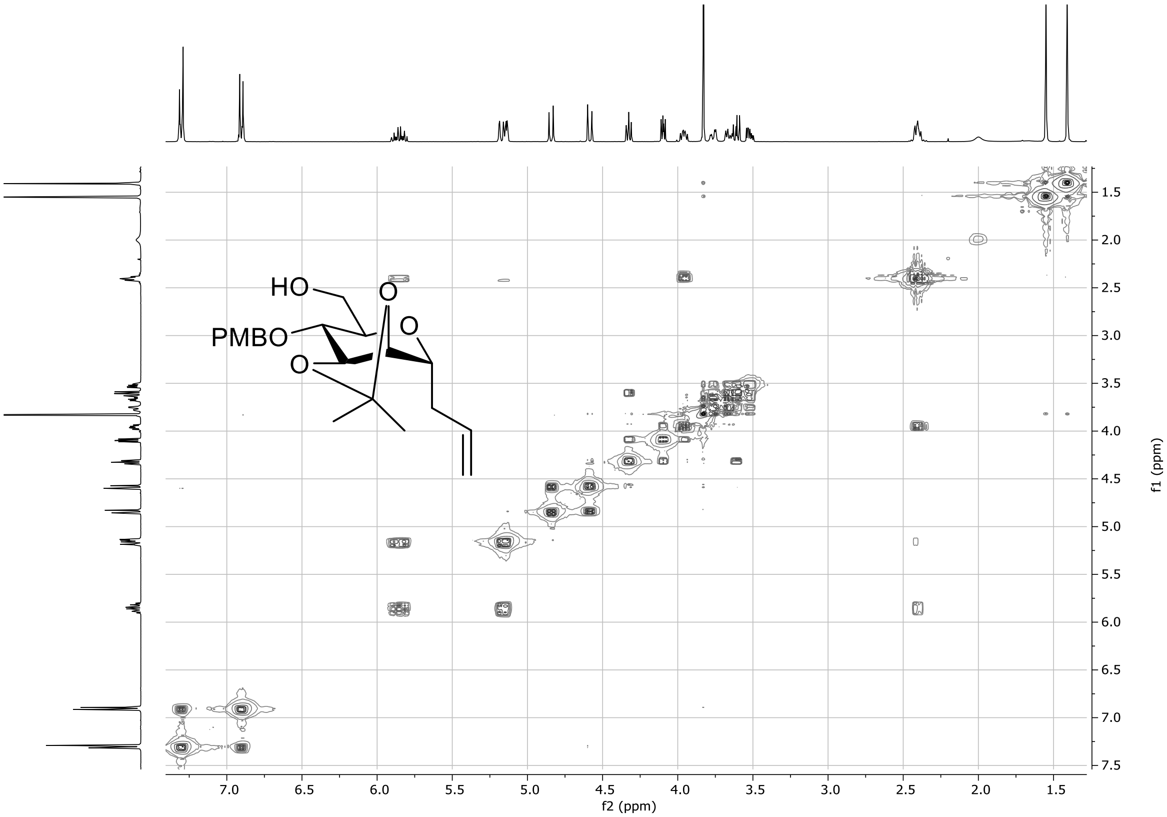

^{1}H-^{13}C HSQC of compound **20**

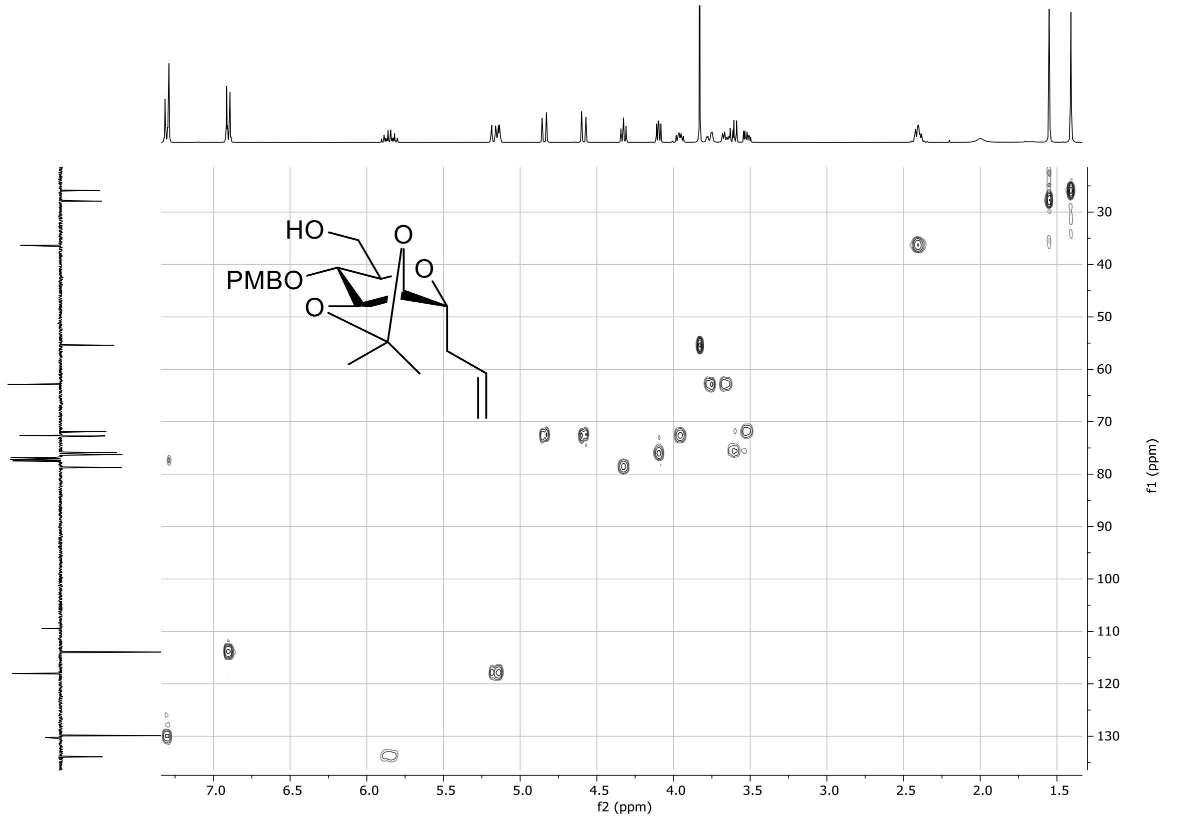

^{1}H NMR, 400 MHz, $CDCl_3$ of compound **S4**

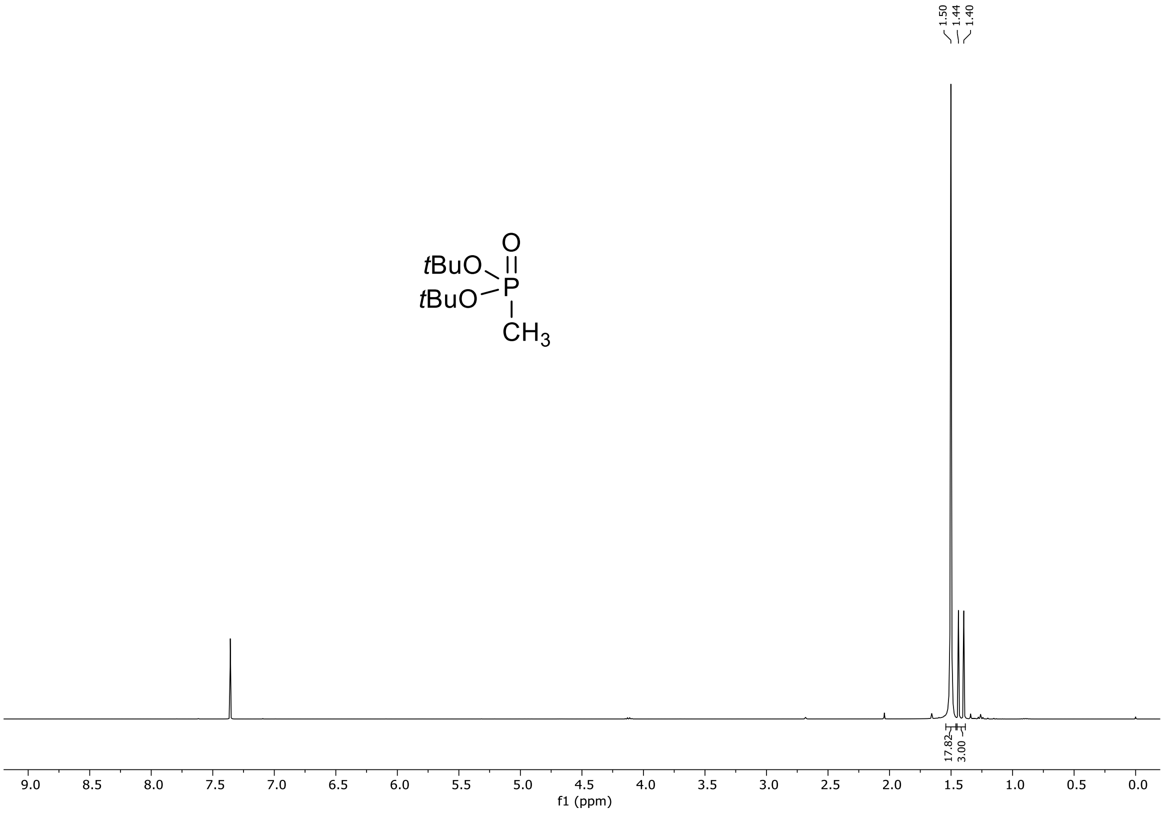

^{13}C-APT NMR, 101 MHz, $CDCl_3$ of compound **S4**

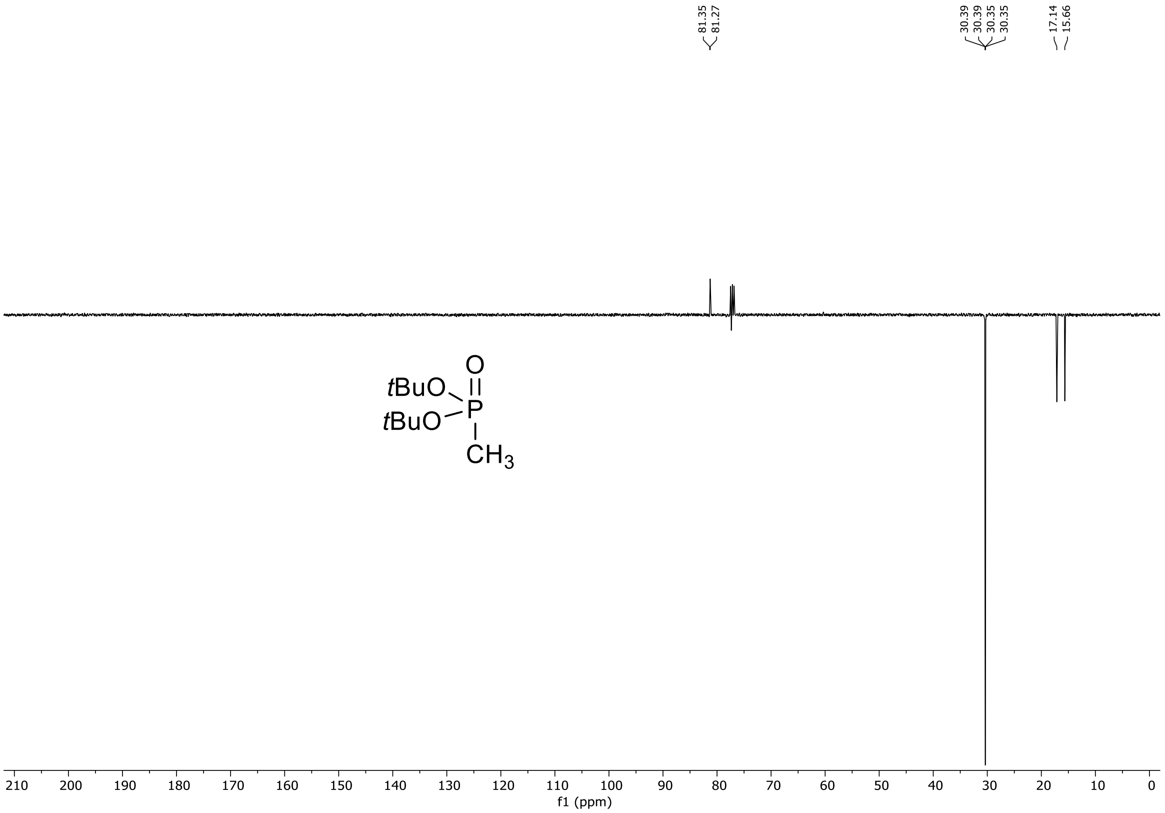

^{1}H-^{13}C HSQC of compound **S4**

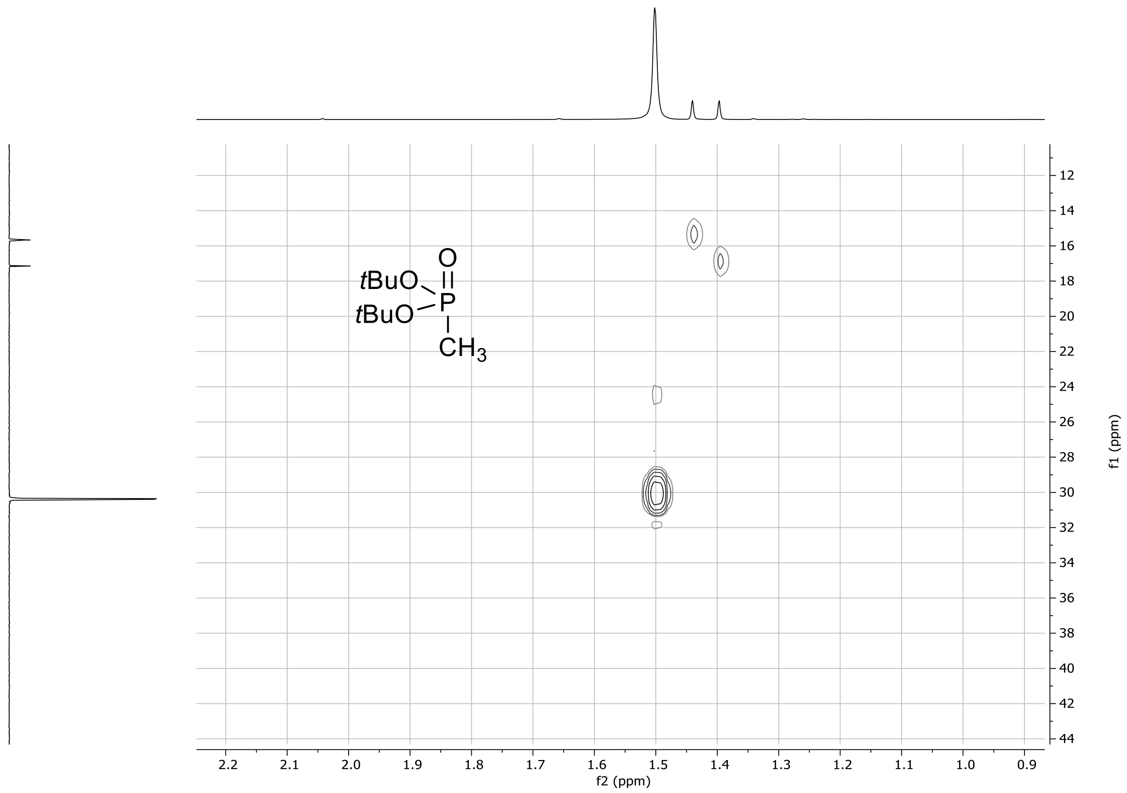

^{31}P NMR of compound **S4**

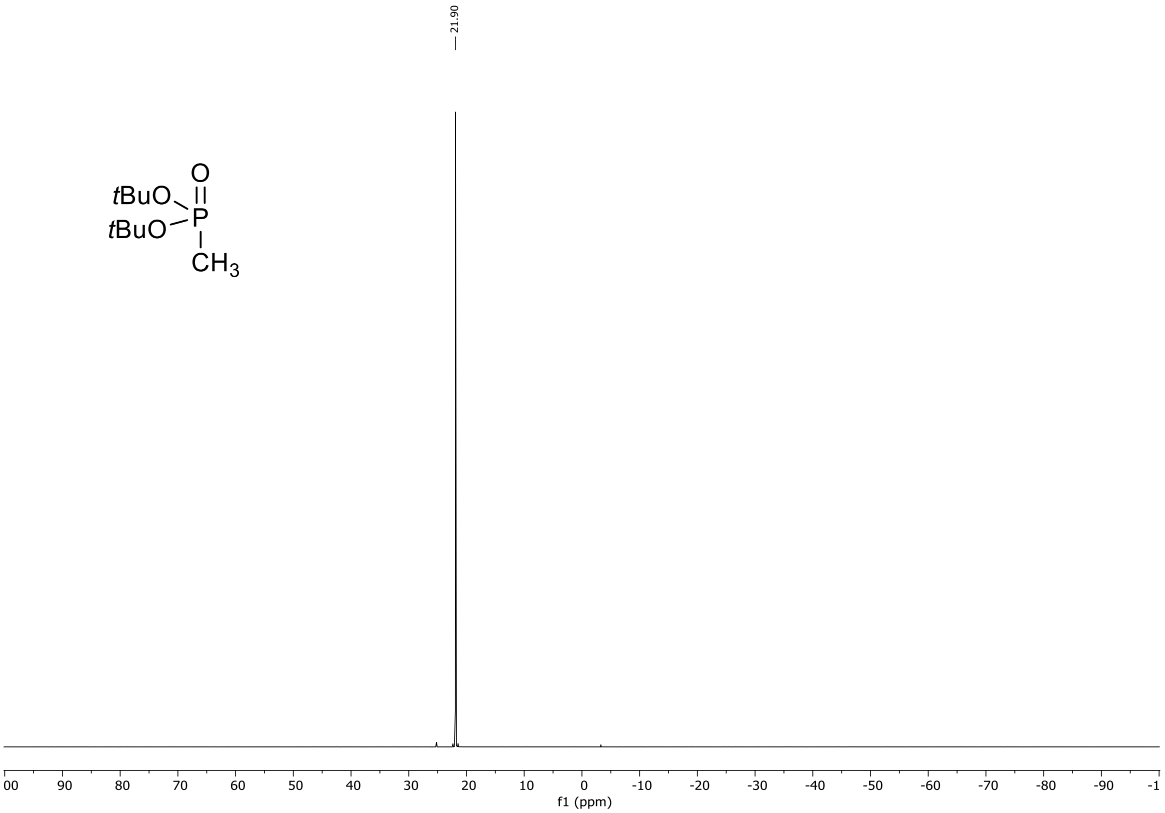

^{1}H NMR, 400 MHz, $CDCl_3$ of compound **21**

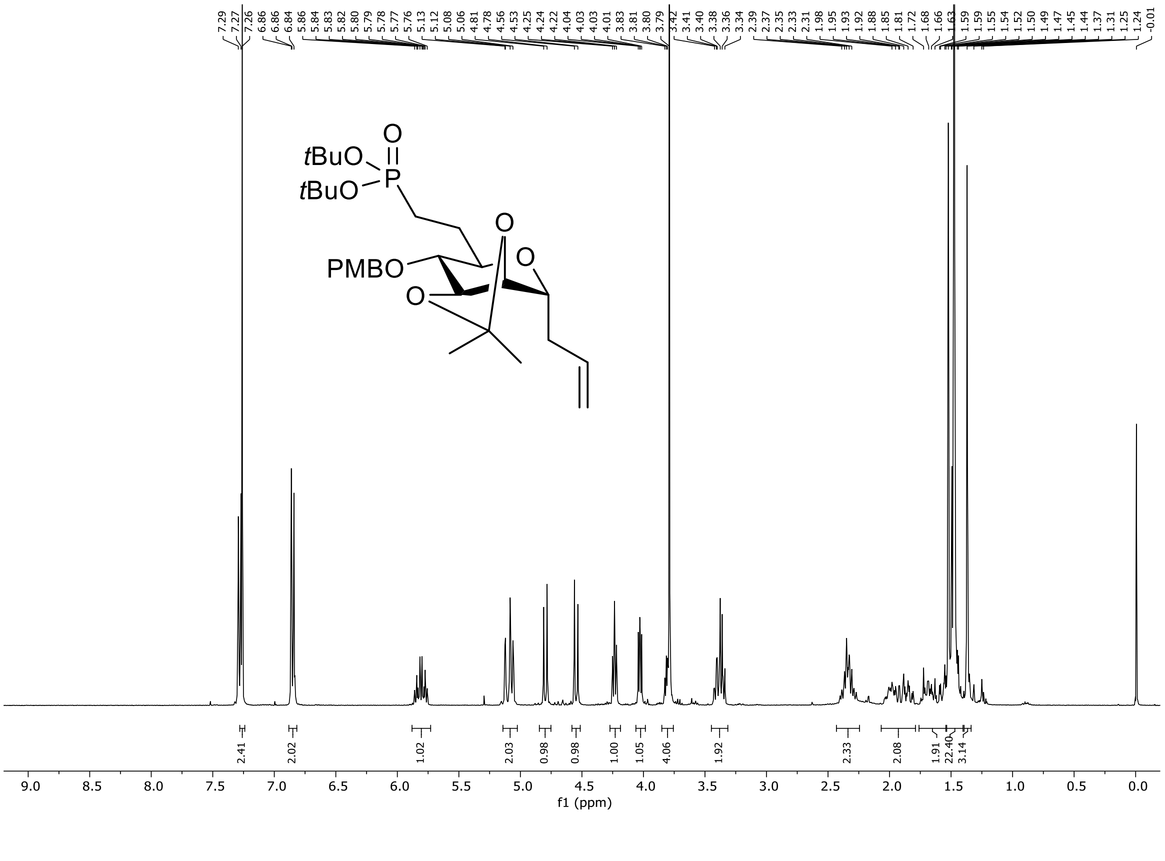

^{13}C-APT NMR, 101 MHz, $CDCl_3$ of compound **21**

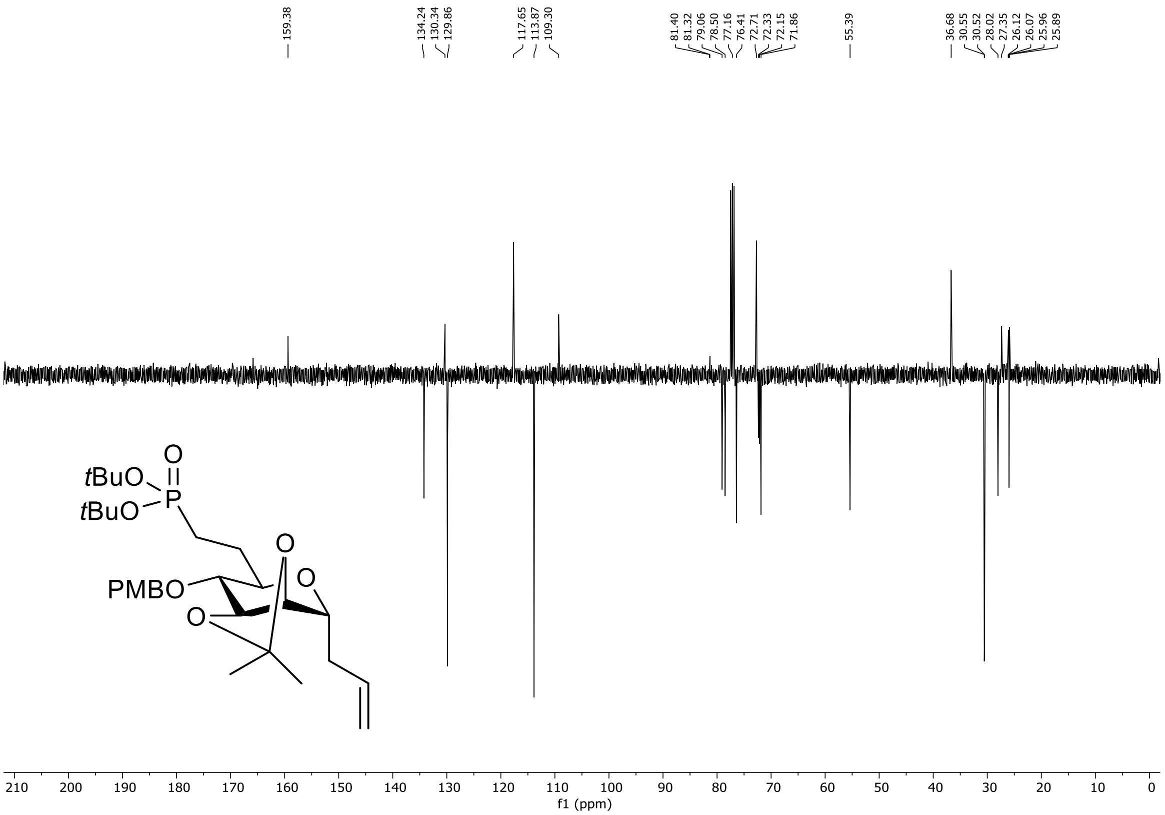

^{1}H-^{1}H COSY of compound **21**

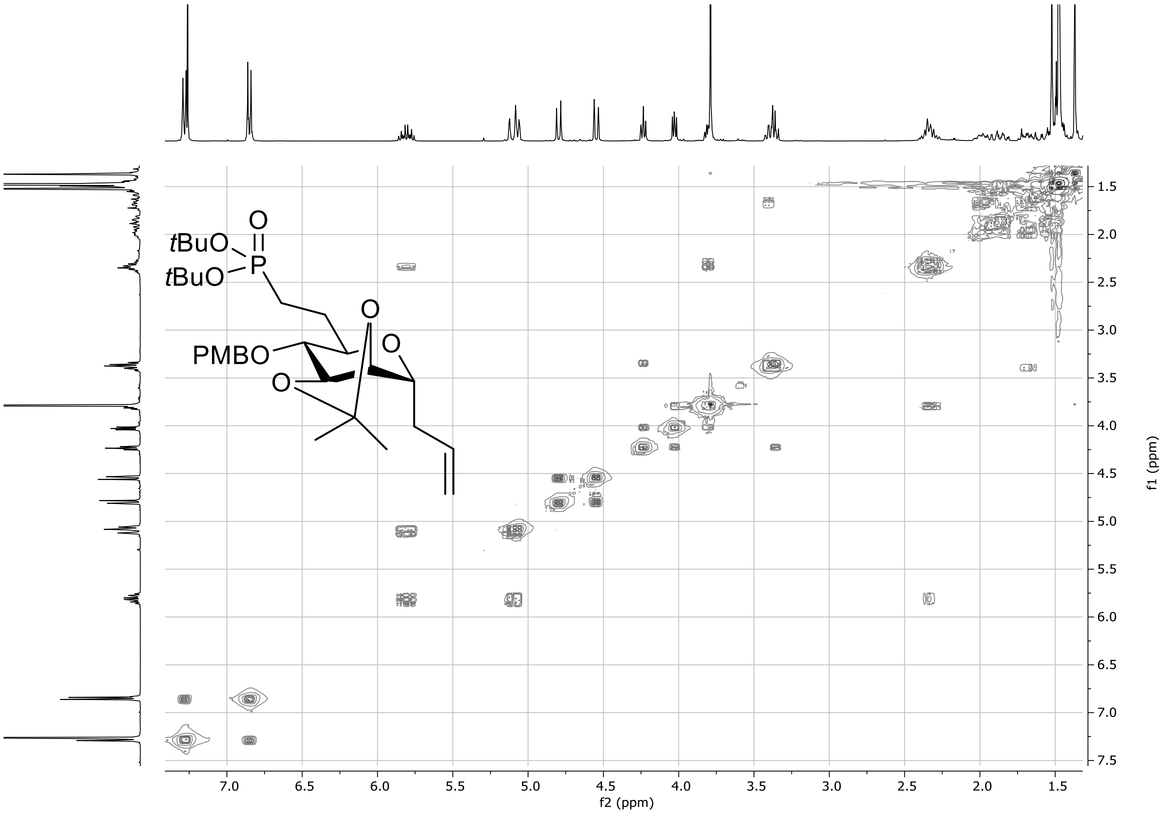

^{1}H-^{13}C HSQC of compound **21**

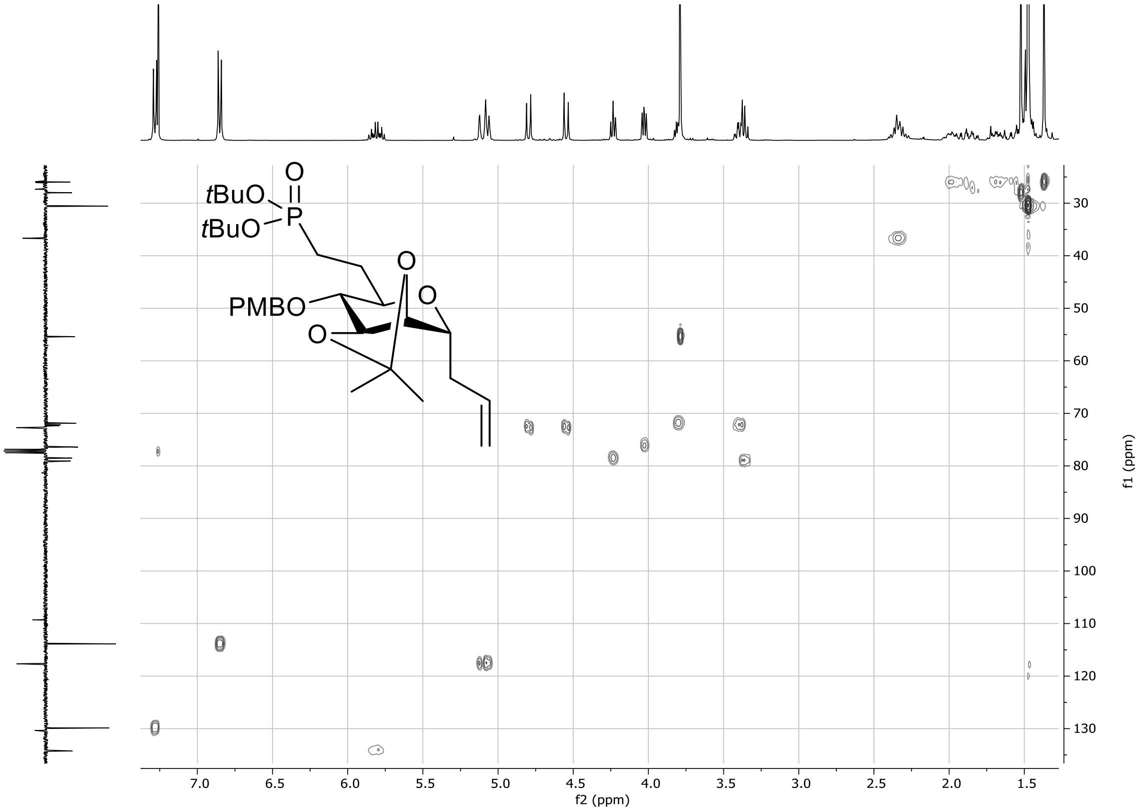

^{31}P NMR of compound **21**

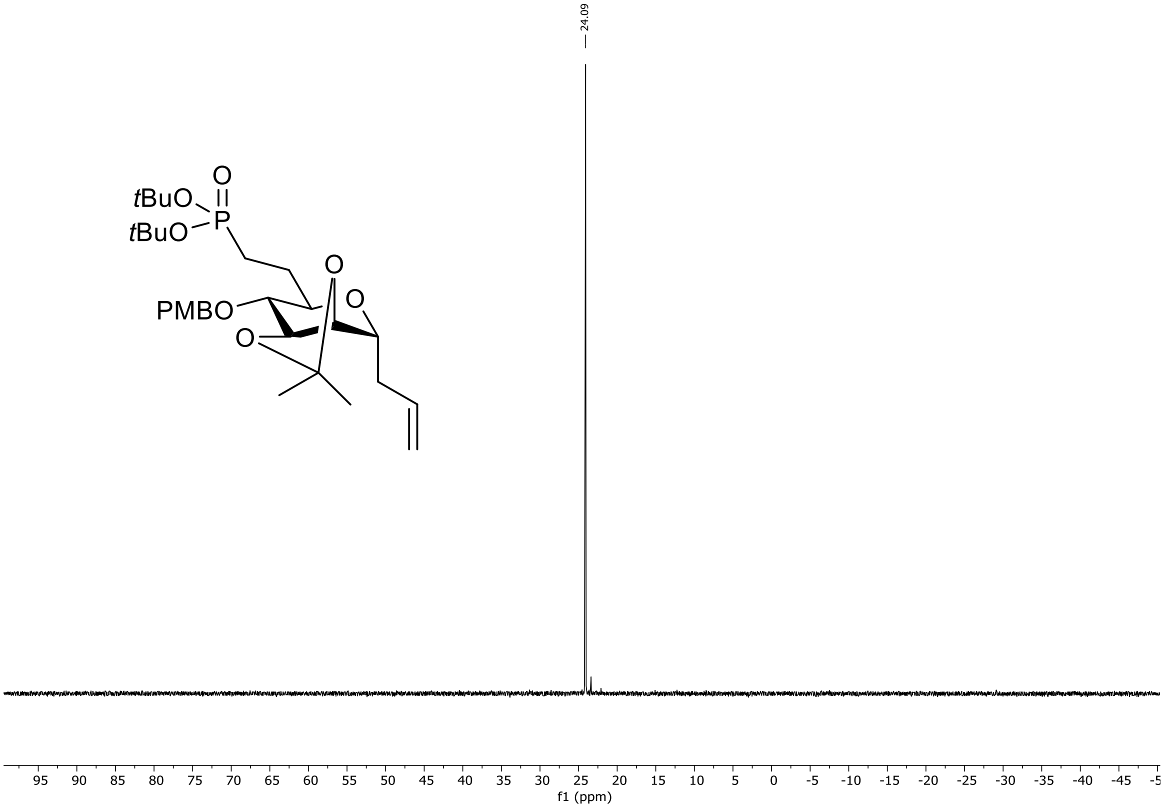

^{1}H NMR, 400 MHz, $CDCl_3$ of compound **22**

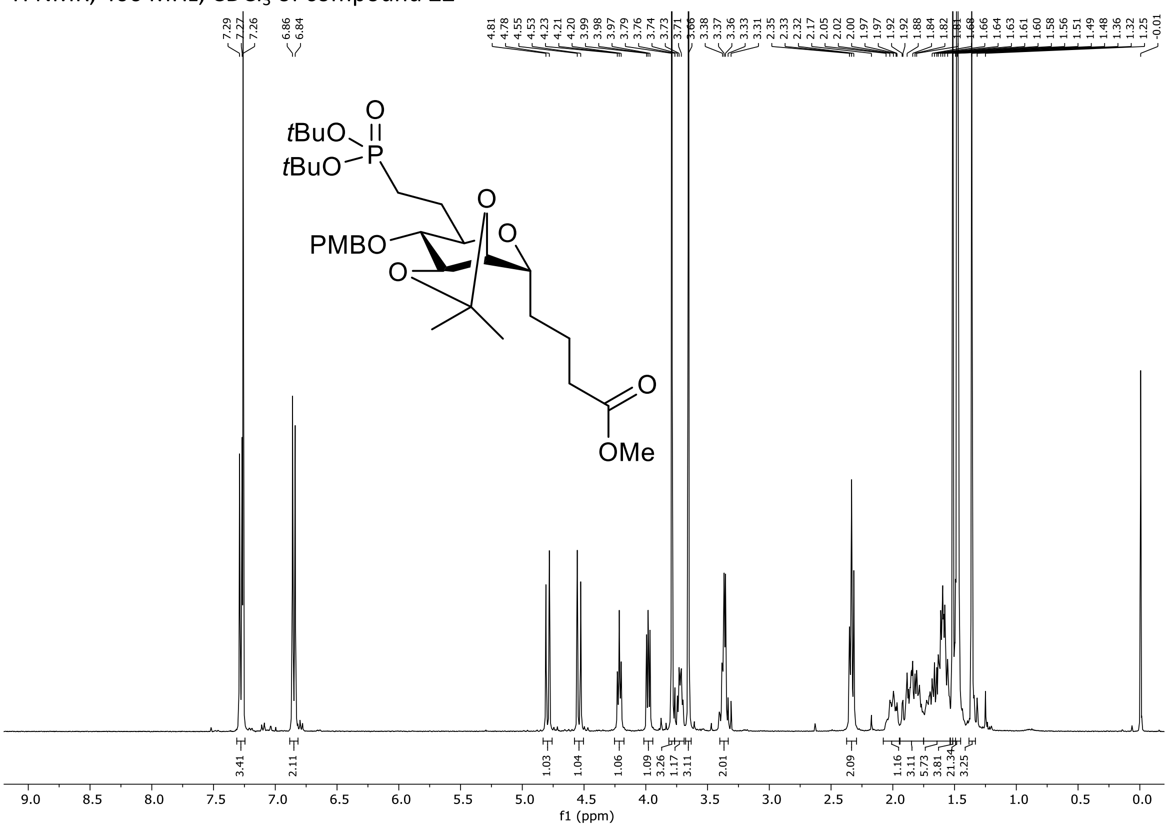

^{13}C-APT NMR, 101 MHz, $CDCl_3$ of compound **22**

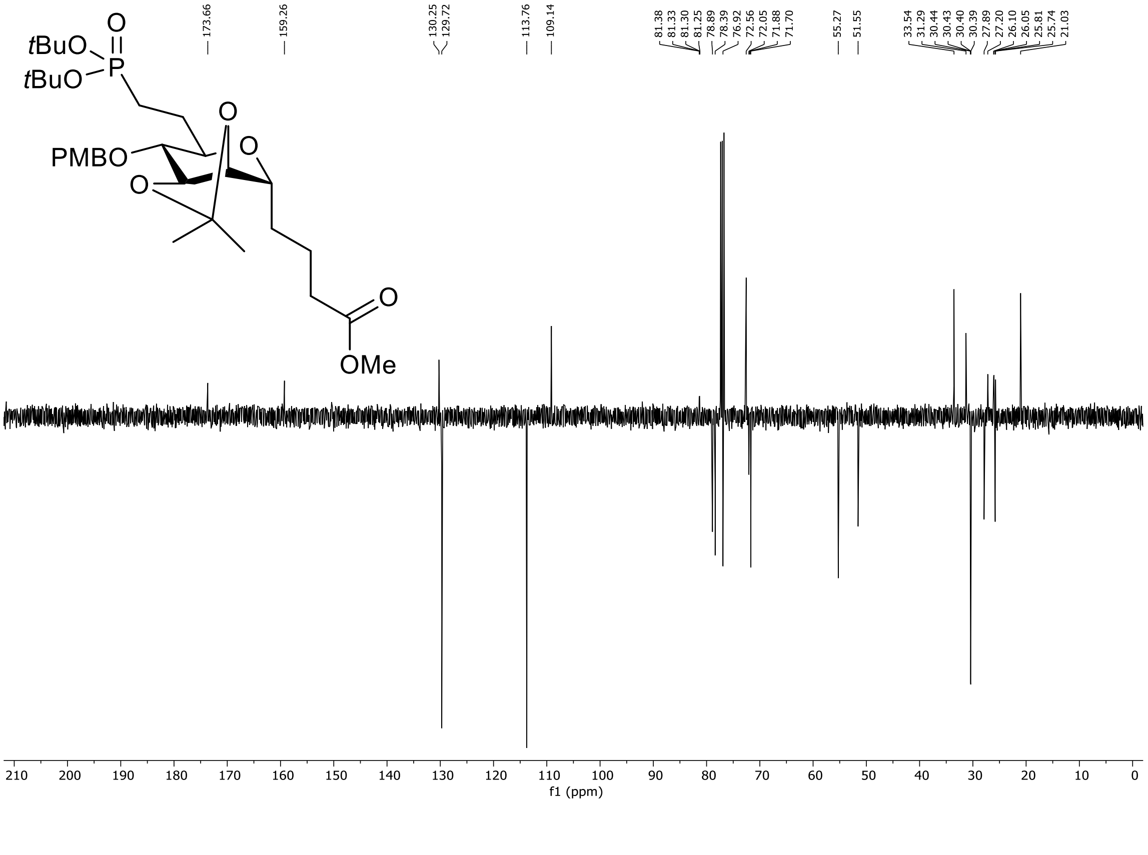

^{1}H-^{1}H COSY of compound **22**

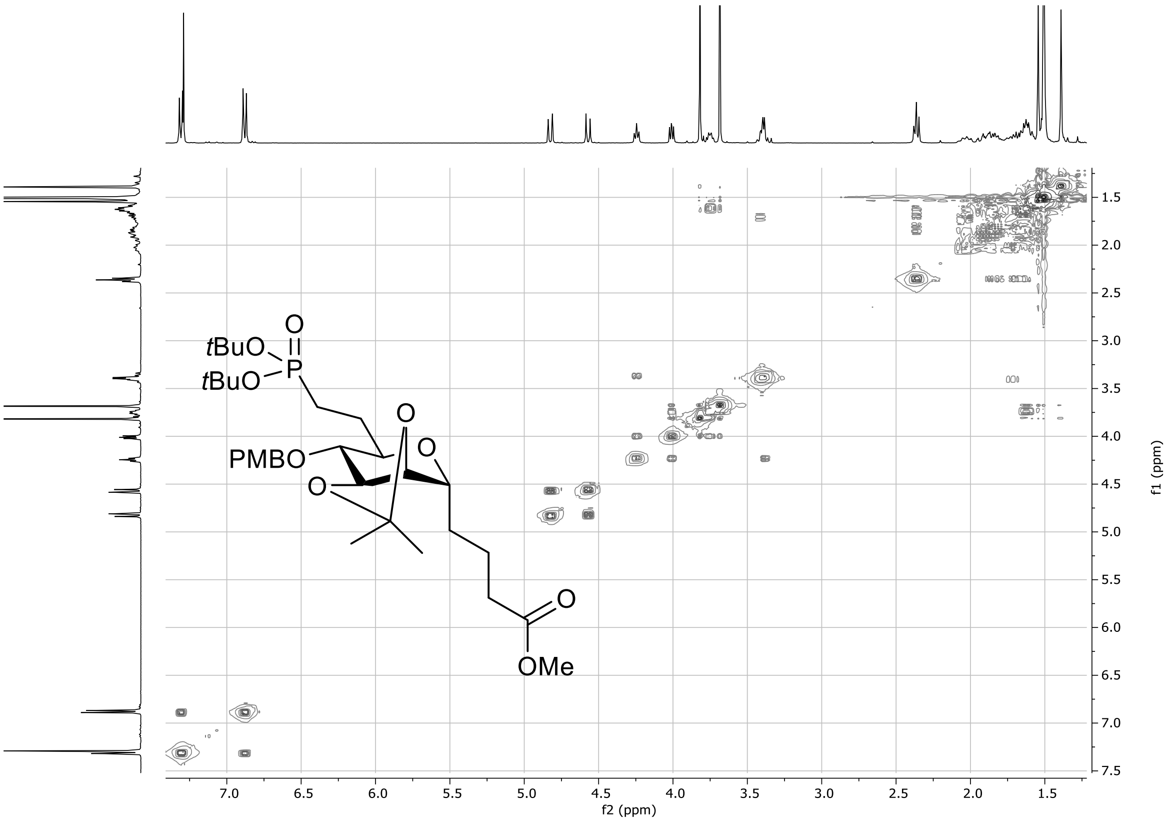

^{1}H-^{13}C HSQC of compound **22**

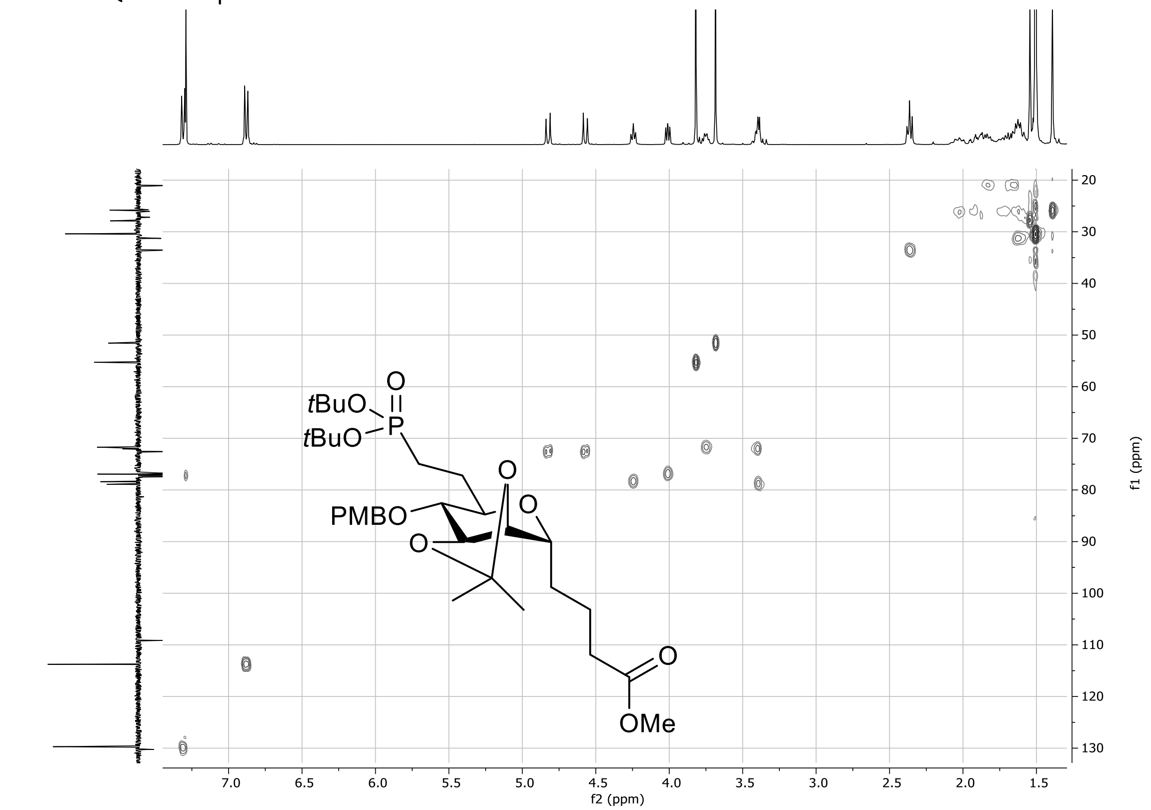

^{31}P NMR of compound **22**

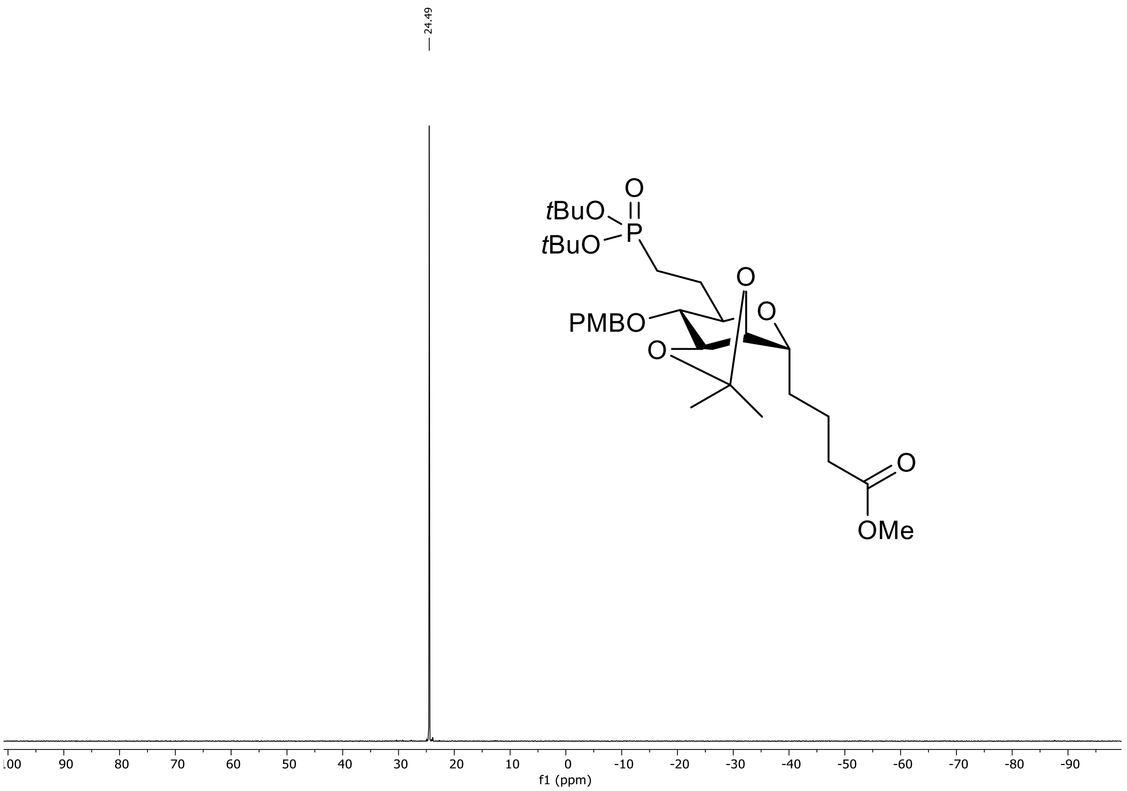

^{1}H NMR, 400 MHz, $CDCl_3$ of compound **23**

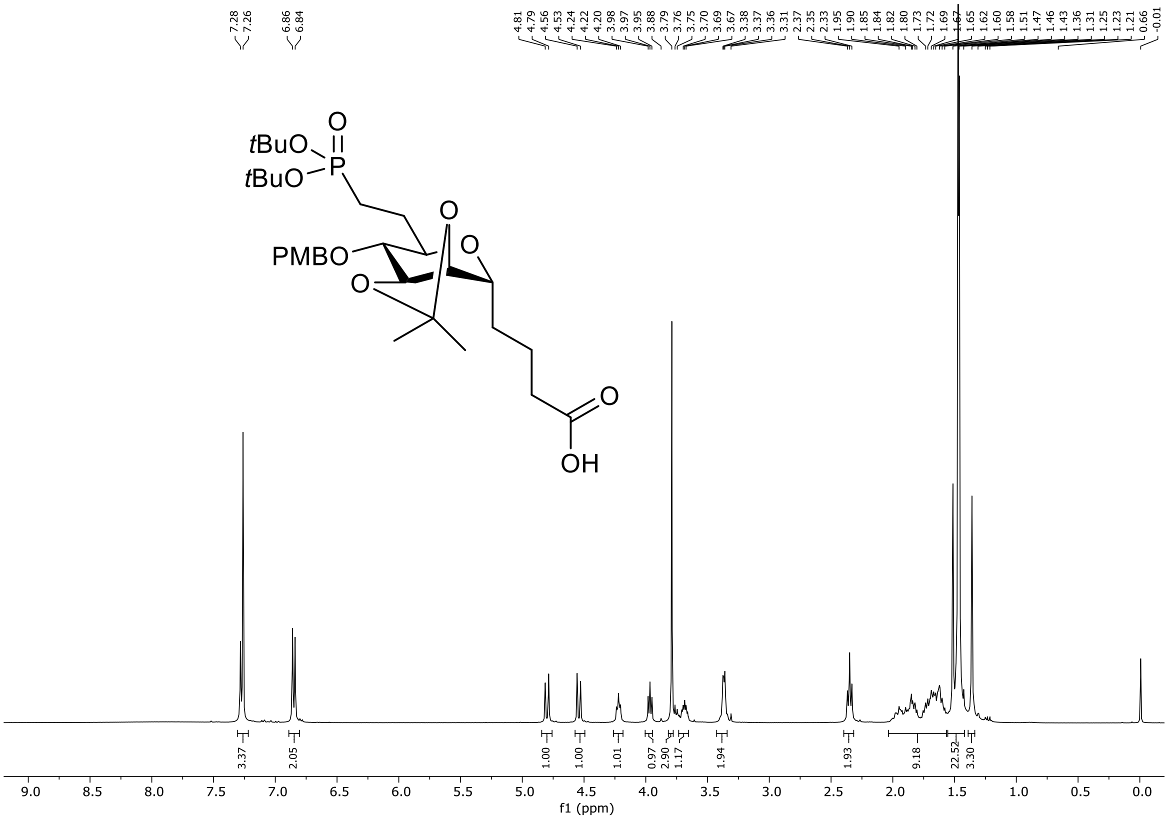

^{13}C-APT NMR, 101 MHz, $CDCl_3$ of compound **23**

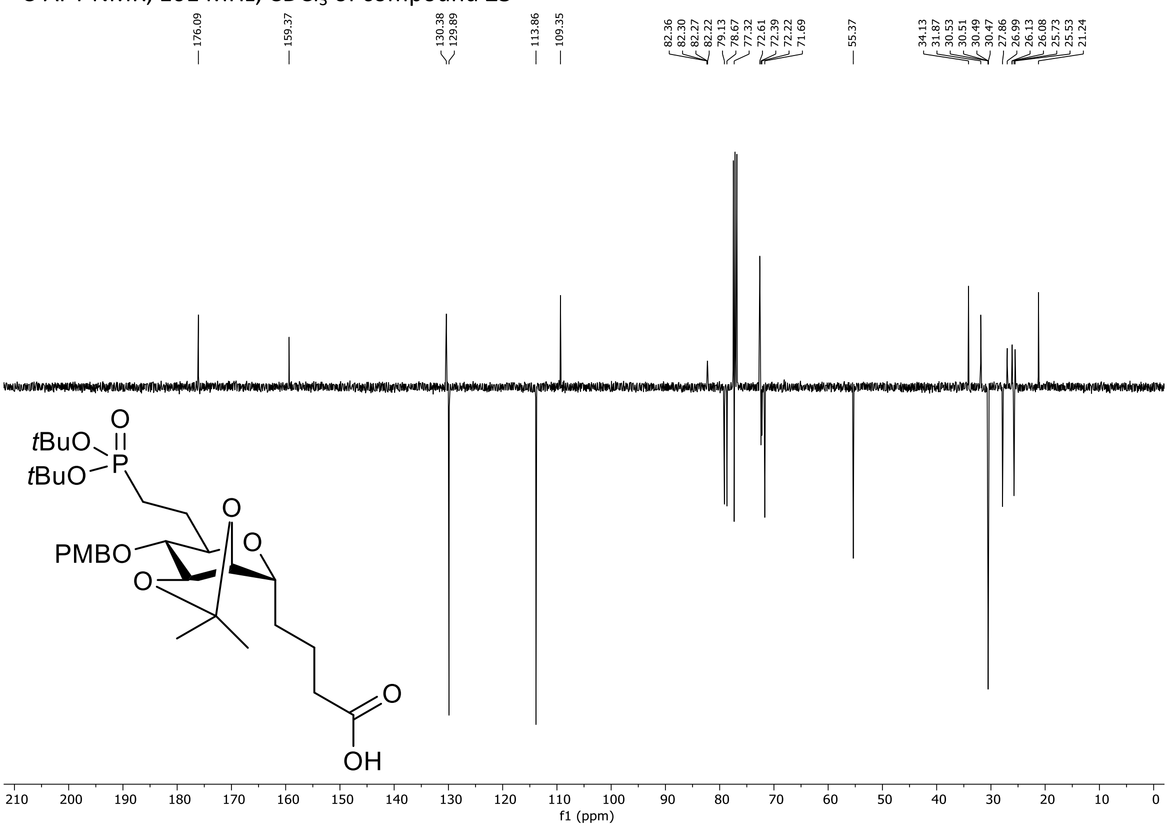

^{1}H-^{1}H COSY of compound **23**

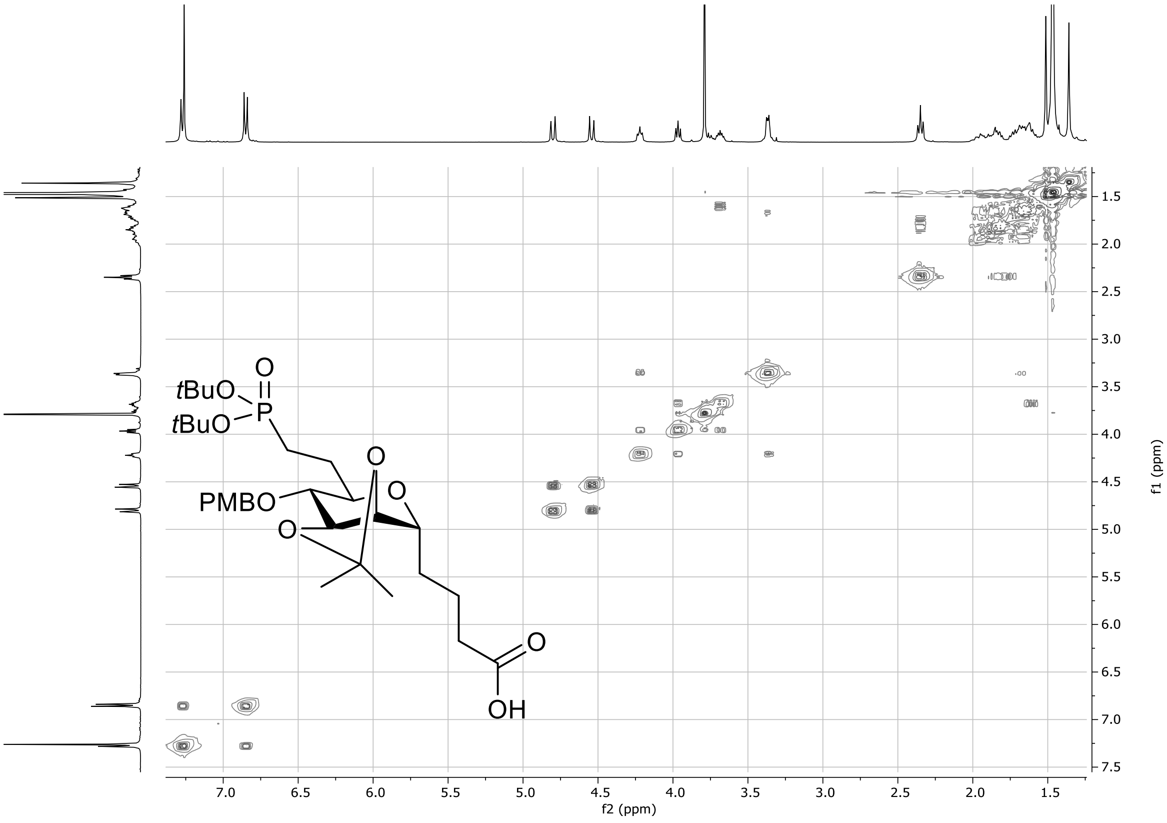

^{1}H-^{13}C HSQC of compound **23**

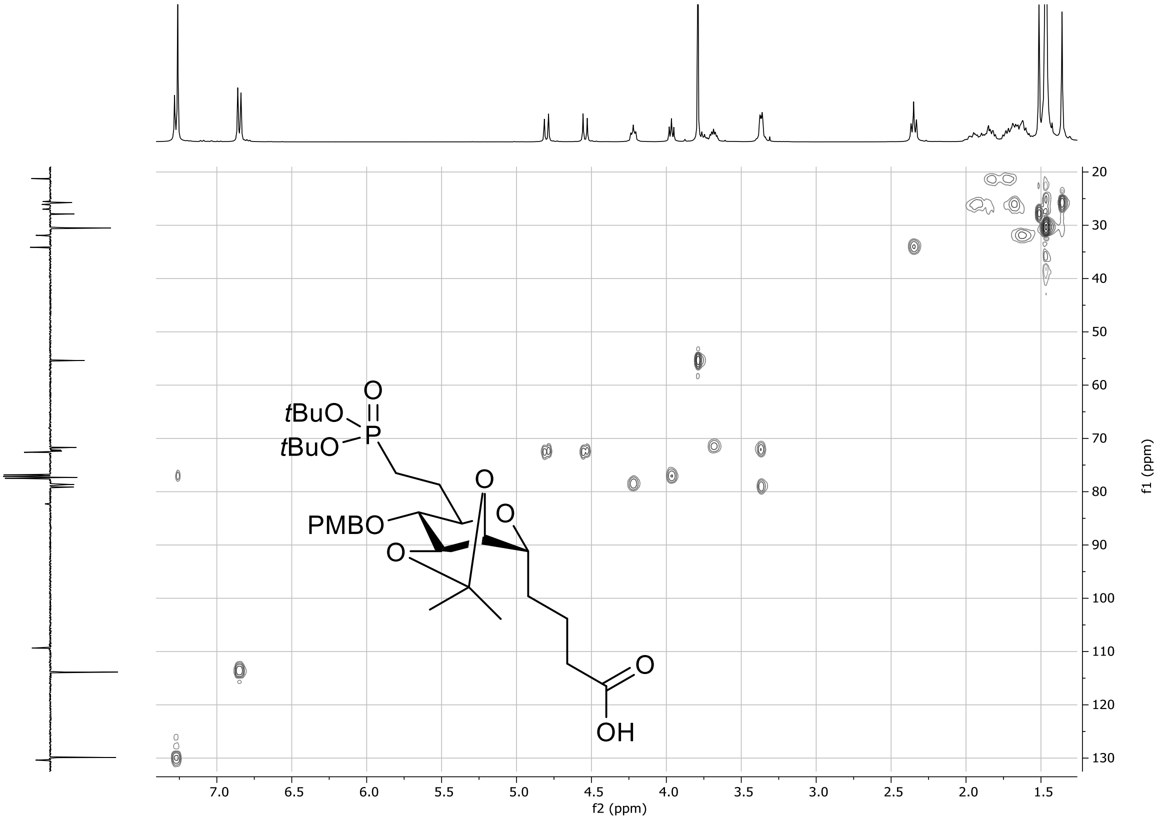

^{31}P NMR of compound **23**

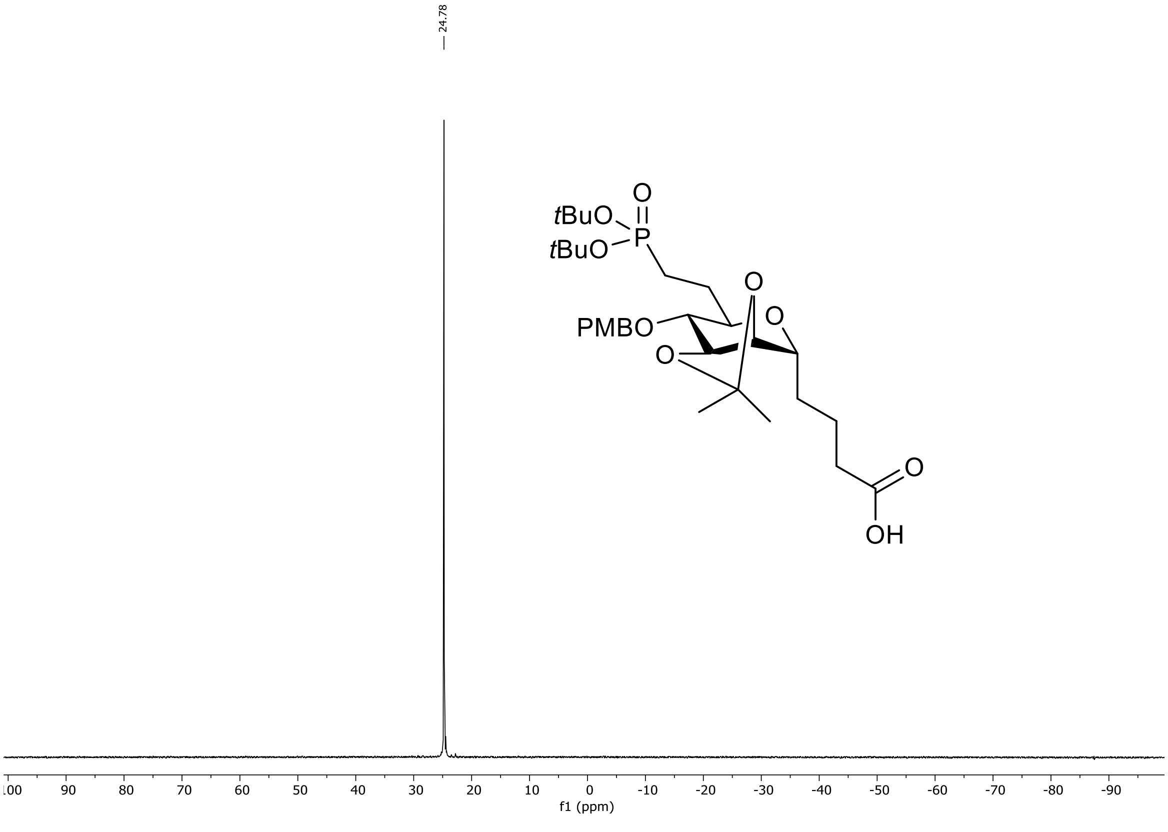

^{1}H NMR, 500 MHz, $CDCl_3$ of compound **24**

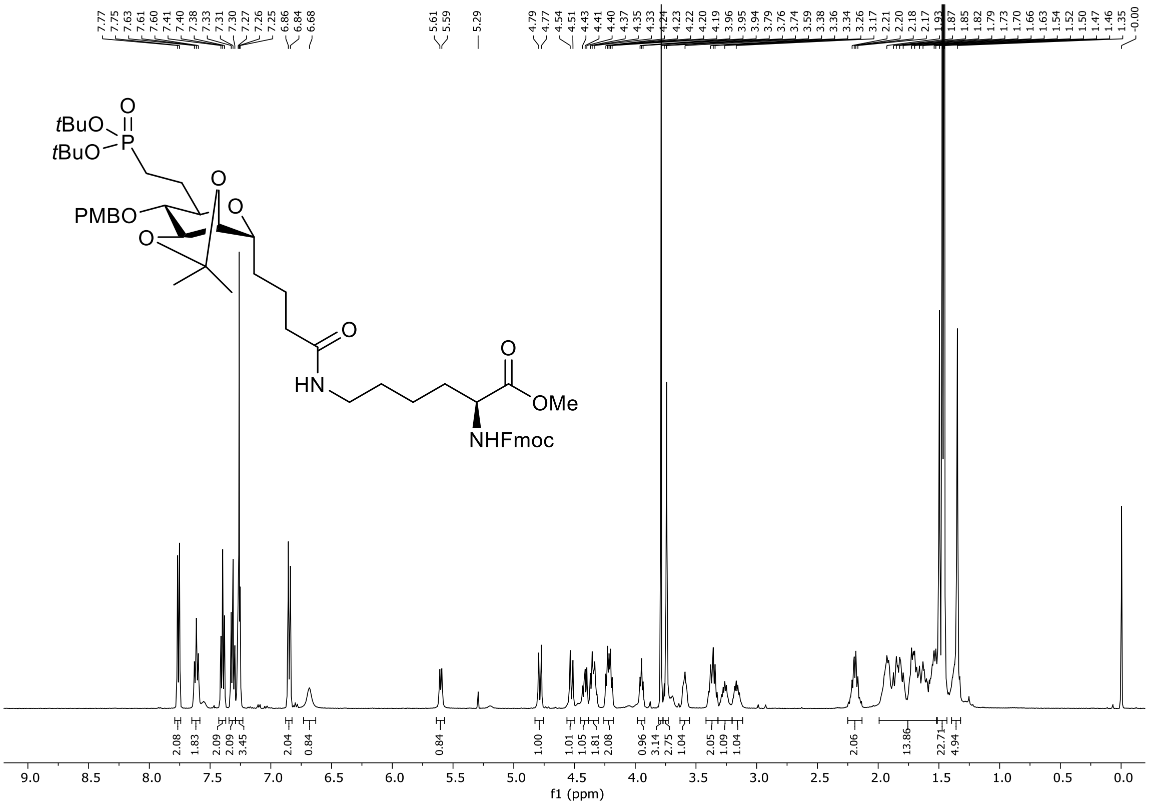

^{13}C-APT NMR, 126 MHz, $CDCl_3$ of compound **24**

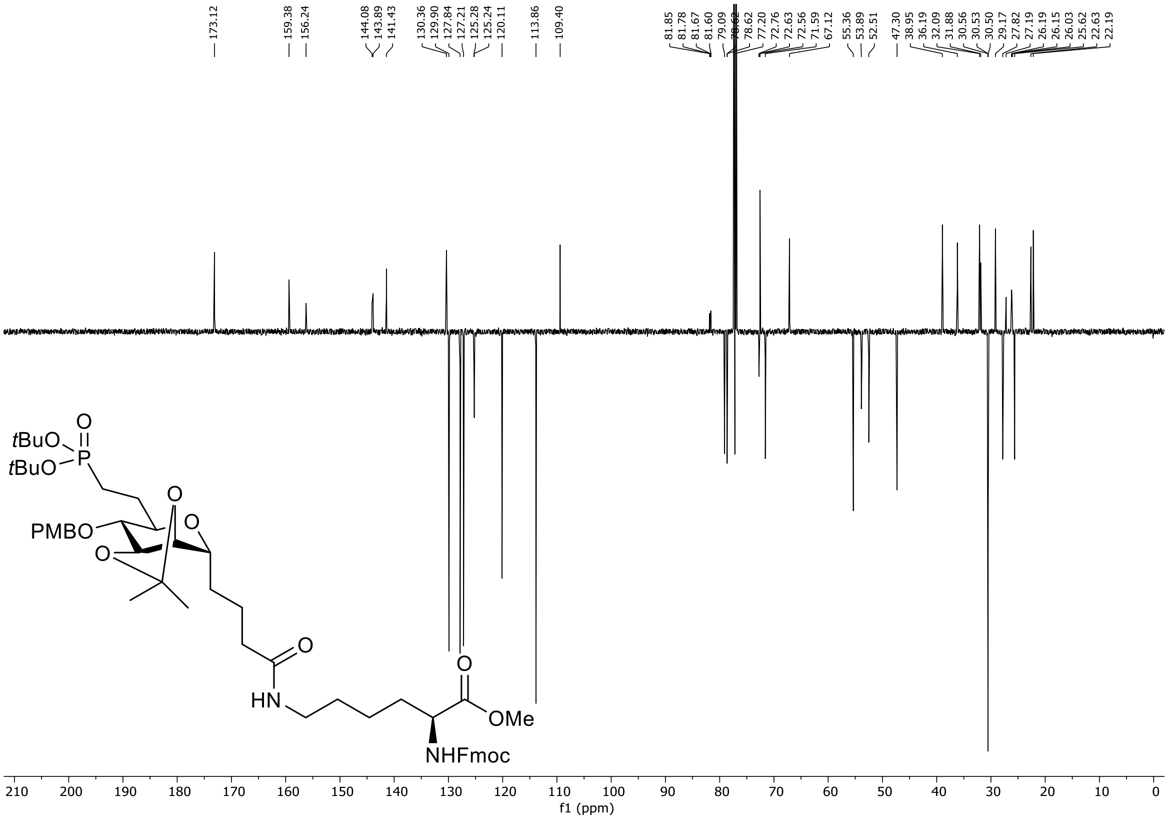

^{1}H-^{1}H COSY of compound **24**

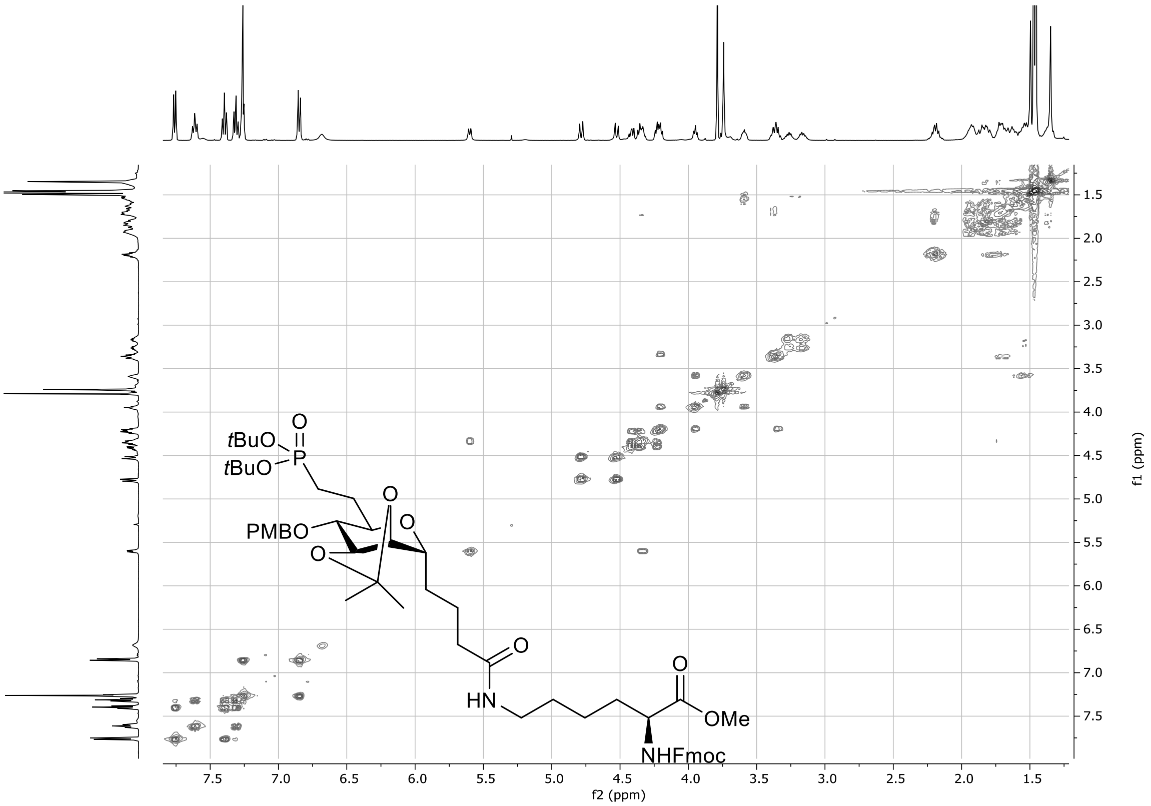

^{1}H-^{13}C HSQC of compound **24**

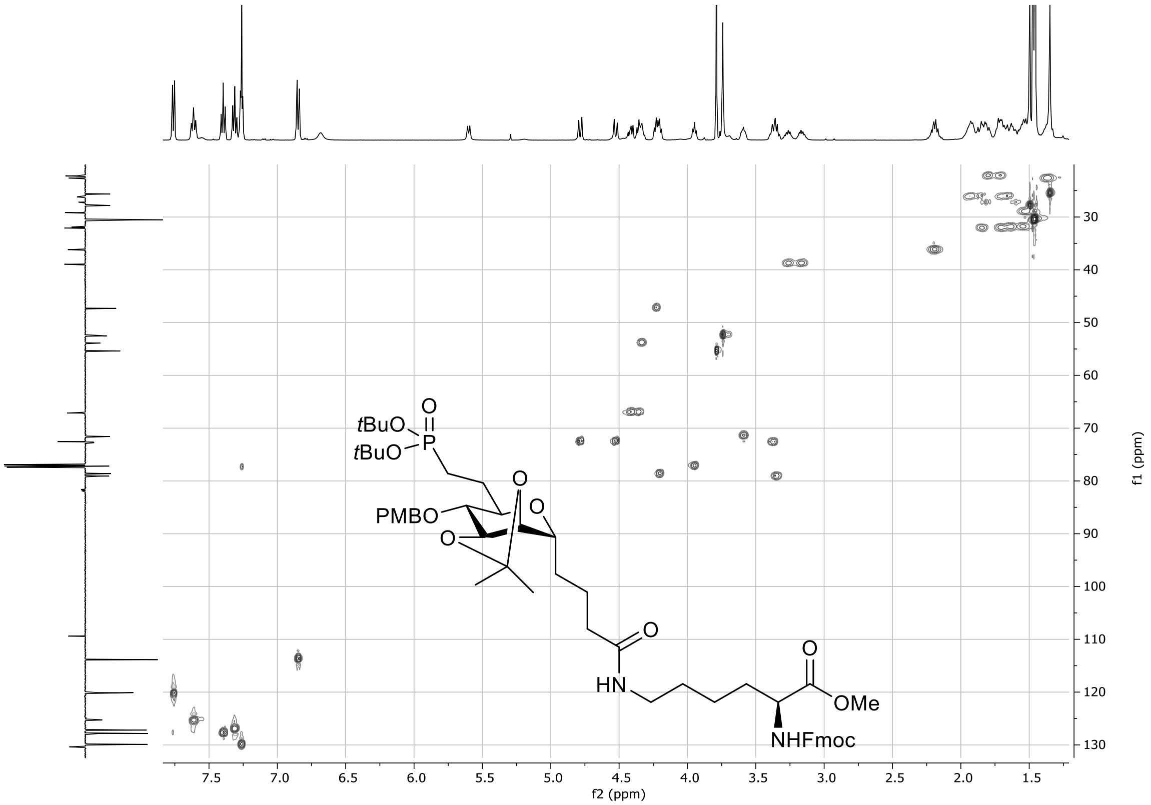

^{31}P NMR of compound **24**

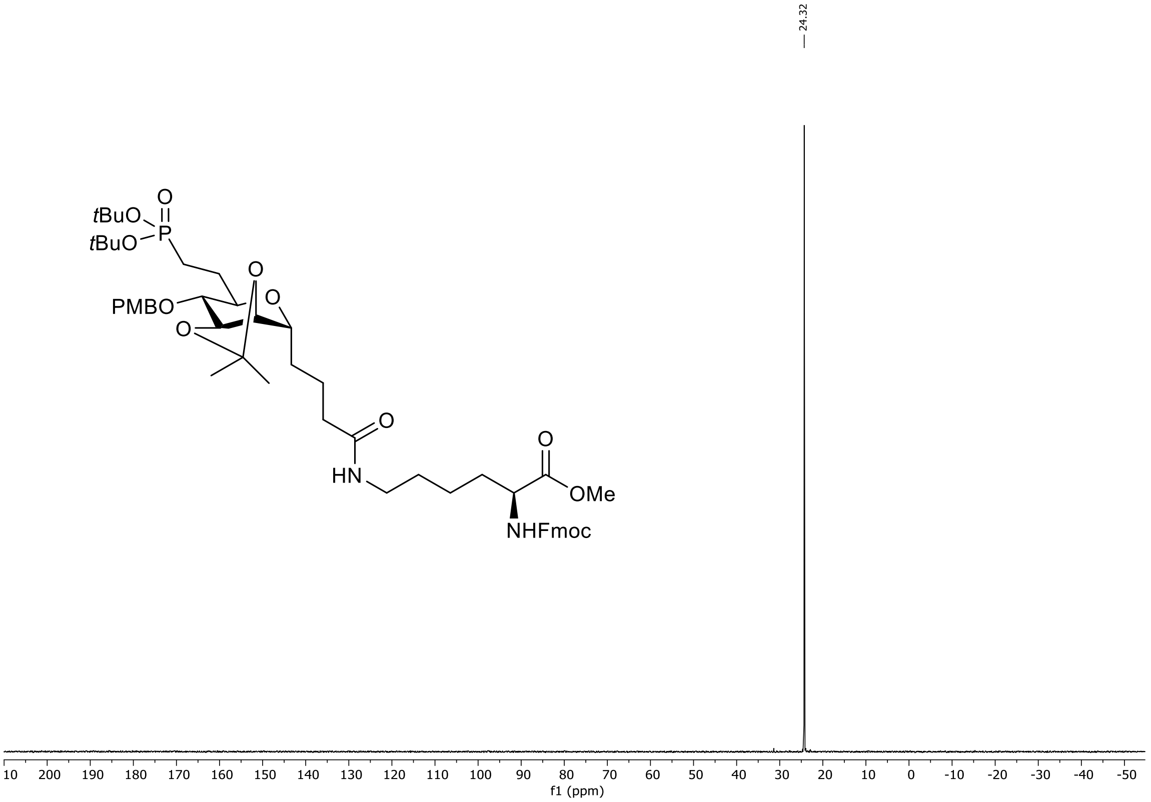

^{1}H NMR, 500 MHz, CD_3OD of compound **10**

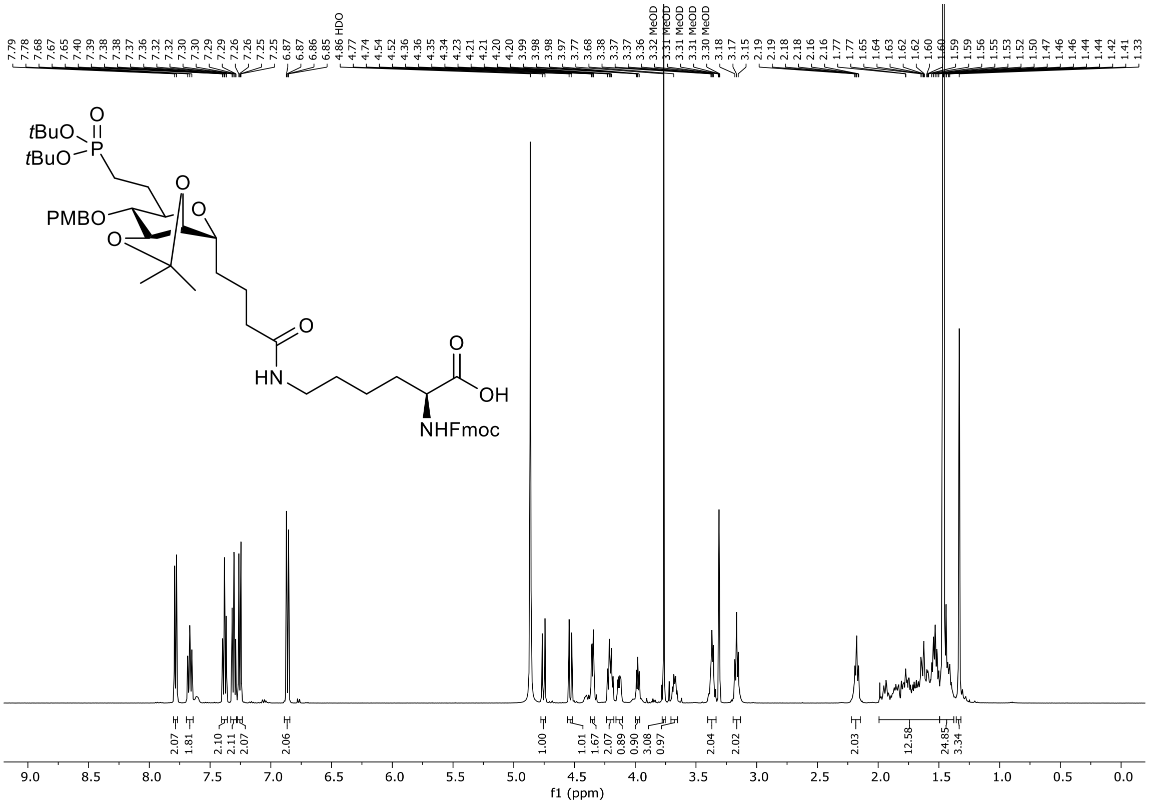

^{13}C-APT NMR, 126 MHz, CD_3OD of compound **10**

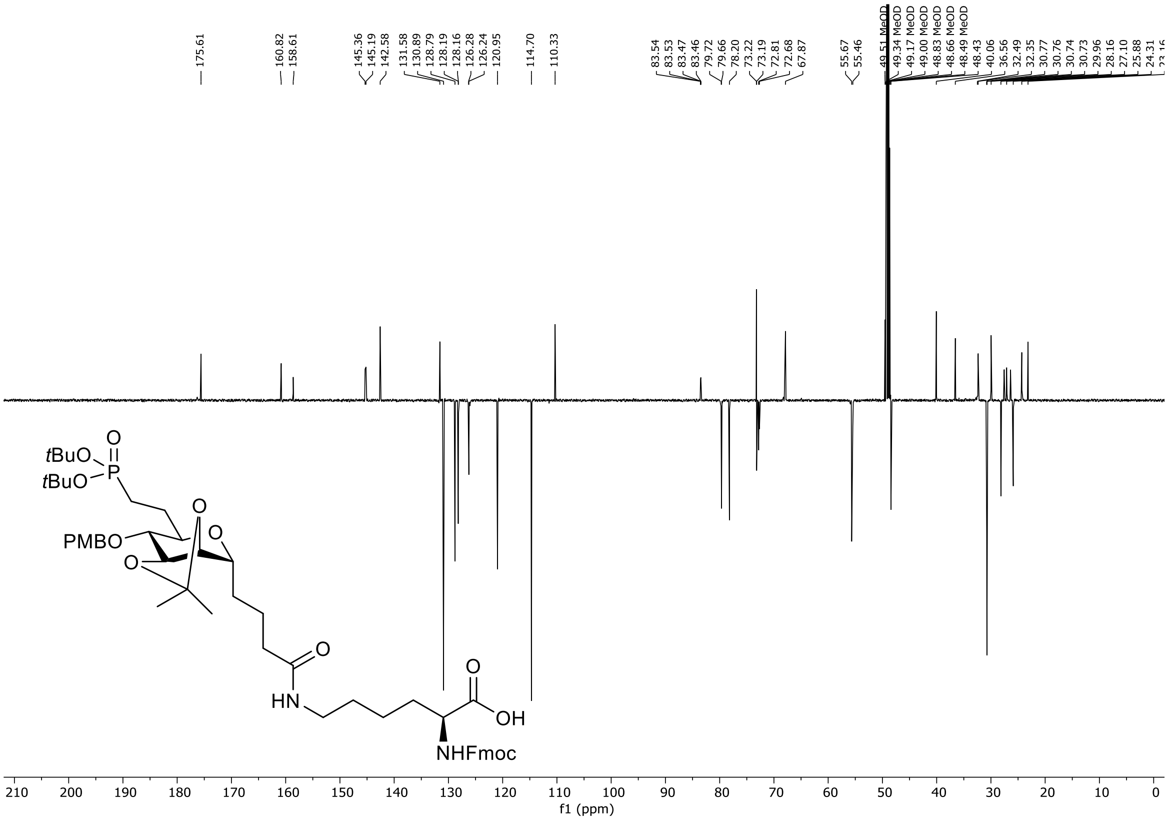

^{1}H-^{1}H COSY of compound **10**

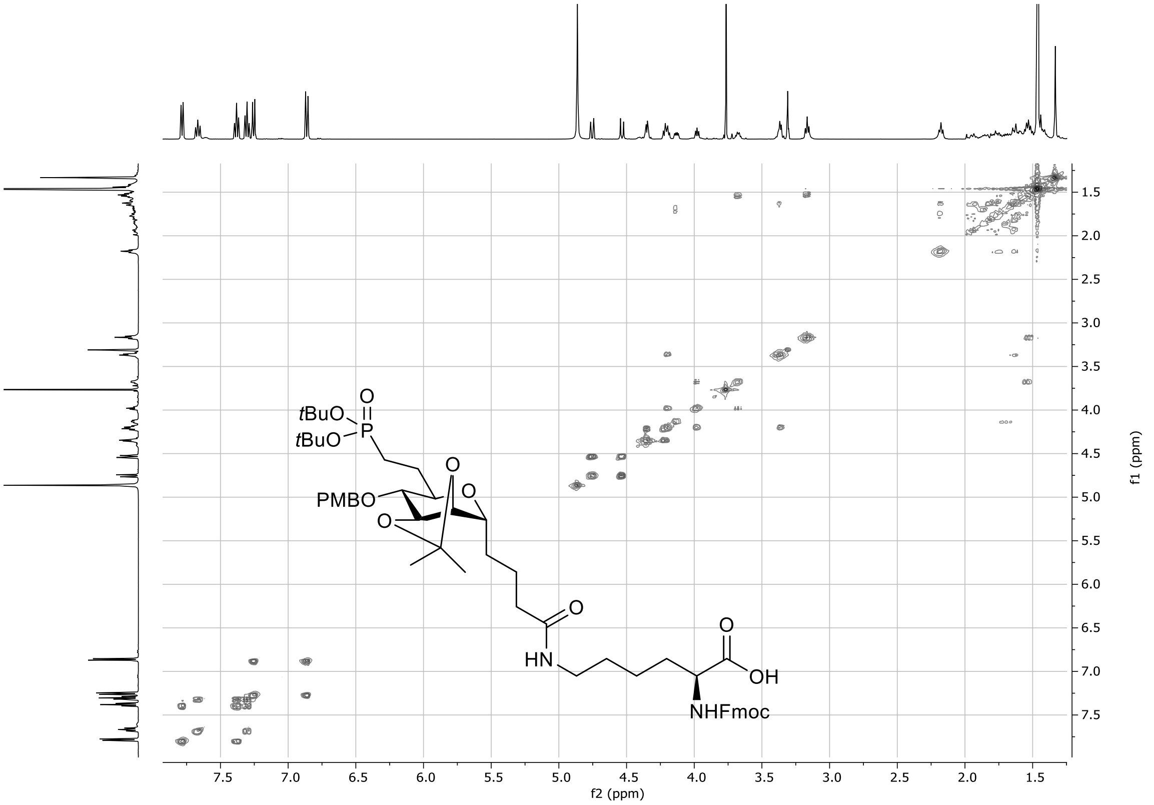

^{1}H-^{13}C HSQC of compound **10**

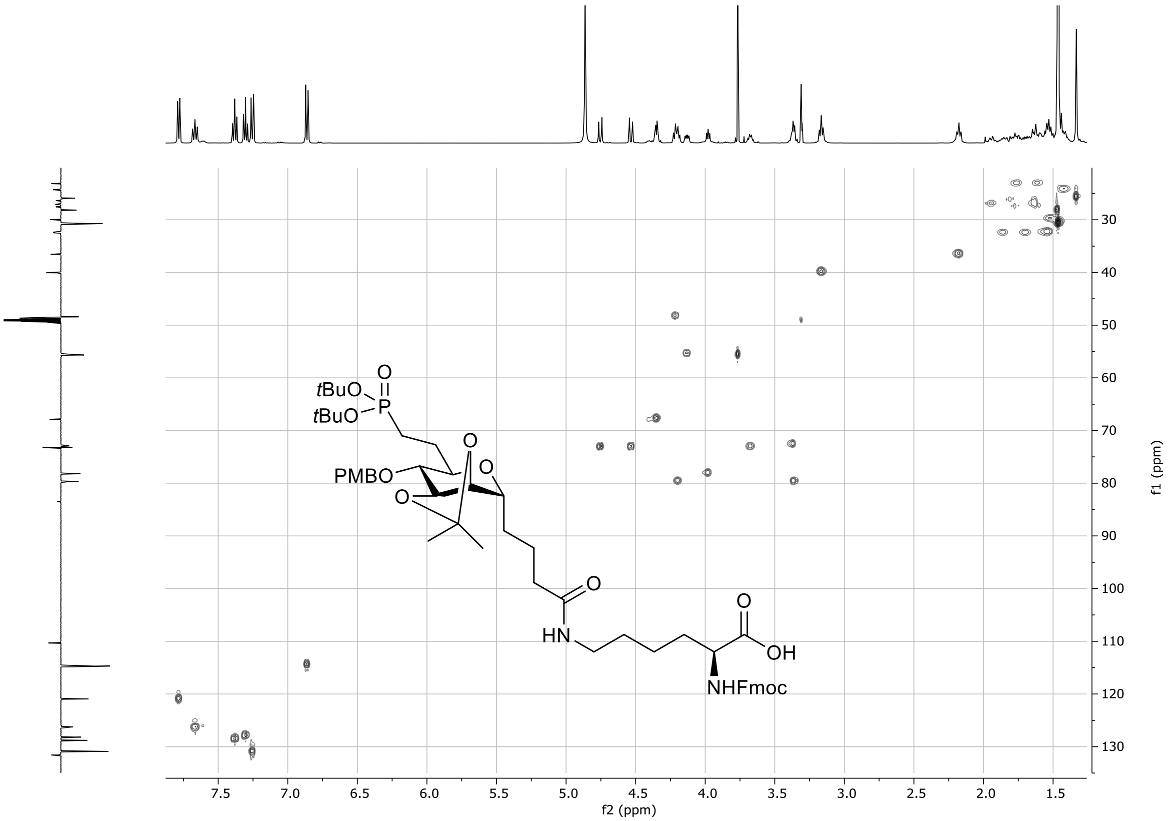

^{31}P NMR of compound **10**

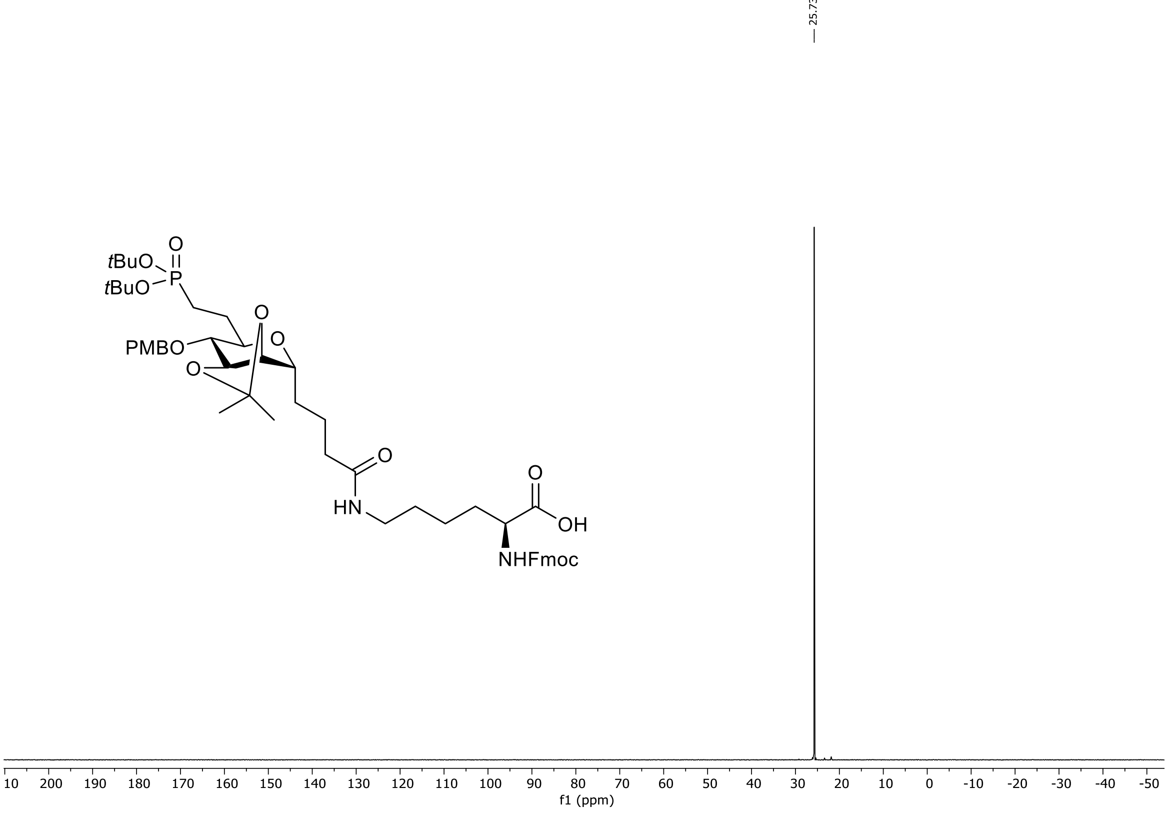

Supplement: Supplementary file 1 — Supplementary [file CBIC-22-434-s001.pdf]
